# Supplementary figures and images for: Transcriptome-wide characterization and functional analysis of MATE transporters in response to aluminum toxicity in Medicago sativa L
Source: PeerJ. 2019 Jan 31;7:e6302. doi: 10.7717/peerj.6302 (PMC6360082; doi:10.7717/peerj.6302)

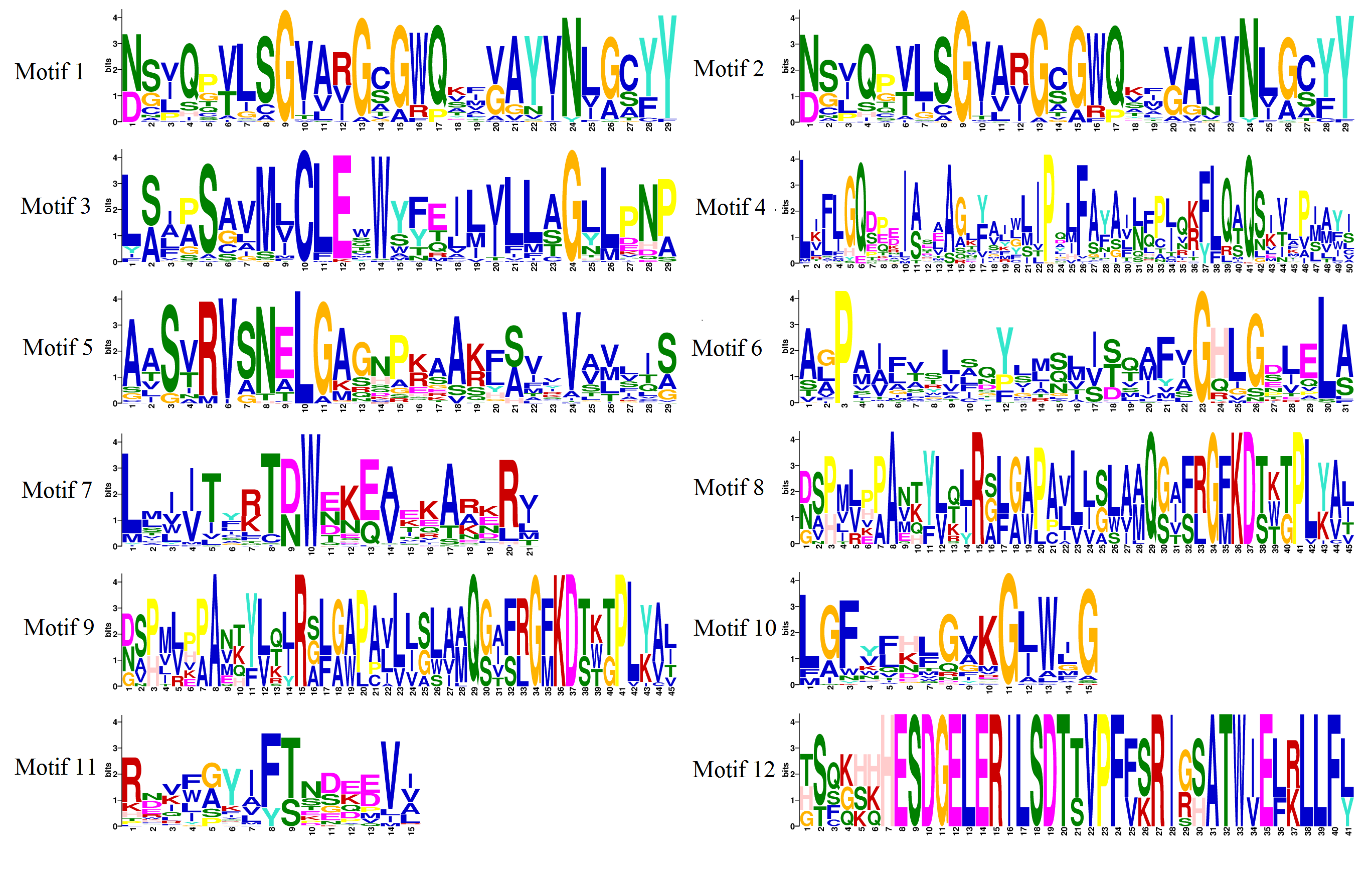

Supplement: Supplemental Information 7 [file peerj-07-6302-s007.png]

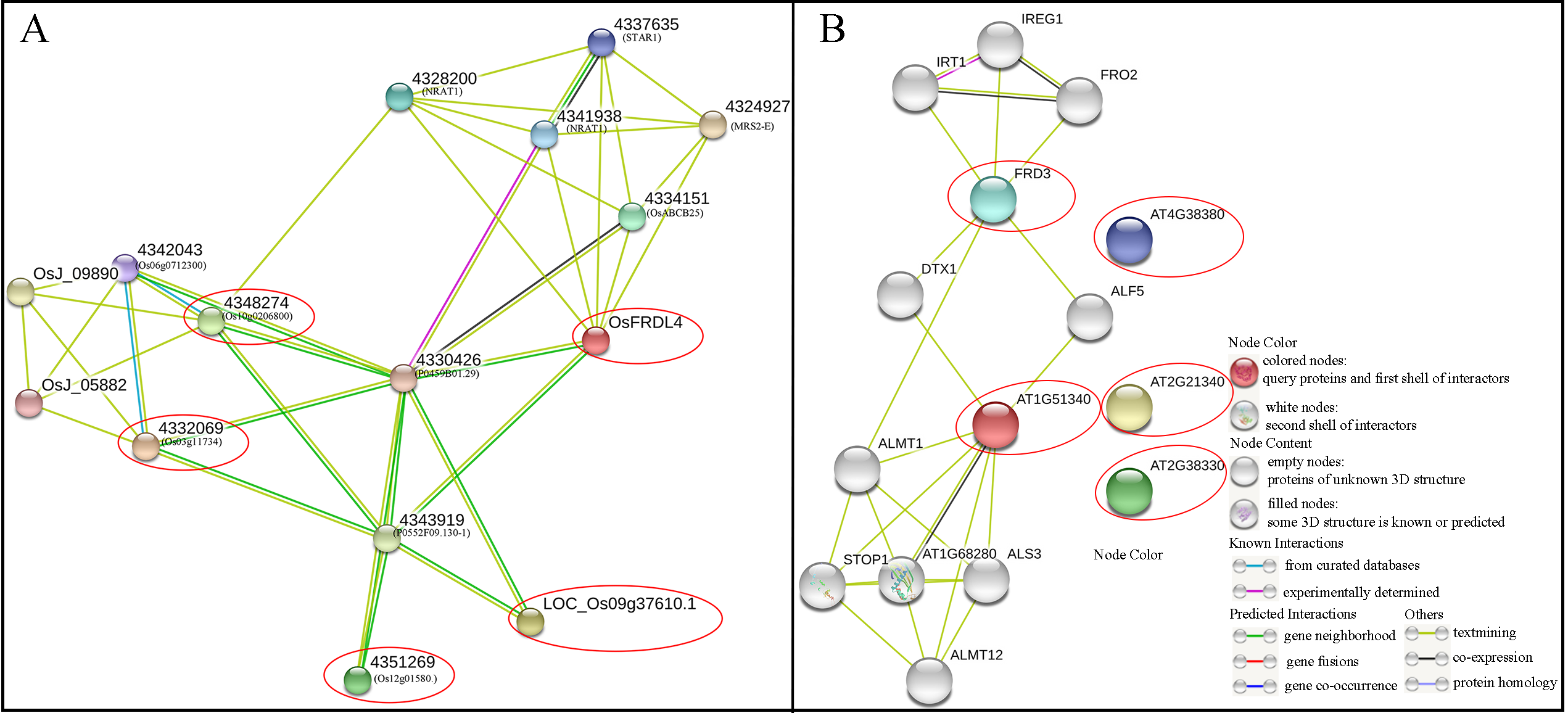

Supplement: Supplemental Information 8 [file peerj-07-6302-s008.png]
