# Supplementary material for: Transcriptome-wide characterization and functional analysis of MATE transporters in response to aluminum toxicity in Medicago sativa L
Source: PeerJ. 2019 Jan 31;7:e6302. doi: 10.7717/peerj.6302 (PMC6360082; doi:10.7717/peerj.6302)
Supplement: Supplemental Information 9 [file peerj-07-6302-s009.doc]

**Supplementary data 1.** The coding sequences and protein sequences of the 88 MsMATE members in alfalfa.

**The coding sequences of 88 MsMATEs**

>MsMATE01

ATGGAAGATAATGGTATCTCAAATAATGCAGTCAAGAACAAGTGGACAATGCCTCTATCAGTTTTCTTCAAAGATGCA

AGCCTTGTATTCAAGATGGATTCCCTAGCTAAGGAGATACTTGGGATTGCATTCCCATCTGCACTTGCTGTTGCTGCT

GATCCAATTGCTTCTCTTATAGACACAGCATTCATAGGTCACTTAGGGCCGGTCGAACTTGCGGCGGCAGGAGTTTCC

ATTGCTGTATTCAACCAAGCTTCAAGGATTACCATTTTCCCTTTGGTCAGTATTACAACTTCTTTTGTAGCTGAAGAA

GATACTATGGATAGAATCAATAGCAAAGCAGCAGAAAAGCAGTTCAATGAAGGCATTAAGGCCAAATCGAATGAAGTC

ATGCCTGATGATCATTTGCTTCAAGACATAGAAGCCGGTGCAATCAAACAGGATGGTACCTTGAAAAATGAGACGAAA

AATGGAGATGATGCAAATTCAAATGTAAGCAAGTCTTCCATTGTTACTAATAGTGGTAACAAGAGTGAGTCAAAACCT

GTAAGGAAGAAGAGGCACATTGCTTCAGCATCTACCGCATTACTTTTCGGCACAGTCCTTGGCCTTATTCAAGCTGCA

ACCCTTATATTTGCAGCTAAACCTCTATTAGGTGCAATGGGTTTAAAATATGATTCTCCTATGCTTGTCCCAGCTGTT

AAGTACTTAAGATTGAGAGCCTTAGGTGCTCCTGCAGTGCTTCTCTCCTTGGCCATGCAAGGAATCTTTAGAGGGTTC

AAAGACACAACAACTCCTTTATATGTCATTGTTTCCGGGTATGCATTGAATGTCGCTATGGATCCACTACTCATATTT

TACTTCAAATTAGGCATCAGAGGTGCAGCCATTTCACATGTGCTCTCTCAGTACATTATGGCAAGTTTACTCTTGTTT

ATATTAATGAAAAAAGTGGATCTCCTACCTCCAAGCATGAAGGATTTGCAGATTTTCAGGTTTCTTAAAAATGGTGGT

CTATTGTTGGCCAGAGTTATAGCAGTGACATTCTGTGTGACCTTATCAGCCTCATTAGCAGCAAGGTTAGGTCCAATT

CCAATGGCTGCATTCCAAACCTGCCTCCAGGTTTGGATGACATCCTCCCTTCTCGCTGATGGTTTAGCTGTTGCAATA

CAGGCAATTCTAGCATGTTCCTTTGCTGAGAAAGACTATAATAAAGTAACTACTGCTGCAACAAGGACACTGCAAATG

AGTTTTGTTTTAGGAGTTGGACTCTCTCTAGTAGTTGGAGGTGGATTATACTTTGGAGCCGGAGTATTTTCCAAAAAT

GTTGCTGTTATTCACCTAATCAGACTAGGCCTCCCGCCTTTGTCTTTGATGGTGTGA

>MsMATE02

ATGGAAGATAATGGTATCTCAAATAATGCAGTCAAGAACAAGTGGACAATGCCTCTATCAGTTTTCTTCAAAGATGCAAG

CCTTGTATTCAAGATGGATTCCCTAGCTAAGGAGATACTTGGGATTGCATTTCCATCTGCACTTGCTGTTGCTGCTGATC

CAATTGCTTCTCTTATAGACACAGCATTCATAGGCCACTTAGGGCCGGTGGAACTTGCGGCGGCAGGAGTTTCCATTGCT

GTATTCAACCAAGCTTCAAGGATTACCATTTTCCCTTTGGTCAGTATTACAACTTCTTTTGTAGCTGAAGAAGATACTAT

GGATAGAATCAATAGCAAAGCAGCAGAAAAGCAGTTCAATGAAGGCATTAAGGCCAAATCGAATGAAGTCATGCCTGATG

ATCATTTGCTTCAAGACATAGAAGCAGGTGCAACCAAACAGGATAGTACCTTGAAAAATGAGACAAAAAATGGAGATGAT

GCAAATTCAAATGTAAGCAAGTCTTCCATTGTTACTAATAGTGGTAACAAGAGTGAGTCAAAACCTATAAGGAAGAAAAG

GCACATTGCTTCAGCATCTACCGCATTACTTTTCGGCACAGTCCTTGGCCTTATTCAAGCTGCAACCCTTATATTTGCAG

CTAAACCTCTATTAGGTGCAATGGGTTTAAAATATGATTCTCCTATGCTTGTCCCAGCTGTTAAGTACTTAAGATTGAGA

GCCTTAGGTGCTCCTGCAGTGCTTCTCTCCTTGGCCATGCAAGGAATCTTTAGAGGGTTCAAAGACACAACAACTCCTTT

ATATGTCATTGTTTCCGGGTATGCATTGAATGTCGCTATGGATCCACTACTCATATTTTACTTCAAATTAGGCATCAGAG

GTGCAGCCATTTCACATGTGCTCTCTCAGTACATTATGGCAAGTTTACTCTTGTTTATATTAATGAAAAAAGTGGATCTC

CTACCTCCAAGCATGAAGGATTTGCAGATTTTCCGGTTTCTTAAAAATGGTGGTCTATTGTTGGCCAGAGTTATAGCAGT

GACATTCTGTGTGACCTTATCAGCCTCATTAGCAGCAAGGTTAGGTCCAATTCCAATGGCTGCATTCCAAACCTGCCTCC

AGGTTTGGATGACATCCTCCCTTCTCGCTGATGGTTTAGCTGTTGCAATACAGGCAATTCTAGCATGTTCCTTTGCTGAG

AAAGACTATAATAAAGTAACTACTGCTGCAACAAGGACACTGCAAATGAGTTTTGTTTTAGGAGTTGGACTCTCTCTAGT

AGTTGGAGGTGGATTATACTTTGGAGCCGGAGTATTTTCCAAAAATGTTGCTGTTATTCACCTAATCAGACTAGGCCTCC

CGTTTGTTGCTGCAACACAACCAATCAATTCATTAGCCTTTGTCTTTGATGGTGTGAACTATGGAGCATCTGATTTCGCT

TATTCTGCATACTCCTTGGTCTTGGTCTCATTAGCAAGTGTTACTTCTTTATTCTTTCTCTACAAGAGTAAGGGTTTTAT

TGGTATCTGGATTGCACTAACCATCTATATGAGTCTTCGCATGTTTGCTGGTGTATGGAGGATGGGAACAGGAACAGGAC

CCTGGCGTTTTCTCAGAGGCCACTCATTGTCTTGA

>MsMATE03

ATGAATATCAAAGCAGCTGAAAATGATAAGAGTAAGTTAACTGAAGTAACACCTGAGAGTGATGTTGTTCAAGACGTAGA

GAAAGGGACACCCAAAGAGAGTAATAAGGCTCAAAAAGAATCTGTGGTAGGACACAATGAAACAAATGGTACACTTGGGA

ACAATGATAAGACTAATGGAGTTGCAGTAAAGAATAATGAACAAGAACCCCATTTACTATCCTCAGATCCTAGGAGCAAT

AAGAGTAAGGAGATAGTTGTGAAGAAGAAGAAGAGACACATTGCTTCAGCATCAACAGCACTACTCTTTGGCTCAATTCT

TGGCCTCCTACAAGCAGCAATCCTTATATTTGGAGCTAAACCTCTATTATATGTGATGGGTGTGAAACATGGCTCTCCAA

TGCTAAAACCAGCAGTGAAGTATTTGACATATAGATCATTTGGTGCTCCTGCGGTTCTTCTCTCATTGGCAATGCAAGGA

ATATTTCGAGGATTTAAGGACACAACAACTCCTTTATATGTCATTGTTGCGGGATATTCATTGAATGTGTTATTGGAGCC

ATTACTTATATTTAAATTGAAAATGGGCATAAAAGGAGCAGCTATTGCACATGTTATCTCTCAGTACATGATGGCATTCA

CCCTCTTCTTCATATTAATGAAAAAAGTGTATCTCCTACCTCCAAGAATAAAAGATCTTCAGATTTTCAGGTTTCTTAGA

AATGGAGGTCTGTTGATGACAAAGGTAATAGCAGTAACATTCTGTGTGACTTTAGCAGCAAGTTTGGCTGCAAGGTTAGG

TTCAATTCCCATGGCTGCCTTTCAACCTTGCCTTCAAGTTTGGTTAGCATCCTCGCTTTTTGCTGATGGATTGGCTATAG

CTGTACAGGCAATCCTAGCTGGTTCTTTCGCGGAGAAAGACTATAACAAGACAACAGCAGCAGCGACGAGGACTCTACAA

TTCGGTTTTATTTTAGGGGCGGGGCTCTCTGTTATTGTTGGATTTGGATTATACTTTGGAGCTGGAATCTTTACCAAAAA

CCTTCAAGTTATACACTTTATCCGAATAGGCGCCCCGATTGTAGCTGCAACACAACCAATCAATACATTAGCCTTTGTCT

TTGATGGTGTAAACTATGGAGCTTCTGATTTTGCATATGCTTCATATTCCTTGGTTACAGTGTCATTGTTAAGTGTTGGT

GTAGAATTTCTACTGTACAGGAGCAACCAATTCATTGGGATATGGATTGCACTATCCATCTATATGACTCTTCGCATGTT

GGCCGGTGTATGGAGGATGGGAACAGGTACAGGACCTTGGAACTATCTCAGAGGATAA

>MsMATE04

ATGAATGAAAATGGCAATGCTAATGAACCCAACAAGAAGTGGAAATTTCCTTTCTTAGTTTTCTTCAATGATGCAAGACT

TATTTTCAAGTTGGATGCACTTTCAAAGGAGATATTAGGGATTGCAATACCCTCTGCATTGGCTGTTGCTGCTGATCCTA

TTGCTTCTCTTATTGACACAGCATTCATAGGCCATTTGGGTCCCGTGGAACTTGCTGCTGCTGGAGTTTCCATTGCTTTG

TTCAACCAAGCTTCAAAGATCACCATATTTCCTCTTGTTAGTATTACAACTTCCTTTGTAGCTGAGGAAGATACTATCAA

AAGGATGAATATCAAAGCAGCTGAAAATGATAAGAGTAAGTTAACTGAAGTAACACCTGAGAGTGATGTTGTTCAAGACG

TAGAGAAAGGGACACCCAAAGAGAGTAATAAGGCTCAAAAAGAATCTGTGGTAGGACACAATGAAACAAATGGTACAGTT

GCAAACGATGATAAAACTAATGGAGTTGTGGCAATGAAGAATGAACAAGAACCCCATTTACTATCCTCAGTTCCAAGGAG

CAATAAGAGTAAGAAGAGACACATTGCTTCAGCATCAACAGCACTACTCTTTGGCTCAATTCTTGGCCTCCTACAAGCAG

CAATCCTTATATTTGGAGCTAAACCTCTATTATATGTGATGGGTGTAAAACATGGCTCTCCAATGCTAAAACCAGCAGTG

AAGTATTTGACATATAGATCATTTGGTGCTCCTGCGGTTCTTCTCTCATTGGCAATGCAAGGAATATTTCGAGGATTTAA

GGACACAACAACTCCTTTATATGTCATCGTTGCGGGATATTCATTGAATGTGTTATTGGAGCCATTACTTATTTTTAAAT

TGAAAATGGGCATAAAAGGAGCAGCTATTGCACATGTTATCTCTCAGTACATGATGGCATTCACCCTCTTCTTCATATTA

ATGAAAAAAGTGTATCTCCTACCTCCAAGAATAAAAGATCTTCAGATTTTCAGGTTTCTTAGAAATGGAGGTCTGTTGAT

GACAAAGGTAATAGCAGTAACATTCTGTGTGACTTTAGCAGCAAGTTTGGCTGCAAGGTTAGGTTCAATTCCCATGGCTG

CCTTTCAACCTTGCCTTCAAGTTTGGTTAGCATCCTCCCTTTTTGCTGATGGTTTGGCTATAGCTGTACAGGCAATCCTA

GCTGGTTCTTTCGCGGAGAAAGACTATAACAAGACAACAGCAGCAGCGACGAGGACTCTACAATTCGGTTTTATTTTAGG

GGCGGGGCTCTCTGTTATTGTTGGATTTGGATTATACTTTGGAGCTGGAATCTTTACCAAAAACCTTCAAGTTATACACT

TTATCCGAATAGGCGCCCCGATTGTAGCTGCAACACAACCAATCAATACATTAGCCTTTGTCTTTGATGGTGTAAACTAT

GGAGCTTCTGATTTTGCATATGCTTCATATTCCTTGGTTACAGTGTCATTGTTAAGTGTTGGTGTAGAATTTCTACTGTA

CAGGAGCAACCAATTCATTGGGATATGGATTGCACTATCCATCTATATGACTCTTCGCATGTTGGCCGGTGTATGGAGGA

TGGGAACAGGTACAGGACCTTGGAACTATCTCAGAGGATAA

>MsMATE05

ATGAATGAAAATGGCAATGCTAATGAACCCAACAAGAAGTGGAAATTTCCTTTCTTAGTTTTCTTCAATGATGCAAGACT

TATTTTCAAGTTGGATGCACTTTCAAAGGAGATATTAGGGATTGCAATACCCTCTGCATTGGCTGTTGCTGCTGATCCTA

TTGCTTCTCTTATTGACACAGCATTCATAGGCCATTTGGGTCCCGTGGAACTTGCTGCTGCTGGAGTTTCCATTGCTTTG

TTCAACCAAGCTTCAAAGATCACCATATTTCCTCTTGTTAGTATTACAACTTCCTTTGTAGCTGAGGAAGATACTATCAA

AAGGATGAATATCAAAGCAGCTGAAAATGATAAGAGTAAGTTAACTGAAGTAACACCTGAGAGTGATGTTGTTCAAGACG

TAGAGAAAGGGACACCCAAAGAGAGTAATAAGGCTCAAAAAGAATCTGTGGTAGGACACAATGAAACAAATGGTACACTT

GGGAACAATGATAAGACTAATGGAGTTGATCCTAGAAGCAATAAGAGTAAGGAGATAGTTGTGAAGAAGAAGAAGAGACA

CATTGCTTCAGCATCAACAGCACTACTCTTTGGCTCAATTCTTGGCCTCCTACAAGCAGCAATCCTTATATTTGGAGCTA

AACCTCTATTATATGTGATGGGTGTGAAACATGGCTCTCCAATGCTAAAACCAGCAGTGAAGTATTTGACATATAGATCA

TTTGGTGCTCCTGCGGTTCTTCTCTCATTGGCAATGCAAGGAATATTTCGAGGATTTAAGGACACAACAACTCCTTTATA

TGTCATTGTTGCGGGATATTCATTGAATGTGTTATTGGAGCCATTACTTATTTTTAAATTGAAAATGGGCATAAAAGGAG

CAGCTATTGCACATGTTATCTCTCAGTACATGATGGCATTCACCCTCTTCTTCATATTAATGAAAAAAGTGTATCTCCTA

CCTCCAAGAATAAAAGATCTTCAGATTTTCAGGTTTCTTAGAAATGGAGGTCTGTTGATGACAAAGGTAATAGCAGTAAC

ATTCTGTGTGACTTTAGCAGCAAGTTTGGCTGCAAGGTTAGGTTCAATTCCCATGGCTGCCTTTCAACCTTGCCTTCAAG

TTTGGTTAGCATCCTCGCTTTTTGCTGATGGATTGGCTATAGCTGTACAGGCAATCCTAGCTGGTTCTTTCGCGGAGAAA

GACTATAACAAGACAACAGCAGCAGCGACGAGGACTCTACAATTCGGTTTTATTTTAGGGGCGGGGCTCTCTGTTATTGT

TGGATTTGGATTATACTTTGGAGCTGGAATCTTTACCAAAAACCTTCAAGTTATACACTTTATCCGAATAGGCGCCCCGA

TTGTAGCTGCAACACAACCAATCAATACATTAGCCTTTGTCTTTGATGGTGTAAACTATGGAGCTTCTGATTTTGCATAT

GCTTCATATTCCTTGGTTACAGTGTCATTGTTAAGTGTTGGTGTAGAATTTCTACTGTACAGGAGCAACCAATTCATTGG

GATATGGATTGCACTATCCATCTATATGACTCTTCGCATGTTGGCCGGTGTATGGAGGATGGGAACAGGTACAGGACCTT

GGAACTATCTCAGAGGATAA

>MsMATE06

ATGGCAGAGAAAGAGAGTTTGTTTTCTATAGGTGATTGGATGAGAATACCAATTTGCACTTTCTTCAAGGATGCTAGACT

AGTATTTAAATTAGACGATCTTGGTCGCGAAATATTGTCAATTGCTTTGCCCGCTGCAATGGCTTTGACGGCTGATCCTA

TAGCATCACTGGTCGATACGGCCTTCATTGGCCAATTAGGTCCAGTGGAGCTTGCTGCAGTAGGAGTTTCCATAGCTCTA

TTCAATCAAGCATCAAGGATCTTTATATTCCCACTAGTCAGTGTCACAACTTCTTTTGTGGCTGAGGAAGATGCCTTAAG

CGACGCAAGCTCACAGGTAGAGGAGAATGGATGCTTGGAAGCTGCGACACCTCCGGATGCTGAAACCAAAGAGTTCTTAC

CGCAGAAAAATTCGGTTGTTGAAAGCTTTAATGTAGTTAAGGATGATGGACATAAGAGAAGGAAAATTCCTTCAGCTTCG

TCAGCGCTATATTTTGGAGGTATCCTTGGCCTCGTCCAGGCAACGTTGCTTATTTCTGCAGCAAAACCTTTATTGAACTT

CATGGGAGTGACTTCTGATTCTCCTATGCTACATCCTGCAATGCAGTACTTGAAATTGAGATCTCTTGGTGCTCCTGCGG

TTCTTCTTTCATTAGCGATGCAGGGAGTTTTTCGAGGATTTAAAGACACTAAAACTCCTTTATATGCCACTGGTATGTAT

GCCTTGACAAACTTCAATTGTTATTTGCTATAG

>MsMATE07

ATGGCAGAGAAAGAGAGTTTGTTTTCTATAGGTGATTGGATGAGAATACCTATTTGCACTTTCTTCAAGGATGCTAGACT

AGTATTTAAATTAGACGATCTTGGTCGCGAAATATTGTCAATTGCTTTGCCCGCTGCAATGGCTTTGACGGCTGATCCTA

TAGCATCATTGGTCGATACGGCCTTCATTGGCCAATTAGGTCCAGTGGAGCTTGCTGCAGTAGGAGTTTCCATAGCTCTA

TTCAATCAAGCATCAAGGATCTTTATATTCCCACTAGTCAGTGTCACAACTTCTTTTGTGGCTGAGGAAGATGCCTTAAG

CGACGCAAGCTCACAGGTAGAGGAGAATGGATGCTTGGAAGCTGCGACACCTCCGGATGCTGAAACCAAAGAGTTCTTAC

CGCAGAAAAATTCGGTAGTTGAAAGCTTTAATGTAGTTAAGGTTGATGGAAGTAAGAGAAGGCAAATTCCTTCAGCTTCG

TCAGCGCTATATTTTGGAGGTATCCTTGGCCTCGTCCAGGCAACGTTGCTTATTTCTGCAGCAAAACCTTTATTGAACTT

CATGGGAGTGACTTCTGATTCTCCTATGCTACATCCTGCAATGCAGTACTTGAAATTGAGATCTCTTGGTGCTCCTGCGG

CTCTTCTTTCATTAGCAATGCAGGGAGTTTTTCGAGGATTTAAAGACACTAAAACTCCTTTATATGCCACTGTGGCAGGA

GATTTGACGAATATAGCACTAGATCCGCTTTTCATTTTTGTATTCCGCATGGGTGTCAACGGTGCAGCCATTGCACATGT

TATATCTCAGTATCTACTTTCAGCTATACTCCTGTGGAGTTTGAACAAACAAGTTGATCTTATACCTCCAAGCATCAAAC

ATCTGCAATTCGATCGATTTGCCAAAAATGGTTTTCTATTATTCATGAGAGTCATTGCGGTAACATTCTGCGTGACACTG

GCTGCATCATTAGCTGCACACCACGGAGCAACATCCATGGCTGCATTTCAAGTCTATCTGCAAGTTTCGTTGGCAGTGTC

CCTTCTTGCGGATGGGCTGGCTGTTGCCGGGCAGGCGATTCTTGCAGGTGCATTTGCTAACAAGGACTATGAAAAGGCCT

CAACAACTGCTACCCGAGTATTGCAGATGGGCATGGTTCTTGGATTGGCACTTGCATTCATTCTTGGAACAGGATTGCAC

TTTGGAGCTAAACTATTTACAAAAGATGATGATGTCCTACACCTCATTAGAGTTGGGGTCCCGGTAATTTATTCGTGTTC

AAATTTGGAAAGGATATATTGA

>MsMATE08

ATGAGAATACCAATTTGCACTTTCTTCAAGGATGCTAGACTAGTATTTAAATTAGACGATCTTGGTCGCGAAATATTGTC

AATTGCTTTGCCCGCTGCAATGGCTTTGACGGCTGATCCTATAGCATCATTGGTCGATACGGCCTTCATTGGCCAATTAG

GTCCAGTGGAGCTTGCTGCAGTAGGAGTTTCCATAGCTCTATTCAATCAAGCATCAAGGATCTTTATATTCCCACTAGTC

AGTGTCACAACTTCTTTTGTGGCTGAGGAAGATGCCTTAAGCGACGCAAGCTCACAGGTAGAGGAGAATGGATGCTTGGA

AGCTGCGACACCTCCGGATGCTGAAACCAAAGAGTTCTTACCGCAGAAAAATTCGGTTGTTGAAAGCTTTAATGTAGTTA

AGGATGATGGACGTAAGAGAAGGCAAATTCCTTCAGCTTCGTCAGCGCTATATTTTGGAGGTATCCTTGGCCTCGTCCAG

GCAACGTTGCTTATTTCTGCAGCAAAACCTTTATTGAACTTCATGGGAGTGACTTCTGATTCTCCTATGCTACATCCTGC

AATGCAGTACTTGAAATTGAGATCTCTTGGTGCTCCTGCGGTTCTTCTTTCATTAGCGATGCAGGGAGTTTTTCGAGGAT

TTAAAGACACTAAAACTCCTTTATATGCCACTGTGGCAGGAGATTTGACGAATATAGCACTAGATCCGCTTTTCATTTTT

GTATTCCGCATGGGTGTCAACGGTGCAGCCATTGCACATGTTATATCTCAGTATCTACTTTCAGCTATACTCCTGTGGAG

TTTGAACAAACAAGTTGATCTTATACCTCCAAGCATCAAACATCTGCAATTCGATCGATTTGCCAAAAATGGTTTTCTAT

TATTCATGAGAGTCATTGCGGTAACATTCTGCGTGACACTGGCTGCATCGTTAGCTGCACACCACGGATCAACATCCATG

TCTGCATTTCAAGTCTGTCTGCATTCCACGAATCAATATGCTTATTTTGATTTTTATATGACTGTTCTGTTTGTGCTAAT

GCAGCCAACAATTTGTCCAAATGCTACATGTAACAACAGAGCAAAGTCTCCTTCGCCAAGAGAGCAAATTTACTGA

>MsMATE09

ATGGCAGAGAAAGAGAGTTTGTTTTCTATAGGTGATTGGATGAGAATACCAATTTGCACTTTCTTCAAGGATGCTAGACT

AGTATTTAAATTAGACGATCTTGGTCGCGAAATATTGTCAATTGCTTTGCCCGCTGCAATGGCTTTGACGGCTGATCCTA

TAGCATCATTGGTCGATACGGCCTTCATTGGCCAATTAGGTCCAGTGGAGCTTGCTGCAGTAGGAGTTTCCATAGCTCTA

TTCAATCAAGCATCAAGGATCTTTATATTCCCACTAGTCAGTGTCACAACTTCTTTTGTGGCTGAGGAAGATGCCTTAAG

CGACGCAAGCTCACAGGTAGAGGAGAATGGATGCTTGGAAGCTGCGACACCTCCGGATGCTGAAACCAAAGAGTTCTTAC

CGCAGAAAAATTCGGTAGTTGAAAGCTTTAATGTAGTTAAGGATGATGGAAGTAAGAGAAGGCAAATTCCTTCAGCTTCG

TCAGCGCTATATTTTGGAGGTATCCTTGGCCTCGTCCAGGCAACGTTGCTTATTTCTGCAGCAAAACCTTTATTGAACTT

CATGGGAGTGACTTCTGATTCTCCTATGCTACATCCTGCAATGCAGTACTTGAAATTGAGATCTCTTGGTGCTCCTGCGG

TTCTTCTTTCATTAGCAATGCAGGGAGTTTTTCGAGGATTTAAAGACACTAAAACTCCTTTATATGCCACTGTGGCAGGA

GATTTGACGAATATAGCACTAGATCCGCTTTTCATTTTTGTATTCCGCATGGGTGTCAACGGTGCAGCCATTGCACATGT

TATATCTCAGTATCTACTTTCAGCTATACTCCTGTGGAGTTTGAACAAACAAGTTGATCTTATACCTCCAAGCATCAAAC

ATCTGCAATTCGATCGATTTGCCAAAAATGGTTTTCTATTATTCATGAGAGTCATTGCGGTAACATTCTGCGTGACACTG

GCTGCATCGTTAGCTGCACACCACGGATCAACATCCATGTCTGCATTTCAAGTCTGTCTGCATTCCACGAATCAATATGC

TTATTTTGATTTTTATATGACTGTTCTGTTTGTGCTAATGCAGCCAACAATTTGTCCAAATGCTACATGTAACAACAGAG

CAAAGTCTCCTTCGCCAAGAGAGCAAATTTACTGA

>MsMATE10

ATGAACATGAATGCCGAAACCACTCCTGAACAACAATTACCATCCAATCAAATTCAAAATGAAATATCAGATGTGAAACG

TGAGCTTATATCACTCTCCTTGCCTGCCCTTGCTGGACAAGCAATTGACCCAATTGCACAATTGATGGAAACTGCTTACA

TTGGTCGACTCGGTACTCTTGAATTGGCTTCTGCTGGTGTTTCTGTTGTCATCTTTAACATCATTTCTAAGCTTTTTAAC

ATTCCCCTTCTTAGTGTCGCTACTTCTTTCGTTGCCGAAGACATGGCCAACATTTCTGGCAATGCTAGTATAGAAATTAG

TGGGAATAGCAATCCTTTCAAAGCAGTTTATCAAAGAAACCAACTCTCCTCTGTCTCCACTGCTTTGCTATTAGCCTTGG

GGATTGGCATTTTTGAGGCTTTAGCTTTGTATTTTGGATCCGGAATCTTTCTTCGTTTAATTGGTGTATCACCGGGAAAT

CCAACACTCGTTCCCGCACAAAAATTTCTCTCTTTGAGAGCATTTGGTGCTCCTGCAGTCGTGCTTTCTTTGGCTCTTCA

AGGAATTTTTCGTGGTTTTAAGGATACTAAAACTCCCGTTATATGTCTAGGCATTGGTAATCTTTCAGCGGTCTTCTTAT

TTCCCTTACTTATGTATTACTTTAGGTTGGGTGTAGCCGGTGCAGCCATTTCCACTGTTCTCTCTCAATATATTGGGACC

TTGTTGATGATATGGTGTCTAAATAAGAGAGCTGTGTTACTACCTCCAAAAATGGGAAACCTACAATTTGGCGGTTATAT

TAAGTCTGGTGGTTTCGTTCTTGGAAGAACACTTGCTGTTCTTACAACCATGACATTGGGGACATCAATGGCTGCTCGTC

ATGGTCCCGTAGCTATGGCTGCACATCAAATATGCATGCAAGTGTGGTTAGCTGTTTCCCTTCTTACAGATGCATTGGCG

GTATCTGGTCAGGCCCTAATTGCAAGTTCTCTATCAAGACATGAATACAAAGCTGTGAAGGAAATTACTCATTTTGTATT

AAAGATTGGATTGCTGACAGGCATCTGCTTGACTGCAATTCTGGGGGCATCTTTTGGATCTTTAGCTACCCTTTTTACCC

AGGACATTGAAGTCTTGCAGGTTGTCAGAACTGGAGTGTTGTTTGTCAGTGCTTCTCAACCTTTTAATGCACTGGCTTAT

ATTTTTGATGGTCTCCATTATGGTGTTTCTGATTTCCGATATGCTGCTTTCTCCATGATGTTTGTGGGAGCAGTCTCCTC

AGCATTTTTGGTATTTTCCCCTTCACATTTTGGCCTTCGCGGTGTATGGCTGGGATTGACTCTCTTCATGGCACTCCGTG

TAGTAGCTGGTTCTGTCAGATTACTATCAAAGAATGGTCCTTGGTGGTTTCTACACAAGGATTTTCAGATTGCAGAGATG

GGTTCTTAA

>MsMATE11

ATGGATATGCTGTCTATTGCCTTGCCTGCTGCTGTTGCCTTGGCAGCTGATCCAATTGCCTCATTGATCGACACAGCGTT

CGTCGGTCACATAGGGGCGGTTGAATTAGCTGCAGTTGGGGTTTCAGCTTCTGTGGTTAACCTCGTGTCGAAAGTATTCA

ATGTTCCTTTACTTAATATTACTACATCCTTTGTTGCCGAGGAACAAGCGCTGATTGGAAAGGAGGAAGATTCCGGTCAA

ATTGAGGAAAATGGAAAGGCCCAACGCAAGAAGCTCCTTTCCTCAGTATCAACTTCTTTAGCACTTGCTGCAGGTCTTGG

AATTGCTGAAACTGTCGCACTTTCACTTGGCTCTGGACCTCTTATGACTATCCTGGGTATAGCTGCTGACTCTCCAATAC

GTGAACCTGCTGAACATTTTCTTACATTGAGGGCCTTTGGTGCTCTACCAATTGTGATTGCATTAGCTGCACAAGGCACT

TTTCGTGGATTTAAGGATACAAAGACACCTCTATATGCAGTTGGCAAGTATTGCAGCAGCTTTCTCCCTCGCTCTCTCCA

TTTACTTGCTCATAACTTACACACAGTTGATTCCAGTTACAATTGCTTATGA

>MsMATE12

ATGGATATGCTGTCTATTGCCTTGCCTGCTGCTGTTGCCTTGGCAGCTGATCCAATTGCCTCCTTGATCGACACAGCGTT

CGTCGGCCACATAGGGGCGGTTGAATTAGCTGCAGTTGGGGTTTCAGCTTCTGTGTTTAACCTCGTGTCGAAAGTATTCA

ATGTTCCTTTACTTAATATTACTACATCCTTTGTTGCCGAGGAACAAGCGCTGATTGGAAAGGAGGAAGATTCCGGTCAA

ATTGAGGAAAATGGAAAGGCCCAACGCAAGAAGCTTCTTTCCTCAGTATCAACTTCTTTAGCACTTGCTGCAGCTCTTGG

AATTGCTGAAACTGTCGCACTTTCACTTGGCTCTGGCCCTCTTATGACTATCCTGGGTATAGCTGCTGACTCTCCAATAC

GTGAACCTGCTGAACATTTTCTTACATTGAGGGCCTTTGGTGCTCTACCAATTGTGATTGCATTAGCTGCACAAGGCACT

TTTCGTGGATTTAAGGATACAAAGACACCTCTATATGCAGTTGGTGCTGGGAACTTTCTTGTTGTGATATTGGATCCAAT

ATTGATATTTCTTTGTGGTCTTGGCATTAGTGGTGCTGCAATTGCTACAGTGATCTCTGAATATTTAATAGCTTTCATTC

TTCTATGGAATTTGAGTGGCAAAGTTCTGCTAACCCCTTTTGACTTTGATGGGCCAAAGTTTTTCAGCTATCTGAAATCT

GGTGGTCTGCTAATTGCCAGGACTTTGGCTGTGTTTATAACTATGACACTGACAACATCTTTGGCAGCTAACCAGGGCCC

TATACCTATGGCAGGCCATCAAATTTGCATGGAAGTTTGGTTGTCTATATCTTTGCTTACCGATGCTCTAGCACTGGCTG

GTCAGTCTCTTCTTGCCAGTAGTTACTCACTGGGAAATTATGAGCACGCACGCCTCATTATATATAGAGTGATACAGATT

GGTTTAGGAGTTGGAGTCACTTTGTCGATGATCTTATTTTTTGGGTTTGGACCATTTTCTAGTTTATTTAGCACAGACTC

GGAAGTTTTGGATGTTGCTCAGTCAGGTATATTGTTTGTTGCTGGATCTCAGCCAGTGAATGCTTTGGCGTTTGTTATTG

ATGGGCTTTATTATGGGGTGTCGGACTTTGAGTACGCTGCTTACTCGATGGTGCTAGTTGGACTAATTTCTTCAGTTTTC

ATGCTGGTGGCTGCTCCAGTAGTTGGACTTCCTGGAGTCTGGACAGGATTGTTTCTCTTCATGGCGTTGCGTGTTCTAGC

TGGAGTTTGGAGGTTGAGCAGCAAAAGTGGTCCATGGGATATGATCTGGTATGAAAATAGAGCAGAAGACTGA

>MsMATE13

ATGGATATGCTGTCTATTGCCTTGCCTGCTGCTGTTGCCTTGGCAGCTGATCCAATTGCCTCATTGATCGACACAGCGTT

CGTCGGTCACATAGGGGCGGTTGAATTAGCTGCAGTTGGGGTTTCAGCTTCTGTGTTTAACCTCGTGTCGAAAGTATTCA

ATGTTCCTTTACTTAATATTACTACATCCTTTGTTGCCGAGGAACAAGCGCTGATTGGAAAGGAGGAAGATTCCGGTCAA

ATTGAGGAAAATGGAAAGGCCCAACGCAAGAAGCTCCTTTCCTCAGTATCAACTTCTTTAGCACTTGCTGCAGGTCTTGG

AATTGCTGAAACTGTCGCACTTTCACTTGGCTCTGGACCTCTTATGACTATCCTGGGTATAGCTGCTGACTCTCCAATAC

GTGAACCTGCTGAACATTTTCTTACATTGAGGGCCTTTGGTGCTCTACCAATTGTGATTGCATTAGCTGCACAAGGCACT

TTTCGTGGATTTAAGGATACAAAGACACCTCTATATGCAGTTGGTGCTGGGAACTTTCTTATTGTGATATTGGATCCAAT

ATTGATATTTCTGTGTGGTCTTGGCATTAGTGGTGCTGCAATTGCTACAGTGATCTCTGAATATTTAATAGCTTTCATTC

TTCTATGGAATTTGAGTGGCAAAGTTCTGCTAACCCCTTTTGACTTTGATGGGGCAAAGTTTTTCAGCTATCTGAAATCT

GGTGGTCTGCTAATTGCCAGGACTTTGGCTGTGTTTATAACTATGACACTGACAACATCTTTGGCAGCTAACCAGGGCCC

TATACCTATGGCAGGCCATCAAATTTGCATGGAAGTTTGGTTGTCTATATCTTTGCTTACCGATGCTCTAGCACTGGCTG

GTCAGGTTGGATCATACTATTGCATTGTCTTATAA

>MsMATE14

ATGTATACCATTCAAATTTACCTGAGAAATGTAATCAGTGATACTCCTTTTTCCTTCAAATTGGTAGTTCCATTGATTGT

CCTTCCCGTTTCATTATTGTCTTACAGGTTTTCTATTACACTGGCTGCATCATTAGCTGCACACCACGGATCAACATCCA

TGGCTGCATTTCAAGTCTGTCTGCAAGTTTGGTTGGCAGTGTCCCTTCTTGCGGATGGTCTGGCTATTGCCAGGCAGGCG

ATTCTTGCAGGTGCATTTGCTAACAAGGACTATGAAAAGGTGATTGTACATTATTTTAAGAAGATCGCTGGTTCTTTTTA

A

>MsMATE15

ATGGCACTCAAAATTCCATCAATATCTCTTCTTCCTCGCTCTCTTCACAACTTTCCTCCCCGCCAAAACCCTAATCTCAA

ACCGCTTCCCCTCTCTCCAACCATTTCTCACTCTCACCTCCCTCACCATTTCTCAAGCCTCTCAATCTCCGCACTTCACC

GAACTCAGTTCGTAACCGCCCGCGCGATTCAACCTCAACAACTCACCGGCGATGAAGGCCAAATCACCGAAGCTTCTGAA

GAAGCTAAAATTGAAGATGAAGAAGAAACGACACAGGGTGTTGAAAAGGAACTAGCGAATCAAGGTATATGGATTCAGTT

GAAGGAAATTGTTAAATTTACAGCACCTGCAACTGGACTTTGGATATGTGGACCGTTGATGAGTTTAATCGACACCGCGG

TTATTGGTCAGGGAAGTTCAATTGAGCTTGCTGCTTTAGGTCCTGCTACTGTTGTTTGTGATTACATGAGTTATGTCTTC

ATGTTTCTATCAGTTGCTACTTCCAATATGGTTGCTACTGCCCTTGCTAAACAGGATAGGGAGGAAGTGCAGCATCACAT

ATCTGTCTTGCTTTTTATTGGGTTAGCATGTGGCTCGGCGATGCTTTTTTTCACAAGGTTATTAGGCGCAACAACGCTAG

CAGCTTTTACTGGATCAAAGAATGTACATCTAGTACCCGCAGCTAATACTTATGTCCAGATTCGAGGGTTGGCATGGCCT

TGTTTACTTATTGGATCGATTGCTCAAAGTGCAAGTCTTGGTATGAAAGATTCTTGGGGACCCTTGAAGGCTTTGGCTGC

TGCCAGTATTATAAATGGAATTGGTGATATAGTTCTGTGCAGATATTTAAACTATGGGATTGCTGGGGCTGCATGGGCTA

CATTGGCATCCCAAGTCGTTGCTGCGTATATGATGAGCAAAGCTCTAAATGACAAGGGATACAATGCATTTTCCTTCACC

ATTCCTTCAGGGAAGGAATTTCTGTCAATATTTAGTCTTGCTGCTCCTGTATTTGTGACATTGATGTTAAAGGTGGCTTT

CTACTCTCTAATTATATATTTTGCTACATCAATGGGTACAAATAAAATAGCTGCTCATCAAGTCATGCTTCAGGTCTACA

TGTTATGCGCAATATGTGGTGAACCTCTCTCCCAAACTGCCCAATCATTTATGCCTGAGTTGATGTACGGAGTAAATCGG

AGTTTGGCAAAGGCTAGGTCGCTTCTAAGGTCTCTTCTAACTATTGGAGCTGTATTCGGGTTGCTTTTGGGGATTGTTGT

AACATCTGTTACTTGGTTATTCCCCTACATTTTTACACCTGATCAAATGGTCATCCAGGAGATGCATAGGATTCTGATTC

CATTCTTTTTAGCACTACTGGTCACACCTGCAACAGTCGGCCTGGAGGGAACATTGCTGGCTGGGCGGGATCTAAGATTT

ATAAGTTTGTCAATGACTGGATGCTTTTGTTTGAATGGTCTAGTATTATTGATCCTATCCAGCAGATATGGTTTGCAAGG

CTGTTGGTTTTCACTTGCTGGATTTCAATGGGTTCGGTTTTCATCGGCCCTTCTGCGCCTTCTATCTCCCAATGGCATTT

TATACTCGGAAGATATAAGCCAGTCTGAGCTGCAAAAATTGAAGACTGCATAA

>MsMATE16

ATGGCGCACCAATTCTCACTGCATTTCAATCATCAAACTCTTCACCTCGTTAACCGCAACCTCATTTCCCATTTAAACCG

CCACTTACCTCTTCATTCCCTTCTCAACACCACCACCACCACCGTTATCCATTCAACTAACCAACGCATTATTACATCCT

CGAGCCGGAACCGTCGCTCCGGGTTCCTAACACCTCGCGTACTTCAAAATCAAGAAGTCACAAACGAATCCGAACACCAA

GAACAAATCAGCGAAGTTTCTTCTAAAGAACAAGCACAAGAAGAAGAAATGAAGGAAATACTTGTTGAGCAGAATATATG

GATTCAGATGAAGGAAATTGTATTGTTTACTGGACCTGCTATTGGTCTTTGGTTATGTGGACCGTTGATGAGTCTCATTG

ATACTGCTGTTGTTGGTCAAGGAAGTTCAATTGAACTCGCTGCTTTAGGTCCTGCTACTGTTGTTTGTGATTACATGAGC

TACGCCTTCATGTTTCTATCAATTGCTACTTCCAATATGGTTGCTACTGCCCTTGCTAAACAGGATAGGGAGGAAGTGCA

GCATCACATATCTGTCTTGCTTTTTATTGGGTTAGCATGTGGCTCGGCGATGCTTTTTTTCACAAGGTTATTAGGCGCAA

CAACGCTAGCAGCTTTTACTGGATCAAAGAATGTACATCTAGTACCCGCAGCTAATACTTATGTCCAGATTCGAGGGTTG

GCATGGCCTTGTTTACTTATTGGATCGATTGCTCAAAGTGCAAGTCTTGGTATGAAAGATTCTTGGGGACCCTTGAAGGC

TTTGGCTGCTGCCAGTATTATAAATGGAATTGGTGATATAGTTCTGTGCAGATATTTAAACTATGGGATTGCTGGGGCTG

CATGGGCTACATTGGCATCCCAAGTCGTTGCTGCGTATATGATGAGCAAAGCTCTAAATGACAAGGGATACAATGCATTT

TCCTTCACCATTCCTTCAGGGAAGGAATTTCTGTCAATATTTAGTCTTGCTGCTCCTGTATTTGTGACATTGATGTTAAA

GGTGGCTTTCTACTCTCTAATTATATATTTTGCTACATCAATGGGTACAAATAAAATAGCTGCTCATCAAGTCATGCTTC

AGGTCTACATGTTATGCGCAATATGTGGTGAACCTCTCTCCCAAACTGCCCAATCATTTATGCCTGAGTTGATGTACGGA

GTAAATCGGAGTTTGGTAAAGGCTCGGTCGCTTCTAAGGTCTCTTCTAACTATTGGAGCTGTATTCGGGTTGCTTTTGGG

GATTGTTGTAACATCTGTTACTTGGTTATTCCCCTACATTTTTACACCTGATCAAATGGTCATCCAGGAGATGCATAGGA

TTCTGATTCCATTCTTTTTAGCACTACTGGTCACACCTGCAACAGTCGGCCTGGAGGGAACATTGCTGGCTGGGCGGGAT

CTAAGATTTATAAGTTTGTCAATGACTGGATGCTTTTGTTTGAATGGTCTAGTATTATTGATCCTATCCAGCAGATATGG

TTTGCAAGGCTGTTGGTTTTCACTTGCTGGATTTCAATGGGTTCGGTTTTCATCGGCCCTTCTGCGCCTTCTATCTCCCA

ATGGCATTTTATACTCGGAAGATATAAGCCAGTCTGAGCTGCAAAAATTGAAGACTGCATAA

>MsMATE17

ATGGCACAAAAACTTTCACTACACTTCAATCACACTCTTCACACTTCACTACACTTGAATCGCCACGTACCTCTTCGT

TTCCTCCCACCTTCCCTTCTAAGGAAGAACACTACTATCCATTCACCAAACCAATGCATTATTATATCCTCGAGCCAG

AACAGTCGCTTCGAGTTTCTAACAGCTTGCTCAGTTCAAAATTACGATGCTATAGATGAAGCCGAAGAAAAAGACCAA

ATCAGCGAGGTTTCTTCTAAAGAAGAAGAAGAAGAAGTGAAGGAACTGGTTGAGCAGAGTATATGGATTCAGATGAAG

GAAATTATTCTGTTTACTGGACCTGCTATTGGTCTTTGGTTATGCGGACCGTTGATGAGTCTCATTGATACTGCTGTT

GTTGGTCAAGGAAGTTCAATTGAACTTGCTGCTTTAGGTCCTGCTACTGTTTTTTGTGATTATTTGGGTTATTTTTTT

ATGTTTCTATCGGTTGCTACTTCGAATATGGTTGCTACTGCCCTTGCTAAACAGGATAGGGAGGAAGTGCAGCATCAC

ATATCTGTCTTGCTTTTTATTGGGTTAGCATGTGGCTCGGCGATGCTTTTTTTCACAAGGTTATTAGGCGCAACAACG

CTAGCAGCTTTTACTGGATCAAAGAATGTACATCTAGTACCCGCAGCTAATACTTATGTCCAGATTCGAGGGTTGGCA

TGGCCTTGTTTACTTATTGGATCGATTGCTCAAAGTGCAAGTCTTGGTATGAAAGATTCTTGGGGACCCTTGAAGGCT

TTGGCTGCTGCCAGTATTATAAATGGAATTGGTGATATAGTTCTGTGCAGATATTTAAACTATGGGATTGCTGGGGCT

GCATGGGCTACATTGGCATCCCAAGTCGTTGCTGCGTATATGATGAGCAAAGCTCTAAATGAGAAGGGATACAATGCA

TTTTCCTTCACCATTCCTTCAGGGAAGGAATTTCTGTCAATATTTAGTCTTGCTGCTCCTGTATTTGTGACATTGATG

TTAAAGGTGGCTTTCTACTCTCTAATTATATATTTTGCTACATCAATGGGTACAAATAAAATAGCTGCTCATCAAGTC

ATGCTTCAGGTCTACATGTTATGCGCAATATGTGGTGAACCTCTCTCCCAAACTGCCCAATCATTTATGCCTGAGTTG

ATGTATGGAGTAAATCGGAGTTTGGCAAAGGCTCGGTCGCTTCTAAGGTCTCTTCTAACTATTGGAGCTGTATTCGGG

TTGCTTTTGGGGATTGTTGTAACATCTGTTACTTGGTTATTCCCCTACATTTTTACACCTGATCAGATGGTCATCCAG

GAGATGCATAGGATTCTGATTCCATTCTTTTTAGCACTACTGGTCACACCTGCAACAGTCGGCCTGGAGGGAACATTG

CTGGCTGGGCGGGATCTAAGATTTATAAGTTTGTCAACGAGTGGATGCTTTTGTTCAAGTGCTCTTGTACTATTGATC

TTACGAAGCAGATATGGTCTGCAAGGCTGTTGGTTTTCCCTTGTTGGATTTCAATGGGCTCGGTTTTTAATGGCCCTT

CTGCGCCTTCTATCTCCCAGTGGTATTTTATACTCAGAAGATGTAAGCCGGTATGCGGAGCAAAAGTTGAAGACTGTA

TAG

>MsMATE18

ATGACACTCAAACTTCAACTACACATTCATCACATTTCTTCCTTCAAATTCCTCAACCTCGCCTCACCTTCTCAGTCTCA

CTCACCTCTCCGTTTTCATGCACCTAACGACACTTTTACTATTTCGTCTAAGTTATTCCATGTGGCGTCAAAGAGACGTA

GTATTAGGACTTTAAACGCTCGCGTGGTTGGAAGTAATGAACTAACCGATGAGTCTGAAGAAATGGGAGAGAAAAAAGAA

CTAGCAGATCAGAGTGTGTGGAATCAGATGAAGGAAATTGTTAAGTTTACTGGACCAGCTATGGGATTATGGTTATGTGA

TCCGCTTATGAGCCTCATCGATACTGCTGTCATTGGTCAGGGAAGCTCAACTGAGCTTGCTGCTTTAGGTCCTGCTACAG

TGGTTTGCGATTACATGACCTTAACGTTTATGTTTCTGTCGGTTGTTACATCCAATATCATTGCTACTGCCCTTGCTAAA

CAGGATACAGAAGAAGTGCAGCATCACATATCTGTCTTGCTTTTTGTTGGGTTGGCTTGTGGCTTCATGATGCTTTTGTT

CACATGGCTATTTGGTGCTGCCACACTCACTGCTTTCACTGGGATAAAGAATGCACATGTAGTACCTGCAGCTAACACTT

ACGTACAGATTCGAGGATTGGCGTGGCCTGCTTTACTTGTTGGATGGGTTGCTCAGAGTGCAAGCCTTGGTATGAAAGAT

TCATGGGGACCCTTAAAGGCTTTGGCTGCCGCCAGTGTTATAAATGGAATTGGTGATATACTTTTATGCAGCTGTTTAGG

CTATGGTATTGCAGGGGCGGCATGGGCTACAATGGTGTCACAAGTTGTAACCGCGTATATGATGATTCAGACCCTAAACA

AGAGGGGATACAATGCATTTGCCTTCTCCATTCCTTCAATGAAGGAATTTCTAACAATACTTAGTCTTGCTGCTCCTGTA

TATTTGACATCGATATCAAAGGTGGCTTTCTTTTCTCTACTTATATATGTTGCTACATCAATGGGTACACAAACAATGGC

CGCTCATCAGGTCATGATTCAAATCTACATGGCATGTACAGTTTGGGGAGAACCTCTTTGCCAAACAGCGCAATCATTTA

TGCCGGAGTTAACGTATGGAGTAAATCGGAGTTTTCCAAAGGCTCCGATTGCTACTAAGGTCTCTTATAATTATTGGAGC

CATACTTGGATTGTTATTAGGGATAGTTGGAACATCTCTTATTTGGTTATTCCCATACATATTTACATCTGA

>MsMATE19

ATGACACTCAAACTTCAACTACACATTCATCACATTTCTTCCTTCAAATTCCTCAACCTCGCCTCACCTTCTCAGTCTCA

CTCACCTCTCCGTTTTCATGCACCTAACGACACTTTTACTATTTCGTCTAAGTTATTCCATGTGGCGTCAAAGAGACGTA

GTATTAGGACTTTAAACGCTCGCGTGGTTGGAAGTAATGAACTAACCGATGAGTCTGAAGAAATGGGAGAGAAAAAAGAA

CTAGCAGATCAGAGTGTGTGGAATCAGATGAAGGAAATTGTTAAGTTTACTGGACCAGCTATGGGATTATGGTTATGTGA

TCCGCTTATGAGCCTCATCGATACTGCTGTCATTGGTCAGGGAAGCTCAACTGAGCTTGCTGCTTTAGGTCCTGCTACAG

TGGTTTGCGATTACATGACCTTAACGTTTATGTTTCTGTCGGTTGTTACATCCAATATCATTGCTACTGCCCTTGCTAAA

CAGGATACAGAAGAAGTGCAGCATCACATATCTGTCTTGCTTTTTGTTGGGTTGGCTTGTGGCTTCATGATGCTTTTGTT

CACATGGCTATTTGGTGCTGCCACACTCACTGCTTTCACTGGGATAAAGAATGCACATGTAGTACCTGCAGCTAACACTT

ACGTACAGATTCGAGGATTGGCGTGGCCTGCTTTACTTGTTGGATGGGTCGCACAGAGTGCAAGTCTTGGTATGAAAGAT

TCCTGGGGACCCTTGAAAGCTTTAGCTGCTGCCAGTGTTATAAATGGTGTTGGTGATATAGTTTTGTGCACCTATTTAGG

CTATGGAATTGCAGGGGCTGCATGGGCCACAATGGCATCACAAGTTGTTGCTGCTTATATGATGATGCGAACTCTAAACA

TGAAGGGATACAATGCCTTTGCCTTATCTATCCCTTCCGGGAGGGAATTTCTAACGATACTTGGGCTTGCTGCTCCTGTG

TTTATGACAATGATGTCAAAGGTGGCTTTCTACTCCTTGCTAATATATTTTGCAACATCAATGGGTACACATACAATGGC

TGCTCATCAAGTCATGGTCCAAACCTTTTGCATGTGCACGGTATGGGGTGAACCTCTCTCACAAACTGCTCAATCATTTA

TGCCCGAATTGTTATATGGAGTTAATCGGAATTTGTCAAAGGCCCGGATGCTTCTAAGATCTCTTGCGGTAATCGGAGCT

ACACTTGGATTGTTATTAGGAATTGTTGGAACATCAGTTCCTTTCTTATTTCCCTACATTTTTACATCTGATCAGATGGT

CATACGGGAGATGCACAAGGTGCTGGTTCCATACTTTGTAGCACTGGCTGTGACACCCCCTACTCACAGCCTTGAGGGAA

CGTTGATGGCTGGAAGGGATCTAAGATTCATAAGTCTGTCAATGATTGGATGCTTATGTGGGGGTGCACTGGTGTTATCG

ATCTTGTGTAGCAGATACGGTTTGCAAGGCTGTTGGTTTTCCCTCGCATTATTTCAATGGGCTCGGTTTTCAGTGGCCCT

CTTGCGACTTCTTTCTCCCAAGGGCATTTTATACTCGGAAGATATAGACCATAATAGACTACAAAAGCTTAAAACTGCTT

AG

>MsMATE20

ATGGGAAACGCAAACCTTGGAGTCCTGAGAGTTAGAGGAGGAGATGAAAATAATAGTCCGGTAGCCATTGAAACCGCCAT

CTTCTTTCTTTCTTTCTTTCTTTCTTTCTTTTCTTTCACTTCAAAAATGGCGCACCAATTCTCACTGCATTTCAATCATC

AAACTCTTCACCTCGTTAACCGCAACCTCATTTCCCATTTAAACCGCCACTTACCTCTTCATTCCCTTCTCAACACCACC

ACCACCACCGTTATCCATTCAACTAACCAACGCATTATTACATCCTCGAGCCGGAACCGTCGCTCCGGGTTCCTAACACC

TCGCGTACTTCAAAATCAAGAAGTCACAAACGAATCCGAACACCAAGAACAAATCAGCGAAGTTTCTTCTAAAGAACAAG

CACAAGAAGAAGAAATGAAGGAAATACTTGTTGAGCAGAATATATGGATTCAGATGAAGGAAATTGTATTGTTTACTGGA

CCTGCTATTGGTCTTTGGTTATGTGGACCGTTGATGAGTCTCATTGATACTGCTGTTGTTGGTCAAGGAAGTTCAATTGA

ACTCGCTGCTTTAGGTCCTGCTACTGTTGTTTGTGATTACATGAGCTACGCCTTCATGTTTCTATCAATTGCTACTTCCA

ATATGGTTGCTACTGCCCTTGCTAAACAGGATAGGGAGGAAGTGCAGCATCACATATCTGTCTTGCTTTTTGTTGGGTTG

GCTTGTGGCTTCATGATGCTTTTGTTCACATGGCTATTTGGTGCTGCCACACTCACTGCTTTCACTGGGATAAAGAATGC

ACATGTAGTACCTGCAGCTAACACTTACGTACAGATTCGAGGATTGGCGTGGCCTGCTTTACTTGTTGGATGGGTCGCAC

AGAGTGCAAGTCTTGGTATGAAAGATTCCTGGGGACCCTTGAAAGCTTTAGCTGCTGCCAGTGTTATAAATGGTGTTGGT

GATATAGTTTTGTGCACCTATTTAGGCTATGGAATTGCAGGGGCTGCATGGGCCACAATGGCATCACAAGTTGTTGCTGC

TTATATGATGATGCGAACTCTAAACATGAAGGGATACAATGCCTTTGCCTTATCTATCCCTTCCGGGAGGGAATTTCTAA

CGATACTTGGGCTTGCTGCTCCTGTGTTTATGACAATGATGTCAAAGGTGGCTTTCTACTCCTTGCTAATATATTTTGCA

ACATCAATGGGTACACATACAATGGCTGCTCATCAAGTCATGGTCCAAACCTTTTGCATGTGCACAGTATGGGGTGAACC

TCTCTCGCAAACTGCTCAATCATTTATGCCCGAATTGTTATATGGAGTTAATCGGAATTTGTCAAAGGCCCGGATGCTTC

TAAGATCTCTTGCGGTAATCGGAGCTACACTTGGATTGTTATTAGGAATTGTTGGAACATCAGTTCCTTTCTTATTTCCC

TACATTTTTACATCTGATCAGATGGTCATACGGGAGATGCACAAGGTGCTGGTTCCATACTTTGTAGCACTGGCTGTGAC

ACCCCCTACTCACAGCCTTGAGGGAACGTTGATGGCTGGAAGGGATCTAAGATTCATAAGTCTGTCAATGATTGGATGCT

TATGTGGGGGTGCACTGGTGTTATCGATCTTGTGTAGCAGATACGGTTTGCAAGGCTGTTGGTTTTCCCTCGCATTATTT

CAATGGGCTCGGTTTTCAGTGGCCCTCTTGCGACTTCTTTCTCCCAAGGGCATTTTATACTCGGAAGATATAGACCATAA

TAGACTACAAAAGCTTAAAACTGCTTAG

>MsMATE21

ATGGCACTCAAAATTCCATCAATATCTCTTCTTCCTCGCTCTCTTCACAACTTTCCTCCCCGCCAAAACCCTAATCTCAA

ACCGCTTCCCCTCTCTCCAACCATTTCTCACTCTCACCTCCCTCACCATTTCTCAAGCCTCTCAATCTCCGCACTTCACC

GAACTCAGTTCGTAACCGCCCGCGCGATTCAACCTCAACAACTCACCGGCGATGAAGGCCGAATCAGCGAAGCTTCTGAA

GAAGCTAAAATTGAAGATGAAGCGGCGACACAGGGTGTTGAAAAGGAACTAGCGAATCAAGGTATATGGATTCAGTTGAA

GGAAATTGTTAAATTTACAGCACCTGCAACTGGACTTTGGATATGTGGACCGTTGATGAGTTTAATCGACACCGCGGTTA

TTGGTCAGGGAAGTTCAATTGAGCTTGCTGCTTTAGGTCCTGCTACAGTTGTTTGTGATTACATGAGTTATGTCTTCATG

TTTCTATCAGTTGCTACTTCCAATATGGTTGCTACTGCCCTTGCTAAACAGGATACAGAAGAAGTGCAGCATCACATATC

TGTCTTGCTTTTTGTTGGGTTGGCTTGTGGCTTCATGATGCTTTTGTTCACATGGCTATTTGGTGCTGCCACACTCACTG

CTTTCACTGGGATAAAGAATGCACATGTAGTACCTGCAGCTAACACTTACGTACAGATTCGAGGATTGGCGTGGCCTGCT

TTACTTGTTGGATGGGTCGCACAGAGTGCAAGTCTTGGTATGAAAGATTCCTGGGGACCCTTGAAAGCTTTAGCTGCTGC

CAGTGTTATAAATGGTGTTGGTGATATAGTTTTGTGCACCTATTTAGGCTATGGAATTGCAGGGGCTGCATGGGCCACAA

TGGCATCACAAGTTGTTGCTGCTTATATGATGATGCGAACTCTAAACATGAAGGGATACAATGCCTTTGCCTTATCTATC

CCTTCCGGGAGGGAATTTCTAACGATACTTGGGCTTGCTGCTCCTGTGTTTATGACAATGATGTCAAAGGTGGCTTTCTA

CTCCTTGCTAATATATTTTGCAACATCAATGGGTACACATACAATGGCTGCTCATCAAGTCATGGTCCAAACCTTTTGCA

TGTGCACAGTATGGGGTGAACCTCTCTCGCAAACTGCTCAATCATTTATGCCCGAATTGTTATATGGAGTTAATCGGAAT

TTGTCAAAGGCCCGGATGCTTCTAAGATCTCTTGCGGTAATCGGAGCTACACTTGGATTGTTATTAGGAATTGTTGGAAC

ATCAGTTCCTTTCTTATTTCCCTACATTTTTACATCTGATCAGATGGTCATACGGGAGATGCACAAGGTGCTGGTTCCAT

ACTTTGTAGCACTGGCTGTGACACCCCCTACTCACAGCCTTGAGGGAACGTTGATGGCTGGAAGGGATCTAAGATTCATA

AGTCTGTCAATGATTGGATGCTTATGTGGGGGTGCACTGGTGTTATCGATCTTGTGTAGCAGATACGGTTTGCAAGGCTG

TTGGTTTTCCCTCGCATTATTTCAATGGGCTCGGTTTTCAATGGCCCTCTTACGACTTCTTTCTCCCAAGGGCATTTTAT

ACTCGGAAGATATAGACCATAATAGACTACAAAAGCTTAAAACTGCTTAG

>MsMATE22

ATGGTTATAAAGCAGATGGGCATGGTTCTTGGATTGGCACTTGCATTCATTCTTGGAACAGGATTGCACTTTGGAGCTAA

ACTATTTACAAAAGATGATGATGTCCTACACCTCATTAGAGTTGGGATCCCGTTTGTAGCACTCACTCAACCCCTGAACT

GTTTGGCCTTTGTATTTGATGGTGTCAACTTTGGGGCATCTGATTTTGCATATTCAGCCTTCTCCATGGTTATAGTGGCA

ATTATTAGCATAATTTGTCTACTTATTTTGTCATCTGCTGGTGGATTCATTGGAATTTGGGTTGCTTTGACTATTTATAT

GAGTCTAAGAGCATTTGCTGGCTTCTTGAGGATTGGAACTGGATCAGGACCATGGGAATTCCTTAGGAGCTAA

>MsMATE23

ATGCTATTTCTTTGTTTGTGTTTGGTTTTTCTTTTTGTTGTGTTGTTCGGCGCAAGAGTTTCAAATGCATTAGGAGCTGG

ATGTCCACATGCAGCACGATTGTCTGTCTATGCTGCTATGGCTATCGCAGTTTCTGAGGCTATATTGGTGAGTTCCATCA

TTTTCGCCTCTCGACGGGTGTTAGGTTATATATTTAGCAATGAGCAGGATGTGGTGGATTATGTCACAGATATGGCTCCT

CTGATAAGTCTATCTGTTATAGCAGATAGTTTGCATGGTACCCTTTCAGGTATTGCTAGAGGATGTGGTTGGCAGAAGTC

AGGAGCATATGTGAACCTTGGATCCTATTATGTTTTTGGAATTCCAATTGCTGTTATATTGGGTTTCTGGTTTGAATTGA

GAGGAAAAGGCCTTTGGATTGGGATAATTGTTGGTGCCTCCTGTCAAGCAGTTTTGCTATCCCTTATAACAAGTTTTACA

AACTGGGAAAAACAGGCAATTAAGGCACGGGAAAGGATATTTCGAGAACGTTTTGTGATAGAAGATAGACTAGTTTGA

>MsMATE24

ATGTCATCAACATGCAAATTAGGAGAAAACCTTAGCTCAAACCATAAAACTAACCAACCAACGTCACCTCCTCCACCATT

ACAAACCCGAAAATGCGACACAAATCCAGCGAAGACGCTGCTTTCAGAGGAGCTAAGAGTTCAAGGAAGATTAGCTTTTC

CAATGGTGTTGATGAATTTAGCTTGGTTTGCCAAGACAGCAATCACAACAGCATTTTTGGGTCGACTAGGTGAGCTCAGC

TTAGCCGGTGGCGCGCTCGGGTTCACTTTCGCTAACGTTACTGGCTTCTCTGTCTTGAATGGTCTCTGTGGTGCCATGGA

ACCTATTTGTGGACAGGCTCATGGAGCTAAAAACGTGAGACTCCTTCACAAGACACTTCTCATGACAATTGTATTGTTGC

TATTGGTAACAATTCCTATTACTTTCATGTGGCTTCATATTGACAAAATTTTGATTCATTTTGGCCAACAACAAGAAATT

TCCACTGTTGCTGGTACTTATGTTTACTATCTCATACCTGATTTGTTTGTTATGTCACTCTTGTGTCCCTTAAAAGCTTA

CTTGAGCTCTCAAAGCATCACTCTTCCTACCATGTTTAGTTCTGGGGTAGCACTTGCTTTTCATGTTCCTGTTAACATAC

TACTCTCAAAAACCATGGGTCTAAGAGGGGTTTCCATGGCTGTTTGGATAACTGATCTTATTGTTGTTGTTCTTCTGGCC

ATTTATGTTTTAATTCTAGAGAATCGAAAGGTATTGGCGTGGAAAGAAGGAGGATGGTGGGATCAGAGTATTATGGATTG

GATTAGGCTAATCAAGCTATCTGGATCTTGTTGTCTCAACACATGTATGGAGTGGAGCTGCTATGAAATTCTAGTCTTGC

TTACTGGCCACCTCGCAAACGCCAAGCAAGCATTGGGAGTTTTAGCCATTGTGTTGAACTTTGACTATTTACTTTTCTCA

GTGATGCTGTCATTGGCCACTTGTGTTTCCACCCGTGTCTCAAACGAGCTTGGCGCAAACCAAGCCGACCGAGCGTACCG

GTCAGCGCGTGTGTCTCTAGGAATAGGTTTTATCGCAGGTTGCACCGGCAGCTTGGTGATGGTGGCTGCAAGGGGAATTT

GGGGGCAACTCTTCAGCCATGATAGGGGCACTATAAATGGAGTAAAAAAGACAATGTTGTTGATGGCTCTAGTGGAGTTG

TTTAATTTTCCATTGGCAGTTTGTGGAGGCATAGTTCGAGGGACAGCTCGACCTTGGTTGGGTATGTATGCAAATCTAGG

CGGGTTTTATTTTCTGGCTCTGCCCCTTGGTGTTGTTTTTGCCTTCAAGCTTCGTCTTGGCCTTGTTGGACTCTTCTTTG

GACTTCTTACTGGTATTGTTGTTTGCTTGTCATTATTATTAGTATTCATTGCTAGGATAAAGTGGGTGGAAGAAGCTGCC

AAGGCACAAATATTAGCAAGTAACGACCAAGTTAAAGAAGTTCCCTGTGATGACGCAGAAGTACCAACTGAGGCTCGTGA

AAATGACAAAGTGTAA

>MsMATE25

ATGAGAGAAGATGATAAAGACCATGATTTCTTTTCACACAAATTCCCAACAACCTCTCAGGTGGTGGAAGAGTTGAAG

GAACTATGGAGCATGGCTTTACCAATAACAGCCATGAACATGCTAGTTTTTGTCAGAGCTGTTGTTTCAGTTCTCTTC

CTTGGTCGTCTTGGTAGCTTAGAGCTAGCAGGTGGTGCACTGTCCATAGGTTTCACAAACATAACAGGGTACTCTGTT

CTCGTCGGCCTCGCCTCCGGTCTCGAACCAGTTTGTAGCCAAGCTTTTGGTAGCAAAAACTGGGAACTTCTTTCCCTT

TCACTACAACGCATGGTTCTCATACTTCTCATGGCAATTGTTCCCATAAGTCTCCTTTGGCTTAACCTTGAAAAAATC

ATGCTTTTCATGGGACAAGATGGTAAAATCACTGAAATGGCAGCAATATATTGTTTCTATTCACTACCTGATCTTTTG

ACAAATACTTTGTTACAACCTTTGAGAGTGTTCTTAAGGTCACAAAAAGTGACTAAACCTATGATGTATTGCTCTTTG

ATAGCAGTTGTTTTTCATGTGCCTTTGAATTATTTCTTGGTCATGGTGATGCAATTTGGTGTACCGGGCGTGGCGATG

GCGTCCGTGTTAACGAATATGAACATGGTTGTGTTGATGGCGGGGTATGTCGGCTTGTTTAGGAAGAAGGAGATGATG

TTAAGGTGGCCGGGGTGCGGTGAAGGAGGGATGATGGTGGTGAGTGAGGGTTTGGGGGAGTTGATGAAATTGGCTGTA

CCTAGTTGTCTTATGATATGTTTGGAATGGTGGTGGTATGAGATTGTTACTGTTTTGGCTGGTTATTTGGAGAATCCT

ACATTGGCTGTTGCTGCTACTGGGATTTTGATTCAGACAACTAGTATGATGTATACTGTCCCTATGGCTCTTGCTGGT

TGTGTTTCTGCTAGGGTAGGGAATGAGCTTGGAGCAGGTAAACCATACAAAGCAAAGCTAGCAGCCATGGTAGCCCTA

GGATGTGCATTTGTGATGGGCTTTATCAATGTAACATGGACAGTTATCCTTAGATACGGATGGGCCGGGCTATTCACA

AACGACGAGCCCGTCAAAGCCTTGGTTGCATCAGTCATGCCAATTATGGGCCTATGTGAGCTTGGAAACTGCCCACAA

ACAACCGGCTGCGGCATCCTTCGTGGAACAGCACGGCCCGTCATAGGTGCCAACATAAACCTAGGTTCATTCTACTTT

GTCGGTACTCCGGTGGCCGTGGGCTTGGCATTTTGGTTCAAGATTGGGTTTAGTGGGCTTTGGTTTGGGCTTTTATCT

GCACAAGTGGCATGTGCTTTGTCAATTTTATATGTTGTTATCATAAAAACTGATTGGGAAGCTGAGGCATTGAAGGCA

GAGAAGCTAACAAAGGTAGAGATGGTGATTTGCAATGAAAGTAAGAAGAATAAAGACAAGAAGAAAAATGAAGAATGC

AAAGGTTTATTAGAGAATGAAAATGGGAACAAAATTGACATGTGCTAA

>MsMATE26

ATGGTTGCTCAAGAGAAATCCCAAAAGACATACCCGACAACTGCTGAGGTGGTGGACGAGCTAAAGAAAATGATGGACAT

AGGTTTCCCAATAGCAGCCATGAGCATAGTAGGATACCTCAAAAACATGATCTTAGTTGTTTGCATGGGAAAGTTAGGAA

GCCTTGAGTTAGCAAGTGGTGCTTTGGCCATAGGCTTCACCAATGTAACCGGTTACTCAGTCCTTTCAGGTCTAGCCATG

GGAATGGAACCTCTATGTACTCAAGCCATTGGTTCACAAAACTTCTCTTTAGTCTCTCTCATATTAAGAAGAACAATTCT

CATGTTATTAGTAGCTTCATTACCCATTTCACTCTTATGGCTCAATCTTGAACCATTCATGCTATCCCTTCATCAAAATC

AAGACATAACAAGAATAGCAAGCCTCTATTGCCGCCTATCGATTCCCGATCTTATAGCGAATAGCTTGCTTCATCCAATT

CGCATCTATTTACGTAGCAAAGGAACAACTTGGCCATTATTGTGGTGTACTTCACTTTCCGTTATCATTCATATCCCTAT

CATCATTTTCTTAACCTTCAAACTTCACCTTGGTGTACAAGGTATTGCTATTTCAGCTTTTGTTGCCAATTTCAATACCC

TTTTCTTCCTTTTATCATACATGTTCTACATGCATGTCTCACATGTTTCAATCTCCATACCTATCCCTAGTCCTCCCTTG

TTATCATCACAACAAGAAAAACCAGCAAGTGTCAAAACCCTAGGCAAAGAATGGGGCATGTTAATAAGGTTTTCCATTCA

AAGTTGTCTTGGAGTTTGCTTAGAATGGTGGTGGTATGAGTTCATGACAATTCTAGCTGGTTACCTTTACAACCCTCGTG

TAGCTTTAGCCACGGCGGGAATAGTAATACAAACAACATCACTTATGTACACTTTACCGACGGCACTTAGTGCTTCGGTT

TCAACAAGGGTAGGGAATGCACTTGGAGCTGGTCAACCATCAAGGGCAAATTTGTCAACCATGGTAGCAATTGGAATGTC

ACTAGCAAGTTCAACATTAGGGTTATTATGGACAACATTAGGAAGAGAAAAATGGGGGAAGGTGTTCACAAATGATAAAG

AGGTGTTAGAGTTGACAATGGCGGTTTTACCTATAATTGGTGTTTGTGAGTTAGCAAATTGTCCACAAACAACTAGTTGT

GGAATGCTTAGAGGGAGTGCAAGGCCAGGTATTGGGGCAGGGATAAATTTTTACTCATTTTACTTGGTGGGGGCGCCAAT

AGGAATAGTGTTGGGGTTTGTTTTGAAATTAGGGTTGGTGGGGTTTTGTTATGGTTTGTTGGCAGCACAAATAGCATGTG

TGGTATCAATTCTTGTTGTGGTATATAACACTGATTGGGAAAGAGAGTCATTGAAGGCAAAAAGCTTGGTGGGTAATGAT

ACATGTGACACATTATTTGCTCATGTTGAAGACCAAACAATCAAATGTGAGCAAGGTATTGTCTTTCTCAATGAGAACAA

GTGA

>MsMATE27

ATGTCTGAAACAAAGATCAATACCTTAAACGAGCCAATGATTTCCAAAGACACCTTAAACCAAAGAGACATCATAGTA

ACTGAAACAAAATCTCTATTATCATTAGCTTTACCAACAGCACTAACAGCACTAATCTTCTATGCACGTTCCATGATA

TCTATGATGTTCCTTGGTAAACTTGGTGATGTGGAACTTGCATCAGGTTCATTAGCCATAGCTTTTGCAAACATAACC

GGTTATTCAGTTCTTTCTGGTTTATCTTTAGGTATGGAACCTCTTTGTTCTCAAGCTTTTGGTGCTAACCGTCCAAAA

CTTCTGTCATTAACACTTCAACGGTGTATAATCTTCCTATTATCATGTTCCTTACCCATTTCTTTTCTATGGTTTAAT

ATGTCTAGAATCTTTCATTTTTTACATCAAGATGATAAAATCACACAAATGTCACAAACTTATCTTGTGTTTTTGTTA

CCTGATCTTGTTACTAATTCTTTTCTTCAACCAATTAGGATTTACCTTCGTGCTCAATCTGTCACCTACCCGGTTACT

TTAGCATCTTTGGTTGGAACTTTTTTACATTTACCTTTTAACTTTTTACTCTTCAAAAAGGGTATTTCTGGTATTGCC

ATTGCTTCTGCTGCCTCCAATTTTTCAGTTTTGGTTGTATTGGTGGTTTATGTTTGGATAAGTGGGGTCCACATTGCC

ACATGGAATGCTCCGAGTCGGGAGTGTTTTTTTGGGTGGGGGCCGTTGATTAAGCTAGCTGCGCCGAGCTGTGTTTCG

GTTTGTTTGGAGTGGTGGTGGTATGAGATTGTGATAGTTTTGTGTGGATTTCTTGTGGACCCCACTGCCACTGTGGCA

TCTATGGGGATATTAATTCAGACGACGTCTTTGATTTATGTTTTTCCGTCTTCTATGGGACTCGCCGTTTCGACGCGT

ATTGGGAATGCGCTGGGAGCAAATCGTCCGAGAAATGCGAGGTTTTCCGCTGTGATCGCAGTGTTTTTTGCAGCAGTT

ATGGGATTTACTGCGGTTATTTTTACTATGATGATGAGGTGGCAGTGGGGGAAGATGTTCACCGCCGATGAAGATATT

ATACGCTTGACGGCGGCGGCATTGCCGATTTTAGGATTGTGTGAGCTAGGAAATTGTCCGCAGACAGTTGGTTGCGGT

GTTGTCCGAGGGACAGCGAGGCCGAAAGTGGCGGCGAATGTGAATTTGAGTGCTTTTTATATGGTGGGAATGCCGGTG

GCGGTTGGGCTTGCTTTTTGGTTTGATTTTGGATTTTGCGGGCTTTGGTTAGGCCTTTTATCAGCCCAGGTTTGTTGT

GCGGGCTTAATGTTGTATATAGTTGGAACAACTGATTGGGAACAACAGGCTCGTCGGGCACAGTTATTAACAACATTT

GATGAAGTGGATAATGGATTGGAAGGACAAAAAGAATCATTGATTAGTGGTTTGGAAAGTGCTTGA

>MsMATE28

ATGTGTAATCCAAAGCCATCTTCACCAACTTCACCATTTCTTTCTCCAACAAAAACTCACCTCATAAATCCTCACACCAA

AGCTTCTTATTCAAATCCTCCTACCCTTGATGATGATCATGTTCAAGATGAAATTCATAGATGGCCTACTCTTAAAGAGG

CCATAACAGAAATCAAAGAAATAGGAAAAATATCAGGTCCAACAACAATAACTGGTTTATTGTTATATTCAAGAGCTATG

ATATCAATGATTTTTCTTGGATATCTTGGAGAAATGGAACTAGCAGGAGGTTCACTTTCAATAGGCTTTGCAAATATCAC

TGGTTACTCAGTGATATCAGGATTAGCCATGGGAATGGAACCAATTTGTGGACAAGCTTATGGAGCAAAACAATGGAAGA

TACTTGGCTTAACACTTCAAAGAACAGTGCTTCTTCTTCTTTCAACTTCAATCCCTATCTCATTCATTTGGATTAACATG

AAAAGAATCCTATTGTTTTCAGGTCAAGATGAAGAAATTTCATCAATGGCACAAAGTTTTATACTTTTTTTAGTACCTGA

TCTTTTTCTACTCTCAATTCTTCACCCTTTGAGAATCTATTTAAGGACTCAAGGAATCACATTGCCCTTAACATATTGTT

CAGCTGTTTCTGTTTTGCTTCATATCCCTCTCAATTTTCTGTTAGTGGTTCATTTCCAAATGGGGATTGCGGGGGTGTCA

ATAGCAATGGTTTTGACAAATTTAAACCTTGTTATTTTACTTTCATCTTTTTTATACTTCTCTAGTGTTTATAAAAAGTC

ATGGATTTCACCAAGTTTAGATTGCATTAAAGGGTGGTCCTCATTGCTTTCGCTAGCGATTCCAACTTGTGTTTCGGTTT

GTCTCGAATGGTGGTGGTATGAGTTCATGATAATGATGTGTGGACTTTTGGTTAATCCAAAAGCAACAATTGCTTCAATG

GGAATACTTATTCAAACAACATCTTTGGTTTATGTTTTTCCATCATCACTTAGTCTTGGTGTTTCAACAAGAATAGGAAA

TGAGTTAGGTGCAAATAGGCCACAAAAAGCAAGAATTTCAATGATAGTTTCACTTTTTGTTGCTATGGTTTTAGGACTTG

GAGCAATGCTCTTCACAACATTGATGAGAAACCAATGGGGAAAGTTTTTCACAAATGACAAAGAAATTCTTGAGTTAACA

TCTATTGTGTTACCAATTGTTGGACTTTGCGAGCTCGGAAATTGTCCACAAACAACAGGTTGTGGTGTCCTTAGGGGAAG

TGCACGACCAACAATTGGAGCTAATATAAATTTAGGATCATTTTACCTTGTTGGTATGCCAGTTGCAATCCTTTTGGGAT

TTGTGGCTAAATTGGGGTTTCCAGGGTTGTGGATTGGGTTACTTGCAGCTCAAGGCTCATGTGCAATGCTTATGTTGGTT

GTTCTTTGTAGAACTGATTGGAATTTGCAAGTTCAAAGAGCTAAAGAACTCACAAAAAGTTCAACAATAAGTGATGATGT

TGATGCTAAATTACCAACATTTATGGAAGGTAATGTGAACAAGAATAATGTTCATGGTTGTCTTGAAGAAATTGTTATCA

CTCATGATGTGTTTACTAAGAAATCTTCACTTGAAACAGATCCACTTATCATAACATCTACTACTACCAATTGCATTGAA

GATTAG

>MsMATE29

ATGTGTAAACCTAAGCCATCCTCAACATCCCCATTTCTCTGTCCCACACAAACCAACCTCATAACTTCTGATCCCAAA

CTACTCATAAATGACCCTCCTCATGACGAAGTTCAAGATCAAAATGAACTTCAAAGATGGCCAACTCCAAATGAGGTG

ATAGAAGAATTGAAGGCCATAGGTAAAATATCAGGTCCAACAGCTATAACTGGTTTACTCTTATATTCAAGAGCTATG

ATCTCTATGCTTTTCCTTGGCTATCTCGGCGAGACAGAACTAGCAGGAGGTTCTCTCTCCATAGGTTTTGCCAACATA

ACTGGTTACTCTGTCCTATCTGGTTTAGCCATGGGAATGGAACCCATTTGTGGACAGGCTTACGGAGCTAAACAATGG

AAGATACTTGGTTTAACACTTCAAAGAACCGTTCTTTTACTTCTTTCCACTTCCATTCCCATTGCATATTTATGGCTT

AACATGAAAAAGATTCTCATGTCTTGTGGTCAAGATGAAGAAATTTCATCAACAGCACAAACTTTCATTCTTTTTTCA

CTACCAGATCTTTTCTTTCTCTCGTTTCTTCATCCTCTTCGTATCTACCTAAGAACACAAAACATAACATTGCCATTA

ACGTATTGTTCAGCCATATCTGTGACTCTTCATGTTCCTTTGAATTTTCTTCTTGTGATCCATTTCAAAATGGGTGTT

GTAGGGGTGGCAATAGCAATGATTTGGTTCAATCTCAACCTTTTGATTTTCCTTTCATCTTTTGTATTTTTCTCACGT

GTTTATAAAGATTCATGGGTTTTTCCTAGTATGGATTGTCTCAAAGGTTGGTCTTCGTTACTTGCGCTTTCGATTCCG

TCGTGTGTTTCTGTCTGTTTAGAATGGTGGTGGTATGAACTCATGATAATTTTGTGTGGACTTTTGGTCAACCCAAAG

TCAACCATTTCTTCAATGGGTATTCTTATTCAAACAACTTCTTTGGTTTATGTTTTTCCATCTTCTCTTAGTTTTGGT

GTTTCAACTAGAGTTGGGAATTTGTTAGGTGCTAATTCTCCTTCAAAAGCACGTTTTTCTATGATAGTGTCAATGTTT

TGTGGTTTTGGTTTGGGAATTTTGGCTATGGTTTTTACTACTTTGATGAGAAATCAATGGGGGAGATTGTTTACTAGT

GATGACGAGATTCTTAATCTAACGGCTATGGTTTTGCCAATTGTTGGCCTTTGTGAGATTGGAAATTGCCCACAAACA

ACGGGTTGTGGTGTTTTGAGAGGTAGTGCTAGACCTACGGTGGGTGCAAATATCAATTTGGGGTCTTTTTATTTGGTA

GGTATGCCTGTGGCTATTGTTCTTGGGTTTGTTGTGAAGATGGGTTTTGTTGGGCTTTGGTTTGGGTTGCTTGCAGCC

CAAGGCTCTTGTGCTGTTCTCATGTTGTATGTGCTTTGTACAACGGATTGGAATGATCAAATTGAAAGATCAAAGAAT

CTCACAAAAGCTACTACTACTACTATTGGTTTTTCTGATTCTACATTCATCACAAAAACAGTACTGCGTCATGATAAC

AACAACAATAATCACTGTGGTTGTCTTGAAGAGATCATAGTAATCACACATGATGATGCTACCAAGACATGTACACAC

TCACTTGAATCAGACCCACTGCTACCAAACATGTAG

>MsMATE30

ATGTGTCATTTAACTTCTCAACCTCCTTCCAAATGCAATTCAAAATCTGAATATCTAATAGTTTCAATCAAAGACACAAA

AGAGTCCAACAACAACATGATGACCAATCCACTTATCCAAAAAGACACAAACATAGAAAATCCAACAACACAATTCCAAA

AAACCCATCTTAGAGCTACCTTCAAAGAAGTTATTTCTATATCCAAAATTGCTTTTCCAATGATTTTCACCGGTCTCTTA

CTCTATTGTCGTTCAATGATCTCCATGCTCTTTCTTGGTCACCTTGGTGAACTTGCCTTAGCCGGTGGTTCACTTGCGGT

TGGCTTCGCGAACATTACCGGTTACTCAATCCTCTCCGGTCTCGCTGTAGGAATGGAACCTATTTGTGGACAAGCCTTTG

GTGCCAAAAGATTCACTCTCCTTGGTCTATGTTTACAAAAAACCATTCTCTTGCTACTCTTAACTTCTATCCCTATTTCG

TTACTTTGGCTTTACACAAAACATATCCTTCTTTTATGTGGCCAAGAAGAAGATATAGCTACACAAGCTCAAATCTATCT

TCTATATTCCATTCCTGATCTCTTAGCACAATCTTTTTTACCCCCTTTAAGAATTTACCTTAGAAGCCAATCCATTACTC

TGCCTCTCACACTTTGTGCTACTTTAGCAATTTTTCTTCACATTCCCATAAACTATTTTCTTGTTTCTCACCTCAACATG

GGAATCAAAGGTGTAGCTTTAAGTGGGGTGTGGACAAATTTCAACCTTGTTGCATCTTTGATTCTCTACATAATTTTCTC

TGGCACACACAAGAAAACATGGGGTGGTTTTTCATCAGAATGTTTCAAACAATGGAAATCACTTCTTAATTTAGCTGTAC

CAAGTTGTCTTTCTGTTTGCCTTGAATGGTGGTGGTATGAAATCATGATTTTGTTATGTGGTTTGTTGATAAATCCAAGA

GCAACCGTTGCTTCTATGGGAATTTTGATTCAAACTACTTCTTTGCTATATATTTTTCCATCATCAATAAGTTTTAGTGT

TTCCACTAGAGTTGGTAACAAACTTGGTGCTCAAAAGCCATCAAAAGCAAAACTTTCAGCCATAGTAGGACTCTCTTGTA

GCTTCATTTTAGGTGTGTTTGCTTTGTTTTTTGCTATAATGGTTAGGAACATTTGGGCTAGCATGTTCACTGAAGACAAA

GAGATAATAAAAATTACATCTTTAGTGTTACCTCTAATAGGGCTATGTGAACTTGGAAATTGTCCACAAACAACAGGTTG

TGGGGTGTTAAGAGGAACAGCAAGGCCTAAAGTAGGTGCAAATATCAATTTTGGTTGTTTTTACATTGTTGGAATGCCTG

TGGCAATTTGGTTAGCTTTTTATGTTGGGTTTGATTTTCAAGGTTTGTGGCTTGGGTTGCTTGTGGCTCAGGGAACTTGT

GCTGTGACTATGTTGGTTGTTTTGAGTCAAACAGATTGGGATTGTGAAGCACTAAGAGCTAAGAAATTAACTGGAATTGG

AGAAGCAACAACAAAAAATGATGTTTTTGTTGATGACAGCAAAGAAGTTGATGAAGAGAAGTTACTTAAAGCAGAAATTA

AGGAATATTCTTCTTAA

>MsMATE31

ATGTGCAAGTTATCATCATCATCAACCTCTGTCTCCACTTTGTATGAAAGCAATAACAACCAAACAAATAATATATCCAC

AACCACAATCAAAACACAGAATAATATCAAACCTGACATGTTAACACCGTTGATCCCTAAATCCCCAACATTCAAACAAC

AAAAGAAAACCCATTTTTCTCTCGCCCTCAATGAAGCCAAACATATTTCAAACATAGCATTACCTATGGTTTTAACAGGT

TTATTACTTTATTCTCGTTCCATCATTTCAATGTTGTTCCTTGGTCGTGTTGGTGAGCTTGCTTTAGCAGGTGGGTCACT

TGCCATTGGATTTGCAAACATAACAGGTTACTCCATTCTCTCTGGTCTTGCCATGGGAATGGAACCTATTTGTGGTCAAG

CTTTTGGTGCTAAAAGATTCAAACTCCTTGGTTTAACAATGCAAAGAACAGTGATTCTTCTTCTTGTAACTTCAATTTTC

ATTTCATTCTTATGGCTTAACATGAAGAGATTATTACTTTTATGTGGCCAACAAGAAGATATAGCAAACGTTGCACAATC

TTACATTCTTTATTCTCTTCCTGATCTTGTAGCACAATCATTGCTACACCCTTTGAGAATCTATCTTCGAAGCCAATCCA

TAACACTTCCTTTAACATATAGTGCTACTTTGTCTATTCTTCTTCACATTCCTATAAACTATTTTCTTGTCAATGTTCTT

CAATTGGGAATAAGAGGAATCGCTTTAGGTTCCGTTTGGACGAATTTCAACCTCGTTGTTTCGCTGATTATTTACATTTG

GGTTTCTGGAACACACAAGAAAACATGGAGTGGAATTTCCTCTGCTTGTTTCAAAGGATGGAAATCGCTTTTGAATTTAG

CAATTCCAAGCTGCATTTCGGTTTGTCTCGAATGGTGGTGGTACGAAATCATGATTTTGCTTTGTGGGTTATTGCTTAAT

CCACATGCAACTGTTGCATCTATGGGTGTGTTGATTCAAACCACTGCATTGATATACATTTTTCCTTCTTCTTTGAGTTT

TGGTGTTTCAACAAGAGTTGGAAATGAACTTGGTGCTGAAAACCCACAAAAAGCAAAACTTGCAGCAATAGTTGGACTCT

GTTTCAGTTTTGTTTTGGGTTTCTCCGCTTTGTTTTTCGCCTTTTCTGTTAGAAACATTTGGGCTACTATGTTCACAAGT

GATCCACAAATAATTGCTTTAACATCAATGGTGTTACCGATTATAGGACTATGTGAGCTTGGAAATTGTCCACAAACAAC

TGTTTGTGGTGTTTTGAGGGGAACGGCGAGGCCGAAATTAGGCGCAAATATAAATTTAGGTTGTTTTTATCTTGTGGGAA

TGCCTGTTGCAGTTTGGTTGAGTTTCTTTGCTGGATTTGATTTCAAAGGTTTGTGGTTTGGTCTTATGGCTGCTCAAGGT

TCATGTATGGTTACAATGTTGTTTGTTTTGGTTCGTACAAATTGGGAAAATCAAGCAGAGAGAGCTAAGGAGTTAACATC

ATCAGATTCAAGTGAAGAAGAACAAGAGGAAGAGAAAGTGATTATCAACTCAAGTTCATGTGGCACAAAAGAGTGCTCTG

ATTCATTAGTTTGA

>MsMATE32

ATGGCGGCAAGTATCATTTCAGATGGCACAGAAGCACCTCTATTAGCAGATGATCATGGAAAACAAAACACAAGACCACA

AATAGAGAAATGGTGGAACAAAATCTTGGACATAGAGGAAGCCAAAATTCAACTCATGTTTTCATTACCAATGATTCTTA

CAAATTTATTTTATTACTTGATTACTTTGGTTTCTGTCATGCTTGTTGGTCATCTTGGTGAGCTTCAGTTAGCTGGTGCT

ACTCTTGCTAATTCTTGGTTTAGTGTCACTGGGGTAGCAGTTATGGTTGGTTTAAGTGGTGCACTAGAAACACTCTGCGG

ACAAGGATTTGGCGCAAAAGAATATCACATGTTGGGAATTTATCTACAAGGCTCTTGCATTATATCTTTCATTTTTTCAA

TCATTATATCAATTGTTTGGTTCTACACAGAACAAATTCTAGTGCTACTTCATCAATCACAAGACATTGCTAGAACAGCA

GCACTCTATATGAAGTTTCTTATACCAGGATTATTTGCATATAGCATCTTGCAAAACTTGTTGAGGTTTCTACAAACACA

ATCTGTAGTAATGCCACTGGTTATACTTTCAGCTATACCGGCATTAGTTCATGTTGGAATTGCATATGGATTTGTTCAAT

GGACTGGTTTAAATTTCATAGGTGGACCGACTGCAACTTCTATTTCACTATGGATATCAATGATAATGTTAGGATTATAT

GTAATGTATGCAAAGAAATTTAAGAATACATGGAGAGGATTCTCAATGCAATCATTTGATTACCTGCTTGCAAACATAAG

ACTAGCTTTGCCTTCTGCTGCAATGGTATGTTTGGAGTATTGGGCTTTTGAAGTTTTGGTTTTCTTAGCTGGATTAATGC

CTGATTCACAAATAACAACTTCATTGATTGCAATATGTGCCAACACAGAATTCATTGCTTACATGATCACTTATGGTCTT

AGTGCAGCTGCAAGCACAAGAGTTTCCAATGAATTGGGAGCAGGCCAACCAGAAAGAGCAAAACATGCAATGGGAGTCAC

CCTAAAGCTCTCTCTCCTCCTTGGTTTATGTTTTGTTTTGATACTTGTATTTGGCCATGATATATGGATTCAGCTTTTTA

GTGATAGTCCTATTATCAAAAAGGAGTTTGCTTCAGTGACACCTTTGCTTGCTATTTCCATACTACTAGATTCTGTCCAA

GGTGTCTTATCAGGGGTGGTTAGAGCATGTGGTTGGCAGTACGTAGCTGTTTATGTCAACCTTGCAACTTTTTATCTCAT

TGGTTTACCAATTTCATGTCTCCTTGGATTTAAGACCGATTTGCAGTATAAGGGTTTATGGATTGGTCTGATATGTGGAT

TGGTGTGTCAAACTGGGGCACTCTTACTTTTGACAAGGCATGTCAAATGGACTAAACTGAATCTCTCAGGGGACAAAGAT

AAAGACCAACCTATTGTTGTTTAA

>MsMATE33

ATGGCAACAAGTGGCATTTCAGATGGCACAGCAACACAGAACACAGAACCTCAAATGGAGAAGAAATGGTGGAACAAAAT

CTTGGACATTAAGGAAGCCAAACATCAACTCATGTTTTCACTGCCAATAATTCTTACAACAATATTATACTACTCAATCA

ATTTGGTTTCTGTCATGCTTGTTGGTCATCTTGGTGAGCTTCAGTTAGCTGGTGCTACTCTTGCTAACTCCTGGTTTGGT

GTCACTGCCGTGGGGGTTATGGTTGGTTTAAGTGGTGCATTGGATACACTCTGCGGACAAGGATTTGGTGCAAAAGAATA

TCACATGTTGGGAATTTATTTACAAAGCTCTTGCATTATATCTTTTATTTTTTCAATCATTATATCAATTATTTGGTTCT

ATACAGAACAAATTCTAGTGCTACTTCATCAATCACAAGACATTGCTAGAACAGCTGCACTCTATATGAAGTTTCTTATA

CCAGGATTATTTGCATTTGGCACCTTGAGAAACATGTTGAGGTTTCTGCTAACACAATCTGTAGTGATGCCACTGGTTAT

CCTTTCAGCTATCCCAGCAATTGTTCATGTTGGTATTGCATATGGATTTGTTCATTGGTCAGGTCTTAATTTCAAAGGTG

GACCAGTTGCAACTTCTATTTCACAATGGTTATCTATGATATTGGTAGGTTTTTATATCTTGTATGCAAAAAAGTTTAAG

AATACATGGAGAGGATTTTCAATGCGATCATTTCAGTACTTGTTTACAAACTTGAAACTAGCTCTTCCCTCTGCAGCAAT

GCTATGGTATGAGTTTCTAAAAGCCTTTCACTTTGTTTGTCCCTTTTGA

>MsMATE34

ATGGCAGCAAGTGGCATTTCAGATGGCACAGCAACACAGAACACAGAACCTCAAATGGAGAAGAAATGGTGGAACAAAAT

CTTGGACATTAAGGAAGCCAAACATCAACTCATGTTTTCACTGCCAATAATTCTTACAACAATATTATACTACTCAATCA

ATTTGGTTTCTGTCATGCTTGTTGGTCATCTTGGTGAGCTTCAGTTAGCTGGTGCTACTCTTGCTAACTCCTGGTTTGGT

GTCACTGCCGTGGGGGTTATGGTTGGTTTAAGTGGTGCATTGGATACACTCTGCGGACAAGGATTTGGTGCAAAAGAATA

TCACATGTTGGGAATTTATTTACAAAGCTCTTGCATTATATCTTTTATTTTTTCAATCATTATATCAATTATTTGGTTCT

ATACAGAACAAATTCTAGTGCTACTTCATCAATCACAAGACATTGCTAGAACAGCTGCACTCTATATGAAGTTTCTTATA

CCAGGATTATTTGCATTTGGCACCTTGAGAAACATGTTGAGGTTTCTGCTAACACAATCTGTAGTGATGCCACTGGTTAT

CCTTTCAGCTATCCCAGCAATTGTTCATGTTGGTATTGCATATGGATTTGTTCATTGGTCAGGTCTTAATTTCAAAGGTG

GACCAGTTGCAACTTCTATTTCACAATGGTTATCTATGATATTGGTAGGTTTTTATATCTTGTATGCAAAGAAGTTTAAG

AATACATGGAGAGGATTTTCAATGCGATCATTTCAGTACTTGTTTACAAACTTGAAACTAGCTCTTCCCTCTGCAGCAAT

GCTATGTTTGGAGTCTTTGGCCTTTGAAGTCTTGGTTTTCTTAGCTGGATTAATGTCTGACTCACAAATAACAACTTCAT

TGATTGCAATATGTGAAAACACAGAATTCATTGCTTACTTGATCACTTATGGTCTTAGTGCAGCCGCAAGCACAAGGGTT

TCCAATGAACTTGGGGCAGGCCAACCAGAAAGAGCCAAACATGCAATGAGAGTCAGTCTAAAGCTCTCTCTCCTCCTTGG

TTTATGTTTTGCTTTGATGATTGTATTTGGTCATGATATATGGATTCGGCTGTTTAGCAGTAGTCCTACTATCAAACATA

AGTTTGCTTCAATATCACCCTTCCTTGCTATTTCCATACTACTTGATTCTGTCCAAGGTGTCTTATCAGGGGTGGTTAGA

GCATGTGGTTGGCAGCACGTAGCTGTTTATGTCAACCTTGCAACTTTTTATCTCATTGGTTTACCAATTTCATGTATCCT

TGGATTTAAGACCAATTTGCAATATAAGGGTTTATGGATTGGTCTGATTTGTGGGCTGGCATGTCAAACTGTGACACTCT

TACTTTTGACAAGATATGCCAAATGGACTAAACTGAATCTCTCAGGAGACAAAGATAAAGATCAACCTGTTGTTGTTTTA

ACAACTGAATGCATGCCAATTAGGACTGAATAA

>MsMATE35

ATGAAGTTTCTTATACCAGGATTATTTGCATATAGCATCTTGCAAAACTTGTTGAGGTTTCTACAAACACAATCTGTA

GTAATGCCACTGGTTATACTTTCAGCTATACCGGCATTAGTTCATGTTGGAATTGCATATGGATTTGTTCAATGGACT

GGTTTAAATTTCATAGGTGGACCGACTGCAACTTCTATTTCACTATGGATATCAATGATAATGTTAGGATTATATGTA

ATGTATGCAAAGAAATTTAAGAATACATGGAGAGGATTCTCAATGCAATCATTTGATTACCTGCTTGCAAACATAAGA

CTAGCTTTGCCTTCTGCTGCAATGGTATGTTTGGAGTATTGGGCTTTTGAAGTTTTGGTTTTCTTAGCTGGATTAATG

CCTGATTCACAAATAACAACTTCATTGATTGCAATATGTGCCAACACAGAATTCATTGCTTACATGATCACTTATGGT

CTTAGTGCAGCTGCAAGCACAAGAGTTTCCAATGAATTGGGAGCAGGCCAACCAGAAAGAGCAAAACATGCAATGGGA

GTCACCCTAAAGCTCTCTCTCCTCCTTGGTTTATGTTTTGTTTTGATACTTGTATTTGGCCATGATATATGGATTCAG

CTTTTTAGTGATAGTCCTATTATCAAAAAGGAGTTTGCTTCAGTGACACCTTTGCTTGCTATTTCCATACTACTAGAT

TCTGTCCAAGGTGTCTTATCAGGGGTGGTTAGAGCATGTGGTTGGCAGTACGTAGCTGTTTATGTCAACCTTGCAACT

TTTTATCTCATTGGTTTACCAATTTCATGTCTCCTTGGATTTAAGACCGATTTGCAGTATAAGGGTTTATGGATTGGT

CTGATATGTGGATTGGTGTGTCAAACTGGGGCACTCTTACTTTTGACAAGGCATGTCAAATGGACTAAACTGAATCTC

TCAGGAGACAGAGATAAAGATCAACCTGTTGTTGTTTTAACAACTGAATGCATGCCAATTAGGACTGAATAA

>MsMATE36

ATGGAAAAGGGTTTGTTAGAGAAAGACAGAGAAGGTGGTTCAAGAAGCATAACATGGGGTGTATTTGTTCAAGAGGTGAA

AGATGTTTGTTTTCTAGCTCTGCCTATGATCGCTGTCACTTTGTCACAATATTTTCTACAAATTATTTCAATGATGATGG

TTGGTCGTTTGGGTAAACTTGCTCTTTCTAGCACAGCTATTGCTATCTCTCTTTGTGCTGTCTCTGGCTTCAGTCTTCTT

TTTGGAATGTCATGTGCACTTGAAACTCAATGCGGACAAGCTTATGGTGCAAAGCAATATAAAAAATTTGGTGTTCAAAT

TTACACTGCTGTCTTTTCTCTTATTATAGCTTGTCTTCCTCTATCTCTATTATGGATCTTCTTGGGAAGGCTATTGATTT

TACTCGGTCAAGACCCTCTGATTTCACAAGAAGCTGGAAAATTTGCTATGTGCATGATTCCTGCTCTTTTTGCTTATGCA

ACTCTTCAGGCACTGGTCAGATACTTTCTGATGCAAAGTTTGATTCTTCCTCTTGTTATAAGTTCCTCTGTTACCCTTGG

ATTCCATGTAGCTTTTTGTTGGTTATTAGTTTTTAAATCTGGATTAGGTAGCTTAGGAGCTGCATTTTCTATTGGCACTT

CATACTGGTTGAATGTGATTATCCTTGGATTATATATGAAATTCTCTGCTGATTGTGAAAAAACTCGGGTTACAATTTCA

ATGGAGTCATTCACTGGAATAGGTGAGTTCTTTCGCTATGCTATTCCTTCAGCTGGAATGATTTGCTTTGAATGGTGGTC

ATTTGAGCTCTTAGTCTTTCTTTCTGGTCTTCTACCAAATCCACAGCTTGAAACTTCTGTTTTATCCATATGTTTGTCAA

TCATCTCAACACTCTATACAATTCCAGAAGCAACTGGCTCAGCAGCAAGCGCAAGAGTTTCAAATGCATTAGGAGCTGGA

TGTCCACATGCAGCACGATTGTCTGTCTATGCTGCTATGGCTATCGCAGTTTCTGAGGCTATATTGGTGAGTTCCATCAT

TTTCGCCTCTCGACGGGTGTTAGGTTATATATTTAGCAATGAGCAGGATGTGGTGGAGTATGTCACAGATATGGCTCCTC

TGATAAGTCTATCTGTTATAGTGGATAGTTTACATGGTACCCTTTCAGGTATTGCTAGAGGATGTGGTTGGCAGAAGTCA

GGAGCATATGTGAACCTTGGATCCTATTATATTTTTGGAATTCCAATTGCTGCTATATTGGGTTTCTGGTTTGAATTGAG

AGGAAAAGGTCTTTGGATTGGGATAATTGTTGGTGCCTCCTGTCAAGCAGTTTTGCTATCCCTTATAACAAGTTTTACAA

ACTGGGAAAAACAGGCAATTAAGGCACGGGAAAGGATATTTCGAGAAGGTTTTGTGATAGAAGATAGACTAGTTTGA

>MsMATE37

ATGGAAAAGGGTTTGTTAGAGAAAGACAGAGAAGGTGGTTCAAGAAGCATAACATGGGGTGTATTTGTTCAAGAGGTGAA

AGATGTTTGTTTTCTAGCTCTGCCTATGATCGCTGTCACTTTGTCACAATATTTTCTACAAATTATTTCAATGATGATGG

TTGGTCGTTTGGGTAAACTTGCTCTTTCTAGCACAGCTATTGCTATCTCTCTTTGTGCTGTCTCTGGCTTCAGTCTTCTT

TTTGGAATGTCATGTGCACTTGAAACTCAATGCGGACAAGCTTATGGTGCAAAGCAATATAGAAAATTCGGTGTTCAAGT

TTACACTGCTATCATTTCTCTTATTATAGCTTGTGTTCCTCTCTCTCTATTGTGGCTCAACTTGGGGAAGTTACTGAGTT

TACTTGGCCAAGACCCTTTGATTTCACAAGAAGCTGGAAAATTTGCCATGTGCATGATTCCTGCTCTGTTTGCCTATGCA

ACGCTTCAGGCACTGGTCAGATACTTTCTGATGCAAAGTTTGATTCTTCCTCTTGTTATAAGTTCCTCTGTTACCCTTGG

ATTCCATGTAGCTTTTTGTTGGTTATTAGTTTTTAAATCTGGATTAGGTAGCTTAGGAGCTGCATTTTCTATTGGCACTT

CATACTGGTTGAATGTGATTATCCTTGGATTATATATGAAATTCTCTGCTGATTGTGAAAAAACTCGGGTTACAATTTCA

ATGGAGTCATTCGCTGGAATAGGTGAGTTCTTTCGCTATGCTATTCCTTCAGCTGGAATGATTTGCTTTGAATGGTGGTC

ATTTGAGCTCCTAGTCTTTCTTTCTGGTCTTCTACCAAATCCACAGCTTGAAACTTCTGTTTTATCCATATGTTTGTCAA

TCATCTCAACACTCTATACAATTCCAGAAGCAACTGGCTCAGCAGCAAGCGCAAGAGTTTCAAATGCATTAGGAGCTGGA

TGTCCACATGCAGCACGATTGTCTGTCTATGCTGCTATGGCTATCGCAGTTTCTGAGGCTATATTGGTGAGTTCCATCAT

TTTCGCCTCTCGACGGGTGTTAGGTTATATATTTAGCAATGAGCAGGATGTGGTGGACTATGTCACATATATGGTTCCTC

TGATAAGTCTAAATGTTATAGTAGATAGTTTACATGGCACCCTTTCAGGTATTGCTAGAGGATCTGGTTGGCAGAAGTTG

GGAGCATATGTGAACCTTGGAGCCTATTATGTTTTTGGAATTCCAATTGCTGTTATATTGGGTTTCTGGTTTGAGTTAAG

AGGAAAAGGACTGTGGATTGGGATACTTGTGGGTGCCTTCTGTCAAGCACTTTTGCTAGCCCTTATAACAGGTTTTACAA

ACTGGGAAAAACAGGCAATCAAAGCACGGGAAAGGATATTTCAAGGAAGACAGGAAGTTTTGCAGTAG

>MsMATE38

ATGGAAAAGGGTTTGTTAGAGAAAGACAGAGAAGGTGGTTCAAGAAGCATAACATGGGGTGTATTTGTTCAAGAGGTGAA

AGATGTTTGTTTTCTAGCTCTGCCTATGATCGCTGTCACTTTGTCACAATATTTTCTACAAATTATTTCAATGATGATGG

TTGGTCGTTTGGGTAAACTTGCTCTTTCTAGCACAGCTATTGCTATCTCTCTCTGTGGTGTCTCTGGCTTCAGTCTTCTT

TTTGGAATGTCATGTGCACTTGAAACTCAATGCGGACAAGCTTATGGTGCAAAGCAATATAAAAAATTTGGTGTTCAAAT

TTACACTGCTGTCTTTTCTCTTATTATAGCTTGTCTTCCTCTATCTCTATTATGGATCTTCTTGGGAAGGCTATTGATTT

TACTCGGTCAAGACCCTCTGATTTCACAAGAAGCTGGAAAATTTGCCATGTGCATGATTCCTGCTCTGTTTGCCTATGCA

ACGCTTCAGGCACTGGTCAGATACTTTCTGATGCAAAGTTTGATACTTCCTCTTGTTATAAGTTCCTCTGTTACCCTGTG

CTTCCATGTAGCTTTTTGTTGGCTGTTAGTTTTTAAATCTGGATTAGGTTGCTTAGGAGCTGCACTTTCTATCGGTACTT

CATACTGGTTGAACGTGATTATACTTGGATTATATATGAAATTCTCCACTGATTGTGAAGAAACTCGGGTTCCGATTTCA

ATGGAGCCATTCCTTGGAATAGGAGAGTTCTTTCGATACGCTATTCCATCAGCAGGAATGATTTGGTAG

>MsMATE39

ATGGATCTTCTTGGGAAGGCTATTGATTTTACTCGGTCAAGACCCTCTGATTTCACAAGAAGCTGGAAAATTTGCCATGT

GCATGATTCCTGCTCTGTTTGCCTATGCAACGCTTCAGGCACTGGTAGCTTAGGAGCTGCATTTTCTATTGGCACTTCAT

ACTGGTTGAATGTGATTATCCTTGGATTATATATGAAATTCTCTGCTGATTGTGAAAAAACTCGGGTTACAATTTCAATG

GAGTCATTCGCTGGAATAGGTGAGTTCTTTCGCTATGCTATTCCTTCAGCTGGAATGATTTGCTTTGAATGGTGGTCATT

TGAGCTCCTAGTCTTTCTTTCTGGTCTTCTACCAAATCCACAGCTTGAAACTTCTGTTTTATCCATATGTTTGTCAATCA

TCTCAACACTCTATACAATTCCAGAAGCAACTGGCTCAGCAGCAAGCGCAAGAGTTTCAAATGCATTAGGAGCTGGATGT

CCACATGCAGCACGATTGTCTGTCTATGCTGCTATGGCTATCGCAGTTTCTGAGGCTATATTGGTGAGTTCCATCATTTT

CGCCTCTCGACGGGTGTTAGGTTATATATTTAGCAATGAGCAGGATGTGGTGGACTATGTCACATATATGGTTCCTCTGA

TAAGTCTAAATGTTATAGTAGATAGTTTACATGGCACCCTTTCAGGTATTGCTAGAGGATCTGGTTGGCAGAAGTTGGGA

GCATATGTGAACCTTGGAGCCTATTATGTTTTTGGAATTCCAATTGCTGTTATATTGGGTTTCTGGTTTGAGTTAAGAGG

AAAAGGACTGTGGATTGGGATACTTGTGGGTGCCTTCTGTCAAGCACTTTTGCTAGCCCTTATAACAGGTTTTACAAACT

GGGAAAAACAGGCAATCAAAGCACGGGAAAGGATATTTCAAGGAAGACAGGAAGTTTTGCAGTAG

>MsMATE40

ATGGGTGAAGAGAGAAGAGTGATGGAAGAGAGTCTCTTATCAAAACAAATTGATTCGAAAGCAGAGAATGACAATGAAGA

AGAGAGAAAGAATAGAGAAATTAGTTGGGATATTTACACGAAAGAATTGAAGAGGATTTGTTACCTATCAGGTCCTATGG

TAGCTGTAACATCTTCACAATATCTTTTACAGGTTGTGTCCATCATGATTGTTGGTCACTTAGGTGAACTCTATCTCTCT

AGTGCCGCCTTAGCCATTTCCTTTACTGGTGTCACCGGTTTCAGCTTCCTGATGGGAATGGCTAGTGGACTAGAAACAAC

ATGTGGACAGGCTTATGGAGCTAAACAATATCAAAGAATAGGAGTACAAACATACACATCTATATTTTCTCTCATATTGG

TTTGTTTACCACTCTCTTTTATTTGGATCAACATTGAGAACATACTAGTTTTCACAGGACAAGACCCTCTAATTGCACAT

GAAGCAGGAAGATTCACAATTTGGCTTCTTCCAGCACTTTTTGCATATGCAATATTGCAACCACTAGTTAGATATTTTCA

AATACAAAGCTTGCTTATTCCAATGCTTTTAAGCTCTTGTGTCACTCTTTGTATCCATATACCTCTTTGCTGGGCTTTGG

TATTCAAGACAGGATTGAGTAATATTGGTGGTGCAATTGCAATGAGCATTTCCATTTGGTTAAATGTGATTTTTCTTGGA

TTATACATGAGGTACTCTTCCTCATGCGCAAAAACTAGAGCGCCAATTTCTATGGAACTATTTCAAGGAATTTGGGAGTT

TTTTCGCTTCGCTATACCTTCTGCTGTGATGGTTTGCCTTGAGTGGTGGTCATATGAGCTAATTGTCTTGCTCTCTGGAC

TTTTACCTAATCCACAACTTGAAACTTCAGTTCTATCAGTTTGTCTCAATACCATTGCAACTCTCTATACAATACCATTT

GGAATTGGTGCTGCAGCAAGTACAAGGGTTTCAAATGAATTAGGAGCTGGTAATCCATTTGAAGCACGTGTTGCCGTGTT

AGCTGCTATGTCACTTGCACTCACTGAAGCAAGTATAGTGAGCGCAACCCTCTTTGCATGCCGCCATGTTTATGGCTACA

TTTTTAGTAGTGATACGGAAGTTGTTAAATACGTCACTGTCTTGGCTCCTCTGGTTTCTATATCTGTTATACTCGACAGC

ATACAAGGCGTTCTCGCAGGGGTTGCTAGAGGTTGTGGGTGGCAACACATAGGAGTTTATGTGAATCTAGGAGCCTTCTA

CCTATGTGGGATTCCTGTTGCTGCTGCATTGGCATTTTGGGTTCAAGTGGGAGGAAAAGGACTTTGGATTGGTATACAAG

TCGGTGCTTTTGTTCAATGTATTCTACTTTCTATCATAACATGTTGTATAAATTGGGAGCAACTGGCAATTAAGGCTAGA

CAGAGGTTGTTTGATGTTCAATTTTCTGGAGAGAATAGATTGGTATGA

>MsMATE41

ATGATCATGAAGGAAACAATGGAAGAAGGTTCAAATAATAACAAGTGTGAATGGATAAAAACCAGGACTACATTAATGGA

GGAGCTGAAGAAAATGGGTACTATAGCAGTACCAATGGTGGCAACGAGTGTGCTACAATATCTTCTACAAGTAGTATCGG

TTATGATGGTTGGACATCTTAACCAACTCTCTCTTTCAAGTGTTGCTATTGCTACATCTCTCACTAATGTTTCTGGGTTT

AGTATTTTGTCAGGGATGGCTGGTGGATTAGAAACTTTATGTGGCCAAGCTTATGGAGCAGGACATTTTGAAAAACATGG

AATATATACATATACTGCAGTAATATCCCTCACTATGGTTTGTGCACCAATCACTATTATATGGACTTTCATGGACAAAA

TATTAATTCTCATAGGTCAAGACCCCACAATCTCCCTTCAAGCTCGTACATTTGCACTTTGGCTAATACCTGCTTTATTT

GCCTCAGCAATTCTTAAACCTCTCACACGTTTTTTCCAAACTCAAAGTTTAATTTTTCCAATGATTATTAGTTCATTTAT

AGTTTTGTGCTTCCATGGAGTAATGTGTTGGACATTAGTATTCAAATTAGGGTTAGGGCATGTTGGTGCTGCAATTTCCT

TTAGTTTAGGAACTTGGTTGAATGTGTTGATACTTTTGTCCTTTGTGAAATATTCATCTTCTTGTGAGAAAACACGTGTG

CCATTTTCCATGAAGGCTTTTCTTGGTATTAGAGAGTTCTTTGGTCTTGCTGTTCCATCAGCAGCTATGGTTTGTCTTAA

ATGGTGGGCATGTGAGTTGCTTGTTTTGCTAGCTGGACTTTTTCCAGATCCAAAGTTGGAGACATCAGTTCTTTCTATAT

GTTTGACAATATCGACATTGCACTTCACCATATCCTATGGGCTTGGGGCAGCTGCTAGCACAAGAATTTCAAACGAATTA

GGAGCTGGGAATCCAAAAGCAGTTCGTTTTTCTATTTGTACAGCAATGTTCCTTGCAACTACAGAGGCTCTTATTATAAC

TGCAATCTTATTAGGTTGCAGATGTGTTTTGGGTTATGCTTATACCAATGATACTATGGTTGTTCATTATGTGGCTGGTA

TGACACCTTTGCTATGTGTATCAATTTTTACAGACAGCTTGCAAGCAGTTCTTTCAGGGGTTGCTAGAGGAAGTGGGTGG

CAATATGTTGGAGCTTATGTTAACCTTGGAGCATTTTATCTAGTAGGAATTCCTATAGGGGTGGTATTGGGTTTCATTGT

ACATTTCAAAGCAAAGGGACTTTGGATTGGAATAGTTGCTGGCTCCATTGTCCAAACAATTTTTCTTTCCATCATCACAT

CTCTTACAAATTGGAAAAAACAGGCAATCATGGCAAGAGAGAGAATATTTGATGCCACTTCTTCTGATGAAAGTGTAACA

GATCACATGACCAGAGCATAA

>MsMATE42

ATGGGTAAGGAAGAAGCAACACCTCTGCTAACAAAGAATGAAGAGAATGATGAGTTGGCACCATTGGAAGGTGCCTTTTG

GGCAGAGTTCAAGAGGGTGGGATCCATGGCAGCTCCAATGGTGACTGTAACTGTATCTCAATACCTTCTTCAAGTTGTGT

CGTTGATGATGGTTGGACATATTGGAATACTAACTTCCTTCTCTGGTGTTGCACTTGCTATCTCTTTTGCTGAATGTACT

GGCTTCTGTGTTCTTATTGGAATGGCAGGTGCATTAGAAACTTTATGTGGCCAAACCTACGGAGCTGAAGAATTCAGCAA

GATTGGAAACTACATTTGCAGTGCAATGATCACCTTGATTTTGGTTTGTTTCCCCATATCACTCATGTGGATATTCATTG

ATAAATTACTCTTGCTTTTCGGTCAAGACACTGAAATTGCTCAAGCAGCTCGCGAATATTGCATATGCTTGATCCCAGCA

CTTTTTGGCCATGCTGTTCTTCAATCTTTGATTCGCTACTTCCAGATTCAGAGTATGATCTTTCCAATGGTTTTCAGCTC

CATTGTAATTTTGTGTTTGCATGTACCTATTTGTTGGTGTCTGGTATTTAAATTGGGACTAGGACATGTTGGAGCAGCAT

TTGCAATTGGTATTGCTTATTGGTTGAATGTGATTTGGCTTGGAATTTATATGAAGTATTCTCCAGCTTGTGAAAAAACG

AAGATTGTGTTTTCTTACAATTCCTTACTATACATTGCTGAGTTCTGCCAATTTGCCATCCCTTCTGGACTAATGTTTTG

TCTTGAATGGTGGTCATTTGAGATACTTACAATCGTTGCCGGGCTTTTACCTAATTCGCAGCTCGAAACCTCAGTTCTTT

CAGTATGTCTTAGCACAACTACATTGCACTACTTCATTCCTCATGCAATTGGAGCTTCTGCAAGTACTCGGGTTTCGAAT

GAATTAGGAGCAGGGAATCCAAGGGCGGCAAAAGGCGCTGTTCGAGTTGCTGTAATTATTGGAATTGCTGAGGCAGTTAT

AGTCAGCACCTTGTTTCTTTGTTTTAGGAATTTTATAGGAAATGCTTATAGCAATGACAAGGAAGTTGTGGATTATGTTA

CAGACATGGTTCCTCTTCTTTGTGTGTCTGTTAGTGCAGATAGTATAATATGTGCTCTTTCAGGGATTGCAAGAGGTGGA

GGATTTCAGACAATAGGGGCTTATGTGAACCTTGGAGCCTATTATCTTGTAGGTGCTCCTATAGCATATTTTTTGGGTTT

TGGCCTAAAGCTTAATGCCAAGGGACTCTGGATGGGAACACTAACAGGATCTATTCTTAATGTAATCATACTAGCTGTTG

TAACAATGTTAACAGATTGGCAGAAAGAGGCAACAAAAGCAAGGGAAAGAATAGCTGAGAAGCCTATTGAAGCTCATGAT

GGATCAATATGA

>MsMATE43

ATGACCATGGAAGGAGTTGAGATTGAGAATACATTCATTCAAGAGTTGAAGAAAGTGAGTTTCATGGCAGCTCCAATGGT

AGCTGTAACTGTTTCACAATATCTTCTTCAAGTGGTTTCTCTTATGATGGTGGGACATCTTGGTATACTTGTTTCATTCT

CTGGTGTTTCCATTGCCATGTCTTTTGCTGAAGTTACTGGCTTTAGTGTCCTTTTGGGAATGGCTGGTGCATTGGAAACA

CTATGTGGACAAACCTTTGGTGCAGAGGAGTATGGAAAACTTGGAAACTACACTTGCTGTGCAATATTAACTCTAACTGT

AGTTTGTTTCCCAATATCTCTTGTGTGGATATTCACAGATAAAATACTACTGTTTTTTAGTCAAGACCCTGGAATGTCTC

ATGTAGCTCGCGAGTACTGCATATACCTCATTCCCGCTCTATTTGGCTATGCACTTCTTCAAGCGTTGATTCGCTATTTC

CAGACTCAGGGAATGATCTTTCCCATGGTTTTTAGCTCAGTATCTGCTCTATTTTTGCACATTCCTATTTGTTGGATTCT

GGTTTTTAAGTTGGGATTAGGACACATTGGAGCTGCTTTAGCTATTGGAATTTCTTATTGGTTGAATGTGATATGGCTTT

GGGTTTATATTAAGTACTCTCCATCATGTCAAAAAACCAAGATTGTGTTTTCTACTCATGCTTTACATAACCTACCAGAG

TTCTGCAAATATGCTATTCCTTCTGGACTCATGTTTTGTTTTGAATGGTGGTCCTTTGAGATTCTTATTTTAATTGCCGG

GCTTTTACCTAATCCTCAACTTGAAACCTCGGTTCTTTCTGTCTGCTTGAACACTACCTCATTGCACTTTTTCATTCCAT

ATGCAATTGGAGCTTCTGCAAGTACTCGTGTTTCAAATGAATTAGGAGCAGGAAATCCAAAGACAGCTAAGGGAGCTGTT

AGAGTGGTTGTGATTATTGGAATTGCTGAGGCAATTATTGTCAGCACTTTCTTCCTTTGCTTTAGAAATATTTTAGGATA

TGCTTATAGCAATGATGAACAAGTTGTGAATTACATTGCGGATATGGTTCCTCTTCTTTGTGTTTCTGTTAGTGCTGATA

GTCTAATTGGAGCTCTTTCAGGGGTTGCTAGAGGAGGTGGATTTCAAGAAATGGGGGCTTATGTGAACCTTGGAGCTTAC

TATATTGTGGGAATTCCTATTGGTTTGTTATTAGGTTTTCATCTAAAATTAAATGCAAAGGGATTGTGGATGGGAACTCT

ATCAGGATCTGTTTTAAATGTCATTATACTATCTATTGTAACTGCATTAACTGATTGGCAGAAAGAGGCAACAAAAGCAA

GGGAGAGGATAATTGACCAGTCAATAAAAACTAACAACACATTGGTGGTGGCATGA

>MsMATE44

ATGAAAAACTCTAGAGAGATGAGTAAGGAGGTGACAACACCATTGCTAAGAAAGAGTGATGACCATGGAAGTGATGGAAG

AGTTGAGATTGAGAATACATTCATTCAAGAGTTGAAGAAAGTGAGTTTCATGGCAGCTCCAATGGTAGCTGTAACTGTTT

CACAATATCTTCTTCAAGTGGTTTCTCTTATGATGGTGGGACATCTTGGTATACTTGTTTCATTCTCTGGTGTTTCCATT

GCCATGTCTTTTGCTGAAGTTACTGGCTTTAGTGTCCTTTTGGGAATGGCTGGTGCATTGGAAACACTATGTGGACAAAC

CTTTGGTGCAGAGGAGTATGGAAAACTTGGAAACTACACTTGCTGTGCAATATTAACTCTAACTGTAGTTTGTTTCCCAA

TATCTCTTGTGTGGATATTCACAGATAAAATACTACTGTTTTTTAGTCAAGACCCTGGAATGTCTCATGTAGCTCGCGAG

TACTGCATATACCTCATTCCCGCTCTATTTGGCTATGCACTTCTTCAAGCGTTGATTCGCTATTTCCAGACTCAGGGAAT

GATCTTTCCCATGGTTTTTAGCTCAGTATCTGCTCTATTTTTGCACATTCCTATTTGTTGGATTCTGGTTTTTAAGTTGG

GATTAGGACACATTGGAGCTGCTTTAGCTATTGGAATTTCTTATTGGTTGAATGTGATATGGCTTTGGGTTTATATTAAG

TACTCTCCATCATGTCAAAAAACCAAGATTGTGTTTTCTACTCATGCTTTACATAACCTACCAGAGTTCTGCAAATATGC

TATTCCTTCTGGACTCATGTTTTGTTTTGAATGGTGGTCCTTTGAGATTCTTATTTTAATTGCCGGGCTTTTACCTAATC

CTCAACTTGAAACCTCGGTTCTTTCTGTCTGCTTGAACACTACCTCATTGCACTTTTTCATTCCATATGCAATTGGAGCT

TCTGCAAGTACTCGTGTTTCAAATGAATTAGGAGCAGGAAATCCAAAGACAGCTAAGGGAGCTGTTAGAGTGGTTGTGAT

TATTGGAATTGCTGAGGCAATTATTGTCAGCACTTTCTTCCTTTGCTTTAGAAATATTTTAGGATATGCTTATAGCAATG

ATGAACAAGTTGTGAATTACATTGCGGATATGGTTCCTCTTCTTTGTGTTTCTGTTAGTGCTGATAGTCTAATTGGAGCT

CTTTCAGGGGTTGCTAGAGGAGGTGGATTTCAAGAAATGGGGGCTTATGTGAACCTTGGAGCTTACTATATTGTGGGAAT

TCCTATTGGTTTGTTATTAGGTTTTCATCTAAAATTAAATGCAAAGGGATTGTGGATGGGAACTCTATCAGGATCTGTTT

TAAATGTCATTATACTATCTATTGTAACTGCATTAACTGATTGGCAGAAAGAGGCAACAAAAGCAAGGGAGAGGATAATT

GACCAGTCAATAAAAACTAACAACACATTGGTGGTGGCATGA

>MsMATE45

ATGTTGATAGTTTATATATGTGGGTTTTCTAAACATAGCAATTGGAAGAGGAAGGTAGCTAGGGCCCCTAGGCAACAAAT

GAGAGAGATGATGATTGGCATATGCAGAAAGGATATTACAGAAGAAGTGAAGAAGCAGCTATGGCTTGCAGTGCCAATGA

TATTTGCTAGTGTTTTTCAATATAGCTTACAAATGATATCTCTCATGTTTATTGGCCATATGAATGATGAGGTGCTTCTA

GCTGGTGCTGCTTTGGCAAATTCAGTTACGAGTGTTTTTGGTTATAGTGTATTGGTAGGTTTCTCATGTGCACTAGAAAC

ATTTTGTGGTCAATCATATGGAGCACAGAAGTACCATATGGTGGGAATACACTTGCAGAGAGCCATATTGATAAACATGC

TTCTTACTATACCTCAGTCCATTATTTTGGCAAACCTAAGACCTATTCTAATTTTTTTATATCAAGACCCAAATATAGCA

GCAGAAGCTGGATTCTATGGAAGATATTTGATCCCAAACGTTTTTGCAAATGCTATTCTTAGTTGTATAGTAAAATTTCT

TCAAACTCAGAACATAGTAGTTCCTATGTTGCTAGCTTCTGGAATTACTAGCTTGGTACATTTTCTTAACTGTTGGATTT

GGATTATAAAATTAAGGCATGGAATCAAAGGAGCTGCAATTGCAACTTGCATTTCAAATTGGCTTTACACAGTGCTACTA

GTACTTTACATCAAATTCTCATCTTCTTGCAGAAGTACTTGGACTGGTTTCTCAAGGGAGTCATTGAATAACATCCCTCA

GTTCCTCAGGATTGCTTTTCCTTCAGCAATCATGGTCTGCTTAGAATCATGGATGTATGAAATAATGGTGCTCCTATCCG

GTACTCTTCCTAATCCAAAATTGCAAACATCAGTACTTTCTTTATGCATGAATATAGGAGCAGTTGTGTGGATGATTTCC

CTTGGACTAAGTGGAGCTGCAAGCGTTCGTGTCTCAAATGAACTCGGAGCAGGTAATGCAAGGGCTGCACGTTTGGCAGT

GTGCGTTGTTGTGGTGATTGTTGTTACCGAAGGCATTTTAGTTGGAACAGTGATGATACTGTTACGCAATATTTGGGGGT

ATGCTTATACCAGTAAAGTTGAAGTGGTTAAACAAATAGAAATTATGTTTCCAATTCTTGCAGCAGGCAATCTCATTGAT

GCTCTACAAAGTGTTCTTGCAGGCATTGCTCGAGGATCTGGTTGGCAAAAAGCTGGTGCAGTTGTCTATTTGGGGTCATG

CTATTTAGTTGGGATTCCAGCAGCCATAATATTTGCTTTTGTACTGCATACTGGTGTTAAGGGGCTATTGTTTGGGATCA

TATGTGCACTCATTGCTCAAGCCTTTTCTCTGATGATCATTACATTACGCACTGATTGGGAGAAAGAGGCAAACAAGGCT

AAAGATAGAGTCTATAAATCTATAACACCAGAGAGTTTTCGGAATAGACATTCTTGA

>MsMATE46

ATGATAATTGGCTTATGCAGAAAGGATATTACAGAAGAAGTGAAGAAGCAGCTATGGCTTGCAGTGCCAATGATATTTGC

TAGTGTTTTTCAATATAGCTTACAAATGATATCTCTCATGTTTATTGGCCATATGAATGATGAGGTGCTTCTAGCTGGTG

CTGCTTTGGCAAATTCAGTTACGAGTGTTTTTGGTTATAGTGTATTGGTAGGTTTCTCATGTGCACTAGAAACATTTTGT

GGTCAATCATATGGAGCACAGAAGTACCATATGGTGGGAATACACTTGCAGAGAGCCATATTGATAAACATGCTTCTTAC

TATACCTCAGTCCATTATTTTGGCAAACCTAAGACCTATTCTAATTTTTTTATATCAAGACCCAAATATAGCAGCAGAAG

CTGGATTCTATGGAAGATATTTGATCCCAAACGTTTTTGCAAATGCTATTCTTAGTTGTATAGTAAAATTTCTTCAAACT

CAGAACATAGTAGTTCCTATGTTGCTAGCTTCTGGAATTACTAGCTTGGTACATTTTCTTAACTGTTGGATTTGGATTAT

AAAATTAAGGCATGGAATCAAAGGAGCTGCAATTGCAACTTGCATTTCAAATTGGCTTTACACAGTGCTACTAGTACTTT

ACATCAAATTCTCATCTTCTTGCAGAAGTACTTGGACTGGTTTCTCAAGGGAGTCATTGAATAACATCCCTCAGTTCCTC

AGGATTGCTTTTCCTTCAGCAATCATGGTCTGCTTAGAATCATGGATGTATGAAATAATGGTGCTCCTATCCGGTACTCT

TCCTAATCCAAAATTGCAAACATCAGTACTTTCTTTATGCATGAATATAGGAGCAGTTGTGTGGATGATTTCCCTTGGAC

TAAGTGGAGCTGCAAGCGTTCGTGTCTCAAATGAACTCGGAGCAGGTAATGCAAGGGCTGCACGTTTGGCAGTGTGCGTT

GTTGTGGTGATTGTTGTTACCGAAGGCATTTTAGTTGGAACAGTGATGATACTGTTACGCAATATTTGGGGGTATGCTTA

TACCAGTAAAGTTGAAGTGGTTAAACAAATAGAAATTATGTTTCCAATTCTTGCAGCAGGCAATCTCATTGATGCTCTAC

AAAGTGTTCTTGCAGGCATTGCTCGAGGATCTGGTTGGCAAAAAGCTGGTGCAGTTGTCTATTTGGGGTCATGCTATTTA

GTTGGGATTCCAGCAGCCATAATATTTGCTTTTGTACTGCATACTGGTGTTAAGGGGCTATTGTTTGGGATCATATGTGC

ACTCATTGCTCAAGCCTTTTCTCTGATGATCATTACATTACGCACTGATTGGGAGAAAGAGGCAAACAAGGCTAAAGATA

GAGTCTATAAATCTATAACACCAGAGAGCATAGTCATATGA

>MsMATE47

ATGATAATTGGCTTATGCAGAAAGGATATTACAGAAGAAGTGAAGAAGCAGCTATGGCTTGCAGTGCCAATGATATTTGC

TAGTGTTTTTCAATATAGCTTACAAATGATATCTCTCATGTTTATTGGCCATATGAATGATGAGGTGCTTCTAGCTGGTG

CTGCTTTGGCAAATTCAGTTACGAGTGTTTTTGGTTATAGTGTATTGGTAGGTTTCTCATGTGCACTAGAAACATTTTGT

GGTCAATCATATGGAGCACAGAAGTACCATATGGTGGGAATACACTTGCAGAGAGCCATATTGATAAACATGCTTCTTAC

TATACCTCAGTCCATTATTTTGGCAAACCTAAGACCTATTCTAATTTTTTTATATCAAGACCCAAATATAGCAGCAGAAG

CTGGATTCTATGGAAGATATTTGATCCCAAACGTTTTTGCAAATGCTATTCTTAGTTGTATAGTAAAATTTCTTCAAACT

CAGAACATAGTAGTTCCTATGTTGCTAGCTTCTGGAATTACTAGCTTGGTACATTTTCTTAACTGTTGGATTTGGATTAT

AAAATTAAGGCATGGAATCAAAGGAGCTGCAATTGCAACTTGCATTTCAAATTGGCTTTACACAGTGCTACTAGTACTTT

ACATCAAATTCTCATCTTCTTGCAGAAGTACTTGGACTGGTTTCTCAAGGGAGTCATTGAATAACATCCCTCAGTTCCTC

AGGATTGCTTTTCCTTCAGCAATCATGGTCTGCTTAGAATCTTGGATGTATGAAATAATGGTTCTCCTCTCTGGTACCCT

TCCTAATCCAAAATTGCAAACTTCAGTGCTTGCTATATGCATGAATATAGCGTCAGTTGTGTGGATGCTTTCCTCTGGAT

TCACTGGTGCTGCAAGCATTCGTGTTTCAAATGAATTAGGTGCTGGTAATCCTAGAGCTGCACGTTTGGCCGTGTGTGTC

GTTGTGGTGCTTAATATTACTGAGGCAATCTTAGTTGGAACAGTGATGATATTGTTACGAAATATATGGGGCTATGCTTA

TACCAAAGACATTGAAGTTGTCAAACAAATAGCAATTATGTTACCAATTCTTGCAGTATCCTACTTTCTGGATTCACTAC

AAAGTGTTCTTGCAGGCATTGCTCGAGGATCTGGTTGGCAAAAAGCTGGTGCAGTTGTCTATTTGGGGTCATGCTATTTA

GTTGGGATTCCAGCAGCCATAATATTTGCTTTTGTACTGCATACTGGTGTTAAGGGGCTATTGTTTGGGATCATATGTGC

ACTCATTGCTCAAGCCTTTTCTCTGATGATCATTACATTACGCACTGATTGGGAGAAAGAGGCAAACAAGGCTAAAGATA

GAGTCTATAAATCTATAACACCAGAGAGTTTTCGGAATAGACATTCTTGA

>MsMATE48

ATGATAATTGGCTTATGCAGAAAGGATATTACAGAAGAAGTGAAGAAGCAGCTATGGCTTGCAGTGCCAATGATATTT

GCTAGTGTTTTTCAATATAGCTTACAAATGATATCTCTCATGTTTATTGGCCATTTAGATGATGAGGTGCTTCTAGCT

GGTGCTTCTTTGGCAAATTCATTTATGAACGTCATTGGTATAAGTATATTGTTCTTCATGGAAGCTACATATTCGGAA

AGGGGAAAAAGAAAATTTTCTCGTATCCTTGGGGCGAATCTAGACAAAATGGTTTTTTTTCTCTCGTCTAAATGTGTT

CTTGTAGACGTGATCGGTTTTGAAGAAGTTGTTTGGGGGTAA

>MsMATE49

ATGAATATCTTGTCCAGTACTTTGACAATGGAAACATCGAATCTTGAGACTCCATTGTGTTCAGACCAACATGAACTGGA

TGCCCAAACATTTGATCAAGGTTGTTGCATCAGAGAAGATGTTGTTGAGGAAGCTAAAAAGCAATTATGGCTAGCAGGGC

CACTCATTGCTGTTAGTTTGTTGCAATATAGTTTGCAAATGATATCTATTATGTTTGTTGGTCATCTTGGTAAACTTCCT

CTTTCAGGTGCTTCTTTGGGGAACTCCTTTGCCTCAGTCACTGGTTATAGTGTTTTGTTAGGAATGGGAAGTGCATTGGA

GACACTATGTGGCCAAGCCTATGGAGCTGAGCAGTATCACATGCTAGGAGTTCATACACAAAGGGCAATGCTAGTCCTTA

TAGCGTTGAGCATTCCCTTATCTTTAATTTGGTTTAACACGTGCAACCTTCTCATAGCATTAGGCCAAAACCATGAAATA

TCAACAGAAGCTGGAACATTTAACAGGTGGATGATCCCTGGCCTTTTTGCTTATGCTCTCGTTCAATGCCTTAACAGATT

TCTACAGACACAAAACAATGTTTTTCCAATGCTAATAAGCTCTGGAATCACAACTTTGGTACATCTTGTCTTTTGTTGGG

TTTTTGTGTTTGAATATGAATTAGGGATCAAAGGAGCAGCCTTGGCAATAAGCTTGTCCTATTGGGTTAATGTGTTCATG

TTGGTGATTTACATAAATTCAGCTACAGCTTGTGCATCAACTTGGACTGGAGTTTCCAAAGAAGCTTTGAATGATATTCT

TAGTTTTGTAAGGCTTGCGATGGCTTCAACACTTATGATATGTTTAGAATATTGGTCCTTTGAGATGGTTGTTCTTCTCT

CTGGCCTTCTACCAAACCCACAACTAGAGACATCTGTATTATCAATAAGTCTTAATACATGTTGGATGGTCTATATGATC

TCTGTTGGTCTTGGTGGTGCCATAAGCACACGGGTGTCAAATGAATTGGGTTGTGGGAATGCAAAAGGTGCACTTTTGGC

TCTCCGTGTCATGATAGTGATTGCCATAGTAGAGGGTACAGTAGTAGTATTGGTTACCATTTTGGTAAGGAATGTTTGGG

GAAAGCTTTATAGTAATGAAGATGAAGTTATCAAGTATGTTGCCAAGATGATGCCTCTTCTGGCACTGTCTGACTTCCTG

GATGGTTTCCAGTGTGTGCTTTCTGGAGCTGCTAGAGGTTGTGGTTGGCAAAATCTATGTGCATCTATAAACCTTGGTGC

TTACTATGTTGTGGGAATTCCTTCTGCTATCCTGTTTGCCTTTACCTTCCATATTGGAGGGATGGGGCTTTGGATGGGAA

TCATATGTGGCCTTTGTGTTCAAGGGATAGCTTTGGTTACAGTGAATGCACGCACAAATTGGGATAGAGATGTAACAAAC

ATATTTTATGCTTCAAAGTTCAACCATTTCAATAGGTTGAGGCTATGA

>MsMATE50

ATGAGAAGTGAAAGACAAGAGTTAGTGATAGCAGAATTGAAGAGACAACTATGGTTAGCAGTTCCTTTGTCCTCAGTTGG

AATCTTGCAATACATTCTACAAACAATTTCTATTGTGTTTGTTGGACATCTTGGTACCCTTCCTCTTTCTGGTGCTTCCA

TGGCCACTTCCTTTGCATCTGTTACTGGCTTCACATTACTGATGGGAATTACTAGTGCACTGGACACATTTTGTGGTCAA

TCAAATGGAGCAGAACAGTACCATATGCTTGGAATACATATGCAAAGAGCCATGATTGTGGTTTCAATTGTGAGTGTATT

CCTTGCCATCATATGGGCAAATACAAAACAAATTTTAGTTGTTATGCATCAGGACAAAGCCATATCCAAGGAAGCTGGTT

CATACGCATTTTTTCTGATCCCGAGTTTGTTCGCATATGGTCCTCTCCAATGCATTCTCAAGTTCTTGCAAACACAAAAC

ATTGTCCTCCCAATGGTGGTAACTTCTGGGATTGCAGCTTTGCTTCACACCCTTTTGTGTTGGCTTTTGGTTTTTGAATT

TAAGCTTGGAAGTAAAGGGGCAGCTATATCCAATTCTATATGTTATTGGGTCAATGTGTTGTTGATTTCACTCTATGTAA

AATTTTCTTCTACTTGTAAACAAACATGGACAGGATTTTCAAAGAGAGCTCTGCAGGATCTCTTTGTCTTCCTTAGACTT

GCTATACCTTCAGCTCTCATGGTCTGTTTGAAAGTGTGGACTTTTGAATTGATGGTTCTCATGTCTGGTCTTCTTCCTAA

TCCAGTAATAGAAACGTCAGTGCTTTCAATATGCCTTAATACATTTGGTCTAGCTTGGATGATCCCTTTTGGGTGCAGTT

GTGCTGTCAGCATACGGGTATCGAATGAACTGGGAGGTGGAAATCCAAATGGTGCAAGTCTAGCAGTTCGTGTGGCGTTA

TCGATATCCTTCATTGCAGCCTTATTTATGGTTTTAAGTATGATTCTAGCCAGGAAAGTGTGGGGGCATCTTTACAGTGA

TGACAAACAAGTCATCAGATATGTGTCAGCTATGATGCCAATTTTAGCTATCTCCAGTTTCTTAGATGCCATCCAAAGTA

CACTTTCAGGTGTTCTTGCAGGATGCGGGTGGCAGAAGATCGGTGCCTATGTGAATCTTGGTTCATTTTATGTTGTAGGT

GTACCATGTGCAGTTGTATTAGCCTTTTTTGTACACATGCATGTCATGGGACTGTGGTTAGGGATCATCTCTGCATTTAT

TGTGCAAACTTTACTTTACAGTATTTTTACCATTCGTTCTAACTGGGAGGAAGAAGCAACAAAAGCTCAAAGAAGAGTAG

AGCGCTCAACCACCACCCCAAATACTACCCTCAGAGACAGCATTTCACCATCTCAAAAACTTGAACAAATTCCCTAA

>MsMATE51

ATGTCTCTTGATTCTCCATTGATTGTTGAAGAAACAAAACAAACCAACAAGAAAGAAGAAGATAGAAAAGATTTGGTTGA

AGAAGTGAAGAAACAGTTATGGCTATCAGGGCCATTGATATCAGTTACACTTTTAAATTTTGGTATCAATCTTATTTCAG

TTATGTTTGTTGGTCATCTTGGTGAGTTACCTCTCTCTGGTGCTTCCATGGCTACTTCTTTTGCTTCTGTCACTGGTTTC

AGTTTATTGCAAGGAATGGCAAGTGCCTTGGACACATTATGTGGACAATCATACGGCGCGAAACAATACCGCATGTTAGG

CGTACACATGCAGAGAGCAATGTTCATTCTCATGATCGTTGCGGTTCCCCTCGCGATTATTTGGGCAAACACAAGATCAA

TTCTCATTTTCCTTGGCCAAGATCCTGAAATATCTACAGAAGCTGGCAACTATGCAAAGTTAATGGTTCCATGCCTTTTT

GCTTATGGTCTTCTACAATGCCTCAACAGATTCTTACAGACTCAAAATATTGTATTTCCAATGATGTTTTGCTCTGCGAT

GACAACGTTACTTCATCTTCCTATTTGTTGGTTTATGGTATACAAATCTGGATTAGGTAGCAGAGGGGCTGCCATAGCAA

ATTCTATATCCTATTGGATCAACGTTACCATACTCGCACTCTATGTCAAGTTTTCTCCTTCATGTAAAAAAACATGGACT

GGTTTTTCTAAAGAAGCATTTGCACTGAACAATATCCCAATTTTCCTGAAGCTTGCCATTCCTTCAGCTGTAATGGTTTG

CTTGGAAATGTGGTCATTTGAATTAATGGTTCTCCTTTCCGGTCTTCTTCCAAATCCAAAGTTGGAAACATCAGTGCTTT

CTATCAGTTTGAATACATCAGCACTTGTTTGGATGATCCCCTTTGGACTTAGCGGAGCTATAAGCATTCGTGTCTCGAAT

GAACTCGGGGCTGGTAATCCACGGGCTGCACGTTTGGCAGTGTATGTCGTTGTAGTGATAGCCGTTATCGAGAGCATCGT

AGTTGGAGCTGTGATTATATTAATTCGCAATATATGGGGATATGCTTATAGCAATGAAGAAGAAGTTGTCAAATATGTAG

CAATTATGTTGCCTATTATTGCAGTATCCAACTTTCTGGATGGACTACAATGTGTTCTTTCAGGCACTGCTAGAGGAGTT

GGTTGGCAAAAAATCGGTGCATATGTCAATCTCGGGTCATATTATTTAGTCGGGATTCCAGCAGCCGTTGTATTAGCTTT

CGTATTGCATGTTGGTGGGAAGGGACTGTGGCTGGGGATCATATGTGCACTCGTTGTTCAAGTGCTTTCTCTTACGATCA

TTACAATACGCACTGATTGGGAGAAAGAGGCAAAGAAGGCTAATGATAGAGTTTATGATTCAATAACAACAGAGAGCTTA

GTCTCATGA

>MsMATE52

ATGTCTCTTGATTCTCCATTGATTGTTGAAGAAACAAAACAAAACAACAAGAAAGAAGAAGATAGAAGAGAATTGGTTGA

AGAAGTGAAGAAACAGTTATGGCTATCAGGGCCATTGATATCAGTTACACTTTTAAATTTTGGTATCAATCTTATTTCAG

TTATGTTTGTTGGTCATCTTGGTGAGTTACCTCTCTCTGGTGCTTCCATGGCTACTTCTTTTGCTTCTGTCACTGGTTTC

AGTTTATTGCAAGGAATGGCAAGTGCCTTGGACACATTATGTGGACAATCATACGGCGCGAAACAATACCGCATGTTAGG

CGTACACATGCAGAGAGCAATGTTCATTCTCATGGTCGTTGCGATTCCCCTCGCGGTTATTTGGGCAAACACAAGATCAA

TTCTCATTTTCCTTGGCCAAGATCCTGAAATATCTATAGAGGCTGGCAACTATGCAAAGTTAATGGTTCCATGCCTTTTT

GCTTATGGTCTTCTACAATGCCTCAACAGATTCTTACAGACTCAAAATATTGTATTTCCAATGATGTTTTGCTCTGCGAT

GACAACGTTACTTCATCTTCCTATTTGTTGGTTTATGGTATACAAATCTGGATTAGGTAGCAGAGGGGCCGCCATAGCAA

ATTCTATATCCTATTGGATCAACGTTACCATACTCGCACTCTATGTCAAGCTTTCTCCTTCATGTAAAAAAACATGGAAT

GGTTTTTCCAAAGAAGCACTTGCACTGAAAAATATCCCAATTTTCCTGAAGCTTGCCATTCCTTCAGCTATAATGGTTTG

CTTGGAAATGTGGTCATTTGAATTAATGGTTCTCCTTTCCGGTCTTCTTCCAAATCCAAAGTTGGAAACATCAGTTCTTT

CTATCAGTTTGAATACATCAGCACTTGTTTGGATGATCCCCTTTGGACTTAGTGGAGCTATAAGCATTCGTGTCTCGAAT

GAACTCGGGGCTGGTAATCCAGAAGCCGCACGTTTGGCAGTGTATGTCGTTGTAGTGATAGCCATTATTGAGAGCATCGT

AGTTGGAGCTGTGATTATATTAATTCGCAATATATGGGGATATGCTTATAGCAATGAAGAAGAAGTTGTCAAATATGTAG

CAATTATGTTGCCTATTATTGCAGTATCCAACTTTCTGGATGGACTACAATGTGTTCTTTCAGGCACTGCTAGAGGAGTT

GGTTGGCAAAAAATCGGCGCATAG

>MsMATE53

ATGGAAGCTCATGAGGGGTCAATATTACAACTCACTTATTATTATTTTGACGTTATGTTTGTTGGTCATCTTGGTGAGTT

ACCTCTCTCTGGTGCTTCCATGGCTACTTCTTTTGCTTCTGTTACTGGTTTCAGTTTATTGCAAGGAATGGCAAGTGCCT

TGGACACATTATGTGGACAATCATATGGCGCGAAACAATACCGCATGTTAGGCGTACACATGCAAAGAGCAATGTTCATT

CTGATGATCGTTGCGGCTCCCCTCGCGATTATTTGGGCAAACACAAGATCCATTCTCATTTTCCTTGGCCAAGATTCTGA

AATATCTACAGAAGCTGGCAGCTTTGCAAAGTTAATGGTTCCCTGCCTTTTTGCTTACGGTCTTCTACAATGTCTTAACA

GATTCTTACAGACTCAAAATATTGTATTTCCAATGATGTTTAGCTCCGCGATTACGACATTACTTCATATTCCTCTATGT

TGGATAATGGTATACAAGTCTGGATTAGGTAGCGGAGGAGCTGCCATAGCAAGTTCTATATCCTATTGGATCAACGTTAC

CATACTCGCACTCTATGTCAAGTTTTCTCCTTCATGTAAAAAAACATGGACTGGTTTTTCTAAAGAAGCATTTGCACTGA

ACAATATCCCAATTTTCCTGAAGCTTGCCATTCCTTCAGCTGTAATGGTTTGCTTGGAAATGTGGTCATTTGAATTAATG

GTTCTCCTTTCCGGTCTTCTTCCAAATCCAAAGTTGGAAACATCAGTGCTTTCTATCAGTTTGAATACATCAGCACTTGT

TTGGATGATCCCCTTTGGACTTAGCGGAGCTATAAGCATTCGTGTCTCGAATGAACTCGGGGCTGGTAATCCACGGGCTG

CACGTTTGGCAGTGTATGTCGTTGTAGTGATAGCCGTTATCGAGAGCATCGTAGTTGGAGCTGTGATTATACTAATTCGC

AATATATGGGGATATGCTTATAGCAATGAAGAAGAAGTTGTCAAATATGTAGCAATTATGTTGCCTATTATTGCAGTATC

CAACTTTCTGGATGGACTACAATGTGTTCTTTCAGGCACTGCTAGAGGAGTTGGTTGGCAAAAAATCGGTGCATATGTCA

ATCTCGGGTCATATTTTTTAGTCGGGATTCCAGCTGCCGTTGTATTAGCTTTCGTATTGCATGTTGGCGGGAAGGGACTG

TGGCTGGGGATCATATGTGCACTCGTTGTTCAAGTGCTTTCTCTTACGATCATTACAATACGCACTGATTGGGAGAAAGA

GGCAAAGAAGGCTAATGATAGAGTTTATGATTCAATAACAACAGAGAGCTTAGTCTCATGA

>MsMATE54

ATGTCTCTTGATTCTCCATTGATTGTTGAAGAAACAAAACAAAACAACAAGAAAGAAGAAGATAGAAGGGATTTGGTTGA

AGAAGTTAAGAAACAGTTATGGCTATCAGGGCCATTGATAACAGTTACACTTTTAAATTTTGGTATCAATCTTATTTCAG

TTATGTTTGTTGGTCATCTTGGTGAGTTACCTCTCTCTGGTGCTTCCATGGCTACTTCTTTTGCTTCTGTTACTGGTTTC

AGTTTATTGCAAGGAATGGCAAGTGCCTTGGACACATTATGTGGACAATCATATGGCGCGAAACAATACCGCATGTTAGG

CGTACACATGCAAAGAGCAATGTTCATTCTGATGATCGTTGCGGCTCCCCTCGCGATTATTTGGGCAAACACAAGATCCA

TTCTCATTTTCCTTGGCCAAGATTCTGAAATATCTACAGAAGCTGGCAGCTTTGCAAAGTTAATGGTTCCCTGCCTTTTT

GCTTACGGTCTTCTACAATGTCTTAACAGATTCTTACAGACTCAAAATATTGTATTTCCAATGATGTTTAGCTCCGCGAT

TACGACATTACTTCATATTCCTCTATGTTGGATAATGGTATACAAGTCTGGATTAGGTAGCGGAGGAGCTGCCATAGCAA

GTTCTATATCCTATTGGATCAACGTTACCATACTCGCACTCTATGTCAAGTTTTCTCCTTCATGTAAAAAAACATGGACT

GGTTTTTCTAAAGAAGCATTTGCACTGAACAATATCCCAATTTTCCTGAAGCTTGCCATTCCTTCAGCTGTAATGGTTTG

CTTGGAAATGTGGTCATTTGAATTAATGGTTCTCCTTTCCGGTCTTCTTCCAAATCCAAAGTTGGAAACATCAGTGCTTT

CTATCAGTTTGAATACATCAGCACTTGTTTGGATGATCCCCTTTGGACTTAGCGGAGCTATAAGCATTCGTGTCTCGAAT

GAACTCGGGGCTGGTAATCCACGGGCTGCACGTTTGGCAGTGTATGTCGTTGTAGTGATAGCCGTTATCGAGAGCATCGT

AGTTGGAGCTGTGATTATACTAATTCGCAATATATGGGGATATGCTTATAGCAATGAAGAAGAAGTTGTCAAATATGTAG

CAATTATGTTGCCTATTATTGCAGTATCCAACTTTCTGGATGGACTACAATGTGTTCTTTCAGGCACTGCTAGAGGAGTT

GGTTGGCAAAAAATCGGTGCATATGTCAATCTCGGGTCATATTTTTTAGTCGGGATTCCAGCTGCCGTTGTATTAGCTTT

CGTATTGCATGTTGGCGGGAAGGGACTGTGGCTGGGGATCATATGTGCACTCGTAGTTCAAGTACTTTCTCTAACGATCA

TTACAATACGCACTGATTGGGAGAAAGAGGCAAAGAAAGCTAATGATAGAGTTTATGATTCAATAACAACAGAGAGCTTA

GTCTCATGA

>MsMATE55

ATGGAAACACCATTGCTGACCAAAAGATTTACTTCAGAAAATGATTACTTGGAAGTGAAAAGTTTGAAGGATGTTAAACA

TGTTTTATGGAGTGAGGCAATGAAGATATGGAAGATAGCACTTCCAGTGGCTTTGTGTTCATTGTTTCAGTACCTCACAA

ACACTTCAACTTCCATCTATGCTGGTCATCTTGGAGATATTGAACTCTCTTCTTTCTCTCTTTATCAAAGTATCCTCAAT

TGCATCTATTCTTTGCTGTTTGGTATGTCAACTGCACTAGCAACACTTTGTGGCCAAGCTTATGGTGCAAGACAATTTCA

AAGTGCTGGTATTTATCTTCAAAGGTCATGGATAGTACTCTTCACCACTTGTATATTCCTCTTGCCAATTCATCTATATG

CAACTCTAATCTTAAAACTTCTTGGTCAAGAAAAAGAGATAGCCGATCTTGCCGGAAAATATGCTATTCTACTTATTCCC

TACATGTTTTCCTTCGCGGTCAATTTGCCGCTCGTGAAGTTTCTTCAAGCACAAAGCAAAGTTAATGTTATTATGTACAT

AGCAATGGTTACATTACTCATTCAAAATGTTCTACTTTACATCTTCATCAGTGTATTTGATTGGGGAATGATTGGTTTAG

CAATGGCAAGTAATATCTCAGGATGGATATTTTCTATTGCATTGGTAATTTATGTCATTGGTTGGTCCAAAGAAGGATGG

AATGGATTGTCTTGGATGGCATTTAGGGAATTATGGGAATTTACTAAGCTAAGTTTTGGTTCATCTGTAATGATTTGTTT

AGAACAGTGGTATACTACAATCATTATCCTTCTTGCTGGCTACCTTGATAATCCTGTGATTGCTCTTGGTTCCTATTCAA

TTTGTCTTAATGTTCAGGGTTGGAACTCCATGCTGCTTCTTGGAGTAAGTACAGCATTAAGTGTTCGTGTCTCTCATACA

CTTGGAATGTCGCATCCAAGAGCATCCAAATACTCTTTCTTGGTGGCAATGTCTCAGTCTCTTCTCATTGGAATCATTTT

CATGACTGTTATTTTCTTGAGTAAAGAAAAGTTTGCCTTCATCTTTACCAACAGTGATGATATGGTACATGCTGCTAGTG

AATTAGCATACCTTCTTGGTATAACAATGGTTATCAACAGTATTTCACAAACTATATCAGGTGTGGTCATTGGATGTGGG

TGGCAAGTTATGGTTGGTTACATAAATTTGGCATGCTATTACATTGTTGGACTCCCTATTGGAATTTACCTTGGATTCAA

CCAGCATTTAGGGGTCAAGGGTCTATGGGGAGGAACAATGTGCGGCAATATTCTTCAGATTTTGGTCCTCGTAGTCATTA

TATACAAGACCAACTGGACCAATGAGGTAGAGCAAACAGCTAATCGCATGAGAATTTGGAGCTCTAACAAAGTTCAAAAG

GACATCATTTGA

>MsMATE56

ATGGAAACACCATTGGTGAATAAAAGCTTTACTTCAGAAAATGATTACTTACCTTTGAAAAACTTGAAAGATGTGAAATA

TGTTTTGTGGAATGAGACATTGAAGATATGGAAGATAGCAATTCCAGTGGCTTTGTCTTTACTTTTTCAGAACCTCATTG

GCTCTTCAAATTCCATCTATGCTGGTCATATTGGAGATATTCAGCTCTCTTCTTACTCTTTGTATCAAAGTGTCATCACT

ACTATCTATTTCTCTATATTGTATGGTATGTCAAATGCACTAGCAACACTATGTGGTCAAGCTTATGGTGCAGGAAAATT

TCAAAATGCTGGTATTTATCTTCAAAGGTCATGGATAGTACTCTTCACCACTTGTATATTCCTCTTGCCAATTCATCTAT

ATGCAACTCTAATCTTAAAACTTCTTGGTCAAGAAAAAGAGATAGCCGATCTTGCCGGAAAATATGCCATTCTACTAATT

CCCTACATGTTTTCCTTCGCCGTCAATTTGCCTCTCGTGAAGTTTCTTCAAGCACAAAGCAAAGTTAATGTTATTATGTA

CATATCAATGGTTACATTACTCATCCAAAATGTTCAACTTTACATCTTCATCAGTGTATTTGATTGGGGAATAACTGGCT

TAGCTATGGCAAGTAATATCACAGGATGGGGATTTGCTATTGCTCAACTAATTTATGCCATTGGTTGGTGTAAAGAAGGA

TGGAATGGATTGTCTTGGATGGCATTTAGGGAATTATGGGAATTTACTAAGCTAAGTTTGGGTTCATCTGTAATGATTTG

TTTAGAACAGTGGTATACTACAATCATTATCCTTCTTGCTGGCTACCTTGATAATCCTGTGATTGCTCTTGGTTCCTATT

CAATTTGCCTTAATATTCAGGGTTGGAACGCCATGCTGCTTCTTGGACTAAGTACAGCACTAAGTGTTCGTGTCTCTAAT

ACACTTGGAATGTCACATCCAAGAGCATCTAAGTACTCTTTCTTGGTGGCAATGTCTCAGTCTCTTCTCATTGGAACCAT

CTTCATGACTGTTATTTTCTTGAGTAAAGAAAAGTTTGCCCTCATCTTTACCAAAAGTGATGATTTGATACATGCTGCTA

GTGAATTAGCATACCTTCTTGGTATAACAATGGTTATCAACAGTATTTCACAAACTATATCAGGTGTGGTAATTGGATGT

GGATGGCAAGTTATGGTTGGTTACATAAACTTGGCATGCTATTACATTGTTGGACTCCCTATTGGAATTTTCCTTGGATT

CAACCAGCATTTGGGGGTTAAGGGTCTTTGGGGAGGCACAATGTGCGGCAATATTCTTCAGATTTTAGTCCTCGTAGTCA

TTATATACAAGACCAACTGGACCAATGAGGTAGAGCAAACAGCTAATCGCATGAGAATTTGGAGCTCTAACAAAGTTCAA

AAGGACATCATTTGA

>MsMATE57

ATGGAAACACCATTGCTGACCAAAAGATTTACTTCAGAAAATGATTACTTGGAAGTGAAAACTTTGAAGGATGTTAAACA

TGTTTTGTGGAGTGAGACAGGGAAGATATGGAAGATAGCACTTCCAGTGGCTTTGTGTTCATTGTTTCAGTACCTCACAA

ACACTTCAACTTCCATCTATGCTGGTCATCTTGGAGATATTCAGCTCTCTTCTTACTCTTTGTATCAAAGTGTCATCACT

ACTATCTATTTCTCTATATTGTATGGTATGTCAAATGCACTAGCAACACTATGTGGTCAAGCTTATGGTGCAGGAAAATT

TCAAAATGCTGGTATTTATCTTCAAAGGTCATGGATAGTACTCTTCACCACTTGTATACTCCTCTTGCCTATTAATATAT

ATGCAACTCCAATCTTAAAATTTCTTGGCCAAGAACAAGAAATAGCTGGCCTTGCTGGCAAATATGCTATTCTTATAACT

CCATACATGTTTTCCATTGCTATCAATTTACCCATCCAGAAATTTCTTCAAGCACAAAGTGAAGTTAAAGTTATTATGTA

CATAGCAATTGTGATATTGCTAGTACAAAATGGTCTACTTTATATCTTCATCAGTGTATTTGATTGGGGAATGATTGGTT

TAGCAATGGCAAGTAATATCTCAGGATGGATATTTTCTATTGCATTGGTAATTTATGTCATTGGTTGGTCTAAAGAAGGA

TGGAATGGATTGTCTTGGATGGCATTTAGGGAATTATGGGAATTTACTAAGCTAAGTTTTGGTTCATCTGTAATGATTTG

TTTAGAACAATGGTATACTGCTTGCATTATCCTTCTTGCTGGTCATCTTGATAATCCTGTCATTGCTGTTGGTTCCTTTT

CAATTTGCCTTAATATTCAGGGTTGGAACTCCATGCTGCTTCTTGGAGTAAGTACAGCACTAAGTGTTCGTGTCTCTCAT

ACACTTGGCATGTCACATCCAAGAGCATCTAAGTACTCTTTCTTGGTGGCAATGTCTCAGTCTCTTCTCATTGGAATCAT

TTTCATGACTGTTATTTTCTTGAGTAAAGAAAAGTTTGCGATCATCTTTACCAACAGTGATGATTTGATACATGCTGCTA

GTGAATTAGCATACTTTCTTGGTATAACCATGGTTCTCAACAGTATCTCACAAACTATATCAGGTGTGGTAATTGGATGT

GGATGGCAAGTTATGGTTGGTTACATAAATTTGGCATGCTATTACATTGTTGGACTCCCTATTGGAATTTTCCTTGGATT

CAACCAGCATTTAGGGGTCAAGGGGCTATGGGGAGGAACAATGTGCGGCAATATTCTTCAGATTTTAGTGCTCATAGTCA

TTATTTATAAGACCAACTGGACCAAAGAGGTAGAGCAAACGGCTAATCGCATGAGAATTTGGAGCTCTAACAACCTTCAA

AACGATGTGATTTGA

>MsMATE58

ATGGAAACACCATTGCTGACCAAAAGATTTACTTCAGAAAATGATTACTTGGAAGTGAAAACTTTGAAGGATGTTAAACA

TGTTTTATGGAGTGAGACAAGGAAGATATGGAAGATAGCACTTCCAGTGGCTTTGTGTTCATTGTTTCAGTACCTCACAA

ACACTTCAACTTCCATCTATGCTGGTCATCTTGGAGACATTGAACTCTCTTCTTTCTCTCTTTATCAAAGTATCATCAAT

TGCATCTATTCTTTGCTGTTTGGTATGTCAACTGCACTAGCAACACTTTGTGGCCAAGCTTATGGTGCAAGACAATTTCA

AAGTGCTGGTATTTATCTTCAAAGGTCATGGATAATACTCTTCACCACTTGCATACTACTCTTGCCAATTAATATATATG

CAACCCCAATCTTAAAATTTCTTGGCCAAGAACAAGAAATAGCTGACCTAGCTGGCAAATATGCAATTCAAATAACTCCA

TACATGTTTTCCATTGCTATCAATTTACCCATCAAGAAATTTCTTCAAGCACAAAGCAAAGTTAAAGTTATTATGTACAT

AGCAATAGTGATATTACTAATACAAAATGGTCTACTTTACATCTTCATCAGTGTATTTGATTGGGGAATAACTGGCTTAG

CAATGGCAAGTAATATCACAGGATGGAGATTTGCTATTGCACAACTAATTTATGCCATTGGTTGGTCCAAAGAAGGATGG

AATGGATTGTCTTGGATGGCATTTAGGGAATTATGGGAATTTACTAAGCTAAGTTTTGGTTCATCTGTAATGATTTGTTT

AGAACAGTGGTATACTACAATCATTATCCTTCTTGCTGGCTACCTTGATAATCCTGTGATTGCTCTTGGTTCATATTCAA

TTTGTCTTAATGTTCAGGGTTGGAACTCCATGCTGCTTCTTGGAGTAAGTACAGCATTAAGTGTTCGTGTCTCTAATACA

CTTGGAATGTCACATCCAAGAGCATCCAAATACTCTTTCTTGGTGGCAATGTCTCAGTCTCTTCTCATTGGAATCATTTT

CATGACTGTTATTTTCTTGAGTAAACAAAAATTTGCCATCATCTTTACCAAAAGTGAGGATATGATACATGCTGCTAGTG

AATTAGCATACCTTCTTGGTATAACAATGGTTATCAACAGTATTTCACAAACTATATCAGGTGTGGTCATTGGATGTGGA

TGGCAAGTTATGGTTGGTTACATAAACTTGGCATGCTATTACATTGTTGGACTCCCTATTGGAATTTACCTTGGATTCAA

CCAGCATTTAGGGGTCAAGGGTCTTTGGGGAGGCACGATATGCGCAAGTATTCTTCAGATTATAGTCCTCACAATCATTA

TTTGTAAGACCAACTGGACCAAGGAGGTAGAGCAAACAGCAAATCGCATGCAAATCTGGATCTCTAACAAGTAA

>MsMATE59

ATGGAAACACCATTGCTGACCAAAAGATTTACTTCAGAAAATGATTACTTGGAAGTGAAAACTTTGAAGGATGTTAAACA

TGTTTTGTGGAGTGAGACAGGGAAGATATGGAAGATAGCACTTCCAGTGGCTTTGTGTTCATTGTTTCAGTACCTCACAA

ACACTTCAACTTCCATCTATGCTGGTCATCTTGGAGACATTGAGCTCTCTTCTTTCTCTCTTTATCAAAGTATCATCAAT

TGCATCTATTCTTTGCTGTTTGGTATGTCAACTGCACTAGCAACACTGTGTGGCCAAGCTTATGGTGCAGGACAATTTCA

AAATGCTGGTATTTATCTTCAAAGGTCATGGATAGTACTCTTCACCACTTGTATACTCCTCTTGCCTATTAATATATATG

CAACTCCAATCTTAAAATTTCTTGGCCAAGAACAAGAAATAGCTGGCCTTGCTGGCAAATATGCTATTCTTATAACTCCA

TACATGTTTTCCATTGCTATCAATTTACCCATCCAGAAATTTCTTCAAGCACAAAGTGAAGTTAAAGTTATTATGTACAT

AGCAATTGTGATATTGCTAGTACAAAATGGTCTACTTTATATCTTCATCAGTGTATTTGATTGGGGAATAACTGGCTTAG

CTATGGCAAGTAATATCACAGGATGGGGATTTGCTATTGCTCAACTAATTTATGCCATTGGTTGGTGTAAAGAAGGATGG

AATGGATTGTCTTGGATGGCATTTAGGGAATTATGGGAATTTACTAAGCTAAGTTTTGGTTCATCTGTAATGATTTGTTT

AGAACAGTGGTATACTACAATCATTATCCTTCTTGCTGGCTACCTTGATAATCCTGTGATTGCTCTTGGTTCATATTCAA

TTTGCCTTAATATTCAGGGTTGGAACGCCATGCTGCTTCTTGGACTAAGTACAGCACTAAGTGTTCGTGTCTCTAATACA

CTTGGAATGTCACATCCAAGAGCATCCAAATACTCTTTCTTGGTGGCAATGTCTCAGTCTCTTCTCATTGGAACCATTTT

CATGACTGTTATTTTCTTGAGTAAACAAAAATTTGCCATCATCTTTACCAAAAGTGAGGATATGATACATGCTGCAAGTG

AATTAGCATACTTTCTTGGTATAACCATGGTTCTCAACAGTGTTTCACAAACTATATCAGGTGTGGTCATTGGATGTGGA

TGGCAAGTTATGGTTGGTTACATAAACTTGGCATGCTATTACATTGTTGGACTCCCTATTGGAATTTTCCTTGGATTCAA

CCAGCATTTGGGGGTGAAGGGTCTTTGGGGAGGCACAATGTGCGGCAATATTCTTCAGATTTTAGTTCTCGTAGTCATTA

TATACAAGACCAACTGGACCAATGAGGTAGAGCAAACAGCTAATCGCATGAGAATTTGGAGCTCTAACAAAGTTCAAAAG

GACATCATTTGA

>MsMATE60

ATGGAAACACCATTGCTGACCAAAAGATTTACTTCAGAAAATGATTACTTGGAAGTGAAAACTTTGAAGGATGTTAAACA

TGTTTTGTGGAGTGAGGCAATGAAGATATGGAAGATAGCACTTCCAGTGGCTTTGTGTTCATTGTTTCAGTACCTCACAA

ACACTTCAACTTCCATCTATGCTGGTCATCTTGGAGATATTGAGCTCTCTTCTTTCTCTCTTTATCAAAGTATCCTCAAT

TGCATCTATTCTTTGCTGTTTGGTATGTCAACTGCAATAGCAACACTGTGTGGCCAAGCTTATGGTGCAGGACAATTTCA

AAATGCTGGTATTTATGTTCAAAGATCATGTATTATACTCTTCACCACTTGTATACTCCTCTTGCCTATTAATATATATG

CAACTCCAATCTTAAAATTTCTTGGCCAAGAACAAGAAATAGCTGACCTAGCTGGCAAATATGCTATTCTTATAATTCCA

AGCATGTTTTCCATTGCTTTCAATTTACCCATCCAGAAATTTCTTCAAGCACAAAGTGAAGTTAAAGTTATTATGTACAT

AGCAATTGTGATATTGCTAATACAAAATGGTCTACTTTATATCTTCATCAGTGTATTTGATTGGGGAATAACTGGCTTAG

CTATGGCAAGTAATATCACAGGATGGGGATTTGCTATTGCTCAACTAATTTATGCCATTGGTTGGTGTAAAGAAGGATGG

AATGGATTGTCTTGGATGGCATTTAGGGAATTATGGGAGTTTACTAAGCTAAGTTTTGGTTCATGTGTAATGATCTGTTT

AGAACAGTGGTATACTACAATCATTATCCTTCTTGCTGGCTACCTTGATAATCCTGTGATTGCTCTTGGTTCCTATTCAA

TTTGCCTTAATATTCAGGGTTGGAACGCCATGCTGCTTCTTGGACTAAGTACAGCACTAAGTGTTCGTGTCTCTAATACA

CTTGGAATGTCACATCCAAGAGCATCCAAATACTCTTTCTTGGTGGCAATGTCTCAGTCTCTTCTCATTGGAACCATCTT

CATGACTGTTATTTTCTTGAGTAAAGAAAAGTTTGCCTTCATCTTTACCAACAGTGATGATTTGATACATGCTGCAAGTG

AATTAGCATACCTTCTTGGTATAACAATGGTTATCAATAGTATCTCACAAACTATATCAGGCGTGGTAATTGGATGTGGA

TGGCAAGTTATGGTTGGTTACATAAACTTGGCATGTTATTATATTGTTGGACTCCCTATTGGAATTTTCCTTGGATTCAA

CCAGCATTTGGGGGTTAAGGGTCTTTGGGGAGGCACGATATGCGCAAGTATTCTTCAGATTATAGTCCTCACAATCATTA

TTTGTAAGACCAACTGGACCAAGGAGGTAGAGCAAACAGCAAATCGCATGCAAATCTGGATCTCTAACAAGTAA

>MsMATE61

ATGGAAACACCATTGCTGACCAAAAGATTTACTTCAGAAAATGATTACTTGGAAGTGAAAACTTTGAAGGATGTTAAACA

TGTTTTGTGGAGTGAGACAGGGAAGATATGGAAGATAGCACTTCCAGTGGCTTTGTGTTCATTGTTTCAGTACCTCACAA

ACACTTCAACTTCCATCTATGCTGGTCATCTTGGAGATATTGAACTCTCTTCTTTCTCTCTTTATCAAAGTATCCTCAAT

TGCATCTATTCTTTGCTGTTTGGTATGTCAACTGCAATAGCAACACTGTGTGGCCAAGCTTATGGTGCAGGACAATTTCA

AAATGCTGGTATTTATGTTCAAAGATCATGTATTATACTTTTCACCACTTGTATACTCCTCTTGCCTATTAATATATATG

CAACTCCAATCTTAAAATTTCTTGGCCAAGAACAAGAAATAGCTGACCTAGCTGGCAAATATGCAATTCTTATAACTCCA

TACATGTTTTCCATTGCTATCAATTTTCCCATCCAGAAATTTCTTCAAGCACAAAGTGAAGTTAAAGTTATTATGTACAT

AGCAATTGTGATATTGCTAATACAAAATGGTCTACTTTATATCTTCATCAGTGTATTTGATTGGGGAATAACTGGCTTAG

CTATGGCAAGTAATATCACAGGATGGGGATTTGCTATTGCTCAACTAATTTATGCCATTGGTTGGTGTAAAGAAGGATGG

AATGGATTGTCTTGGATGGCATTTAGGGAATTATGGGAATTTACTAAGCTAAGTTTTGGTTCATCTGTAATGATTTGTTT

AGAACAGTGGTATACTACAATCATTATCCTTCTTGCTGGCTACCTTGATAATCCTGTGATTGCTCTTGGTTCCTATTCAA

TTTGCCTTAATATTCAGGGTTGGAACGCCATGCTGCTTCTTGGACTAAGTACAGCACTAAGTGTTCGTGTCTCTAATACA

CTTGGAATGTCACATCCAAGAGCATCCAAATACTCTTTCTTGGTGGCAATGTCTCAGTCTCTTCTCATTGGAACCATCTT

CATGACTGTTATTTTCTTGAGTAAAGAAAAGTTTGCCTTCATCTTTACCAACAGTGATGATTTGATACATGCTGCAAGTG

AATTAGCATACCTTCTTGGTATAACAATGGTTATCAACAGTATTTCACAAACTATATCAGGTGTGGTAATTGGATGTGGA

TGGCAAGTTATGGTTGGTTACATAAACTTGGCATGCTATTACATTGTTGGACTCCCTATTGGAATTTTCCTTGGATTCAA

CCAGCATTTGGGGGTTAAGGGTCTTTGGGGAGGCACAATGTGCGGCAATATTCTTCAGATTTTAGTCCTCGTAGTCATTA

TATACAAGACCAACTGGACCAATGAGGTAGAGCAAACAGCTAATCGCATGAGAATTTGGAGCTCTAACAACCTTCAAAAC

GATGTGATTTGA

>MsMATE62

ATGGAAACACCATTGCTGACCAAAAGATTTACTTCAGAAAATGATTACTTGGAAGTGAAAACTTTGAAGGATGTTAAA

CATGTTTTGTGGAGTGAGACAGGGAAGATATGGAAGATAGCACTTCCAGTGGCTTTGTGTTCATTGTTTCAGTACCTC

ACAAACACTTCAACTTCCATCTATGCTGGTCATCTTGGAGATATTGAACTCTCTTCTTTCTCTCTTTATCAAAGTATC

CTCAATTGCATCTATTCTTTGCTGTTTGGTATGTCAACTGCAATAGCAACACTGTGTGGCCAAGCTTATGGTGCAGGG

CAATTTCAAAATGCTGGTATTTATGTTCAAAGATCATGTATTATACTCTTCACCACTTGTATACTCCTCTTGCCTATT

AATATATATGCAACTCCAATCTTAAAATTTCTTGGCCAAGAACAAGAAATAGCTGGCCTTGCTGGCAAATATGCTATT

CTTATAACTCCATACATGTTTTCCATTGCTATCAATTTACCCATCCAGAAATTTCTTCAAGCACAAAGTGAAGTTAAA

GTTATTATGTACATAGCAATTGTGATATTGCTAGTACAAAATGGTCTACTTTATATCTTCATCAGTGTATTTGATTGG

GGAATAACTGGCTTAGCTATGGCAAGTAATATCACAGGATGGGGATTTGCTATTGCTCAACTAATTTATGCCATTGGT

TGGTGTAAAGAAGGATGGAATGGATTGTCTTGGATGGCATTTAGGGAATTATGGGAATTTACTAAGCTAAGTTTTGGT

TCATCTGTAATGATTTGTTTAGAACAGTGGTATACTACAATCATTATCCTTCTTGCTGGCTACCTTGATAATCCTGTG

ATTGCTCTTGGTTCCTATTCAATTTGCCTTAATATTCAGGGTTGGAACGCCATGCTGCTTCTTGGACTAAGTACAGCA

CTAAGTGTTCGTGTCTCTAATACACTTGGAATGTCACATCCAAGAGCATCCAAGTACTCTTTCTTGGTGGCAATGTCT

CAGTCTCTTCTCATTGGAACCATTTTCATGACTGTTATTTTCTTGAGTAAAGAAAAGTTTGCCTTCATCTTTACCAAC

AGTGATGATTTGATACATGCTGCAAGTGAATTAGCATACCTTCTTGGTATAACCATGGTTATCAACAGTATCTCACAA

ACTATTTCAGGTGATTTACTTGGTTTGGTGAGTAGAGAATTTGTCATAATTTCACTTTTGGTCTCTATTTTATCAATC

TCATTAAAATAA

>MsMATE63

ATGGAGGAAAATGACACACAAACCTTCCCACTCCTCACACCATTAAACAACCAACAACATGATCAAATCAACACAGCAGT

ATTCACAGCTAAATCCGATGATATTTCTCCGATCATCGGTGCCGGAGACTTTGCCAGAGAATTTCTCAATGAATCTAAGA

AACTTTGGTACCTTGCTGGACCTGCCATATTCACCTCCATCTCTCAATATTCTCTTGGTGCTGTTACTCAAGTCTTTGCT

GGTCAAGTTGGGACTCTTCAACTTGCGGCTGTTTCCGTCGAAAACTCCGTCATCGCCGGCTTTTGCTTAGGCATCACGAT

GGGGATGGGAAGCGCATTGGAGACACTATGTGGACAAGCTTTTGGAGCCGGAAAACTTAATATGTTAGGAATATACATGC

AAAGATCATGGTTGATTCTCAGTGCCACCGCGCTTATTCTTAGTTTTCTCTACATTTTCGCGTCGCCTCTTCTAAAACTT

ATAGGCCAAACAACCGCGATATCAGAGGCAGCCGGAGTTTTTGCACTATGGATGATTCCTCAACTCTTCGCTTACGCGGT

GAACTTTCCAACCCAAAAGTTCTTGCAAGCCCAGAGTAAGATCATGGCTATGGCATGGATTTCTGCGGTGGCGTTGGTGG

GACACACATTTTTTAGTTGGTTCCTAATGTTGCACCTAGGGTGGGGACTTGTTGGTGCAGCCGTGGTGCTTAATTCTTCG

TGGTGGTTTATTGTTTTGGCTCAAATTGTTTATGTTTTGAGTGGTTCTTGTGGTGAAGCTTGGAGTGGTTTTTCTTTTCA

AGCCTTTCAGAATCTTTGGGGTTTTGTTCGTCTCTCACTAGCTTCTGCTGTTATGATGTGCCTTGAAGTATGGTATTTTA

TGGCATTAATACTATTTGCTGGATATCTGGAGAATGCAGAAGTTTCAGTTGATGCATTGTCTATATGCGCAAACATATTG

GGGTGGACTGTCATGGCATCCTTTGGAATTAATGCAGCCATAAGTGTGAGGGTATCAAATGAATTGGGAGCATCTCATCC

AAGAGCAGCAAAATTCTCACTAGTGGTTGCAGTGATTACATCATTTGTGATTGGTCTCATTCTTTCACTGATTTTAATAA

TATTCCGGAAACAATATCCAGTGTTATTTTCAAATGATCCAGAAGTGAGAGAGGTAGTGATTGAGTTGACACCAATGTTG

GCATTATGCATTGTCATCAACAACATTCAGCCTGTTCTTTCAGGTGTTGCCATCGGTGCTGGGTGGCAATCAGCTGTTGC

TTATGTAAATATTGCATGTTACTATCTCTTTGGTATTCCTTTGGGTCTCTTCTTTGGTTACTATCTTGACTTTGGTGTCT

TGGGGATTTGGTCTGGAATGCTGTCAGGGACAGTCCTACAAACTCTTGTGTTATTCTTCATGGTCTATAGAACTGACTGG

AATAATGAGGCATCACTTGCAGAAGAAAGGATAAGCAAGTGGGGTGGGCAAAAAGTTTTAAAGATGAACGATAATGGAAA

AGATATTCAAGAAACATGA

>MsMATE64

ATGGAGGAAAATGACACACAAACCTTCCCACTCCTCACACCATTAAACAACCAACAACATGATCAAATCAACACAGCA

GTATTCACAGCTAAATCCGATGATATTTCTCCGATCATCGGTGCCGGAGACTTTGCCAGAGAATTTCTCAATGAATCT

AAGAAACTTTGGTACCTTGCTGGACCTGCCATATTCACCTCCATCTCTCAATATTCTCTTGGTGCTGTTACTCAAGTC

TTTGCTGGTCAAGTTGGGACTCTTCAACTTGCGGCTGTTTCCGTCGAAAACTCCGTCATCGCCGGCTTTTGCTTAGGC

ATCACGATGGGGATGGGAAGCGCATTGGAGACACTATGTGGACAAGCTTTTGGAGCCGGAAAACTTAATATGTTAGGA

ATATACATGCAAAGATCATGGTTGATTCTCAGTGCCACCGCGCTTATTCTTAGTTTTCTCTACATTTTCGCGTCGCCT

CTTCTAAAACTTATAGGCCAAACAACCGCGATATCAGAGGCAGCCGGAGTTTTTGCACTATGGATGATTCCTCAACTC

TTCGCTTACGCGGTGAACTTTCCAACCCAAAAGTTCTTGCAAGCCCAGAGTAAGATCATGGCTATGGCATGGATTTCT

GCGGTGGCGTTGGTGGGACACACATTTTTTAGTTGGTTCCTAATGTTGCACCTAGGGTGGGGACTTGTTGGTGCAGCC

GTGGTGCTTAATTCTTCGTGGTGGTTTATTGTTTTGGCTCAAATTGTTTATGTTTTGAGTGGTTCTTGTGGTGAAGCT

TGGAGTGGTTTTTCTTTTCAAGCCTTTCAGAATCTTTGGGGTTTTGTTCGTCTCTCACTAGCTTCTGCTGTTATGATG

TGCCTTGAAGTATGGTATTTTATGGCATTAATACTATTTGCTGGATATCTGGAGAATGCAGAAGTTTCAGTTGATGCA

TTGTCTATATGCGCAAACATATTGGGGTGGACTGTCATGGCATCCTTTGGAATTAATGCAGCCATAAGTGTGAGGGTA

TCAAATGAATTGGGAGCATCTCATCCAAGAGCAGCAAAATTCTCACTAGTGGTTGCAGTGATTACATCATTTGTGATT

GGTCTCATTCTTTCACTGATTTTAATAATATTCCGGAAACAATATCCAGTATTATTTTCAAATGATCCAGAAGTGAGA

GAGGTAGTGATTGAGTTGACACCAATGTTGGCATTATGCATTGTCATCAACAATATTCAGCCTGTTCTTTCAGGTGTT

GCCATCGGTGCTGGGTGGCAATCAGCTGTTGCTTATGTAAATATTGCATGTTACTATCTCTTTGGTATTCCTTTGGGT

CTCTTCTTTGGTTACTATCTTGACTTTGGTGTCTTGAGAATGCCAAGATTTGAAATTGAGAAGGAGAAAGAGGAAAGA

CTCAAGAATCACCAGTAG

>MsMATE65

ATGGAGGAAAATGACACACAAACCTTCCCACTCCTCACACCATTAAACAACCAACAACATGATCAAATCAACACAGCAGT

ATTCACAGCTAAATCCGATGATATTTCTCCGATCATCGGTGCCGGAGACTTTGCCAGAGAATTTCTCAATGAATCTAAGA

AACTTTGGTACCTTGCTGGACCTGCCATATTCACCTCCATCTCTCAATATTCTCTTGGTGCTGTTACTCAAGTCTTTGCT

GGTCAAGTTGGGACTCTTCAACTTGCGGCTGTTTCCGTCGAAAACTCCGTCATCGCCGGCTTTTGCTTAGGCATCACGAT

GGGGATGGGAAGCGCATTGGAGACACTATGTGGACAAGCTTTTGGAGCCGGAAAACTGAACATGTTAGGAATATACATGC

AAAGATCATGGTTGATTCTCAGCGCCACCGCGCTTATTCTTAGCTTTCTCTACATTTTCGCGTCGCCTCTTCTAAAACTT

ATAGGCCAAACAACCGCGATATCAGAGGCAGCCGGAGTTTTTGCACTATGGATGATTCCTCAACTCTTCGCCTACGCGAT

AAACTTTCCAACCCAAAAGTTCTTGCAAGCCCAGAGTAAGATCATGGCTATGGCATGGATTTCTGCGGTAGCGTTGGTGG

GACACACATTTTTTAGTTGGTTCCTAATGTTGCACCTAGGGTGGGGACTTGTTGGTGCAGCCGTGGTGCTTAATTCTTCG

TGGTGGTTTATTGTTTTGGCTCAAATTGTTTATGTTTTGAGTGGTTCTTGTGGTGAAGCTTGGAGTGGTTTTTCTTTTCA

AGCCTTTCAGAATCTTTGGGGTTTTGTTCGTCTCTCACTAGCTTCTGCTGTTATGATGTGCCTTGAAGTATGGTATTTTA

TGGCATTAATACTATTTGCTGGATATCTGGAGAATGCAGAAGTTTCAGTTGATGCATTGTCTATATGGTGA

>MsMATE66

ATGGATGACTCAACACAGCCACTTCTCACACCCAAATCAAAAGAACAACGCCATGAAACCAACACAAACTTTCCCTTACC

CAACTCACCACCTTCTAACACCGCAATATTCACCGCCGCAGCCCCCGACATGGACCTGATCACAAGCCCCAAAGACTTCT

TCAAACAATTCATCGTTGAGTCAAAGATGCTGTGGTACCTTGCCGGGCCTGCTATCTTCTCCTTCGTCTCCAAATATTCC

CTTGGAGCCGTTACTCAAATCTTCGCCGGTCATGTTAGTACCATCGATCTCGCTGCTGTCTCCGTCGAAAACTCCCTCAT

CGCTGGTTTCTCCTTTGGCTTGATGCTTGGGATGGGAAGTGCGCTCGAAACACTATGTGGACAAGCAGTAGGAGCAGGGA

AACTCGACATGTTAGGAATATACATGCAAAGATCATGGGTGATACTATTTTCCATGGCATTTCCGCTATGCCTTTTATAC

ATCTTCGCCGAATCCATTCTAAAATTCATAGGACAAACAACTGAAATATCCGAGGCTGCAGGAACATTTGCTTTATACAT

GATTCCACAATTATTCGCTTACGCGCTGAATTTTCCTGTCGCGAAATTTCTACAAGCGCAAAGTATGGTGATTGTCATCG

CGGTTATATCGGGGGTAGCTATGGTGTTGCATCCGATTTTTAGTTGGTTGTTAATGGTGAAATTTGGGTGGGGATTGGTG

GGTGCTGCGGTGGTTCTTAATGGATCGTGGTGGTTTATTGTGGTGGCTCAATTAGGGTATGTGTTTAGTGGGAAATGTGG

TATAGCTTGGAATGGATTTTCTTTTGAAGCATTTAGGAATCTTTGGGGATTCTTTCGTCTTTCTTTGGCTTCTGCTGTGA

TGTTATGCTTAGAAACATGGTACTTTATGGCATTGATATTGTTTGCTGGATATCTCAAGAATGCAGAAATCTCAGTAGAC

GCCTTCTCTATCTGCATGAATATATTGGGCTGGACCATCATGGTGTCATTTGGAATGAACGTTGCCGTAAGTGTGAGAGT

CTCAAATGAACTAGGAGCAGTTCACCCAAGAACAGCAAGATTTTCATTGGTGGTAGCCGTGATTACATCGATTTTAATCG

GAATATTATTGGCTCTTGTTTTGATTATTTCACGGGACAAGTACCCTGCCTATTTTACCAATGATAAAGAAGTGCAAGAT

TTAGTCAAGGATCTCACACCTTTGTTGGCATTATGTGTTGTCATTAATAATGTTCAACCTGTTCTTTCAGGGGTGGCCAT

TGGGGCAGGATGGCAAGCTGCTGTGGCCTATGTTAACATTGCATGCTACTACCTATTTGGAATTCCTGTGGGTCTTATTT

TGGGTTACAAGGTTAACTTGGGAGTCAAGGGAATATGGTGTGGAATGATGTCAGGGACTATATTGCAAACATGTGTTCTG

TTGCTAATGGTTTACAAAACTAACTGGAACAAAGAGGCCTCCCTAGCTGAAGATAGAATAAGGAGTTGGGGTGGACCTCG

AGAAGTGACTGAAGCCAAAGAAGAAAACATACAAGAGACATGA

>MsMATE67

ATGTTCATGATGAAACAGTTGGGCATGGGGAGTGCTGTTGAGACACTTTGTGGACAAGCATTTGGAGCAAAAAAATAT

GAAATGTTAGGCATATATTTGCAAAGATCAACAGTGCTTCTCACATTAGCTGGTTTAATTTTAACAATAATATACATA

TTCTCCGAACCAATTTTAATATTTCTAGGAGAATCACCAAAAATTGCATCAGCAGCATCACTTTTTGTCTTTGGTTTA

ATCCCACAAATATTTGCCTATGCTATAAACTTTCCAATACAAAAATTTCTTCAAGCACAAAGCATAGTGGCACCAAGT

GCATACATATCAGCAGCAACATTGGTTATTCACCTTGCATTAAGTTATGTTGTTATCTACCAAATTGGGCTTGGTTTA

TTAGGTGCTTCATTGGTTTTGAGTATTTCTTGGTGGATAATTGTGATTGCACAATTTGTTTATATTGTGAAGAGTGAA

AAGTGTAAGCATACTTGGAAAGGGTTTAGTTTTCAAGCTTTTTCAGGGTTGCCAGAGTTTTTTAAATTGTCAGCTGCA

TCAGCTGTGATGTTGTGTTTAGAGACTTGGTACTTTCAAATCTTGGTTTTGCTTGCTGGGTTACTTCCTCATCCTGAA

TTGGCTCTTGATTCTCTTTCTATTTGTACCACAGTGTCTGGATGGACGTTCATGATCTCAGTTGGATTTCAAGCAGCT

GCAAGGTTTGGTTCTCCTTTAATATTTATTGATCGATTTCTGTTTATCATTGGTTGA

>MsMATE68

ATGTTCATGATGAAACAGTTGGGCATGGGGAGTGCTGTTGAGACACTTTGTGGACAAGCATTTGGAGCAAAAAAATATGA

AATGTTAGGCATATATTTGCAAAGATCAACAGTGCTTCTCACATTAGCTGGTTTAATTTTAACAATAATATACATATTCT

CCGAACCAATTTTAATATTTCTAGGAGAATCACCAAAAATTGCATCAGCAGCATCACTTTTTGTCTTTGGTTTAATCCCA

CAAATATTTGCCTATGCTATAAACTTTCCAATCCAAAAATTTCTTCAAGCACAAAGTATAGTTCTACCAAGTGCATACAT

TTCAGCAGGAACTTTAGTTTTTCATCTAATATTGAGTTGGGTTGTTGTGTTTAAGATTGGTTTGGGACTATTGGGAGCTT

CATTGGTTTTGAGTTTTTCTTGGTGGGTTATTGTTGTGGCACAGTTTATTTATATTTTGAAGAGTGAGAAGTGTAAGAGG

ACTTGGAATGGGTTTACTTGGGAGGCGTTTTCAGGGTTGCCGGAGTTTTTTAAATTGTCAGCGGCGTCGGCGGTTATGCT

TTGTTTGGAGTCTTGGTATTTTCAGATTTTGGTTTTGCTTGCTGGACTTCTTCCTGAACCTGAGTTGGCTCTTGATTCTC

TTTCTATTTGTACCACAGTGTCTGGATGGACGTTCATGATCTCAGTTGGATTTCAAGCAGCTGCAAGTGTGAGAGTGAGC

AATGAATTAGGAGCAGGAAATTCAAAATCAGCTTCATTTTCAGTGGTGGTGGTAACAGTGATTTCTTTCATAATATGTGC

AATTATAGCACTTGTGGTGCTTGCATTAAGGGATGTTATCAGCTATGTCTTCACAGACGGTGAAGAGGTTGCTGCTGCTG

TCTCAGATCTCAGTCCACTCCTTGCTCTTGCCATTGTCCTAAATGGTGTTCAACCTGTCTTATCTGGGGTGGCCGTTGGA

TGTGGATGGCAAACATTTGTTGCGTATGTAAACGTTGGTTGTTATTACGGGATTGGCATACCTTTGGGTGCAGTTCTTGG

TTTCTATTTCAAATTTGGTGCCAAGGGAATATGGTTGGGAATGTTAGGTGGCACGGTTTTGCAAACAATTATTTTAATGT

GGGTAACATTTCGAACGGATTGGAATAATGAGGTTGTAGAATCAAACAAGAGGCTAAACAAGTGGGAGGGCAAGACCGAG

TCACTCCTCAAGAATTGA

>MsMATE69

ATGGCTACTTCTACTCATGTTCACAAAGATATTGATGAACCTTTGCTAGTACCAAGTGAACCAACACCAATACTACTATC

ATCATTCACTCATTCATTTGGTTCAAAGCATGAATCAGATGGTGAACTTGAACGAATACTCTCAGATACAAGTGTTCCTT

TTGTGAAACGTATTAGACATGCTACATGGGTTGAATTTAAGCTACTTTTTTACCTAGCTGCACCTGCTGTAATTGTGTAC

CTTATCAACTATGTGATGTCAATGTCTACTCAAATATTTTCTGGTCATCTTGGTAATTTGGAGCTTGCTGCTGCTTCCCT

TGGAAATAATGGTATCCAAATATTTGCTTATGGACTCATGTTGGGCATGGGGAGTGCTGTTGAGACACTTTGTGGACAAG

CATTTGGAGCAAAAAAATATGAAATGTTAGGCATATATTTGCAAAGATCAACAGTGCTTCTCACATTAGCTGGTTTAATT

TTAACAATAATATACATATTCTCCGAACCAATTTTAATATTTCTAGGAGAATCACCAAAAATTGCATCAGCAGCATCACT

TTTTGTCTTTGGTTTAATCCCACAAATATTTGCCTATGCTATAAACTTTCCAATACAAAAATTTCTTCAAGCACAAAGCA

TAGTGGCACCAAGTGCATACATATCAGCAGCAACATTGGTTATTCATCTTGCATTAAGTTATGTTGTTATCTACCAAATT

GGGCTTGGTTTATTAGGTGCTTCATTGGTTTTGAGTATTTCTTGGTGGATAATTGTGATTGCACAATTTGTTTATATTGT

GAAGAGTGAAAAGTGTAAGCATACTTGGAAAGGGTTTAGTTTTCAAGCTTTTTCAGGGTTGCCAGAGTTTTTTAAATTGT

CAGCTGCATCAGCTGTGATGTTGTGTTTAGAGACTTGGTACTTTCAAATCTTGGTTTTGCTTGCTGGCTTACTTCCTCAT

CCTGAATTGGCTCTTGATTCTCTTTCTATTTGTACCACAGTGTCTGGATGGACGTTCATGATCTCAGTTGGATTTCAAGC

AGCTGCAAGTGTGAGAGTGAGCAATGAACTAGGAGCAAGAAATCCAAAATCAGCATCATTTTCAGTGAAAGTGGTGACAT

TGATATCCTTTCTCATATCAGTAATAGCAGCACTGATTGTGCTTGCATTAAGAGATGTTATTAGCTATGTTTTCACTGAG

GGTGAAGTGGTGGCTGCTGCTGTCTCAGATCTTTGTCCACTTCTCTCTCTTTCTCTTGTCCTCAATGGCATTCAGCCTGT

CCTATCTGGGGTGGCTGTTGGTTGTGGATGGCAAGCTTTTGTTGCTTATGTGAATGTGGGTTGTTATTATATAATTGGTA

TACCATTGGGGGCGGTTCTTGGCTTTTATTTCAATTTTGGTGCTAAGGGAATATGGCTAAGAATGCTTGGTGGAACCACC

ATGCAGACAATTATTCTAATGTGGGTCACATTTAGAACGGATTGGAACAAAGAGGTGAAAGAAGCAGCCAAGAGGTTGAA

CAAATGGGAGGATAAGAAAAAGGAGCCGCTTCTAAACTAG

>MsMATE70

ATGGCTACTTCTACTCATGTTCACAAAGATATTGATGAACCTTTGCTAGTACCAAGGGAACCAACACCAATACTACTATC

ATCATTCACTCATTCATGTGGTTCAAAGCATGAATCAGATGGTGAACTTGAACGAATACTCTCAGATACAAGTGTTCCTT

TTGTGAAACGTATTAGACATGCTACATGGGTTGAATTTAAGCTACTTTTTTACCTAGCTGCACCTGCTGTAATTGTGTAC

CTTATCAACTATGTGATGTCAATGTCTACTCAAATATTTTCTGGTCATCTTGGTAATTTGGAGCTTGCTGCTGCTTCCCT

TGGAAATAATGGTATCCAAATATTTGCTTATGGACTCATGTTGGGCATGGGGAGTGCTGTTGAGACACTATGTGGACAAG

CATTTGGAGCAAAAAAATATGAAATGTTAGGCATATATTTGCAAAGATCAACAGTGCTTCTCACAATAGCCGGTTTAATT

TTAACAATAATATACATATTCTCCGAACCAATTTTAATATTTCTAGGAGAATCACCAAAAATTGCATCAGCAGCATCACT

TTTTGTCTTTGGTTTAATCCCACAAATATTTGCCTATGCTATAAACTTTCCAATACAAAAATTTCTTCAAGCACAAAGCA

TAGTGGCACCAAGTGCATACATATCAGCAGCAACATTGGTTATTCACCTTGCATTAAGTTATGTTGTTATCTACCAAATT

GGGCTTGGTTTATTAGGTGCTTCATTGGTTTTAAGTATTTCTTGGTGGATAATTGTGATTGCACAATTTGTTTATATTGT

GAAGAGTGAAAAGTGTAAGCATACTTGGAAAGGGTTTAGTTTTCAAGCTTTTTCAGGGTTGCCAGAGTTTTTTAAATTGT

CAGCTGCATCAGCTGTGATGTTGTGTTTAGAGACTTGGTACTTTCAAATCTTGGTTTTGCTTGCTGGCTTACTTCCTCAT

CCTGAATTGGCTCTTGATTCTCTTTCTATTTGTACCACAGTGTCTGGATGGACGTTCATGATCTCAGTTGGATTTCAAGC

AGCTGCAAGTGTGAGAGTGAGCAATGAATTAGGAGCAGGAAATTCAAAATCAGCTTCATTTTCAGTGGTGGTGGTAACAG

TGATTTCTTTCATAATATGTGCAATTATAGCACTTGTGGTGCTTGCATTAAGGGATGTTATCAGCTATGTCTTCACAGAC

GGTGAAGAGGTTGCTGCTGCTGTCTCAGATCTCAGTCCACTCCTTGCTCTTGCCATTGTCCTAAATGGTGTTCAACCTGT

CTTATCTGGGGTGGCCGTTGGATGTGGATGGCAAACATTTGTTGCGTATGTAAACGTTGGTTGTTATTACGGGATTGGCA

TACCTTTGGGTGCAGTTCTTGGTTTCTATTTCAAATTTGGTGCCAAGGGAATATGGTTGGGAATGTTAGGTGGCACGGTT

TTGCAAACAATTATTTTAATGTGGGTAACATTTCGAACGGATTGGAATAATGAGGTTGTAGAATCAAACAAGAGGCTAAA

CAAGTGGGAGGGCAAGACCGAGTCACTCCTCAAGAATTGA

>MsMATE71

ATGGCTACTTCTACTCATGTTCACAAAGATATTGATGAACCTTTGCTAGTACCTAGTGAACCAACACCAATACTACTATC

ATCATTCACTCATTCATTTGGTTCAAAGCATGAATCAGATGGTGAACTTGAACGAATACTCTCAGATACAAGTGTTCCTT

TTGTTAAACGTATTAGACATGCTACATGGGTTGAATTTAAGCTACTTTTTTACCTAGCTGCCCCTGCTGTAATTGTGTAC

CTTATCAACTATGTGATGTCAATGTCTACTCAAATATTTTCTGGTCATCTTGGTAATTTGGAGCTTGCTGCTGCTTCCCT

TGGAAATAATGGTATCCAAATCTTTGCTTATGGACTCATGTTGGGCATGGGGAGTGCTGTTGAGACACTATGTGGACAAG

CATTTGGTGCAAAAAAGTATGAAATGTTAGGCATATATTTGCAAAGATCAACAGTGCTTCTCACAATAGCCGGTTTAATT

TTAACAATAATATACATATTCTCCGAACCAATTTTAATATTTCTAGGAGAATCACCAAAAATTGCATCAGCAGCATCACT

TTTTGTCTTTGGTTTAATCCCACAAATATTTGCCTATGCTATAAACTTTCCAATCCAAAAATTTCTTCAAGCACAAAGTA

TAGTTCTACCAAGTGCATACATTTCAGCAGGAACTTTAGTTTTTCATCTAATATTGAGTTGGGTTGTTGTGTTTAAGATT

GGTTTGGGACTATTGGGAGCTTCATTGGTTTTGAGTTTTTCTTGGTGGGTTATTGTTGTGGCACAGTTTATTTATATTTT

GAAGAGTGAAAAGTGTAAGAGGACTTGGAATGGGTTTACTTGGGAGGCGTTTTCGGGGTTGCCGGAGTTTTTTAAATTGT

CGGCCGCGTCGGCGGGTATGCTTTGTTTAGAGTCTTGGTATTTTCAGATTTTGGTTTTGCTTGCTGGACTTCTTCCTCAA

CCTGAGTTGGCTCTTGATTCTCTTTCTATTTGTTCCACAGTTTCTGGATGGGTGTTCATGATCTCAGTTGGATTCAATGC

AGCTGCAAGTGTGAGAGTGAGCAATGAACTAGGAGCAAGAAATCCAAAATCAGCATCATTTTCAGTGAAAGTGGTGACAT

TGATATCCTTTCTCATATCAGTAATAGCAGCACTGATTGTGCTTGCATTAAGAGATGTTATTAGCTATGTTTTCACTGAG

GGTGAAGTGGTGGCTGCTGCTGTCTCAGATCTTTGTCCACTTCTCTCTCTTTCTCTTGTCCTCAATGGCATTCAGCCTGT

CCTATCTGGGGTGGCTGTTGGTTGTGGATGGCAAGCTTTTGTTGCTTATGTGAATGTGGGTTGTTATTATATAATTGGTA

TACCATTGGGGGCGGTTCTTGGCTTTTATTTCAATTTTGGTGCTAAGGGAATATGGCTAGGAATGCTTGGTGGAACCACC

ATGCAGACAATTATTCTAATGTGGGTCACATTTAGAACGGATTGGAACAAGGAGGTGAAAGAAGCAGCCAAGAGGTTGAA

CAAATGGGAGGATAAGAAAAAGGAGCCCCTTCTAAACTAA

>MsMATE72

ATGGCTTCAGTGAAGCAAAACAGTGCTGAAGAACCACTATTGTTCAACCACAGTGGTACATCTCAGAAGCACCACCATGA

ATCTGATGGTGAGCTTGAAAGGATACTATCAGACACCACCGTGCCATTCTTCAGCCGTATCGGCTCCGCCACATGGATTG

AGCTCAGACTCCTTTTCTTGTTGGCTGCTCCAGCTGTTTTTGTTTATCTTATTAATTATGTTATGTCTATGTCCACACAA

ATCTTTTCCGGCCACCTTGGTAATCTTGAGCTTGCTGCGGCGTCTCTTGGAAACACCGGGATTCAAATCTTTGCTTATGG

TCTCATGCTGGGTATGGGAAGTGCAGTTGAGACACTATGTGGACAAGCATATGGAGCAGAAAAATATGACATGCTAGGAA

CATATTTACAAAGATCAACAATTCTTCTAACAATAACCGGTTTTTTCCTCACAGTTATTTACGTATTAAGCGAACCAATC

CTAGTATTCATTGGACAATCACCAAGAATAGCTTCTGCAGCAGCACTTTTTGTGTATGGACTTATCCCACAAATATTTGC

TTATGCAGTAAATTTTCCAATCCAAAAATTTCTTCAAGCACAAAGCATAGTGGCACCAAGTGCATACATATCAGCAGCAA

CATTGGTTATTCACCTTGCATTAAGTTATGTTGTTATCTACCAAATTGGGCTTGGTTTATTAGGTGCTTCATTGGTTTTA

AGTATTTCTTGGTGGATAATTGTGATTGCACAATTTGTTTATATTGTGAAGAGTGAAAAGTGTAAGCATACTTGGAAAGG

GTTTAGTTTTCAAGCTTTTTCAGGGTTGCCAGAGTTTTTTAAATTGTCAGCTGCATCAGCTGTGATGTTGTGTTTAGAGA

CTTGGTACTTTCAAATCTTGGTTTTGCTTGCTGGGTTACTTCCTCATCCTGAATTGGCTCTTGATTCTCTTTCTATTTGT

ACCACAGTGTCTGGATGGACGTTCATGATCTCAGTTGGATTTCAAGCAGCTGCAAGTGTGAGAGTGAGCAATGAATTAGG

AGCAGGAAATTCAAAATCAGCTTCATTTTCAGTGGTGGTGGTAACAGTGATTTCTTTCATAATATGTGCAATTATAGCAC

TTGTGGTGCTTGCATTAAGGGATGTTATCAGCTATGTCTTCACAGACGGTGAAGAGGTTGCTGCTGCTGTCTCAGATCTC

AGTCCACTCCTTGCTCTTGCCATTGTCCTAAATGGTGTTCAACCTGTCTTATCTGGGGTGGCCGTTGGATGTGGATGGCA

AACATTTGTTGCGTATGTAAACGTTGGTTGTTATTACGGGATTGGCATACCTTTGGGTGCAGTTCTTGGTTTCTATTTCA

AATTTGGTGCCAAGGGAATATGGTTGGGAATGTTAGGTGGCACGGTTTTGCAAACAATTATTTTAATGTGGGTAACATTT

CGAACGGATTGGAATAATGAGGTTGTAGAATCAAACAAGAGGCTAAACAAGTGGGAGGGCAAGACCGAGTCACTCCTCAA

GAATTGA

>MsMATE73

ATGGCTTCAGTGAAGCAAAACAGTGCTGAAGAACCACTATTGTTCAACCACAGTGGTACATCTCAGAAGCACCACCATGA

ATCTGATGGTGAGCTTGAAAGGATACTATCAGACACCACCGTGCCATTCTTCAGCCGTATCGGCTCCGCCACATGGATTG

AGCTCAGACTCCTTTTCTTGTTGGCTGCACCAGCTGTTTTTGTTTATCTTATTAATTATGTTATGTCTATGTCCACACAA

ATCTTTTCCGGCCACCTTGGTAATCTTGAGCTTGCCGCGGCGTCTCTGGGAAACACCGGGATTCAAATCTTTGCTTATGG

TCTCATGCTGGGTATGGGAAGTGCAGTTGAGACACTATGTGGACAAGCATATGGAGCAGAAAAATATGACATGCTAGGAA

CATATTTACAAAGATCAACAATTCTTCTAACAATAACCGGTTTTTTCCTCACAGTTATTTACGTATTAAGCGAACCAATC

CTAGTATTCATTGGACAATCACCAAGAATAGCTTCTGCAGCAGCACTTTTTGTGTATGGACTTATCCCACAAATATTTGC

TTATGCAGTAAATTTTCCAATCCAAAAATTTCTTCAAGCACAAAGCATAGTGGCACCAAGTGCATACATATCAGCAGCAA

CATTGGTTATTCACCTTGTATTAAGTTATGTTGTTATCTACCAAATTGGGCTTGGTTTATTAGGTGCTTCATTGGTTTTA

AGTATTTCTTGGTGGATAATTGTGATTGCACAATTTGTTTATATTGTGAAGAGTGAAAAGTGTAAGCATACTTGGCAAGG

GTTTAGTTTTCAAGCTTTTTCAGGGTTGCCAGAGTTTTTTAAATTGTCAGCTGCTTCAGCTGTGATGTTGTGTTTAGAGA

CTTGGTACTTTCAAATCTTGGTTTTGCTTGCTGGGTTACTTCCTCATCCTGAATTGGCTCTTGATTCTCTTTCTATTTGT

ACCACAGTGTCTGGATGGACGTTCATGATCTCAGTTGGATTTCAAGCAGCTGCAAGTGTGAGAGTGAGCAATGAACTAGG

AGCAAGAAATCCAAAATCAGCATCATTTTCAGTGAAAGTGGTGACATTGATATCCTTTCTCATATCAGTAATAGCAGCAC

TGATTGTGCTTGCATTAAGAGATGTTATTAGCTATGTTTTCACTGAGGGTGAAGTGGTGGCTGCTGCTGTCTCAGATCTT

TGTCCACTTCTCTCTCTTTCTCTTGTCCTCAATGGCATTCAGCCTGTCCTATCTGGGGTGGCTGTTGGTTGTGGATGGCA

AGCTTTTGTTGCTTATGTGAATGTGGGTTGTTATTATATAATTGGTATACCATTGGGGGCGGTTCTTGGCTTTTATTTCA

ATTTTGGTGCTAAGGGAATATGGCTAGGAATGCTTGGTGGAACCACCATGCAGACAATTATTCTAATGTGGGTCACATTT

AGAACGGATTGGAACAAGGAGGTAAAAGACAACTTTGCAATTATTTCATTTCTATGA

>MsMATE74

ATGGCTTCAGTGAAGCAAAATAGTGCTGAAGAACCACTATTGTTCAACCACAGTGGTACATCACAGAAGCAGCATGAATC

TGACGGCGAGCTTGAGAGGATACTATCAGACACCACCGTGCCATTCTTCAGCCGTATCGGCTCCGCCACATGGATTGAGC

TCAGACTTCTTTTCTTGTTGGCTGCACCAGCTGTTTTTGTTTATCTTATTAATTATGTTATGTCTATGTCCACACAAATC

TTTTCCGGCCACCTTGGTAATCTTGAGCTTGCTGCGGCGTCTCTTGGAAACACCGGGATTCAAATCTTTGCTTATGGTCT

CATGCTGGGTATGGGAAGTGCAGTTGAGACACTATGTGGACAAGCATATGGAGCAGAAAAATATGACATGCTAGGAACAT

ATTTACAAAGATCAACAATTCTTCTAACAATAACCGGTTTTTTCCTCACAGTTATTTACGTATTAAGCGAACCAATCCTA

GTATTCATTGGACAATCACCAAGAATAGCTTCTGCAGCAGCACTTTTTGTGTATGGACTTATCCCACAAATATTTGCTTA

TGCAGTAAATTTTCCAATCCAAAAATTTCTTCAAGCACAAAGCATAGTGGCACCAAGTGCATACATATCAGCAGCAACAT

TGGTTATTCACCTTGCATTAAGTTATGTTGTTATCTACCAAATTGGGCTTGGTTTATTAGGTGCTTCATTGGTTTTAAGT

ATTTCTTGGTGGATAATTGTGATTGCACAATTTGTTTATATTGTGAAGAGTGAAAAGTGTAAGCATACTTGGAAAGGGTT

TAGTTTTCAAGCTTTTTCAGGGTTGCCAGAGTTTTTTAAATTGTCAGCTGCATCAGCTGTGATGTTGTGTTTAGAGACTT

GGTACTTTCAAATCTTGGTTTTGCTTGCTGGCTTACTTCCTCATCCTGAATTGGCTCTTGATTCTCTTTCTATTTGTACC

ACAGTGTCTGGATGGACGTTCATGATCTCAGTTGGATTCAACGCAGCTGCAAGGTTTGGTTCTCCTTTAATATTTATTGA

TCGATTTCTGTTTATCATTGGTTGA

>MsMATE75

ATGGCTTCAGTGAAGCAAAACAGTGCTGAAGAACCACTATTGTTCAACCACAGTGGTACATCTCAGAAGCACCACCATGA

ATCTGATGGTGAGCTTGAAAGGATACTATCAGACACCACCGTGCCATTCTTCAGCCGTATCGGCTCCGCCACATGGATTG

AGCTCAGACTCCTTTTCTTGTTGGCTGCACCAGCTGTTTTTGTTTATCTTATTAATTATGTTATGTCTATGTCCACACAA

ATCTTTTCCGGCCACCTTGGTAATCTTGAGCTTGCCGCGGCGTCTCTGGGAAACACCGGGATTCAAATCTTTGCTTATGG

TCTCATGCTGGGTATGGGAAGTGCAGTTGAGACACTATGTGGACAAGCATATGGAGCAGAAAAATATGACATGCTAGGAA

CATATTTACAAAGATCAACAATTCTTCTAACAATAACCGGTTTTTTCCTCACAGTTATTTACGTATTAAGCGAACCAATC

CTAGTATTCATTGGACAATCACCAAGAATAGCTTCTGCAGCAGCACTTTTTGTGTATGGACTTATCCCACAAATATTTGC

TTATGCAGTAAATTTTCCAATCCAAAAATTTCTTCAAGCACAAAGTATAGTTCTACCAAGTGCATACATTTCAGCAGGAA

CTTTAGTTTTTCATCTAATATTGAGTTGGGTTGTTGTGTTTAAGATTGGTTTGGGACTATTGGGAGCTTCATTGGTTTTG

AGTTTTTCTTGGTGGGTTATTGTTGTGGCACAGTTTATTTATATTTTGAAGAGTGAAAAGTGTAAGAGGACTTGGAATGG

GTTTACTTGGGAGGCGTTTTCGGGGTTGCCGGAGTTTTTTAAATTGTCGGCCGCGTCGGCGGGTATGCTTTGTTTAGAGT

CTTGGTATTTTCAGATTTTGGTTTTGCTTGCTGGACTTCTTCCTCAACCTGAGTTGGCTCTTGATTCTCTTTCTATTTGT

TCCACAGTTTCTGGATGGGTGTTCATGATCTCAGTTGGATTCAATGCAGCTGCAAGTGTGAGAGTGAGCAATGAACTAGG

AGCAAGAAATCCAAAATCAGCATCATTTTCAGTGAAAGTGGTGACATTGATATCCTTTCTCATATCAGTAATAGCAGCAC

TGATTGTGCTTGCATTAAGAGATGTTATTAGCTATGTTTTCACTGAGGGTGAAGTGGTGGCTGCTGCTGTCTCAGATCTT

TGTCCACTTCTCTCTCTTTCTCTTGTCCTCAATGGCATTCAGCCTGTCCTATCTGGGGTGGCTGTTGGTTGTGGATGGCA

AGCTTTTGTTGCTTATGTGAATGTGGGTTGTTATTATATAATTGGTATACCATTGGGGGCGGTTCTTGGCTTTTATTTCA

ATTTTGGTGCTAAGGGAATATGGCTAGGAATGCTTGGTGGAACCACCATGCAGACAATTATTCTAATGTGGGTCACATTT

AGAACGGATTGGAACAAGGAGGTGAAAGAAGCAGCCAAGAGGTTGAAAAGACAACTTTGCAATTATTTCATTTCTATGAT

GACACTGTGCAGGAAATGGGAGAATCATTGTTATTGTGTCTAA

>MsMATE76

ATGGCTTCAGTGAAGCAAAACAGTGCTGAAGAACCACTATTGTTCAACCACAGTGGTACATCTCAGAAGCACCACCATGA

ATCTGATGGTGAGCTTGAAAGGATACTATCAGACACCACCGTGCCATTCTTCAGCCGTATCGGCTCCGCCACATGGATTG

AGCTCAGACTCCTTTTCTTGTTGGCTGCACCAGCTGTTTTTGTTTATCTTATTAATTATGTTATGTCTATGTCCACACAA

ATCTTTTCCGGCCACCTTGGTAATCTTGAGCTTGCCGCGGCGTCTCTGGGAAACACCGGGATTCAAATCTTTGCTTATGG

TCTCATGCTGGGTATGGGAAGTGCAGTTGAGACACTATGTGGACAAGCATATGGAGCAGAAAAATATGACATGCTAGGAA

CATATTTACAAAGATCAACAATTCTTCTAACAATAACCGGTTTTTTCCTCACAGTTATTTACGTATTAAGCGAACCAATC

CTAGTATTCATTGGACAATCACCAAGAATAGCTTCTGCAGCAGCACTTTTTGTGTATGGACTTATCCCACAAATATTTGC

TTATGCAGTAAATTTTCCAATCCAAAAATTTCTTCAAGCACAAAGTATAGTTCTACCAAGTGCATACATTTCAGCAGGAA

CTTTAGTTTTTCATCTAATATTGAGTTGGGTTGTTGTGTTTAAGATTGGTTTGGGACTATTGGGAGCTTCATTGGTTTTG

AGTTTTTCTTGGTGGGTTATTGTTGTGGCACAGTTTATTTATATTTTGAAGAGTGAAAAGTGTAAGAGGACTTGGAATGG

GTTTACTTGGGAGGCGTTTTCGGGGTTGCCGGAGTTTTTTAAATTGTCGGCCGCGTCGGCGGTTATGCTTTGTTTAGAGT

CTTGGTATTTTCAGATTTTGGTTTTGCTTGCTGGACTTCTTCCTCAACCTGAGTTGGCTCTTGATTCTCTTTCTATTTGT

ACCACAGTTTCTGGATGGGTGTTCATGATCTCAGTTGGATTCAATGCAGCTGCAAGTGTGAGAGTGAGCAATGAACTAGG

AGCAAGAAATCCAAAATCAGCATCATTTTCAGTGAAAGTGGTGACATTGATATCCTTTCTCATATCAGTAATAGCAGCAC

TGATTGTGCTTGCATTAAGAGATGTTATTAGCTATGTTTTCACTGAGGGTGAAGTGGTGGCTGCTGCTGTCTCAGATCTT

TGTCCACTTCTCTCTCTTTCTCTTGTCCTCAATGGCATTCAGCCTGTCCTATCTGGGGTGGCTGTTGGTTGTGGATGGCA

AGCTTTTGTTGCTTATGTGAATGTGGGTTGTTATTATATAATTGGTATACCATTGGGGGCGGTTCTTGGCTTTTATTTCA

ATTTTGGTGCTAAGGGAATATGGCTAGGAATGCTTGGTGGAACCACCATGCAGACAATTATTCTAATGTGGGTCACATTT

AGAACGGATTGGAACAAGGAGGTGAAAGAAGCAGCCAAGAGGTTGAACAAATGGGAGGATAAGAAAAAGGAGCCCCTTCT

AAACTAA

>MsMATE77

ATGGCTTCAGTGAAGCAAAACAGTGCTGAAGAACCACTATTGTTCAACCACAGTGGTACATCACAGAAGCAGCATGAATC

TGACGGCGAGCTTGAGAGGATACTATCAGACACCACCGTGCCATTCTTCAGCCGTATCAGCTCCGCCACATGGATTGAGC

TCAAACTCCTTTTCTTGTTGGCTGCACCAGCTGTTTTTGTTTATCTTATTAATTATGTTATGTCTATGTCCACACAAATC

TTCTCCGGCCACCTTGGTAATCTTGAACTTGCCGCGGCGTCTCTTGGAAACACCGGGATTCAAATCTTTGCTTATGGTCT

CATGCTGGGTATGGGAAGTGCAGTTGAGACACTATGTGGACAAGCATATGGAGCAGAAAAATATGACATGCTAGGAACAT

ATTTACAAAGATCAACAATTCTTCTAACAATAACCGGTTTTTTCCTCACAGTTATTTACGTATTAAGCGAACCAATCCTA

GTATTCATTGGACAATCACCAAGAATAGCTTCTGCAGCAGCACTTTTTGTGTATGGACTTATCCCACAAATATTTGCTTA

TGCAGTAAATTTTCCAATCCAAAAATTTCTTCAAGCACAAAGTATAGTTCTACCAAGTGCATACATTTCAGCAGGAACTT

TAGTTTTTCATCTAATATTGAGTTGGGTTGTTGTGTTTAAGATTGGTTTGGGACTATTGGGAGCTTCATTGGTTTTGAGT

TTTTCTTGGTGGGTTATTGTTGTGGCACAGTTTATTTATATTTTGAAGAGTGAGAAGTGTAAGAGGACTTGGAATGGGTT

TACTTGGGAGGCGTTTTCAGGGTTGCCGGAGTTTTTTAAATTGTCAGCGGCGTCGGCGGTTATGCTTTGTTTGGAGTCTT

GGTATTTTCAGATTTTGGTTTTGCTTGCTGGACTTCTTCCTGAACCTGAGTTGGCTCTTGATTCTCTTTCTATTTGCACC

ACAGTTTCTGGATGGGTGTTCATGATCTCAGTTGGATTCAACGCAGCTGCAAGTGTGAGAGTGAGCAATGAACTAGGAGC

AAGAAATCCAAAATCAGCATCATTTTCAGTGAAAGTGGTGACATTGATATCATTTCTAATATCAGTTATAGCAGCACTGA

TTGTGCTTGCATTGAGAGATGTTATTAGCTATGTGTTCACTGAGGGTGAAGTGGTTGCTGCTGCTGTCTCAGATCTTTGT

CCACTTCTCTCTCTTTCTCTTGTCCTCAATGGCATTCAGCCAGTCCTATCTGGGGTGGCTGTTGGGTGTGGATGGCAAGC

TTTTGTTGCTTATGTGAATGTGGGTTGTTATTATATAATTGGTATACCATTGGGGGCGGTTCTTGGCTTTTATTTCAATT

TTGGTGCTAAGGGAATATGGCTAGGAATGCTTGGTGGAACCACCATGCAGACAATTATTCTAATGTGGGTCACATTTAGA

ACGGATTGGAACAAAGAGGTGAAAGAAGCAGCCAAGAGGTTGAACAAATGGGAGGATAAGAAAAAGGAGCCGCTTCTAAA

CTAG

>MsMATE78

ATGGGCAGCATCAATAGAGAGAATGATACTGATCACAATCTTATCCAATCTCTCTTGTCAAAAGAAATATCGTCCATTAA

CGAACATCAACATGAGGATGAAGATGAACAAGAATTTGGCAAGAAACTATGGATTGAAACGAAGAAACTATGGCACATAG

TTGGTCCCTCTATTTTCAGCCGTGTTGCATCTTTCACCATGAACGTCGTTACTCAAGCCTTTGCCGGTCACCTTGGCGAC

GTTCAACTCGCTTCCATTTCCATCGCCAATACCGTCATCGTTGGCTTCAATTTCGGCCTCCTTTTGGGGATGGCAAGCGC

ATTGGAGACACTTTGTGGGCAAGCTTTTGGGGCAAAGAAACATAACCTATTAGGAATATACCTACAAAGATCTTGGATTG

TTCTGTTCCTTTGTTGTTTTTTACTGTTGCCATTTTACATCTTTGCCACTCCGATTCTCAAACTTCTAGGACAACCTGAT

GACGTGGCAGAGTGGAGTGGTGTTGTGGCTATCTGGCTTATACCTTTGCATTTCAGTTTTGCATTTCAGTTTCCTCTTCA

GAGGTTTCTACAATGCCAGCTCAAAACTGGTGTCATTGCTTGGGTTTCTTTAGTTGGTTTGGTGGTAAATGTGGTACTTA

GTTGGCTGTTGATTTTTGTTTGGGATTTTGGACTTATTGGTGCTGCTATTGCTTTGGATGTTTCTTGGTGGATTTTGGTA

TTTGGGATGTTGGCTTACACTGTTTGTGGTGGGTGTCCTTTAACTTGGACTGGTTTTTCAATTGAAGCCTTTTCTGGTCT

TTGGGATTTCTTCAAACTCTCTTTTGCTTCTGGGGTCATGCTCTGTAGAGAATTGAAGATATCAAAGATAAAACATATGC

ATTGGTATGTATGTCGGAGTCAACTAGGTCATGTATTGAGGGACGGAGGGAGTATCATCTATGGTTGGAGAAGTAATGTT

GGTGATTTGTGCAGTTTAGAGAACTGGTATTACAGGATACTGCTACTTATGACTGGCCAGTTGGAGAATGCGACTGTTGC

TGTTGATGCCTTGTCTGTATGTATGACTATCAATGGGTGGGAAATGATGATTCCCCTTGCTTTCTTTGCTGGTACCGGGG

TAAGAGTAGCAAACGAACTAGGAGCAGGAAAAGGGAAATCAGCAAAATTTGCAATGCAAGTATCAGTGGCACAATCAACA

GTGATTGGGTTCATATTTTGCATTCTGATAATGATATTCCATAGACAATTTGCATACATTTTCACCACCAGCCCTCCTGT

GCTTGAAGCTGTTAACGATATGTCAATCCTCTTGGCTGTCACAATTCTTCTCAATAGTGTTCAACCTATTCTCTCTGGAG

TAGCTGTTGGTTCAGGATGGCAAGTATTTGTAGCATATGTAAATATAGGATGCTACTATCTAATTGGACTCCCACTTGGA

ATTCTCATGGGATGGGTCTTTAACACTGGTGTTGAAGGTATATGGGGTGGGATGATATTTGGTGGTACAGCAATCCAGAC

GTTGATACTTATCATAGTAACAGCACGATGTGATTGGGAAAATGAGGCAGAGAAGGCTAGGTCACGTGTAAACAAGTGGT

CTGCCACAAAACCTGATGACCAACTGCAGATTACCGAGTAA

>MsMATE79

ATGGGCAGCATCAATAGAGAGAATGATACTGATCACAATCTTATCCAATCTCTCTTGTCAAAAGAAATATCGTCCATTAA

CGAACATCAACATGAGGATGAAGATGAACAAGAATTTGGCAAGAAACTATGGATTGAAACGAAGAAACTATGGCACATAG

TTGGTCCCTCTATTTTCAGCCGTGTTGCATCTTTCACCATGAACGTCGTTACTCAAGCCTTTGCCGGTCACCTTGGCGAC

GTTCAACTCGCTTCCATTTCCATCGCCAATACCGTCATCGTTGGCTTCAATTTCGGCCTCCTTTTGGGGATGGCAAGCGC

ATTGGAGACACTTTGTGGGCAAGCTTTTGGGGCAAAGAAACATAACCTATTAGGAATATACCTACAAAGATCTTGGATTG

TTCTGTTCCTTTGTTGTTTTTTACTGTTGCCATTTTATATCTTTTCCACTCCGATTCTCAAACTTCTAGGACAACCTGAT

GACGTGGCAGAGTGGAGTGGTGTTGTGGCTATCTGGCTTATACCTTTGCATTTCAGTTTTGCATTTCAGTTTCCTCTTCA

GAGGTTTCTACAATGCCAGCTCAAAACTGGTGTCATTGCTTGGGTTTCTTTGGTTGGTTTGGTGGTAAATGTGGTACTTA

GTTGGCTGTTGATTTTTGTTTGGGATTTTGGACTTATTGGTGCTGCTATTGCTTTGGATGTTTCTTGGTGGATTTTGGTT

TTTGGGATGTTGGCTTATACTGTTTGTGGTGGGTGTCCTTTAACTTGGACTGGTTTTTCAATTGAAGCCTTTTCCGGTCT

TTGGGATTTCTTCAAACTCTCTTTTGCTTCTGGGGTCATGCTCTGTTTAGAGAACTGGTATTACAGGATACTGCTACTTA

TGACTGGCCAGTTGGAGAATGCGACTGTTGCTGTTGATGCCTTGTCTGTATGTATGACTATCAATGGGTGGGAAATGATG

ATTCCCCTTGCTTTCTTTGCTGGTACCGGGGTAAGAGTAGCAAACGAACTAGGAGCAGGAAAAGGGAAATCAGCAAAATT

TGCAATGCAAGTATCAGTGGCACAATCAACAGTGATTGGGTTCATATTTTGCATTCTGATAATGATATTCCATAGACAAA

TTGCATACATTTTCACCACCAGCCCTCCTGTGCTTGAAGCTGTTAACGATATGTCAATCCTCTTGGCTGTCACAATTCTT

CTCAATAGTGTTCAACCTATTCTCTCTGGAGTAGCTGTTGGTTCAGGATGGCAAGTATTTGTAGCATATGTAAATATAGG

ATGCTACTATCTAATTGGACTCCCACTTGGAATTCTTATGGGATGGGTCTTTAACACTGGTGTTGAAGGTATATGGGGTG

GTATGATATTTGGTGGTACAGCAATCCAAACGTTGATACTTATCATAGTAACAGCACGATGTGATTGGGAAAATGAGGCA

GAGAAGGCTAGGTCACGTGTAAACAAGTGGTCTGCCACAAAACCTGATGACCAACTGCAGATTACCGAGTAA

>MsMATE80

ATGCCAAGCAAGGAACTAGCTTCACCATTAGGAGAAACAAAGGTTCCATTATTATCACCAAATCATCTTTCAACAACA

AATGAAGAAGAAGAACCACAAGATCTTACAAGAAAGGTTTGGATTGAATCAAAGAAGCTATGGCATATAGTTGGCCCT

GCAATTTTCAGCCGTATTGCATCTTATATGATGTTGGTCATCACTCAAGCCTTTGCTGGTCATCTTGGTGACCTTGAA

CTTGCAGCTATCTCCATAGCCAATAATGTTGTTGTTGGCTTTGATTTTGGCCTCTTGTTAGGAATGGCAAGTGCATTA

GAAACATTATGTGGACAAGCATTTGGAGCAAAACAATATTACATGTTAGGAGTATACATGCAACGATCATGGATAGTA

TTATTCATATGTTGTATTTTTCTCTTACCAATCTACCTTTTTGCAACGCCAGTGTTAAGACTACTGGGCCAACCAGAG

GATTTAGCTGTATTATCAGGACAAGTTTCAGTGTGGTTGATACCATTACATTTTGCATTTGCATTTCAATTTCCTCTC

AACAGATTCTTACAAAGCCAGCTCAAAACAGCTGCTATAGCTTGGGTGTCTTTGTTTGCACTTTTGGTTCATGTTTTT

GTGAGCTGGTTGTTTGTCTTTAAGTTTCAATTTGGTGTAATTGGAACTGCTGCTACTTTGAATTTCTCTTGGTGGGCC

CTTACTGTAGGGCTTTTTTGTTACACTGTTTATGGTGGTTGTCCTTTGACTTGGAATGGGTTTTCTATGGAGGCTTTT

TCTGGGCTTTGGGAATTTGTTAAACTTTCTGCTGCTTCTGGTGTTATGCTATGTTTGGAGAATTGGTATTATAGAATT

CTGATATTAATGACCGGGAATCTTCCAAATGCTGAGATTGCAGTAGATGCTCTATCCATATGCATGACCATAAATGGG

TTGGAAATGATGATTCCATTGGCATTCTTTGCTGCAACAGGAGTGAGGGTTGCAAACGAGCTTGGAGCTGGGAATGGG

AAAGGAGCAAAATTTGCTACTATAGTGTCTGTGTTGACATCATTAATAATTGGACTTTTCTTCTGGATGTTGATTATG

ATATTTCATGATAAATTTGGCTACATATTCTCTACTAGTAAACCTGTTCTTGATGAAGTAAGCAAGCTTTCACTTCTA

TTAGCATTCACAATTCTTCTCAATAGTGTCCAACCAGTTCTATCAGGAGTGGCAGTTGGATCAGGGTGGCAATCATAT

GTTGCATATATAAATTTGGGGTGCTACTATATGATTGGGGTTCCTCTTGGATTTTTAATGGGTTGGTACTTTGATCAA

GGTGTTATGGGAATTTGGGCAGGGATGATTTTTGGAGGAACAGCAACTCAAACATTAATTTTGTGTTTGATTACACTT

CGATGTGACTGGGATAAAGAGGCAGAGAAAGCAAAATTACATATAACAAAGTGGTCAAATCGAAAACAACAACTAAGC

TAG

>MsMATE81

ATGCCAAGCAAGGAACTAGCTTCACCATTAGGAGAAACAAAGGTTCCATTATTATCACCAAATCATCTTTCAACAACAAA

TGAAGAAGAAGAACCACAAGATCTTACAAGAAAGGTTTGGATTGAATCAAAGAAGCTATGGCATATAGTTGGCCCTGCAA

TTTTCAGCCGTATTGCATCTTATATGATGTTGGTCATCACTCAAGCCTTTGCTGGTCATCTTGGTGACCTTGAACTTGCA

GCTATCTCCATAGCCAATAATGTTGTTGTTGGCTTTGATTTTGGCCTCTTGTTAGGAATGGCAAGTGCATTAGAAACATT

ATGTGGGCAAGCATTTGGGGCAAAACAATATTACATGTTAGGAGTATACATGCAACGATCATGGATAGTACTATTCATAT

GTTGTATTTTTCTCTTACCAATCTACCTTTTTGCAACGCCAGTGTTAAGACTATTGGGCCAACCAGAAGATTTAGCAGTA

TTATCAGGACAAGTTTCAATGTGGTTGATACCATTACATTTTGCATTTGCATTTCAATTTCCTCTCAATAGATTCTTACA

AAGCCAGCTCAAAACAGCTGCTATAGCTTGGGTGTCTTTGTTTGCACTTTTGGTTCATGTTTTTGTGAGCTGGTTGTTTG

TGTTTAAGTTTCAATTTGGTGTAATTGGAACTGCTGCTACTTTGAATTTCTCTTGGTGGGCCCTTACTGTAGGGCTTTTT

TGTTACACTGTTTATGGCGGTTGTCCTTTGACTTGGAATGGGTTTTCTATGGAGGCTTTTTCTGGGCTTTGGGAATTTGT

TAAACTTTCTGCTGCTTCTGGTGTTATGTTATGTTTGGAGAATTGGTATTATAGAATTCTGATATTAATGACCGGGAATC

TTCCAAATGCAGAGATTGCAGTAGATGCTCTATCCATATGCATGACCATAAATGGATTGGAAATGATGATTCCATTGGCA

TTCTTTGCTGCAACAGGAGTGAGGGTTGCGAACGAGCTTGGAGCTGGAAATGGGAAAGGAGCAAAATTTGCTACTATAGT

GTCTGTGTTGACATCATTAATAATTGGACTTTTCTTCTGGATGTTGATTATGATATTTCATGATAAATTTGGCTACATAT

TCTCTACTAGTAAACCTGTTCTTGATGAAGTAAGCAAGCTTTCACTTCTATTAGCATTCACAATTCTTCTCAATAGTGTC

CAACCAGTTCTATCAGGAGTGGCAGTTGGATCAGGGTGGCAATCATATGTTGCATATATAAATTTGGGGTGCTACTATAT

GATTGGGGTTCCTCTTGGATTTTTAATGGGTTGGTACTTTGATCAAGGTGTTATGGGAATTTGGGCAGGGATGATTTTTG

GTGGAACAGCAACTCAAACATTAATTTTGTGTTTGATTACACTTCGATGTGACTGGGATAAAGAGGTAAATTTAACTAGC

TATCTATCTATTTATTCAACTTTTTAA

>MsMATE82

ATGCCAAGCAAGGAACTAGCTTCACCATTAGGAGAAACAAAGGTTCCATTATTATCACCAAATCATCTTTCAACAACAAA

TGAAGAAGAAGAACCACAAGATCTTACAAGAAAGGTTTGGATTGAATCAAAGAAGCTATGGCATATAGTTGGCCCTGCAA

TTTTCAGCCGTATTGCATCTTATATGATGTTGGTCATCACTCAAGCCTTTGCTGGTCATCTTGGTGACCTTGAACTTGCA

GCTATCTCCATAGCCAATAATGTTGTTGTTGGCTTTGATTTTGGCCTCTTGTTAGGAATGGCAAGTGCATTAGAAACATT

ATGTGGACAAGCATTTGGAGCAAAACAATATTACATGTTAGGAGTATACATGCAACGATCATGGATAGTACTATTCATAT

GTTGTATTTTTCTCTTACCAATCTACCTTTTTGCAACACCAGTGTTAAGACTATTGGGCCAACCAGAAGATTTAGCAGTA

TTATCAGGACAAGTTTCAATGTGGTTGATACCATTACATTTTGCATTTGCATTTCAATTTCCTCTCAACAGATTCTTACA

AAGCCAGCTCAAAACAGCTGCTATAGCTTGGGTGTCTTTGTTTGCACTTTTGGTTCATGTTTTTGTGAGCTGGTTGTTTG

TCTTTAAGTTTCAATTTGGTGTAATTGGAACTGCTGCTACTTTGAATTTCTCTTGGTGGGCCCTTACTGTAGGGCTTTTT

TGTTACACTGTTTATGGCGGTTGTCCTTTGACTTGGAATGGGTTTTCTATGGAGGCTTTTTCTGGGCTTTGGGAATTTGT

TAAACTTTCTGCTGCTTCTGGTGTTATGCTATGTTTGGAGAATTGGTATTATAGAATTCTGATATTAATGACCGGGAATC

TTCCAAATGCAGAGATTGCAGTAGATGCTCTATCCATATGCATGACAATAAATGGATTGGAAATGATGATTCCATTGGCA

TTCTTTGCTGCAACAGGAGTGAGGGTTGCGAACGAGCTTGGAGCTGGAAATGGGAAAGGAGCAAAATTTGCTACTATAGT

GTCTGTGTTGACATCATTAATAATTGGACTTTTCTTCTGGATGTTGATTATGATATTTCATGATAAATTTGGCTACATAT

TCTCTACTAGTAAACCTGTTCTTGATGAAGTAAGCAAGCTTTCACTTCTATTAGCATTCACAATTCTTCTCAATAGTGTC

CAACCAGTTCTATCAGGTATCACCTTAACAATTAACATTTCAAATTCAAATATTTAA

>MsMATE83

ATGCCAAGCAAGGAACTAGCTTCACCATTAGGAGAAACAAAGGTTCCATTATTATCACCAAATCATCTTTCAACAACAAA

TGAAGAAGAAGAACCACAAGATCTTACAAGAAAGGTTTGGATTGAATCAAAGAAGCTATGGCATATAGTTGGCCCTGCAA

TTTTCAGCCGTATTGCATCTTATATGATGTTGGTCATCACTCAAGCCTTTGCTGGTCATCTTGGTGACCTTGAACTTGCA

GCTATCTCCATAGCCAATAATGTTGTTGTTGGCTTTGATTTTGGCCTCTTGTTAGGAATGGCAAGTGCATTAGAAACATT

ATGTGGGCAAGCATTTGGGGCAAAACAATATTACATGTTAGGAGTATACATGCAACGATCATGGATAGTACTATTCATAT

GTTGTATTTTTCTCTTACCAATCTACCTTTTTGCAACGCCAGTGTTAAGACTATTGGGCCAACCAGAAGATTTAGCAGTA

TTATCAGGACAAGTTTCAATGTGGTTGATACCATTACATTTTGCATTTGCATTTCAATTTCCTCTCAATAGATTCTTACA

AAGCCAGCTCAAAACAGCTGCTATAGCTTGGGTGTCTTTGTTTGCACTTTTGGTTCATGTTTTTGTGAGCTGGTTGTTTG

TGTTTAAGTTTCAATTTGGTGTAATTGGAACTGCTGCTACTTTGAATTTCTCTTGGTGGGCCCTTACTGTAGGGCTTTTT

TGTTACACTGTTTATGGCGGTTGTCCTTTGACTTGGAATGGGTTTTCTATGGAGGCTTTTTCTGGGCTTTGGGAATTTGT

TAAACTTTCTGCTGCTTCTGGTGTTATGTTATGTTTGGAGAATTGGTATTATAGAATTCTGATATTAATGACCGGGAATC

TTCCAAATGCAGAGATTGCAGTAGATGCTCTATCCATATGCATGACCATAAATGGATTGGAAATGATGATTCCATTGGCA

TTCTTTGCTGCAACAGGAGTGAGGGTTGCGAACGAGCTTGGAGCTGGAAATGGGAAAGGAGCAAAATTTGCTACTATAGT

GTCTGTGTTGACATCATTAATAATTGGACTTTTCTTCTGGATGTTGATTATGATATTTCATGATAAATTTGGCTACATAT

TCTCTACTAGTAAACCTGTTCTTGATGAAGTAAGCAAGCTTTCACTTCTATTAGCATTCACAATTCTTCTCAATAGTGTC

CAACCAGTTCTATCAGGAGTGGCAGTTGGATCAGGGTGGCAATCATATGTTGCATATATAAATTTGGGGTGCTACTATAT

GATTGGGGTTCCTCTTGGATTTTTAATGGGTTGGTACTTTGATCAAGGTGTTATGGGAATTTGGGCAGGGATGATTTTTG

GTGGAACAGCAACTCAAACATTAATTTTGTGTTTGATTACACTTCGATGTGACTGGGATAAAGAGGCAGAGAAAGCAAAA

TTACATATTACAAAGTGGTCAAATCGAAAACAACAACTAAGCTAG

>MsMATE84

ATGGGATCAGAAGAACTAAACAAGAACCTTCTGCAGCATCAAAACCCTTCAGAGGAGGAAGAAGAGCCACTAAGGAAAAG

GGTATGGGAAGAAAGCAAGAAGCTGTGGATAGTAGCAGGCCCTGCCATATTCAATAGGTTTTCAACATTTGGTATCATGG

TAGTTGCTCAATCCTTCATTGGTCATATTGGTTCAACCGAATTAGCTGCTTATGCCCTTGTTATGACTGTCCTAGTCAGG

TTTGCAAATGGTATTCTGTTGGGTATGGCAAGTGCATTAGAAACACTATGTGGACAAGCATATGGAGCAAAACAATATGA

TATGCTTGGAGTGTATCTTCAAAGATCATGGATAGTTATATTCTTAACCTCAATTCTTCTTCTTCCAATTTACATTTTTA

CCAAACCAATTTTAGTGGCACTTGGCCAAGATGAAAATATTGCACAAGTTGCTGGAAGCATTTCAATATGGTCAATAGGT

ATAGTATTTGCTTTCTCTGCATCATTCACTTGCCAAATGTTCCTACAAGCACAAAGCAAGAACAAGATTATTGCTTACCT

TGCAGCAGTTTCAATTTCAATACATGTTTTTATGTCATGGCTTTTAACTGTTAAGTTCAAGTTTGGCCTTAATGGTGCAA

TGACATCAATCCTTTTGGCTTATTGGATGCCAAATTTGGGGCAGCTTGTTTTTATCATGACAAAATGTCCTGATACATGG

AAGGGTTTCTCATTTTTGGCCTTTAAAGATCTTTGGCCTGTTATCAAGCTTTCTTTATCTTCTGGAGCTATGTTATGTCT

TGAAATATGGTACAACACAGTTTTGATTCTTCTAACAGGGAACATGGAAAATGCTGAGATTTCCATTGATGCTCTTGCTA

TATGCCTCAACATCAATGGGTGGGAAATGATGATAGCCCTTGGTTTCTTCGCTGCAGCTAGTGTTAGGGTGTCAAATGAA

CTAGGCAGAGGGAGTTCCAAGGCAGCAAAATTTTCCATTGTCATAACAGTGCTCACATCATTTTCCATTGGATTTGTGCT

ATTCTTGATCTTTCTGTTCCTAAGAGGAAGACTTGCTTACATTTTCACCCCAAATCCAGATGTGGCAAATGCAGTTGGAG

ATCTATCACCTTTGTTATCATTCTCCATATTGATGAACAGTGTCCAACCTGTGCTCTCTGGTGTTTCTGTTGGAGCTGGC

TGGCAAAGTGTTGTAGCATATGTCAACATAGGCTGCTATTACCTAATTGGTATTCCTATCGGAGTTGTGCTTGGTAATCT

TCTTCATTTGCAAGTTAAGGGTGTTTGGATTGGAATGTTGTTTGGAACTTTTGTTCAAACCATAATGCTTATGATAATCA

CATTTAAGACTGACTGGGACAAGCAGGTTGAGATTGCTAGAAATCGTGTTAATAAATGGGCTGTAGTGGAGAATGATGAA

TCAAACAACACATCAAGGATATCCAACTAA

>MsMATE85

ATGGAGGGAGATCATGATCCAAAGAAGAAGTTGCTAGATGAAGAAGAAGAAGAATTGTCATTGGTGAAGAAGATGTGGAA

AGAGAGCAAGCTAATGTGGGTGGTTGCAGGTCCAGCCATATTCACAAGATTTTCATCTTTTGGTGTCCAAATTATCACTC

AATCATTTGTTGGTCATATTGGTTCTACTGAACTTGCTGCATATTCTCTAGTATTCACTGTTCTTGTTAGATTCGTCAAT

GGCATTCTGTTAGGAATGGCTAGTGCATTGGCAACACTATGTGGACAAGCATACGGTGCAAAAGAATATGGAATGATGGG

NNNNNNTCTTCAAAGATCATGGATAGTTTTATCTTTAACTGCACTCATTCTTCTTCCACTATTCATCTTCACAACTCCAA

TTTTAATTATCCTAGGCCAAGATGAAACCATAGCACAAGTAGCAGGAACCATTGGTTATTGGTCAATTCCAATTTTGTTT

GCTTTTATTGCCTCATTCACTACCCAAACATTCCTTCAGTCACAAAGCAGGAACATTATTATTGCATACTTGGCAGCTTT

CTCAATTTCTGTTCATGTATTACTCTCTTGGCTTTTAACAATGAAAATCAAGCTTGGGATTGTTGGTGCAATGATTTCAA

TAAGTTTGGCCTTATGGATTCCAAATATTGGTCAACTTATATTTATTACATGTGGTTGGTGTTCTGATACTTGGAAAGGT

TTCTCATTTTTAGCTTTACAAGATCTTTGGCCTGTTGTCAAGCTTTCCCTTTCTTCAGGTTTCATGTTATGTCTTGAGCT

ATGGTACAACACGGTATTGATTCTTTTAACAGGCAACATGGAAAATGCAGAGATTCAAATTGATGCTCTATCCATATGTC

TCAACATCAATGGATGGGAAATGATGATATCACTTGGTTTCATGGCTGCAGCAAGTGTTAGAGTGGCAAATGAGCTTGGA

AAAGGAAGTGCCAAAGATGCAAAGTTTGCAGTGAACATGATAGTCCTTACATCATTCACAATTGGGTTCCTTCTGTTTTT

ATTTTTCTTATTTTTTAGAGAAAGACTTGCTTATATTTTTACCACAAACAAGGACGTGGCCTCAGCTGTTGGTGATTTGT

CACCTCTATTGGCAGTTTCTATACTACTAAACAGTGTTCAACCTGTACTATCAGGAGTTGCTATTGGAGCAGGGTGGCAA

AGTATTGTAGCATATGTGAACCTAGGTTGTTATTACATCATAGGTATTCCTGTTGGGATTGTTCTTGGTAAAGTTTACCA

TTTGCAAGTCAAGGGTATATGGATTGGTATGTTGTTTGGAACACTAATGCAAACTATTATACTACTCATAATCAGCTACA

AAACTGATTGGGACAAACAGGTAACTATTGCTCGGAATCGTATTAATAAGTGGTCTAAGGTGGACCCTGATCATGAAACA

GTTGCATCAGATAATTAG

>MsMATE86

ATGGAAAGAGATCTAAAACAGAATCTACTACTGAAAAAATCAGAACAAGAAGAAAATGAGTTATCACTAGGAAAAAGGGT

TTGGAATGAAACAAAGCTTATGTGGGTGGTTGCAGCACCAGCAATATTCACAAGATTTTCAACTTTTGGTATTCAAATTA

TTAGTCAAGCTTTTGTTGGTCATATTGGTTCAAGAGAACTCGCTGCTTTTGCTCTTGTTTTCACTGTTCTCATTAGGTTC

GCCAATGGTATTCTCTTGGGAATGGCAACTGCATTGGCGACCCTTTGTGGACAAGCATATGGTGCAAAAGAATATGGAAT

GATGGGAGTATATCTTCAAAGATCATGGATAGTTTTATTCCTAACTGCACTTGTTCTTCTTCCTGTGTTTGTCTTCACAA

CCCCAATTTTGACTCTCTTAGGCCAAGATGAAAGCATATCAGAAGTTGCAGGAAACATCTCTCTTTGGTCAATTCCAATT

ATGTTTGCTTTTATTGTCTCATTCACTTGCCAAACATTCCTTCAATCACAAAGCAAGAACACCATCATTGCATTCTTGGC

AGCTTTTTCCATAATCATTCATGCATTCCTCTCTTGGCTTTTGACAATGAAATACGAATTTGGGATTGCTGGTGCAATGA

TTTCAACTATTTTGGCATATTGGATTCCCAACATTGGTCAACTCATATTTGTTACATGTGGTTGGTGTCCTGAGACTTGG

CAAGGTTTCTCATTTTTAGCATTCAAAGATCTTTGGCCTGTCGTCAAACTTTCCCTTTCAGCTGGTGCAATGTTGTGTCT

TGAGCTATGGTACAACACAATATTGGTTCTTTTGACAGGGAACATGAAAAATGCTGAGGTTGAAATTGATGCACTATCTA

TATGTCTCAACATCAATGGATGGGAAATGATGATATCACTTGGTTTCATGGCTGCAGCAAGTGTTCGAGTATCAAATGAG

CTTGGTAAAGGAAGCGCGAAAGCCGCGAAGTTCTCGATCGTTGTGACAGTGCTTACGTCGTTGGCCATCGGATCCTTTCT

GTTTTTGTTCTTCTTATTTTTTAGAGAAAGACTTGCTTATATTTTCACCTCGAATAAAGAGGTGGCCGCAGCTGTCGGAG

AATTATCGCCTTTGTTATCGATCTCTATACTATTAAACAGTGTTCAACCAGTACTCTCAGGAGTGGCAATTGGAGCAGGG

TGGCAAAGCACAGTAGCATATGTGAACATAGGTTGTTATTACATCATAGGTATTCCTGTTGGGGTTGTTCTTGGTAATGT

TATCCATTGGCAAGTCAAGGGTATCTGGATGGGAATGTTGTTTGGAACATTGATTCAAACTATAGTTCTACTTATAATCA

CCTACAAAACCAACTGGGATGAGCAGGTGACTGTAGCTCGCAAGCGTGTAAACAGGTGGTCCAACGTGGACAGTACTGAT

CAAGAAACAAAAACTAACTTAATTGAAAAATAG

>MsMATE87

ATGGAAAGAGATCTAAAACAGAATCTACTACTGAAAAAATCAGAACAAGAAGAAAATGAACAAGAAGAGTTATCGCTAGG

AAAAAGGGTTTGGAATGAAACAAAGCTTATGTGGGTGGTTGCAGCACCAGCAATATTCACAAGATTTTCAACTTTTGGTA

TTCAAATTATTAGTCAAGCTTTTGTTGGTCATATTGGTTCAAGAGAACTCGCTGCTTTTGCTCTTGTTTTCACTGTTCTC

ATTAGGTTCGCCAATGGTATTCTCTTGGGAATGGCAACTGCATTGGCGACCCTTTGTGGACAAGCATATGGTGCAAAAGA

ATATGGAATGATGGGAGTATATCTTCAAAGATCATGGATAGTTTTATTCCTAACTGCACTTGTTCTTCTTCCTGTGTTTG

TCTTCACAACCCCAATTTTGACTCTCTTAGGCCAAGATGAAAGCATATCAGAAGTTGCAGGAAACATCTCTCTTTGGTCA

ATTCCAATTATGTTTGCTTTTATTGTCTCATTCACTTGCCAAACATTCCTTCAATCACAAAGCAAGAACACCATCATTGC

ATTCTTGGCAGCTTTTTCCATAATTATTCATGCATTCCTCTCTTGGCTTTTGACAATGAAATACGAATTTGGGATTGCTG

GTGCAATGATTTCAACTATTTTGGCATATTGGATTCCCAACATTGGTCAACTCATATTTGTTACATGTGGTTGGTGTCCT

GAGACTTGGCAAGGTTTCTCATTTTTAGCATTCAAAGATCTTTGGCCTGTCGTCAAACTTTCCCTTTCAGCTGGTGCAAT

GTTGTGTCTTGAGCTATGGTACAACACAATATTGGTTCTTTTGACAGGGAACATGAAAAATGCTGAGGTTGAAATTGATG

CACTATCTATATGTCTCAACATCAATGGATGGGAAATGATGATATCACTTGGTTTCATGGCTGCAGCAAGTGTTCGAGTA

TCAAATGAGCTTGGTAAAGGAAGCGCGAAAGCCGCGAAGTTCTCGATCGTTGTGACAGTGCTTACGTCGTTGGCCATCGG

ATCCTTTCTGTTTTTGTTCTTCTTATTTTTTAGAGAAAGACTTGCTTATATTTTCACCTCGAATAAAGAGGTGGCCGCAG

CTGTCGGAGAATTATCGCCTTTGTTATCGATCTCTATACTATTAAACAGTGTTCAACCAGTACTCTCAGGAGTGGCAATT

GGAGCAGGGTGGCAAAGCACAGTAGCATATGTGAACATAGGTTGTTATTACATCATAGGTATTCCTGTTGGGGTTGTTCT

TGGTAATGTTATCCATTGGCAAGTCAAGGGTATCTGGATGGGAATGTTGTTTGGAACATTGATTCAAACTATAGTTCTAC

TTATAATCACCTACAAAACCAACTGGGATGAGCAGGTGACTGTAGCTCGCAAGCGTGTAAACAGGTGGTCCAAGGTGGAC

AGTACTGATCAAGAAACAAAAACTAACTTAATTGAAAAATAG

>MsMATE88

ATGAAACAAGAAGAGTTATCGCTAGGAAAAAGGGTTTGGAATGAAACAAAGCTTATGTGGGTGGTTGCAGCACCAGCAAT

ATTCACAAGATTTTCAACTTTTGGTATTCAAATTATTAGTCAAGCTTTTGTTGGTCATATTGGTTCAAGAGAACTCGCTG

CTTTTGCTCTTGTTTTCACTGTTCTCATTAGGTTCGCCAATGGTATTCTCTTGGGAATGGCAACTGCATTGGCGACCCTT

TGTGGACAAGCATATGGTGCAAAAGAATATGGAATGATGGGAGTATATCTTCAAAGATCATGGATAGTTTTATTCCTAAC

TGCACTTGTTCTTCTTCCTGTGTTTGTCTTCACAACCCCAATTTTGACTCTCTTAGGCCAAGATGAAAGCATATCAGAAG

TTGCAGGAAACATCTCTCTTTGGTCAATTCCAATTATGTTTGCTTTTATTGTCTCATTCACTTGCCAAACATTCCTTCAA

TCACAAAGCAAGAACACCATCATTGCATTCTTGGCAGCTTTTTCCATAATTATTCATGCATTCCTCTCTTGGCTTTTGAC

AATGAAATACGAATTTGGGATTGCTGGTGCAATGATTTCAACTATTTTGGCATATTGGATTCCCAACATTGGTCAACTCA

TATTTGTTACATGTGGTTGGTGTCCTGAGACTTGGCAAGGTTTCTCATTTTTAGCATTCAAAGATCTTTGGCCTGTCGTC

AAACTTTCCCTTTCAGCTGGTGCAATGTTGTGTCTTGAGCTATGGTACAACACAATATTGGTTCTTTTGACAGGGAACAT

GAAAAATGCTGAGGTTGAAATTGATGCACTATCTATATGTCTCAACATCAATGGATGGGAAATGATGATATCACTTGGTT

TCATGGCTGCAGCAAGTGTTCGAGTATCAAATGAGCTTGGTAAAGGAAGCGCGAAAGCCGCGAAGTTCTCGATCGTTGTG

ACAGTGCTTACGTCGTTGGCCATCGGATCCTTTCTGTTTTTGTTCTTCTTATTTTTTAGAGAAAGACTTGCTTATATTTT

CACCTCGAATAAAGAGGTGGCCGCAGCTGTCGGAGAATTATCGCCTTTGTTATCGATCTCTATACTATTAAACAGTGTTC

AACCAGTACTCTCAGGAGTGGCAATTGGAGCAGGGTGGCAAAGCACAGTAGCATATGTGAACATAGGTTGTTATTACATC

ATAGGTATTCCTGTTGGGGTTGTTCTTGGTAATGTTATCCATTGGCAAGTCAAGGGTATCTGGATGGGAATGTTGTTTGG

AACATTGATTCAAACTATAGTTCTACTTATAATCACCTACAAAACCAACTGGGATGAGCAGGTGACTGTAGCTCGCAAGC

GTGTAAACAGGTGGTCCAAGGTGGACAGTACTGATCAAGAAACAAAAACTAACTTAATTGAAAAATAG

**The protein sequences of 88 MsMATEs**

>MsMATE01

MEDNGISNNAVKNKWTMPLSVFFKDASLVFKMDSLAKEILGIAFPSALAVAADPIASLIDTAFIGHLGPVELAAAGVSIA

VFNQASRITIFPLVSITTSFVAEEDTMDRINSKAAEKQFNEGIKAKSNEVMPDDHLLQDIEAGAIKQDGTLKNETKNGDD

ANSNVSKSSIVTNSGNKSESKPVRKKRHIASASTALLFGTVLGLIQAATLIFAAKPLLGAMGLKYDSPMLVPAVKYLRLR

ALGAPAVLLSLAMQGIFRGFKDTTTPLYVIVSGYALNVAMDPLLIFYFKLGIRGAAISHVLSQYIMASLLLFILMKKVDL

LPPSMKDLQIFRFLKNGGLLLARVIAVTFCVTLSASLAARLGPIPMAAFQTCLQVWMTSSLLADGLAVAIQAILACSFAE

KDYNKVTTAATRTLQMSFVLGVGLSLVVGGGLYFGAGVFSKNVAVIHLIRLGLPPLSLMV

>MsMATE02

MEDNGISNNAVKNKWTMPLSVFFKDASLVFKMDSLAKEILGIAFPSALAVAADPIASLIDTAFIGHLGPVELAAAGVSIA

VFNQASRITIFPLVSITTSFVAEEDTMDRINSKAAEKQFNEGIKAKSNEVMPDDHLLQDIEAGATKQDSTLKNETKNGDD

ANSNVSKSSIVTNSGNKSESKPIRKKRHIASASTALLFGTVLGLIQAATLIFAAKPLLGAMGLKYDSPMLVPAVKYLRLR

ALGAPAVLLSLAMQGIFRGFKDTTTPLYVIVSGYALNVAMDPLLIFYFKLGIRGAAISHVLSQYIMASLLLFILMKKVDL

LPPSMKDLQIFRFLKNGGLLLARVIAVTFCVTLSASLAARLGPIPMAAFQTCLQVWMTSSLLADGLAVAIQAILACSFAE

KDYNKVTTAATRTLQMSFVLGVGLSLVVGGGLYFGAGVFSKNVAVIHLIRLGLPFVAATQPINSLAFVFDGVNYGASDFA

YSAYSLVLVSLASVTSLFFLYKSKGFIGIWIALTIYMSLRMFAGVWRMGTGTGPWRFLRGHSLS

>MsMATE03

MNIKAAENDKSKLTEVTPESDVVQDVEKGTPKESNKAQKESVVGHNETNGTLGNNDKTNGVAVKNNEQEPHLLSSDPRSN

KSKEIVVKKKKRHIASASTALLFGSILGLLQAAILIFGAKPLLYVMGVKHGSPMLKPAVKYLTYRSFGAPAVLLSLAMQG

IFRGFKDTTTPLYVIVAGYSLNVLLEPLLIFKLKMGIKGAAIAHVISQYMMAFTLFFILMKKVYLLPPRIKDLQIFRFLR

NGGLLMTKVIAVTFCVTLAASLAARLGSIPMAAFQPCLQVWLASSLFADGLAIAVQAILAGSFAEKDYNKTTAAATRTLQ

FGFILGAGLSVIVGFGLYFGAGIFTKNLQVIHFIRIGAPIVAATQPINTLAFVFDGVNYGASDFAYASYSLVTVSLLSVG

VEFLLYRSNQFIGIWIALSIYMTLRMLAGVWRMGTGTGPWNYLRG

>MsMATE04

MNENGNANEPNKKWKFPFLVFFNDARLIFKLDALSKEILGIAIPSALAVAADPIASLIDTAFIGHLGPVELAAAGVSIAL

FNQASKITIFPLVSITTSFVAEEDTIKRMNIKAAENDKSKLTEVTPESDVVQDVEKGTPKESNKAQKESVVGHNETNGTV

ANDDKTNGVVAMKNEQEPHLLSSVPRSNKSKKRHIASASTALLFGSILGLLQAAILIFGAKPLLYVMGVKHGSPMLKPAV

KYLTYRSFGAPAVLLSLAMQGIFRGFKDTTTPLYVIVAGYSLNVLLEPLLIFKLKMGIKGAAIAHVISQYMMAFTLFFIL

MKKVYLLPPRIKDLQIFRFLRNGGLLMTKVIAVTFCVTLAASLAARLGSIPMAAFQPCLQVWLASSLFADGLAIAVQAIL

AGSFAEKDYNKTTAAATRTLQFGFILGAGLSVIVGFGLYFGAGIFTKNLQVIHFIRIGAPIVAATQPINTLAFVFDGVNY

GASDFAYASYSLVTVSLLSVGVEFLLYRSNQFIGIWIALSIYMTLRMLAGVWRMGTGTGPWNYLRG

>MsMATE05

MNENGNANEPNKKWKFPFLVFFNDARLIFKLDALSKEILGIAIPSALAVAADPIASLIDTAFIGHLGPVELAAAGVSIAL

FNQASKITIFPLVSITTSFVAEEDTIKRMNIKAAENDKSKLTEVTPESDVVQDVEKGTPKESNKAQKESVVGHNETNGTL

GNNDKTNGVDPRSNKSKEIVVKKKKRHIASASTALLFGSILGLLQAAILIFGAKPLLYVMGVKHGSPMLKPAVKYLTYRS

FGAPAVLLSLAMQGIFRGFKDTTTPLYVIVAGYSLNVLLEPLLIFKLKMGIKGAAIAHVISQYMMAFTLFFILMKKVYLL

PPRIKDLQIFRFLRNGGLLMTKVIAVTFCVTLAASLAARLGSIPMAAFQPCLQVWLASSLFADGLAIAVQAILAGSFAEK

DYNKTTAAATRTLQFGFILGAGLSVIVGFGLYFGAGIFTKNLQVIHFIRIGAPIVAATQPINTLAFVFDGVNYGASDFAY

ASYSLVTVSLLSVGVEFLLYRSNQFIGIWIALSIYMTLRMLAGVWRMGTGTGPWNYLRG

>MsMATE06

MAEKESLFSIGDWMRIPICTFFKDARLVFKLDDLGREILSIALPAAMALTADPIASLVDTAFIGQLGPVELAAVGVSIAL

FNQASRIFIFPLVSVTTSFVAEEDALSDASSQVEENGCLEAATPPDAETKEFLPQKNSVVESFNVVKDDGHKRRKIPSAS

SALYFGGILGLVQATLLISAAKPLLNFMGVTSDSPMLHPAMQYLKLRSLGAPAVLLSLAMQGVFRGFKDTKTPLYATGMY

ALTNFNCYLL

>MsMATE07

MAEKESLFSIGDWMRIPICTFFKDARLVFKLDDLGREILSIALPAAMALTADPIASLVDTAFIGQLGPVELAAVGVSIAL

FNQASRIFIFPLVSVTTSFVAEEDALSDASSQVEENGCLEAATPPDAETKEFLPQKNSVVESFNVVKVDGSKRRQIPSAS

SALYFGGILGLVQATLLISAAKPLLNFMGVTSDSPMLHPAMQYLKLRSLGAPAALLSLAMQGVFRGFKDTKTPLYATVAG

DLTNIALDPLFIFVFRMGVNGAAIAHVISQYLLSAILLWSLNKQVDLIPPSIKHLQFDRFAKNGFLLFMRVIAVTFCVTL

AASLAAHHGATSMAAFQVYLQVSLAVSLLADGLAVAGQAILAGAFANKDYEKASTTATRVLQMGMVLGLALAFILGTGLH

FGAKLFTKDDDVLHLIRVGVPVIYSCSNLERIY

>MsMATE08

MRIPICTFFKDARLVFKLDDLGREILSIALPAAMALTADPIASLVDTAFIGQLGPVELAAVGVSIALFNQASRIFIFPLV

SVTTSFVAEEDALSDASSQVEENGCLEAATPPDAETKEFLPQKNSVVESFNVVKDDGRKRRQIPSASSALYFGGILGLVQ

ATLLISAAKPLLNFMGVTSDSPMLHPAMQYLKLRSLGAPAVLLSLAMQGVFRGFKDTKTPLYATVAGDLTNIALDPLFIF

VFRMGVNGAAIAHVISQYLLSAILLWSLNKQVDLIPPSIKHLQFDRFAKNGFLLFMRVIAVTFCVTLAASLAAHHGSTSM

SAFQVCLHSTNQYAYFDFYMTVLFVLMQPTICPNATCNNRAKSPSPREQIY

>MsMATE09

MAEKESLFSIGDWMRIPICTFFKDARLVFKLDDLGREILSIALPAAMALTADPIASLVDTAFIGQLGPVELAAVGVSIAL

FNQASRIFIFPLVSVTTSFVAEEDALSDASSQVEENGCLEAATPPDAETKEFLPQKNSVVESFNVVKDDGSKRRQIPSAS

SALYFGGILGLVQATLLISAAKPLLNFMGVTSDSPMLHPAMQYLKLRSLGAPAVLLSLAMQGVFRGFKDTKTPLYATVAG

DLTNIALDPLFIFVFRMGVNGAAIAHVISQYLLSAILLWSLNKQVDLIPPSIKHLQFDRFAKNGFLLFMRVIAVTFCVTL

AASLAAHHGSTSMSAFQVCLHSTNQYAYFDFYMTVLFVLMQPTICPNATCNNRAKSPSPREQIY

>MsMATE10

MNMNAETTPEQQLPSNQIQNEISDVKRELISLSLPALAGQAIDPIAQLMETAYIGRLGTLELASAGVSVVIFNIISKLFN

IPLLSVATSFVAEDMANISGNASIEISGNSNPFKAVYQRNQLSSVSTALLLALGIGIFEALALYFGSGIFLRLIGVSPGN

PTLVPAQKFLSLRAFGAPAVVLSLALQGIFRGFKDTKTPVICLGIGNLSAVFLFPLLMYYFRLGVAGAAISTVLSQYIGT

LLMIWCLNKRAVLLPPKMGNLQFGGYIKSGGFVLGRTLAVLTTMTLGTSMAARHGPVAMAAHQICMQVWLAVSLLTDALA

VSGQALIASSLSRHEYKAVKEITHFVLKIGLLTGICLTAILGASFGSLATLFTQDIEVLQVVRTGVLFVSASQPFNALAY

IFDGLHYGVSDFRYAAFSMMFVGAVSSAFLVFSPSHFGLRGVWLGLTLFMALRVVAGSVRLLSKNGPWWFLHKDFQIAEM

GS

>MsMATE11

MDMLSIALPAAVALAADPIASLIDTAFVGHIGAVELAAVGVSASVVNLVSKVFNVPLLNITTSFVAEEQALIGKEEDSGQ

IEENGKAQRKKLLSSVSTSLALAAGLGIAETVALSLGSGPLMTILGIAADSPIREPAEHFLTLRAFGALPIVIALAAQGT

FRGFKDTKTPLYAVGKYCSSFLPRSLHLLAHNLHTVDSSYNCL

>MsMATE12

MDMLSIALPAAVALAADPIASLIDTAFVGHIGAVELAAVGVSASVFNLVSKVFNVPLLNITTSFVAEEQALIGKEEDSGQ

IEENGKAQRKKLLSSVSTSLALAAALGIAETVALSLGSGPLMTILGIAADSPIREPAEHFLTLRAFGALPIVIALAAQGT

FRGFKDTKTPLYAVGAGNFLVVILDPILIFLCGLGISGAAIATVISEYLIAFILLWNLSGKVLLTPFDFDGPKFFSYLKS

GGLLIARTLAVFITMTLTTSLAANQGPIPMAGHQICMEVWLSISLLTDALALAGQSLLASSYSLGNYEHARLIIYRVIQI

GLGVGVTLSMILFFGFGPFSSLFSTDSEVLDVAQSGILFVAGSQPVNALAFVIDGLYYGVSDFEYAAYSMVLVGLISSVF

MLVAAPVVGLPGVWTGLFLFMALRVLAGVWRLSSKSGPWDMIWYENRAED

>MsMATE13

MDMLSIALPAAVALAADPIASLIDTAFVGHIGAVELAAVGVSASVFNLVSKVFNVPLLNITTSFVAEEQALIGKEEDSGQ

IEENGKAQRKKLLSSVSTSLALAAGLGIAETVALSLGSGPLMTILGIAADSPIREPAEHFLTLRAFGALPIVIALAAQGT

FRGFKDTKTPLYAVGAGNFLIVILDPILIFLCGLGISGAAIATVISEYLIAFILLWNLSGKVLLTPFDFDGAKFFSYLKS

GGLLIARTLAVFITMTLTTSLAANQGPIPMAGHQICMEVWLSISLLTDALALAGQVGSYYCIVL

>MsMATE14

MYTIQIYLRNVISDTPFSFKLVVPLIVLPVSLLSYRFSITLAASLAAHHGSTSMAAFQVCLQVWLAVSLLADGLAIARQA

ILAGAFANKDYEKVIVHYFKKIAGSF

>MsMATE15

MALKIPSISLLPRSLHNFPPRQNPNLKPLPLSPTISHSHLPHHFSSLSISALHRTQFVTARAIQPQQLTGDEGQITEASE

EAKIEDEEETTQGVEKELANQGIWIQLKEIVKFTAPATGLWICGPLMSLIDTAVIGQGSSIELAALGPATVVCDYMSYVF

MFLSVATSNMVATALAKQDREEVQHHISVLLFIGLACGSAMLFFTRLLGATTLAAFTGSKNVHLVPAANTYVQIRGLAWP

CLLIGSIAQSASLGMKDSWGPLKALAAASIINGIGDIVLCRYLNYGIAGAAWATLASQVVAAYMMSKALNDKGYNAFSFT

IPSGKEFLSIFSLAAPVFVTLMLKVAFYSLIIYFATSMGTNKIAAHQVMLQVYMLCAICGEPLSQTAQSFMPELMYGVNR

SLAKARSLLRSLLTIGAVFGLLLGIVVTSVTWLFPYIFTPDQMVIQEMHRILIPFFLALLVTPATVGLEGTLLAGRDLRF

ISLSMTGCFCLNGLVLLILSSRYGLQGCWFSLAGFQWVRFSSALLRLLSPNGILYSEDISQSELQKLKTA

>MsMATE16

MAHQFSLHFNHQTLHLVNRNLISHLNRHLPLHSLLNTTTTTVIHSTNQRIITSSSRNRRSGFLTPRVLQNQEVTNESEHQ

EQISEVSSKEQAQEEEMKEILVEQNIWIQMKEIVLFTGPAIGLWLCGPLMSLIDTAVVGQGSSIELAALGPATVVCDYMS

YAFMFLSIATSNMVATALAKQDREEVQHHISVLLFIGLACGSAMLFFTRLLGATTLAAFTGSKNVHLVPAANTYVQIRGL

AWPCLLIGSIAQSASLGMKDSWGPLKALAAASIINGIGDIVLCRYLNYGIAGAAWATLASQVVAAYMMSKALNDKGYNAF

SFTIPSGKEFLSIFSLAAPVFVTLMLKVAFYSLIIYFATSMGTNKIAAHQVMLQVYMLCAICGEPLSQTAQSFMPELMYG

VNRSLVKARSLLRSLLTIGAVFGLLLGIVVTSVTWLFPYIFTPDQMVIQEMHRILIPFFLALLVTPATVGLEGTLLAGRD

LRFISLSMTGCFCLNGLVLLILSSRYGLQGCWFSLAGFQWVRFSSALLRLLSPNGILYSEDISQSELQKLKTA

>MsMATE17

MAQKLSLHFNHTLHTSLHLNRHVPLRFLPPSLLRKNTTIHSPNQCIIISSSQNSRFEFLTACSVQNYDAIDEAEEKDQIS

EVSSKEEEEEVKELVEQSIWIQMKEIILFTGPAIGLWLCGPLMSLIDTAVVGQGSSIELAALGPATVFCDYLGYFFMFLS

VATSNMVATALAKQDREEVQHHISVLLFIGLACGSAMLFFTRLLGATTLAAFTGSKNVHLVPAANTYVQIRGLAWPCLLI

GSIAQSASLGMKDSWGPLKALAAASIINGIGDIVLCRYLNYGIAGAAWATLASQVVAAYMMSKALNEKGYNAFSFTIPSG

KEFLSIFSLAAPVFVTLMLKVAFYSLIIYFATSMGTNKIAAHQVMLQVYMLCAICGEPLSQTAQSFMPELMYGVNRSLAK

ARSLLRSLLTIGAVFGLLLGIVVTSVTWLFPYIFTPDQMVIQEMHRILIPFFLALLVTPATVGLEGTLLAGRDLRFISLS

TSGCFCSSALVLLILRSRYGLQGCWFSLVGFQWARFLMALLRLLSPSGILYSEDVSRYAEQKLKTV

>MsMATE18

MTLKLQLHIHHISSFKFLNLASPSQSHSPLRFHAPNDTFTISSKLFHVASKRRSIRTLNARVVGSNELTDESEEMGEKKE

LADQSVWNQMKEIVKFTGPAMGLWLCDPLMSLIDTAVIGQGSSTELAALGPATVVCDYMTLTFMFLSVVTSNIIATALAK

QDTEEVQHHISVLLFVGLACGFMMLLFTWLFGAATLTAFTGIKNAHVVPAANTYVQIRGLAWPALLVGWVAQSASLGMKD

SWGPLKALAAASVINGIGDILLCSCLGYGIAGAAWATMVSQVVTAYMMIQTLNKRGYNAFAFSIPSMKEFLTILSLAAPV

YLTSISKVAFFSLLIYVATSMGTQTMAAHQVMIQIYMACTVWGEPLCQTAQSFMPELTYGVNRSFPKAPIATKVSYNYWS

HTWIVIRDSWNISYLVIPIHIYI

>MsMATE19

MTLKLQLHIHHISSFKFLNLASPSQSHSPLRFHAPNDTFTISSKLFHVASKRRSIRTLNARVVGSNELTDESEEMGEKKE

LADQSVWNQMKEIVKFTGPAMGLWLCDPLMSLIDTAVIGQGSSTELAALGPATVVCDYMTLTFMFLSVVTSNIIATALAK

QDTEEVQHHISVLLFVGLACGFMMLLFTWLFGAATLTAFTGIKNAHVVPAANTYVQIRGLAWPALLVGWVAQSASLGMKD

SWGPLKALAAASVINGVGDIVLCTYLGYGIAGAAWATMASQVVAAYMMMRTLNMKGYNAFALSIPSGREFLTILGLAAPV

FMTMMSKVAFYSLLIYFATSMGTHTMAAHQVMVQTFCMCTVWGEPLSQTAQSFMPELLYGVNRNLSKARMLLRSLAVIGA

TLGLLLGIVGTSVPFLFPYIFTSDQMVIREMHKVLVPYFVALAVTPPTHSLEGTLMAGRDLRFISLSMIGCLCGGALVLS

ILCSRYGLQGCWFSLALFQWARFSVALLRLLSPKGILYSEDIDHNRLQKLKTA

>MsMATE20

MGNANLGVLRVRGGDENNSPVAIETAIFFLSFFLSFFSFTSKMAHQFSLHFNHQTLHLVNRNLISHLNRHLPLHSLLNTT

TTTVIHSTNQRIITSSSRNRRSGFLTPRVLQNQEVTNESEHQEQISEVSSKEQAQEEEMKEILVEQNIWIQMKEIVLFTG

PAIGLWLCGPLMSLIDTAVVGQGSSIELAALGPATVVCDYMSYAFMFLSIATSNMVATALAKQDREEVQHHISVLLFVGL

ACGFMMLLFTWLFGAATLTAFTGIKNAHVVPAANTYVQIRGLAWPALLVGWVAQSASLGMKDSWGPLKALAAASVINGVG

DIVLCTYLGYGIAGAAWATMASQVVAAYMMMRTLNMKGYNAFALSIPSGREFLTILGLAAPVFMTMMSKVAFYSLLIYFA

TSMGTHTMAAHQVMVQTFCMCTVWGEPLSQTAQSFMPELLYGVNRNLSKARMLLRSLAVIGATLGLLLGIVGTSVPFLFP

YIFTSDQMVIREMHKVLVPYFVALAVTPPTHSLEGTLMAGRDLRFISLSMIGCLCGGALVLSILCSRYGLQGCWFSLALF

QWARFSVALLRLLSPKGILYSEDIDHNRLQKLKTA

>MsMATE21

MALKIPSISLLPRSLHNFPPRQNPNLKPLPLSPTISHSHLPHHFSSLSISALHRTQFVTARAIQPQQLTGDEGRISEASE

EAKIEDEAATQGVEKELANQGIWIQLKEIVKFTAPATGLWICGPLMSLIDTAVIGQGSSIELAALGPATVVCDYMSYVFM

FLSVATSNMVATALAKQDTEEVQHHISVLLFVGLACGFMMLLFTWLFGAATLTAFTGIKNAHVVPAANTYVQIRGLAWPA

LLVGWVAQSASLGMKDSWGPLKALAAASVINGVGDIVLCTYLGYGIAGAAWATMASQVVAAYMMMRTLNMKGYNAFALSI

PSGREFLTILGLAAPVFMTMMSKVAFYSLLIYFATSMGTHTMAAHQVMVQTFCMCTVWGEPLSQTAQSFMPELLYGVNRN

LSKARMLLRSLAVIGATLGLLLGIVGTSVPFLFPYIFTSDQMVIREMHKVLVPYFVALAVTPPTHSLEGTLMAGRDLRFI

SLSMIGCLCGGALVLSILCSRYGLQGCWFSLALFQWARFSMALLRLLSPKGILYSEDIDHNRLQKLKTA

>MsMATE22

MVIKQMGMVLGLALAFILGTGLHFGAKLFTKDDDVLHLIRVGIPFVALTQPLNCLAFVFDGVNFGASDFAYSAFSMVIVA

IISIICLLILSSAGGFIGIWVALTIYMSLRAFAGFLRIGTGSGPWEFLRS

>MsMATE23

MLFLCLCLVFLFVVLFGARVSNALGAGCPHAARLSVYAAMAIAVSEAILVSSIIFASRRVLGYIFSNEQDVVDYVTDMAP

LISLSVIADSLHGTLSGIARGCGWQKSGAYVNLGSYYVFGIPIAVILGFWFELRGKGLWIGIIVGASCQAVLLSLITSFT

NWEKQAIKARERIFRERFVIEDRLV

>MsMATE24

MSSTCKLGENLSSNHKTNQPTSPPPPLQTRKCDTNPAKTLLSEELRVQGRLAFPMVLMNLAWFAKTAITTAFLGRLGELS

LAGGALGFTFANVTGFSVLNGLCGAMEPICGQAHGAKNVRLLHKTLLMTIVLLLLVTIPITFMWLHIDKILIHFGQQQEI

STVAGTYVYYLIPDLFVMSLLCPLKAYLSSQSITLPTMFSSGVALAFHVPVNILLSKTMGLRGVSMAVWITDLIVVVLLA

IYVLILENRKVLAWKEGGWWDQSIMDWIRLIKLSGSCCLNTCMEWSCYEILVLLTGHLANAKQALGVLAIVLNFDYLLFS

VMLSLATCVSTRVSNELGANQADRAYRSARVSLGIGFIAGCTGSLVMVAARGIWGQLFSHDRGTINGVKKTMLLMALVEL

FNFPLAVCGGIVRGTARPWLGMYANLGGFYFLALPLGVVFAFKLRLGLVGLFFGLLTGIVVCLSLLLVFIARIKWVEEAA

KAQILASNDQVKEVPCDDAEVPTEARENDKV

>MsMATE25

MREDDKDHDFFSHKFPTTSQVVEELKELWSMALPITAMNMLVFVRAVVSVLFLGRLGSLELAGGALSIGFTNITGYSVLV

GLASGLEPVCSQAFGSKNWELLSLSLQRMVLILLMAIVPISLLWLNLEKIMLFMGQDGKITEMAAIYCFYSLPDLLTNTL

LQPLRVFLRSQKVTKPMMYCSLIAVVFHVPLNYFLVMVMQFGVPGVAMASVLTNMNMVVLMAGYVGLFRKKEMMLRWPGC

GEGGMMVVSEGLGELMKLAVPSCLMICLEWWWYEIVTVLAGYLENPTLAVAATGILIQTTSMMYTVPMALAGCVSARVGN

ELGAGKPYKAKLAAMVALGCAFVMGFINVTWTVILRYGWAGLFTNDEPVKALVASVMPIMGLCELGNCPQTTGCGILRGT

ARPVIGANINLGSFYFVGTPVAVGLAFWFKIGFSGLWFGLLSAQVACALSILYVVIIKTDWEAEALKAEKLTKVEMVICN

ESKKNKDKKKNEECKGLLENENGNKIDMC

>MsMATE26

MVAQEKSQKTYPTTAEVVDELKKMMDIGFPIAAMSIVGYLKNMILVVCMGKLGSLELASGALAIGFTNVTGYSVLSGLAM

GMEPLCTQAIGSQNFSLVSLILRRTILMLLVASLPISLLWLNLEPFMLSLHQNQDITRIASLYCRLSIPDLIANSLLHPI

RIYLRSKGTTWPLLWCTSLSVIIHIPIIIFLTFKLHLGVQGIAISAFVANFNTLFFLLSYMFYMHVSHVSISIPIPSPPL

LSSQQEKPASVKTLGKEWGMLIRFSIQSCLGVCLEWWWYEFMTILAGYLYNPRVALATAGIVIQTTSLMYTLPTALSASV

STRVGNALGAGQPSRANLSTMVAIGMSLASSTLGLLWTTLGREKWGKVFTNDKEVLELTMAVLPIIGVCELANCPQTTSC

GMLRGSARPGIGAGINFYSFYLVGAPIGIVLGFVLKLGLVGFCYGLLAAQIACVVSILVVVYNTDWERESLKAKSLVGND

TCDTLFAHVEDQTIKCEQGIVFLNENK

>MsMATE27

MSETKINTLNEPMISKDTLNQRDIIVTETKSLLSLALPTALTALIFYARSMISMMFLGKLGDVELASGSLAIAFANITGY

SVLSGLSLGMEPLCSQAFGANRPKLLSLTLQRCIIFLLSCSLPISFLWFNMSRIFHFLHQDDKITQMSQTYLVFLLPDLV

TNSFLQPIRIYLRAQSVTYPVTLASLVGTFLHLPFNFLLFKKGISGIAIASAASNFSVLVVLVVYVWISGVHIATWNAPS

RECFFGWGPLIKLAAPSCVSVCLEWWWYEIVIVLCGFLVDPTATVASMGILIQTTSLIYVFPSSMGLAVSTRIGNALGAN

RPRNARFSAVIAVFFAAVMGFTAVIFTMMMRWQWGKMFTADEDIIRLTAAALPILGLCELGNCPQTVGCGVVRGTARPKV

AANVNLSAFYMVGMPVAVGLAFWFDFGFCGLWLGLLSAQVCCAGLMLYIVGTTDWEQQARRAQLLTTFDEVDNGLEGQKE

SLISGLESA

>MsMATE28

MCNPKPSSPTSPFLSPTKTHLINPHTKASYSNPPTLDDDHVQDEIHRWPTLKEAITEIKEIGKISGPTTITGLLLYSRAM

ISMIFLGYLGEMELAGGSLSIGFANITGYSVISGLAMGMEPICGQAYGAKQWKILGLTLQRTVLLLLSTSIPISFIWINM

KRILLFSGQDEEISSMAQSFILFLVPDLFLLSILHPLRIYLRTQGITLPLTYCSAVSVLLHIPLNFLLVVHFQMGIAGVS

IAMVLTNLNLVILLSSFLYFSSVYKKSWISPSLDCIKGWSSLLSLAIPTCVSVCLEWWWYEFMIMMCGLLVNPKATIASM

GILIQTTSLVYVFPSSLSLGVSTRIGNELGANRPQKARISMIVSLFVAMVLGLGAMLFTTLMRNQWGKFFTNDKEILELT

SIVLPIVGLCELGNCPQTTGCGVLRGSARPTIGANINLGSFYLVGMPVAILLGFVAKLGFPGLWIGLLAAQGSCAMLMLV

VLCRTDWNLQVQRAKELTKSSTISDDVDAKLPTFMEGNVNKNNVHGCLEEIVITHDVFTKKSSLETDPLIITSTTTNCIE

D

>MsMATE29

MCKPKPSSTSPFLCPTQTNLITSDPKLLINDPPHDEVQDQNELQRWPTPNEVIEELKAIGKISGPTAITGLLLYSRAMIS

MLFLGYLGETELAGGSLSIGFANITGYSVLSGLAMGMEPICGQAYGAKQWKILGLTLQRTVLLLLSTSIPIAYLWLNMKK

ILMSCGQDEEISSTAQTFILFSLPDLFFLSFLHPLRIYLRTQNITLPLTYCSAISVTLHVPLNFLLVIHFKMGVVGVAIA

MIWFNLNLLIFLSSFVFFSRVYKDSWVFPSMDCLKGWSSLLALSIPSCVSVCLEWWWYELMIILCGLLVNPKSTISSMGI

LIQTTSLVYVFPSSLSFGVSTRVGNLLGANSPSKARFSMIVSMFCGFGLGILAMVFTTLMRNQWGRLFTSDDEILNLTAM

VLPIVGLCEIGNCPQTTGCGVLRGSARPTVGANINLGSFYLVGMPVAIVLGFVVKMGFVGLWFGLLAAQGSCAVLMLYVL

CTTDWNDQIERSKNLTKATTTTIGFSDSTFITKTVLRHDNNNNNHCGCLEEIIVITHDDATKTCTHSLESDPLLPNM

>MsMATE30

MCHLTSQPPSKCNSKSEYLIVSIKDTKESNNNMMTNPLIQKDTNIENPTTQFQKTHLRATFKEVISISKIAFPMIFTGLL

LYCRSMISMLFLGHLGELALAGGSLAVGFANITGYSILSGLAVGMEPICGQAFGAKRFTLLGLCLQKTILLLLLTSIPIS

LLWLYTKHILLLCGQEEDIATQAQIYLLYSIPDLLAQSFLPPLRIYLRSQSITLPLTLCATLAIFLHIPINYFLVSHLNM

GIKGVALSGVWTNFNLVASLILYIIFSGTHKKTWGGFSSECFKQWKSLLNLAVPSCLSVCLEWWWYEIMILLCGLLINPR

ATVASMGILIQTTSLLYIFPSSISFSVSTRVGNKLGAQKPSKAKLSAIVGLSCSFILGVFALFFAIMVRNIWASMFTEDK

EIIKITSLVLPLIGLCELGNCPQTTGCGVLRGTARPKVGANINFGCFYIVGMPVAIWLAFYVGFDFQGLWLGLLVAQGTC

AVTMLVVLSQTDWDCEALRAKKLTGIGEATTKNDVFVDDSKEVDEEKLLKAEIKEYSS

>MsMATE31

MCKLSSSSTSVSTLYESNNNQTNNISTTTIKTQNNIKPDMLTPLIPKSPTFKQQKKTHFSLALNEAKHISNIALPMVLTG

LLLYSRSIISMLFLGRVGELALAGGSLAIGFANITGYSILSGLAMGMEPICGQAFGAKRFKLLGLTMQRTVILLLVTSIF

ISFLWLNMKRLLLLCGQQEDIANVAQSYILYSLPDLVAQSLLHPLRIYLRSQSITLPLTYSATLSILLHIPINYFLVNVL

QLGIRGIALGSVWTNFNLVVSLIIYIWVSGTHKKTWSGISSACFKGWKSLLNLAIPSCISVCLEWWWYEIMILLCGLLLN

PHATVASMGVLIQTTALIYIFPSSLSFGVSTRVGNELGAENPQKAKLAAIVGLCFSFVLGFSALFFAFSVRNIWATMFTS

DPQIIALTSMVLPIIGLCELGNCPQTTVCGVLRGTARPKLGANINLGCFYLVGMPVAVWLSFFAGFDFKGLWFGLMAAQG

SCMVTMLFVLVRTNWENQAERAKELTSSDSSEEEQEEEKVIINSSSCGTKECSDSLV

>MsMATE32

MAASIISDGTEAPLLADDHGKQNTRPQIEKWWNKILDIEEAKIQLMFSLPMILTNLFYYLITLVSVMLVGHLGELQLAGA

TLANSWFSVTGVAVMVGLSGALETLCGQGFGAKEYHMLGIYLQGSCIISFIFSIIISIVWFYTEQILVLLHQSQDIARTA

ALYMKFLIPGLFAYSILQNLLRFLQTQSVVMPLVILSAIPALVHVGIAYGFVQWTGLNFIGGPTATSISLWISMIMLGLY

VMYAKKFKNTWRGFSMQSFDYLLANIRLALPSAAMVCLEYWAFEVLVFLAGLMPDSQITTSLIAICANTEFIAYMITYGL

SAAASTRVSNELGAGQPERAKHAMGVTLKLSLLLGLCFVLILVFGHDIWIQLFSDSPIIKKEFASVTPLLAISILLDSVQ

GVLSGVVRACGWQYVAVYVNLATFYLIGLPISCLLGFKTDLQYKGLWIGLICGLVCQTGALLLLTRHVKWTKLNLSGDKD

KDQPIVV

>MsMATE33

MATSGISDGTATQNTEPQMEKKWWNKILDIKEAKHQLMFSLPIILTTILYYSINLVSVMLVGHLGELQLAGATLANSWFG

VTAVGVMVGLSGALDTLCGQGFGAKEYHMLGIYLQSSCIISFIFSIIISIIWFYTEQILVLLHQSQDIARTAALYMKFLI

PGLFAFGTLRNMLRFLLTQSVVMPLVILSAIPAIVHVGIAYGFVHWSGLNFKGGPVATSISQWLSMILVGFYILYAKKFK

NTWRGFSMRSFQYLFTNLKLALPSAAMLWYEFLKAFHFVCPF

>MsMATE34

MAASGISDGTATQNTEPQMEKKWWNKILDIKEAKHQLMFSLPIILTTILYYSINLVSVMLVGHLGELQLAGATLANSWFG

VTAVGVMVGLSGALDTLCGQGFGAKEYHMLGIYLQSSCIISFIFSIIISIIWFYTEQILVLLHQSQDIARTAALYMKFLI

PGLFAFGTLRNMLRFLLTQSVVMPLVILSAIPAIVHVGIAYGFVHWSGLNFKGGPVATSISQWLSMILVGFYILYAKKFK

NTWRGFSMRSFQYLFTNLKLALPSAAMLCLESLAFEVLVFLAGLMSDSQITTSLIAICENTEFIAYLITYGLSAAASTRV

SNELGAGQPERAKHAMRVSLKLSLLLGLCFALMIVFGHDIWIRLFSSSPTIKHKFASISPFLAISILLDSVQGVLSGVVR

ACGWQHVAVYVNLATFYLIGLPISCILGFKTNLQYKGLWIGLICGLACQTVTLLLLTRYAKWTKLNLSGDKDKDQPVVVL

TTECMPIRTE

>MsMATE35

MKFLIPGLFAYSILQNLLRFLQTQSVVMPLVILSAIPALVHVGIAYGFVQWTGLNFIGGPTATSISLWISMIMLGLYVMY

AKKFKNTWRGFSMQSFDYLLANIRLALPSAAMVCLEYWAFEVLVFLAGLMPDSQITTSLIAICANTEFIAYMITYGLSAA

ASTRVSNELGAGQPERAKHAMGVTLKLSLLLGLCFVLILVFGHDIWIQLFSDSPIIKKEFASVTPLLAISILLDSVQGVL

SGVVRACGWQYVAVYVNLATFYLIGLPISCLLGFKTDLQYKGLWIGLICGLVCQTGALLLLTRHVKWTKLNLSGDRDKDQ

PVVVLTTECMPIRTE

>MsMATE36

MEKGLLEKDREGGSRSITWGVFVQEVKDVCFLALPMIAVTLSQYFLQIISMMMVGRLGKLALSSTAIAISLCAVSGFSLL

FGMSCALETQCGQAYGAKQYKKFGVQIYTAVFSLIIACLPLSLLWIFLGRLLILLGQDPLISQEAGKFAMCMIPALFAYA

TLQALVRYFLMQSLILPLVISSSVTLGFHVAFCWLLVFKSGLGSLGAAFSIGTSYWLNVIILGLYMKFSADCEKTRVTIS

MESFTGIGEFFRYAIPSAGMICFEWWSFELLVFLSGLLPNPQLETSVLSICLSIISTLYTIPEATGSAASARVSNALGAG

CPHAARLSVYAAMAIAVSEAILVSSIIFASRRVLGYIFSNEQDVVEYVTDMAPLISLSVIVDSLHGTLSGIARGCGWQKS

GAYVNLGSYYIFGIPIAAILGFWFELRGKGLWIGIIVGASCQAVLLSLITSFTNWEKQAIKARERIFREGFVIEDRLV

>MsMATE37

MEKGLLEKDREGGSRSITWGVFVQEVKDVCFLALPMIAVTLSQYFLQIISMMMVGRLGKLALSSTAIAISLCAVSGFSLL

FGMSCALETQCGQAYGAKQYRKFGVQVYTAIISLIIACVPLSLLWLNLGKLLSLLGQDPLISQEAGKFAMCMIPALFAYA

TLQALVRYFLMQSLILPLVISSSVTLGFHVAFCWLLVFKSGLGSLGAAFSIGTSYWLNVIILGLYMKFSADCEKTRVTIS

MESFAGIGEFFRYAIPSAGMICFEWWSFELLVFLSGLLPNPQLETSVLSICLSIISTLYTIPEATGSAASARVSNALGAG

CPHAARLSVYAAMAIAVSEAILVSSIIFASRRVLGYIFSNEQDVVDYVTYMVPLISLNVIVDSLHGTLSGIARGSGWQKL

GAYVNLGAYYVFGIPIAVILGFWFELRGKGLWIGILVGAFCQALLLALITGFTNWEKQAIKARERIFQGRQEVLQ

>MsMATE38

MEKGLLEKDREGGSRSITWGVFVQEVKDVCFLALPMIAVTLSQYFLQIISMMMVGRLGKLALSSTAIAISLCGVSGFSLL

FGMSCALETQCGQAYGAKQYKKFGVQIYTAVFSLIIACLPLSLLWIFLGRLLILLGQDPLISQEAGKFAMCMIPALFAYA

TLQALVRYFLMQSLILPLVISSSVTLCFHVAFCWLLVFKSGLGCLGAALSIGTSYWLNVIILGLYMKFSTDCEETRVPIS

MEPFLGIGEFFRYAIPSAGMIW

>MsMATE39

MDLLGKAIDFTRSRPSDFTRSWKICHVHDSCSVCLCNASGTGSLGAAFSIGTSYWLNVIILGLYMKFSADCEKTRVTISM

ESFAGIGEFFRYAIPSAGMICFEWWSFELLVFLSGLLPNPQLETSVLSICLSIISTLYTIPEATGSAASARVSNALGAGC

PHAARLSVYAAMAIAVSEAILVSSIIFASRRVLGYIFSNEQDVVDYVTYMVPLISLNVIVDSLHGTLSGIARGSGWQKLG

AYVNLGAYYVFGIPIAVILGFWFELRGKGLWIGILVGAFCQALLLALITGFTNWEKQAIKARERIFQGRQEVLQ

>MsMATE40

MGEERRVMEESLLSKQIDSKAENDNEEERKNREISWDIYTKELKRICYLSGPMVAVTSSQYLLQVVSIMIVGHLGELYLS

SAALAISFTGVTGFSFLMGMASGLETTCGQAYGAKQYQRIGVQTYTSIFSLILVCLPLSFIWINIENILVFTGQDPLIAH

EAGRFTIWLLPALFAYAILQPLVRYFQIQSLLIPMLLSSCVTLCIHIPLCWALVFKTGLSNIGGAIAMSISIWLNVIFLG

LYMRYSSSCAKTRAPISMELFQGIWEFFRFAIPSAVMVCLEWWSYELIVLLSGLLPNPQLETSVLSVCLNTIATLYTIPF

GIGAAASTRVSNELGAGNPFEARVAVLAAMSLALTEASIVSATLFACRHVYGYIFSSDTEVVKYVTVLAPLVSISVILDS

IQGVLAGVARGCGWQHIGVYVNLGAFYLCGIPVAAALAFWVQVGGKGLWIGIQVGAFVQCILLSIITCCINWEQLAIKAR

QRLFDVQFSGENRLV

>MsMATE41

MIMKETMEEGSNNNKCEWIKTRTTLMEELKKMGTIAVPMVATSVLQYLLQVVSVMMVGHLNQLSLSSVAIATSLTNVSGF

SILSGMAGGLETLCGQAYGAGHFEKHGIYTYTAVISLTMVCAPITIIWTFMDKILILIGQDPTISLQARTFALWLIPALF

ASAILKPLTRFFQTQSLIFPMIISSFIVLCFHGVMCWTLVFKLGLGHVGAAISFSLGTWLNVLILLSFVKYSSSCEKTRV

PFSMKAFLGIREFFGLAVPSAAMVCLKWWACELLVLLAGLFPDPKLETSVLSICLTISTLHFTISYGLGAAASTRISNEL

GAGNPKAVRFSICTAMFLATTEALIITAILLGCRCVLGYAYTNDTMVVHYVAGMTPLLCVSIFTDSLQAVLSGVARGSGW

QYVGAYVNLGAFYLVGIPIGVVLGFIVHFKAKGLWIGIVAGSIVQTIFLSIITSLTNWKKQAIMARERIFDATSSDESVT

DHMTRA

>MsMATE42

MGKEEATPLLTKNEENDELAPLEGAFWAEFKRVGSMAAPMVTVTVSQYLLQVVSLMMVGHIGILTSFSGVALAISFAECT

GFCVLIGMAGALETLCGQTYGAEEFSKIGNYICSAMITLILVCFPISLMWIFIDKLLLLFGQDTEIAQAAREYCICLIPA

LFGHAVLQSLIRYFQIQSMIFPMVFSSIVILCLHVPICWCLVFKLGLGHVGAAFAIGIAYWLNVIWLGIYMKYSPACEKT

KIVFSYNSLLYIAEFCQFAIPSGLMFCLEWWSFEILTIVAGLLPNSQLETSVLSVCLSTTTLHYFIPHAIGASASTRVSN

ELGAGNPRAAKGAVRVAVIIGIAEAVIVSTLFLCFRNFIGNAYSNDKEVVDYVTDMVPLLCVSVSADSIICALSGIARGG

GFQTIGAYVNLGAYYLVGAPIAYFLGFGLKLNAKGLWMGTLTGSILNVIILAVVTMLTDWQKEATKARERIAEKPIEAHD

GSI

>MsMATE43

MTMEGVEIENTFIQELKKVSFMAAPMVAVTVSQYLLQVVSLMMVGHLGILVSFSGVSIAMSFAEVTGFSVLLGMAGALET

LCGQTFGAEEYGKLGNYTCCAILTLTVVCFPISLVWIFTDKILLFFSQDPGMSHVAREYCIYLIPALFGYALLQALIRYF

QTQGMIFPMVFSSVSALFLHIPICWILVFKLGLGHIGAALAIGISYWLNVIWLWVYIKYSPSCQKTKIVFSTHALHNLPE

FCKYAIPSGLMFCFEWWSFEILILIAGLLPNPQLETSVLSVCLNTTSLHFFIPYAIGASASTRVSNELGAGNPKTAKGAV

RVVVIIGIAEAIIVSTFFLCFRNILGYAYSNDEQVVNYIADMVPLLCVSVSADSLIGALSGVARGGGFQEMGAYVNLGAY

YIVGIPIGLLLGFHLKLNAKGLWMGTLSGSVLNVIILSIVTALTDWQKEATKARERIIDQSIKTNNTLVVA

>MsMATE44

MKNSREMSKEVTTPLLRKSDDHGSDGRVEIENTFIQELKKVSFMAAPMVAVTVSQYLLQVVSLMMVGHLGILVSFSGVSI

AMSFAEVTGFSVLLGMAGALETLCGQTFGAEEYGKLGNYTCCAILTLTVVCFPISLVWIFTDKILLFFSQDPGMSHVARE

YCIYLIPALFGYALLQALIRYFQTQGMIFPMVFSSVSALFLHIPICWILVFKLGLGHIGAALAIGISYWLNVIWLWVYIK

YSPSCQKTKIVFSTHALHNLPEFCKYAIPSGLMFCFEWWSFEILILIAGLLPNPQLETSVLSVCLNTTSLHFFIPYAIGA

SASTRVSNELGAGNPKTAKGAVRVVVIIGIAEAIIVSTFFLCFRNILGYAYSNDEQVVNYIADMVPLLCVSVSADSLIGA

LSGVARGGGFQEMGAYVNLGAYYIVGIPIGLLLGFHLKLNAKGLWMGTLSGSVLNVIILSIVTALTDWQKEATKARERII

DQSIKTNNTLVVA

>MsMATE45

MLIVYICGFSKHSNWKRKVARAPRQQMREMMIGICRKDITEEVKKQLWLAVPMIFASVFQYSLQMISLMFIGHMNDEVLL

AGAALANSVTSVFGYSVLVGFSCALETFCGQSYGAQKYHMVGIHLQRAILINMLLTIPQSIILANLRPILIFLYQDPNIA

AEAGFYGRYLIPNVFANAILSCIVKFLQTQNIVVPMLLASGITSLVHFLNCWIWIIKLRHGIKGAAIATCISNWLYTVLL

VLYIKFSSSCRSTWTGFSRESLNNIPQFLRIAFPSAIMVCLESWMYEIMVLLSGTLPNPKLQTSVLSLCMNIGAVVWMIS

LGLSGAASVRVSNELGAGNARAARLAVCVVVVIVVTEGILVGTVMILLRNIWGYAYTSKVEVVKQIEIMFPILAAGNLID

ALQSVLAGIARGSGWQKAGAVVYLGSCYLVGIPAAIIFAFVLHTGVKGLLFGIICALIAQAFSLMIITLRTDWEKEANKA

KDRVYKSITPESFRNRHS

>MsMATE46

MIIGLCRKDITEEVKKQLWLAVPMIFASVFQYSLQMISLMFIGHMNDEVLLAGAALANSVTSVFGYSVLVGFSCALETFC

GQSYGAQKYHMVGIHLQRAILINMLLTIPQSIILANLRPILIFLYQDPNIAAEAGFYGRYLIPNVFANAILSCIVKFLQT

QNIVVPMLLASGITSLVHFLNCWIWIIKLRHGIKGAAIATCISNWLYTVLLVLYIKFSSSCRSTWTGFSRESLNNIPQFL

RIAFPSAIMVCLESWMYEIMVLLSGTLPNPKLQTSVLSLCMNIGAVVWMISLGLSGAASVRVSNELGAGNARAARLAVCV

VVVIVVTEGILVGTVMILLRNIWGYAYTSKVEVVKQIEIMFPILAAGNLIDALQSVLAGIARGSGWQKAGAVVYLGSCYL

VGIPAAIIFAFVLHTGVKGLLFGIICALIAQAFSLMIITLRTDWEKEANKAKDRVYKSITPESIVI

>MsMATE47

MIIGLCRKDITEEVKKQLWLAVPMIFASVFQYSLQMISLMFIGHMNDEVLLAGAALANSVTSVFGYSVLVGFSCALETFC

GQSYGAQKYHMVGIHLQRAILINMLLTIPQSIILANLRPILIFLYQDPNIAAEAGFYGRYLIPNVFANAILSCIVKFLQT

QNIVVPMLLASGITSLVHFLNCWIWIIKLRHGIKGAAIATCISNWLYTVLLVLYIKFSSSCRSTWTGFSRESLNNIPQFL

RIAFPSAIMVCLESWMYEIMVLLSGTLPNPKLQTSVLAICMNIASVVWMLSSGFTGAASIRVSNELGAGNPRAARLAVCV

VVVLNITEAILVGTVMILLRNIWGYAYTKDIEVVKQIAIMLPILAVSYFLDSLQSVLAGIARGSGWQKAGAVVYLGSCYL

VGIPAAIIFAFVLHTGVKGLLFGIICALIAQAFSLMIITLRTDWEKEANKAKDRVYKSITPESFRNRHS

>MsMATE48

MIIGLCRKDITEEVKKQLWLAVPMIFASVFQYSLQMISLMFIGHLDDEVLLAGASLANSFMNVIGISILFFMEATYSERG

KRKFSRILGANLDKMVFFLSSKCVLVDVIGFEEVVWG

>MsMATE49

MNILSSTLTMETSNLETPLCSDQHELDAQTFDQGCCIREDVVEEAKKQLWLAGPLIAVSLLQYSLQMISIMFVGHLGKLP

LSGASLGNSFASVTGYSVLLGMGSALETLCGQAYGAEQYHMLGVHTQRAMLVLIALSIPLSLIWFNTCNLLIALGQNHEI

STEAGTFNRWMIPGLFAYALVQCLNRFLQTQNNVFPMLISSGITTLVHLVFCWVFVFEYELGIKGAALAISLSYWVNVFM

LVIYINSATACASTWTGVSKEALNDILSFVRLAMASTLMICLEYWSFEMVVLLSGLLPNPQLETSVLSISLNTCWMVYMI

SVGLGGAISTRVSNELGCGNAKGALLALRVMIVIAIVEGTVVVLVTILVRNVWGKLYSNEDEVIKYVAKMMPLLALSDFL

DGFQCVLSGAARGCGWQNLCASINLGAYYVVGIPSAILFAFTFHIGGMGLWMGIICGLCVQGIALVTVNARTNWDRDVTN

IFYASKFNHFNRLRL

>MsMATE50

MRSERQELVIAELKRQLWLAVPLSSVGILQYILQTISIVFVGHLGTLPLSGASMATSFASVTGFTLLMGITSALDTFCGQ

SNGAEQYHMLGIHMQRAMIVVSIVSVFLAIIWANTKQILVVMHQDKAISKEAGSYAFFLIPSLFAYGPLQCILKFLQTQN

IVLPMVVTSGIAALLHTLLCWLLVFEFKLGSKGAAISNSICYWVNVLLISLYVKFSSTCKQTWTGFSKRALQDLFVFLRL

AIPSALMVCLKVWTFELMVLMSGLLPNPVIETSVLSICLNTFGLAWMIPFGCSCAVSIRVSNELGGGNPNGASLAVRVAL

SISFIAALFMVLSMILARKVWGHLYSDDKQVIRYVSAMMPILAISSFLDAIQSTLSGVLAGCGWQKIGAYVNLGSFYVVG

VPCAVVLAFFVHMHVMGLWLGIISAFIVQTLLYSIFTIRSNWEEEATKAQRRVERSTTTPNTTLRDSISPSQKLEQIP

>MsMATE51

MSLDSPLIVEETKQTNKKEEDRKDLVEEVKKQLWLSGPLISVTLLNFGINLISVMFVGHLGELPLSGASMATSFASVTGF

SLLQGMASALDTLCGQSYGAKQYRMLGVHMQRAMFILMIVAVPLAIIWANTRSILIFLGQDPEISTEAGNYAKLMVPCLF

AYGLLQCLNRFLQTQNIVFPMMFCSAMTTLLHLPICWFMVYKSGLGSRGAAIANSISYWINVTILALYVKFSPSCKKTWT

GFSKEAFALNNIPIFLKLAIPSAVMVCLEMWSFELMVLLSGLLPNPKLETSVLSISLNTSALVWMIPFGLSGAISIRVSN

ELGAGNPRAARLAVYVVVVIAVIESIVVGAVIILIRNIWGYAYSNEEEVVKYVAIMLPIIAVSNFLDGLQCVLSGTARGV

GWQKIGAYVNLGSYYLVGIPAAVVLAFVLHVGGKGLWLGIICALVVQVLSLTIITIRTDWEKEAKKANDRVYDSITTESL

VS

>MsMATE52

MSLDSPLIVEETKQNNKKEEDRRELVEEVKKQLWLSGPLISVTLLNFGINLISVMFVGHLGELPLSGASMATSFASVTGF

SLLQGMASALDTLCGQSYGAKQYRMLGVHMQRAMFILMVVAIPLAVIWANTRSILIFLGQDPEISIEAGNYAKLMVPCLF

AYGLLQCLNRFLQTQNIVFPMMFCSAMTTLLHLPICWFMVYKSGLGSRGAAIANSISYWINVTILALYVKLSPSCKKTWN

GFSKEALALKNIPIFLKLAIPSAIMVCLEMWSFELMVLLSGLLPNPKLETSVLSISLNTSALVWMIPFGLSGAISIRVSN

ELGAGNPEAARLAVYVVVVIAIIESIVVGAVIILIRNIWGYAYSNEEEVVKYVAIMLPIIAVSNFLDGLQCVLSGTARGV

GWQKIGA

>MsMATE53

MEAHEGSILQLTYYYFDVMFVGHLGELPLSGASMATSFASVTGFSLLQGMASALDTLCGQSYGAKQYRMLGVHMQRAMFI

LMIVAAPLAIIWANTRSILIFLGQDSEISTEAGSFAKLMVPCLFAYGLLQCLNRFLQTQNIVFPMMFSSAITTLLHIPLC

WIMVYKSGLGSGGAAIASSISYWINVTILALYVKFSPSCKKTWTGFSKEAFALNNIPIFLKLAIPSAVMVCLEMWSFELM

VLLSGLLPNPKLETSVLSISLNTSALVWMIPFGLSGAISIRVSNELGAGNPRAARLAVYVVVVIAVIESIVVGAVIILIR

NIWGYAYSNEEEVVKYVAIMLPIIAVSNFLDGLQCVLSGTARGVGWQKIGAYVNLGSYFLVGIPAAVVLAFVLHVGGKGL

WLGIICALVVQVLSLTIITIRTDWEKEAKKANDRVYDSITTESLVS

>MsMATE54

MSLDSPLIVEETKQNNKKEEDRRDLVEEVKKQLWLSGPLITVTLLNFGINLISVMFVGHLGELPLSGASMATSFASVTGF

SLLQGMASALDTLCGQSYGAKQYRMLGVHMQRAMFILMIVAAPLAIIWANTRSILIFLGQDSEISTEAGSFAKLMVPCLF

AYGLLQCLNRFLQTQNIVFPMMFSSAITTLLHIPLCWIMVYKSGLGSGGAAIASSISYWINVTILALYVKFSPSCKKTWT

GFSKEAFALNNIPIFLKLAIPSAVMVCLEMWSFELMVLLSGLLPNPKLETSVLSISLNTSALVWMIPFGLSGAISIRVSN

ELGAGNPRAARLAVYVVVVIAVIESIVVGAVIILIRNIWGYAYSNEEEVVKYVAIMLPIIAVSNFLDGLQCVLSGTARGV

GWQKIGAYVNLGSYFLVGIPAAVVLAFVLHVGGKGLWLGIICALVVQVLSLTIITIRTDWEKEAKKANDRVYDSITTESL

VS

>MsMATE55

METPLLTKRFTSENDYLEVKSLKDVKHVLWSEAMKIWKIALPVALCSLFQYLTNTSTSIYAGHLGDIELSSFSLYQSILN

CIYSLLFGMSTALATLCGQAYGARQFQSAGIYLQRSWIVLFTTCIFLLPIHLYATLILKLLGQEKEIADLAGKYAILLIP

YMFSFAVNLPLVKFLQAQSKVNVIMYIAMVTLLIQNVLLYIFISVFDWGMIGLAMASNISGWIFSIALVIYVIGWSKEGW

NGLSWMAFRELWEFTKLSFGSSVMICLEQWYTTIIILLAGYLDNPVIALGSYSICLNVQGWNSMLLLGVSTALSVRVSHT

LGMSHPRASKYSFLVAMSQSLLIGIIFMTVIFLSKEKFAFIFTNSDDMVHAASELAYLLGITMVINSISQTISGVVIGCG

WQVMVGYINLACYYIVGLPIGIYLGFNQHLGVKGLWGGTMCGNILQILVLVVIIYKTNWTNEVEQTANRMRIWSSNKVQK

DII

>MsMATE56

METPLVNKSFTSENDYLPLKNLKDVKYVLWNETLKIWKIAIPVALSLLFQNLIGSSNSIYAGHIGDIQLSSYSLYQSVIT

TIYFSILYGMSNALATLCGQAYGAGKFQNAGIYLQRSWIVLFTTCIFLLPIHLYATLILKLLGQEKEIADLAGKYAILLI

PYMFSFAVNLPLVKFLQAQSKVNVIMYISMVTLLIQNVQLYIFISVFDWGITGLAMASNITGWGFAIAQLIYAIGWCKEG

WNGLSWMAFRELWEFTKLSLGSSVMICLEQWYTTIIILLAGYLDNPVIALGSYSICLNIQGWNAMLLLGLSTALSVRVSN

TLGMSHPRASKYSFLVAMSQSLLIGTIFMTVIFLSKEKFALIFTKSDDLIHAASELAYLLGITMVINSISQTISGVVIGC

GWQVMVGYINLACYYIVGLPIGIFLGFNQHLGVKGLWGGTMCGNILQILVLVVIIYKTNWTNEVEQTANRMRIWSSNKVQ

KDII

>MsMATE57

METPLLTKRFTSENDYLEVKTLKDVKHVLWSETGKIWKIALPVALCSLFQYLTNTSTSIYAGHLGDIQLSSYSLYQSVIT

TIYFSILYGMSNALATLCGQAYGAGKFQNAGIYLQRSWIVLFTTCILLLPINIYATPILKFLGQEQEIAGLAGKYAILIT

PYMFSIAINLPIQKFLQAQSEVKVIMYIAIVILLVQNGLLYIFISVFDWGMIGLAMASNISGWIFSIALVIYVIGWSKEG

WNGLSWMAFRELWEFTKLSFGSSVMICLEQWYTACIILLAGHLDNPVIAVGSFSICLNIQGWNSMLLLGVSTALSVRVSH

TLGMSHPRASKYSFLVAMSQSLLIGIIFMTVIFLSKEKFAIIFTNSDDLIHAASELAYFLGITMVLNSISQTISGVVIGC

GWQVMVGYINLACYYIVGLPIGIFLGFNQHLGVKGLWGGTMCGNILQILVLIVIIYKTNWTKEVEQTANRMRIWSSNNLQ

NDVI

>MsMATE58

METPLLTKRFTSENDYLEVKTLKDVKHVLWSETRKIWKIALPVALCSLFQYLTNTSTSIYAGHLGDIELSSFSLYQSIIN

CIYSLLFGMSTALATLCGQAYGARQFQSAGIYLQRSWIILFTTCILLLPINIYATPILKFLGQEQEIADLAGKYAIQITP

YMFSIAINLPIKKFLQAQSKVKVIMYIAIVILLIQNGLLYIFISVFDWGITGLAMASNITGWRFAIAQLIYAIGWSKEGW

NGLSWMAFRELWEFTKLSFGSSVMICLEQWYTTIIILLAGYLDNPVIALGSYSICLNVQGWNSMLLLGVSTALSVRVSNT

LGMSHPRASKYSFLVAMSQSLLIGIIFMTVIFLSKQKFAIIFTKSEDMIHAASELAYLLGITMVINSISQTISGVVIGCG

WQVMVGYINLACYYIVGLPIGIYLGFNQHLGVKGLWGGTICASILQIIVLTIIICKTNWTKEVEQTANRMQIWISNK

>MsMATE59

METPLLTKRFTSENDYLEVKTLKDVKHVLWSETGKIWKIALPVALCSLFQYLTNTSTSIYAGHLGDIELSSFSLYQSIIN

CIYSLLFGMSTALATLCGQAYGAGQFQNAGIYLQRSWIVLFTTCILLLPINIYATPILKFLGQEQEIAGLAGKYAILITP

YMFSIAINLPIQKFLQAQSEVKVIMYIAIVILLVQNGLLYIFISVFDWGITGLAMASNITGWGFAIAQLIYAIGWCKEGW

NGLSWMAFRELWEFTKLSFGSSVMICLEQWYTTIIILLAGYLDNPVIALGSYSICLNIQGWNAMLLLGLSTALSVRVSNT

LGMSHPRASKYSFLVAMSQSLLIGTIFMTVIFLSKQKFAIIFTKSEDMIHAASELAYFLGITMVLNSVSQTISGVVIGCG

WQVMVGYINLACYYIVGLPIGIFLGFNQHLGVKGLWGGTMCGNILQILVLVVIIYKTNWTNEVEQTANRMRIWSSNKVQK

DII

>MsMATE60

METPLLTKRFTSENDYLEVKTLKDVKHVLWSEAMKIWKIALPVALCSLFQYLTNTSTSIYAGHLGDIELSSFSLYQSILN

CIYSLLFGMSTAIATLCGQAYGAGQFQNAGIYVQRSCIILFTTCILLLPINIYATPILKFLGQEQEIADLAGKYAILIIP

SMFSIAFNLPIQKFLQAQSEVKVIMYIAIVILLIQNGLLYIFISVFDWGITGLAMASNITGWGFAIAQLIYAIGWCKEGW

NGLSWMAFRELWEFTKLSFGSCVMICLEQWYTTIIILLAGYLDNPVIALGSYSICLNIQGWNAMLLLGLSTALSVRVSNT

LGMSHPRASKYSFLVAMSQSLLIGTIFMTVIFLSKEKFAFIFTNSDDLIHAASELAYLLGITMVINSISQTISGVVIGCG

WQVMVGYINLACYYIVGLPIGIFLGFNQHLGVKGLWGGTICASILQIIVLTIIICKTNWTKEVEQTANRMQIWISNK

>MsMATE61

METPLLTKRFTSENDYLEVKTLKDVKHVLWSETGKIWKIALPVALCSLFQYLTNTSTSIYAGHLGDIELSSFSLYQSILN

CIYSLLFGMSTAIATLCGQAYGAGQFQNAGIYVQRSCIILFTTCILLLPINIYATPILKFLGQEQEIADLAGKYAILITP

YMFSIAINFPIQKFLQAQSEVKVIMYIAIVILLIQNGLLYIFISVFDWGITGLAMASNITGWGFAIAQLIYAIGWCKEGW

NGLSWMAFRELWEFTKLSFGSSVMICLEQWYTTIIILLAGYLDNPVIALGSYSICLNIQGWNAMLLLGLSTALSVRVSNT

LGMSHPRASKYSFLVAMSQSLLIGTIFMTVIFLSKEKFAFIFTNSDDLIHAASELAYLLGITMVINSISQTISGVVIGCG

WQVMVGYINLACYYIVGLPIGIFLGFNQHLGVKGLWGGTMCGNILQILVLVVIIYKTNWTNEVEQTANRMRIWSSNNLQN

DVI

>MsMATE62

METPLLTKRFTSENDYLEVKTLKDVKHVLWSETGKIWKIALPVALCSLFQYLTNTSTSIYAGHLGDIELSSFSLYQSILN

CIYSLLFGMSTAIATLCGQAYGAGQFQNAGIYVQRSCIILFTTCILLLPINIYATPILKFLGQEQEIAGLAGKYAILITP

YMFSIAINLPIQKFLQAQSEVKVIMYIAIVILLVQNGLLYIFISVFDWGITGLAMASNITGWGFAIAQLIYAIGWCKEGW

NGLSWMAFRELWEFTKLSFGSSVMICLEQWYTTIIILLAGYLDNPVIALGSYSICLNIQGWNAMLLLGLSTALSVRVSNT

LGMSHPRASKYSFLVAMSQSLLIGTIFMTVIFLSKEKFAFIFTNSDDLIHAASELAYLLGITMVINSISQTISGDLLGLV

SREFVIISLLVSILSISLK

>MsMATE63

MEENDTQTFPLLTPLNNQQHDQINTAVFTAKSDDISPIIGAGDFAREFLNESKKLWYLAGPAIFTSISQYSLGAVTQVFA

GQVGTLQLAAVSVENSVIAGFCLGITMGMGSALETLCGQAFGAGKLNMLGIYMQRSWLILSATALILSFLYIFASPLLKL

IGQTTAISEAAGVFALWMIPQLFAYAVNFPTQKFLQAQSKIMAMAWISAVALVGHTFFSWFLMLHLGWGLVGAAVVLNSS

WWFIVLAQIVYVLSGSCGEAWSGFSFQAFQNLWGFVRLSLASAVMMCLEVWYFMALILFAGYLENAEVSVDALSICANIL

GWTVMASFGINAAISVRVSNELGASHPRAAKFSLVVAVITSFVIGLILSLILIIFRKQYPVLFSNDPEVREVVIELTPML

ALCIVINNIQPVLSGVAIGAGWQSAVAYVNIACYYLFGIPLGLFFGYYLDFGVLGIWSGMLSGTVLQTLVLFFMVYRTDW

NNEASLAEERISKWGGQKVLKMNDNGKDIQET

>MsMATE64

MEENDTQTFPLLTPLNNQQHDQINTAVFTAKSDDISPIIGAGDFAREFLNESKKLWYLAGPAIFTSISQYSLGAVTQVFA

GQVGTLQLAAVSVENSVIAGFCLGITMGMGSALETLCGQAFGAGKLNMLGIYMQRSWLILSATALILSFLYIFASPLLKL

IGQTTAISEAAGVFALWMIPQLFAYAVNFPTQKFLQAQSKIMAMAWISAVALVGHTFFSWFLMLHLGWGLVGAAVVLNSS

WWFIVLAQIVYVLSGSCGEAWSGFSFQAFQNLWGFVRLSLASAVMMCLEVWYFMALILFAGYLENAEVSVDALSICANIL

GWTVMASFGINAAISVRVSNELGASHPRAAKFSLVVAVITSFVIGLILSLILIIFRKQYPVLFSNDPEVREVVIELTPML

ALCIVINNIQPVLSGVAIGAGWQSAVAYVNIACYYLFGIPLGLFFGYYLDFGVLRMPRFEIEKEKEERLKNHQ

>MsMATE65

MEENDTQTFPLLTPLNNQQHDQINTAVFTAKSDDISPIIGAGDFAREFLNESKKLWYLAGPAIFTSISQYSLGAVTQVFA

GQVGTLQLAAVSVENSVIAGFCLGITMGMGSALETLCGQAFGAGKLNMLGIYMQRSWLILSATALILSFLYIFASPLLKL

IGQTTAISEAAGVFALWMIPQLFAYAINFPTQKFLQAQSKIMAMAWISAVALVGHTFFSWFLMLHLGWGLVGAAVVLNSS

WWFIVLAQIVYVLSGSCGEAWSGFSFQAFQNLWGFVRLSLASAVMMCLEVWYFMALILFAGYLENAEVSVDALSIW

>MsMATE66

MDDSTQPLLTPKSKEQRHETNTNFPLPNSPPSNTAIFTAAAPDMDLITSPKDFFKQFIVESKMLWYLAGPAIFSFVSKYS

LGAVTQIFAGHVSTIDLAAVSVENSLIAGFSFGLMLGMGSALETLCGQAVGAGKLDMLGIYMQRSWVILFSMAFPLCLLY

IFAESILKFIGQTTEISEAAGTFALYMIPQLFAYALNFPVAKFLQAQSMVIVIAVISGVAMVLHPIFSWLLMVKFGWGLV

GAAVVLNGSWWFIVVAQLGYVFSGKCGIAWNGFSFEAFRNLWGFFRLSLASAVMLCLETWYFMALILFAGYLKNAEISVD

AFSICMNILGWTIMVSFGMNVAVSVRVSNELGAVHPRTARFSLVVAVITSILIGILLALVLIISRDKYPAYFTNDKEVQD

LVKDLTPLLALCVVINNVQPVLSGVAIGAGWQAAVAYVNIACYYLFGIPVGLILGYKVNLGVKGIWCGMMSGTILQTCVL

LLMVYKTNWNKEASLAEDRIRSWGGPREVTEAKEENIQET

>MsMATE67

MFMMKQLGMGSAVETLCGQAFGAKKYEMLGIYLQRSTVLLTLAGLILTIIYIFSEPILIFLGESPKIASAASLFVFGLIP

QIFAYAINFPIQKFLQAQSIVAPSAYISAATLVIHLALSYVVIYQIGLGLLGASLVLSISWWIIVIAQFVYIVKSEKCKH

TWKGFSFQAFSGLPEFFKLSAASAVMLCLETWYFQILVLLAGLLPHPELALDSLSICTTVSGWTFMISVGFQAAARFGSP

LIFIDRFLFIIG

>MsMATE68

MFMMKQLGMGSAVETLCGQAFGAKKYEMLGIYLQRSTVLLTLAGLILTIIYIFSEPILIFLGESPKIASAASLFVFGLIP

QIFAYAINFPIQKFLQAQSIVLPSAYISAGTLVFHLILSWVVVFKIGLGLLGASLVLSFSWWVIVVAQFIYILKSEKCKR

TWNGFTWEAFSGLPEFFKLSAASAVMLCLESWYFQILVLLAGLLPEPELALDSLSICTTVSGWTFMISVGFQAAASVRVS

NELGAGNSKSASFSVVVVTVISFIICAIIALVVLALRDVISYVFTDGEEVAAAVSDLSPLLALAIVLNGVQPVLSGVAVG

CGWQTFVAYVNVGCYYGIGIPLGAVLGFYFKFGAKGIWLGMLGGTVLQTIILMWVTFRTDWNNEVVESNKRLNKWEGKTE

SLLKN

>MsMATE69

MATSTHVHKDIDEPLLVPSEPTPILLSSFTHSFGSKHESDGELERILSDTSVPFVKRIRHATWVEFKLLFYLAAPAVIVY

LINYVMSMSTQIFSGHLGNLELAAASLGNNGIQIFAYGLMLGMGSAVETLCGQAFGAKKYEMLGIYLQRSTVLLTLAGLI

LTIIYIFSEPILIFLGESPKIASAASLFVFGLIPQIFAYAINFPIQKFLQAQSIVAPSAYISAATLVIHLALSYVVIYQI

GLGLLGASLVLSISWWIIVIAQFVYIVKSEKCKHTWKGFSFQAFSGLPEFFKLSAASAVMLCLETWYFQILVLLAGLLPH

PELALDSLSICTTVSGWTFMISVGFQAAASVRVSNELGARNPKSASFSVKVVTLISFLISVIAALIVLALRDVISYVFTE

GEVVAAAVSDLCPLLSLSLVLNGIQPVLSGVAVGCGWQAFVAYVNVGCYYIIGIPLGAVLGFYFNFGAKGIWLRMLGGTT

MQTIILMWVTFRTDWNKEVKEAAKRLNKWEDKKKEPLLN

>MsMATE70

MATSTHVHKDIDEPLLVPREPTPILLSSFTHSCGSKHESDGELERILSDTSVPFVKRIRHATWVEFKLLFYLAAPAVIVY

LINYVMSMSTQIFSGHLGNLELAAASLGNNGIQIFAYGLMLGMGSAVETLCGQAFGAKKYEMLGIYLQRSTVLLTIAGLI

LTIIYIFSEPILIFLGESPKIASAASLFVFGLIPQIFAYAINFPIQKFLQAQSIVAPSAYISAATLVIHLALSYVVIYQI

GLGLLGASLVLSISWWIIVIAQFVYIVKSEKCKHTWKGFSFQAFSGLPEFFKLSAASAVMLCLETWYFQILVLLAGLLPH

PELALDSLSICTTVSGWTFMISVGFQAAASVRVSNELGAGNSKSASFSVVVVTVISFIICAIIALVVLALRDVISYVFTD

GEEVAAAVSDLSPLLALAIVLNGVQPVLSGVAVGCGWQTFVAYVNVGCYYGIGIPLGAVLGFYFKFGAKGIWLGMLGGTV

LQTIILMWVTFRTDWNNEVVESNKRLNKWEGKTESLLKN

>MsMATE71

MATSTHVHKDIDEPLLVPSEPTPILLSSFTHSFGSKHESDGELERILSDTSVPFVKRIRHATWVEFKLLFYLAAPAVIVY

LINYVMSMSTQIFSGHLGNLELAAASLGNNGIQIFAYGLMLGMGSAVETLCGQAFGAKKYEMLGIYLQRSTVLLTIAGLI

LTIIYIFSEPILIFLGESPKIASAASLFVFGLIPQIFAYAINFPIQKFLQAQSIVLPSAYISAGTLVFHLILSWVVVFKI

GLGLLGASLVLSFSWWVIVVAQFIYILKSEKCKRTWNGFTWEAFSGLPEFFKLSAASAGMLCLESWYFQILVLLAGLLPQ

PELALDSLSICSTVSGWVFMISVGFNAAASVRVSNELGARNPKSASFSVKVVTLISFLISVIAALIVLALRDVISYVFTE

GEVVAAAVSDLCPLLSLSLVLNGIQPVLSGVAVGCGWQAFVAYVNVGCYYIIGIPLGAVLGFYFNFGAKGIWLGMLGGTT

MQTIILMWVTFRTDWNKEVKEAAKRLNKWEDKKKEPLLN

>MsMATE72

MASVKQNSAEEPLLFNHSGTSQKHHHESDGELERILSDTTVPFFSRIGSATWIELRLLFLLAAPAVFVYLINYVMSMSTQ

IFSGHLGNLELAAASLGNTGIQIFAYGLMLGMGSAVETLCGQAYGAEKYDMLGTYLQRSTILLTITGFFLTVIYVLSEPI

LVFIGQSPRIASAAALFVYGLIPQIFAYAVNFPIQKFLQAQSIVAPSAYISAATLVIHLALSYVVIYQIGLGLLGASLVL

SISWWIIVIAQFVYIVKSEKCKHTWKGFSFQAFSGLPEFFKLSAASAVMLCLETWYFQILVLLAGLLPHPELALDSLSIC

TTVSGWTFMISVGFQAAASVRVSNELGAGNSKSASFSVVVVTVISFIICAIIALVVLALRDVISYVFTDGEEVAAAVSDL

SPLLALAIVLNGVQPVLSGVAVGCGWQTFVAYVNVGCYYGIGIPLGAVLGFYFKFGAKGIWLGMLGGTVLQTIILMWVTF

RTDWNNEVVESNKRLNKWEGKTESLLKN

>MsMATE73

MASVKQNSAEEPLLFNHSGTSQKHHHESDGELERILSDTTVPFFSRIGSATWIELRLLFLLAAPAVFVYLINYVMSMSTQ

IFSGHLGNLELAAASLGNTGIQIFAYGLMLGMGSAVETLCGQAYGAEKYDMLGTYLQRSTILLTITGFFLTVIYVLSEPI

LVFIGQSPRIASAAALFVYGLIPQIFAYAVNFPIQKFLQAQSIVAPSAYISAATLVIHLVLSYVVIYQIGLGLLGASLVL

SISWWIIVIAQFVYIVKSEKCKHTWQGFSFQAFSGLPEFFKLSAASAVMLCLETWYFQILVLLAGLLPHPELALDSLSIC

TTVSGWTFMISVGFQAAASVRVSNELGARNPKSASFSVKVVTLISFLISVIAALIVLALRDVISYVFTEGEVVAAAVSDL

CPLLSLSLVLNGIQPVLSGVAVGCGWQAFVAYVNVGCYYIIGIPLGAVLGFYFNFGAKGIWLGMLGGTTMQTIILMWVTF

RTDWNKEVKDNFAIISFL

>MsMATE74

MASVKQNSAEEPLLFNHSGTSQKQHESDGELERILSDTTVPFFSRIGSATWIELRLLFLLAAPAVFVYLINYVMSMSTQI

FSGHLGNLELAAASLGNTGIQIFAYGLMLGMGSAVETLCGQAYGAEKYDMLGTYLQRSTILLTITGFFLTVIYVLSEPIL

VFIGQSPRIASAAALFVYGLIPQIFAYAVNFPIQKFLQAQSIVAPSAYISAATLVIHLALSYVVIYQIGLGLLGASLVLS

ISWWIIVIAQFVYIVKSEKCKHTWKGFSFQAFSGLPEFFKLSAASAVMLCLETWYFQILVLLAGLLPHPELALDSLSICT

TVSGWTFMISVGFNAAARFGSPLIFIDRFLFIIG

>MsMATE75

MASVKQNSAEEPLLFNHSGTSQKHHHESDGELERILSDTTVPFFSRIGSATWIELRLLFLLAAPAVFVYLINYVMSMSTQ

IFSGHLGNLELAAASLGNTGIQIFAYGLMLGMGSAVETLCGQAYGAEKYDMLGTYLQRSTILLTITGFFLTVIYVLSEPI

LVFIGQSPRIASAAALFVYGLIPQIFAYAVNFPIQKFLQAQSIVLPSAYISAGTLVFHLILSWVVVFKIGLGLLGASLVL

SFSWWVIVVAQFIYILKSEKCKRTWNGFTWEAFSGLPEFFKLSAASAGMLCLESWYFQILVLLAGLLPQPELALDSLSIC

STVSGWVFMISVGFNAAASVRVSNELGARNPKSASFSVKVVTLISFLISVIAALIVLALRDVISYVFTEGEVVAAAVSDL

CPLLSLSLVLNGIQPVLSGVAVGCGWQAFVAYVNVGCYYIIGIPLGAVLGFYFNFGAKGIWLGMLGGTTMQTIILMWVTF

RTDWNKEVKEAAKRLKRQLCNYFISMMTLCRKWENHCYCV

>MsMATE76

MASVKQNSAEEPLLFNHSGTSQKHHHESDGELERILSDTTVPFFSRIGSATWIELRLLFLLAAPAVFVYLINYVMSMSTQ

IFSGHLGNLELAAASLGNTGIQIFAYGLMLGMGSAVETLCGQAYGAEKYDMLGTYLQRSTILLTITGFFLTVIYVLSEPI

LVFIGQSPRIASAAALFVYGLIPQIFAYAVNFPIQKFLQAQSIVLPSAYISAGTLVFHLILSWVVVFKIGLGLLGASLVL

SFSWWVIVVAQFIYILKSEKCKRTWNGFTWEAFSGLPEFFKLSAASAVMLCLESWYFQILVLLAGLLPQPELALDSLSIC

TTVSGWVFMISVGFNAAASVRVSNELGARNPKSASFSVKVVTLISFLISVIAALIVLALRDVISYVFTEGEVVAAAVSDL

CPLLSLSLVLNGIQPVLSGVAVGCGWQAFVAYVNVGCYYIIGIPLGAVLGFYFNFGAKGIWLGMLGGTTMQTIILMWVTF

RTDWNKEVKEAAKRLNKWEDKKKEPLLN

>MsMATE77

MASVKQNSAEEPLLFNHSGTSQKQHESDGELERILSDTTVPFFSRISSATWIELKLLFLLAAPAVFVYLINYVMSMSTQI

FSGHLGNLELAAASLGNTGIQIFAYGLMLGMGSAVETLCGQAYGAEKYDMLGTYLQRSTILLTITGFFLTVIYVLSEPIL

VFIGQSPRIASAAALFVYGLIPQIFAYAVNFPIQKFLQAQSIVLPSAYISAGTLVFHLILSWVVVFKIGLGLLGASLVLS

FSWWVIVVAQFIYILKSEKCKRTWNGFTWEAFSGLPEFFKLSAASAVMLCLESWYFQILVLLAGLLPEPELALDSLSICT

TVSGWVFMISVGFNAAASVRVSNELGARNPKSASFSVKVVTLISFLISVIAALIVLALRDVISYVFTEGEVVAAAVSDLC

PLLSLSLVLNGIQPVLSGVAVGCGWQAFVAYVNVGCYYIIGIPLGAVLGFYFNFGAKGIWLGMLGGTTMQTIILMWVTFR

TDWNKEVKEAAKRLNKWEDKKKEPLLN

>MsMATE78

MGSINRENDTDHNLIQSLLSKEISSINEHQHEDEDEQEFGKKLWIETKKLWHIVGPSIFSRVASFTMNVVTQAFAGHLGD

VQLASISIANTVIVGFNFGLLLGMASALETLCGQAFGAKKHNLLGIYLQRSWIVLFLCCFLLLPFYIFATPILKLLGQPD

DVAEWSGVVAIWLIPLHFSFAFQFPLQRFLQCQLKTGVIAWVSLVGLVVNVVLSWLLIFVWDFGLIGAAIALDVSWWILV

FGMLAYTVCGGCPLTWTGFSIEAFSGLWDFFKLSFASGVMLCRELKISKIKHMHWYVCRSQLGHVLRDGGSIIYGWRSNV

GDLCSLENWYYRILLLMTGQLENATVAVDALSVCMTINGWEMMIPLAFFAGTGVRVANELGAGKGKSAKFAMQVSVAQST

VIGFIFCILIMIFHRQFAYIFTTSPPVLEAVNDMSILLAVTILLNSVQPILSGVAVGSGWQVFVAYVNIGCYYLIGLPLG

ILMGWVFNTGVEGIWGGMIFGGTAIQTLILIIVTARCDWENEAEKARSRVNKWSATKPDDQLQITE

>MsMATE79

MGSINRENDTDHNLIQSLLSKEISSINEHQHEDEDEQEFGKKLWIETKKLWHIVGPSIFSRVASFTMNVVTQAFAGHLGD

VQLASISIANTVIVGFNFGLLLGMASALETLCGQAFGAKKHNLLGIYLQRSWIVLFLCCFLLLPFYIFSTPILKLLGQPD

DVAEWSGVVAIWLIPLHFSFAFQFPLQRFLQCQLKTGVIAWVSLVGLVVNVVLSWLLIFVWDFGLIGAAIALDVSWWILV

FGMLAYTVCGGCPLTWTGFSIEAFSGLWDFFKLSFASGVMLCLENWYYRILLLMTGQLENATVAVDALSVCMTINGWEMM

IPLAFFAGTGVRVANELGAGKGKSAKFAMQVSVAQSTVIGFIFCILIMIFHRQIAYIFTTSPPVLEAVNDMSILLAVTIL

LNSVQPILSGVAVGSGWQVFVAYVNIGCYYLIGLPLGILMGWVFNTGVEGIWGGMIFGGTAIQTLILIIVTARCDWENEA

EKARSRVNKWSATKPDDQLQITE

>MsMATE80

MPSKELASPLGETKVPLLSPNHLSTTNEEEEPQDLTRKVWIESKKLWHIVGPAIFSRIASYMMLVITQAFAGHLGDLELA

AISIANNVVVGFDFGLLLGMASALETLCGQAFGAKQYYMLGVYMQRSWIVLFICCIFLLPIYLFATPVLRLLGQPEDLAV

LSGQVSVWLIPLHFAFAFQFPLNRFLQSQLKTAAIAWVSLFALLVHVFVSWLFVFKFQFGVIGTAATLNFSWWALTVGLF

CYTVYGGCPLTWNGFSMEAFSGLWEFVKLSAASGVMLCLENWYYRILILMTGNLPNAEIAVDALSICMTINGLEMMIPLA

FFAATGVRVANELGAGNGKGAKFATIVSVLTSLIIGLFFWMLIMIFHDKFGYIFSTSKPVLDEVSKLSLLLAFTILLNSV

QPVLSGVAVGSGWQSYVAYINLGCYYMIGVPLGFLMGWYFDQGVMGIWAGMIFGGTATQTLILCLITLRCDWDKEAEKAK

LHITKWSNRKQQLS

>MsMATE81

MPSKELASPLGETKVPLLSPNHLSTTNEEEEPQDLTRKVWIESKKLWHIVGPAIFSRIASYMMLVITQAFAGHLGDLELA

AISIANNVVVGFDFGLLLGMASALETLCGQAFGAKQYYMLGVYMQRSWIVLFICCIFLLPIYLFATPVLRLLGQPEDLAV

LSGQVSMWLIPLHFAFAFQFPLNRFLQSQLKTAAIAWVSLFALLVHVFVSWLFVFKFQFGVIGTAATLNFSWWALTVGLF

CYTVYGGCPLTWNGFSMEAFSGLWEFVKLSAASGVMLCLENWYYRILILMTGNLPNAEIAVDALSICMTINGLEMMIPLA

FFAATGVRVANELGAGNGKGAKFATIVSVLTSLIIGLFFWMLIMIFHDKFGYIFSTSKPVLDEVSKLSLLLAFTILLNSV

QPVLSGVAVGSGWQSYVAYINLGCYYMIGVPLGFLMGWYFDQGVMGIWAGMIFGGTATQTLILCLITLRCDWDKEVNLTS

YLSIYSTF

>MsMATE82

MPSKELASPLGETKVPLLSPNHLSTTNEEEEPQDLTRKVWIESKKLWHIVGPAIFSRIASYMMLVITQAFAGHLGDLELA

AISIANNVVVGFDFGLLLGMASALETLCGQAFGAKQYYMLGVYMQRSWIVLFICCIFLLPIYLFATPVLRLLGQPEDLAV

LSGQVSMWLIPLHFAFAFQFPLNRFLQSQLKTAAIAWVSLFALLVHVFVSWLFVFKFQFGVIGTAATLNFSWWALTVGLF

CYTVYGGCPLTWNGFSMEAFSGLWEFVKLSAASGVMLCLENWYYRILILMTGNLPNAEIAVDALSICMTINGLEMMIPLA

FFAATGVRVANELGAGNGKGAKFATIVSVLTSLIIGLFFWMLIMIFHDKFGYIFSTSKPVLDEVSKLSLLLAFTILLNSV

QPVLSGITLTINISNSNI

>MsMATE83

MPSKELASPLGETKVPLLSPNHLSTTNEEEEPQDLTRKVWIESKKLWHIVGPAIFSRIASYMMLVITQAFAGHLGDLELA

AISIANNVVVGFDFGLLLGMASALETLCGQAFGAKQYYMLGVYMQRSWIVLFICCIFLLPIYLFATPVLRLLGQPEDLAV

LSGQVSMWLIPLHFAFAFQFPLNRFLQSQLKTAAIAWVSLFALLVHVFVSWLFVFKFQFGVIGTAATLNFSWWALTVGLF

CYTVYGGCPLTWNGFSMEAFSGLWEFVKLSAASGVMLCLENWYYRILILMTGNLPNAEIAVDALSICMTINGLEMMIPLA

FFAATGVRVANELGAGNGKGAKFATIVSVLTSLIIGLFFWMLIMIFHDKFGYIFSTSKPVLDEVSKLSLLLAFTILLNSV

QPVLSGVAVGSGWQSYVAYINLGCYYMIGVPLGFLMGWYFDQGVMGIWAGMIFGGTATQTLILCLITLRCDWDKEAEKAK

LHITKWSNRKQQLS

>MsMATE84

MGSEELNKNLLQHQNPSEEEEEPLRKRVWEESKKLWIVAGPAIFNRFSTFGIMVVAQSFIGHIGSTELAAYALVMTVLVR

FANGILLGMASALETLCGQAYGAKQYDMLGVYLQRSWIVIFLTSILLLPIYIFTKPILVALGQDENIAQVAGSISIWSIG

IVFAFSASFTCQMFLQAQSKNKIIAYLAAVSISIHVFMSWLLTVKFKFGLNGAMTSILLAYWMPNLGQLVFIMTKCPDTW

KGFSFLAFKDLWPVIKLSLSSGAMLCLEIWYNTVLILLTGNMENAEISIDALAICLNINGWEMMIALGFFAAASVRVSNE

LGRGSSKAAKFSIVITVLTSFSIGFVLFLIFLFLRGRLAYIFTPNPDVANAVGDLSPLLSFSILMNSVQPVLSGVSVGAG

WQSVVAYVNIGCYYLIGIPIGVVLGNLLHLQVKGVWIGMLFGTFVQTIMLMIITFKTDWDKQVEIARNRVNKWAVVENDE

SNNTSRISN

>MsMATE85

MEGDHDPKKKLLDEEEEELSLVKKMWKESKLMWVVAGPAIFTRFSSFGVQIITQSFVGHIGSTELAAYSLVFTVLVRFVN

GILLGMASALATLCGQAYGAKEYGMMGXXLQRSWIVLSLTALILLPLFIFTTPILIILGQDETIAQVAGTIGYWSIPILF

AFIASFTTQTFLQSQSRNIIIAYLAAFSISVHVLLSWLLTMKIKLGIVGAMISISLALWIPNIGQLIFITCGWCSDTWKG

FSFLALQDLWPVVKLSLSSGFMLCLELWYNTVLILLTGNMENAEIQIDALSICLNINGWEMMISLGFMAAASVRVANELG

KGSAKDAKFAVNMIVLTSFTIGFLLFLFFLFFRERLAYIFTTNKDVASAVGDLSPLLAVSILLNSVQPVLSGVAIGAGWQ

SIVAYVNLGCYYIIGIPVGIVLGKVYHLQVKGIWIGMLFGTLMQTIILLIISYKTDWDKQVTIARNRINKWSKVDPDHET

VASDN

>MsMATE86

MERDLKQNLLLKKSEQEENELSLGKRVWNETKLMWVVAAPAIFTRFSTFGIQIISQAFVGHIGSRELAAFALVFTVLIRF

ANGILLGMATALATLCGQAYGAKEYGMMGVYLQRSWIVLFLTALVLLPVFVFTTPILTLLGQDESISEVAGNISLWSIPI

MFAFIVSFTCQTFLQSQSKNTIIAFLAAFSIIIHAFLSWLLTMKYEFGIAGAMISTILAYWIPNIGQLIFVTCGWCPETW

QGFSFLAFKDLWPVVKLSLSAGAMLCLELWYNTILVLLTGNMKNAEVEIDALSICLNINGWEMMISLGFMAAASVRVSNE

LGKGSAKAAKFSIVVTVLTSLAIGSFLFLFFLFFRERLAYIFTSNKEVAAAVGELSPLLSISILLNSVQPVLSGVAIGAG

WQSTVAYVNIGCYYIIGIPVGVVLGNVIHWQVKGIWMGMLFGTLIQTIVLLIITYKTNWDEQVTVARKRVNRWSNVDSTD

QETKTNLIEK

>MsMATE87

MERDLKQNLLLKKSEQEENEQEELSLGKRVWNETKLMWVVAAPAIFTRFSTFGIQIISQAFVGHIGSRELAAFALVFTVL

IRFANGILLGMATALATLCGQAYGAKEYGMMGVYLQRSWIVLFLTALVLLPVFVFTTPILTLLGQDESISEVAGNISLWS

IPIMFAFIVSFTCQTFLQSQSKNTIIAFLAAFSIIIHAFLSWLLTMKYEFGIAGAMISTILAYWIPNIGQLIFVTCGWCP

ETWQGFSFLAFKDLWPVVKLSLSAGAMLCLELWYNTILVLLTGNMKNAEVEIDALSICLNINGWEMMISLGFMAAASVRV

SNELGKGSAKAAKFSIVVTVLTSLAIGSFLFLFFLFFRERLAYIFTSNKEVAAAVGELSPLLSISILLNSVQPVLSGVAI

GAGWQSTVAYVNIGCYYIIGIPVGVVLGNVIHWQVKGIWMGMLFGTLIQTIVLLIITYKTNWDEQVTVARKRVNRWSKVD

STDQETKTNLIEK

>MsMATE88

MKQEELSLGKRVWNETKLMWVVAAPAIFTRFSTFGIQIISQAFVGHIGSRELAAFALVFTVLIRFANGILLGMATALATL

CGQAYGAKEYGMMGVYLQRSWIVLFLTALVLLPVFVFTTPILTLLGQDESISEVAGNISLWSIPIMFAFIVSFTCQTFLQ

SQSKNTIIAFLAAFSIIIHAFLSWLLTMKYEFGIAGAMISTILAYWIPNIGQLIFVTCGWCPETWQGFSFLAFKDLWPVV

KLSLSAGAMLCLELWYNTILVLLTGNMKNAEVEIDALSICLNINGWEMMISLGFMAAASVRVSNELGKGSAKAAKFSIVV

TVLTSLAIGSFLFLFFLFFRERLAYIFTSNKEVAAAVGELSPLLSISILLNSVQPVLSGVAIGAGWQSTVAYVNIGCYYI

IGIPVGVVLGNVIHWQVKGIWMGMLFGTLIQTIVLLIITYKTNWDEQVTVARKRVNRWSKVDSTDQETKTNLIEK
