# Supplementary material for: Transcriptome-wide characterization and functional analysis of MATE transporters in response to aluminum toxicity in Medicago sativa L
Source: PeerJ. 2019 Jan 31;7:e6302. doi: 10.7717/peerj.6302 (PMC6360082; doi:10.7717/peerj.6302)
Supplement: Supplemental Information 10 [file peerj-07-6302-s010.doc]

**Supplementary data 2.** Polypeptide sequences of MATE proteins identified from *Arabidopsis*, rice and other plants

**The protein sequences of 57 *Arabidopsis* MATEs**

>AT4G29140.1

MCNPSTTTTTTGSENQESRTGLFLDLFSINSFEPTKRNLRHCENRGSPLMAEAVTEAKSLFTLAFPIAVTALVLYLRSAVSMFFLGQLG

DLELAAGSLAIAFANITGYSVLSGLALGMEPLCSQAFGAHRFKLLSLTLHRTVVFLLVCCVPISVLWFNVGKISVYLHQDPDIAKLAQTYLIFSLPDLLT

NTLLHPIRIYLRAQGIIHPVTLASLSGAVFHLPANLFLVSYLRLGLTGVAVASSITNIFVVAFLVCYVWASGLHAPTWTDPTRDCFRGWAPLLRLAGPSC

VSVCLEWWWYEIMIVLCGLLVNPRSTVAAMGVLIQTTSFLYVFPSSLSFAVSTRVGNELGANRPKTAKLTATVAIVFAAVTGIIAAAFAYSVRNAWGRIF

TGDKEILQLTAAALPILGLCEIGNCPQTVGCGVVRGTARPSTAANVNLGAFYLVGMPVAVGLGFWAGIGFNGLWVGLLAAQISCAGLMMYVVGTTDWESE

AKKAQTLTCAETVENDIIKAVVASTIDGECDEAEPLIRITVLY

>AT5G49130.1

MVVEEDSRLINLQHKYNPTMPEVVEELKRIWDISFPVAAMSILNYLKNMTSVVCMGRLGSLELAGGALAIGFTNITGYSVLSGLATGME

PLCGQAIGSKNPSLASLTLKRTIFLLLLASLPISLLWLNLAPLMLMLRQQHDITRVASLYCSFSLPDLLANSFLHPLRIYLRCKGTTWPLMWCTLVSVLL

HLPITAFFTFYISLGVPGVAVSSFLTNFISLSLLLCYIYLENNNNDKTTSKSLCLDTPLMLYGSRDSGENDVWSTLVKFAVPSCIAVCLEWWWYEFMTVL

AGYLPEPKVALAAAAIVIQTTSLMYTIPTALSAAVSTRVSNELGAGRPEKAKTAATVAVGAAVAVSVFGLVGTTVGREAWGKVFTADKVVLELTAAVIPV

IGACELANCPQTISCGILRGSARPGIGAKINFYAFYVVGAPVAVVLAFVWGLGFMGLCYGLLGAQLACAISILTVVYNTDWNKESLKAHDLVGKNVISPN

VDQIIVKCEEGLH

>AT2G04090.1

MEDPLLLGDDQLITRNLKSTPTWWMNFTAELKNVSSMAAPMATVTVSQYLLPVISVMVAGHCGELQLSGVTLATAFANVSGFGIMYGLV

GALETLCGQAYGAKQYTKIGTYTFSAIVSNVPIVVLISILWFYMDKLFVSLGQDPDISKVAGSYAVCLIPALLAQAVQQPLTRFLQTQGLVLPLLYCAIT

TLLFHIPVCLILVYAFGLGSNGAALAIGLSYWFNVLILALYVRFSSACEKTRGFVSDDFVLSVKQFFQYGIPSAAMTTIEWSLFELLILSSGLLPNPKLE

TSVLSICLTTSSLHCVIPMGIGAAGSTRISNELGAGNPEVARLAVFAGIFLWFLEATICSTLLFTCKNIFGYAFSNSKEVVDYVTELSSLLCLSFMVDGF

SSVLDGVARGSGWQNIGAWANVVAYYLLGAPVGFFLGFWGHMNGKGLWIGVIVGSTAQGIILAIVTACLSWEEQAAKARERIVGRTLE

>AT4G23030.1

MAAPLLMIIKNQTDHRQDPNPNPTHLSSSIQEAKSIAKISLPLILTGLLLYSRSMISMLFLGRLNDLSALSGGSLALGFANITGYSLLS

GLSIGMEPICVQAFGAKRFKLLGLALQRTTLLLLLCSLPISILWLNIKKILLFFGQDEEISNQAEIFILFSLPDLILQSFLHPIRIYLRSQSITLPLTYS

AFFAVLLHIPINYLLVSSLGLGLKGVALGAIWTNVNLLGFLIIYIVFSGVYQKTWGGFSMDCFKGWRSLMKLAIPSCVSVCLEWWWYEIMILLCGLLLNP

QATVASMGILIQTTALIYIFPSSLSISVSTRVGNELGANQPDKARIAARTGLSLSLGLGLLAMFFALMVRNCWARLFTDEEEIVKLTSMVLPIIGLCELG

NCPQTTLCGVLRGSARPKLGANINLCCFYFVGMPVAVWLSFFSGFDFKGLWLGLFAAQGSCLISMLVVLARTDWEVEVHRAKELMTRSCDGDEDDGNTPF

LLDSLDIEENLVF

>AT5G38030.1

MEEDKILTETLLSAAEEPPALPFSSVEDIPPITTVGGFVKEFNVEVKKLWYLAGPAIFMSITQYSLGAATQVFAGHISTIALAAVSVEN

SVIAGFSFGVMLGMGSALETLCGQAFGAGKLSMLGVYLQRSWVILNVTAVILSLLYIFAAPILAFIGQTPAISSATGIFSIYMIPQIFAYAVNYPTAKFL

QSQSKIMVMAAISAVALVLHVLLTWFVIEGLQWGTAGLAVVLNASWWFIVVAQLVYIFSGTCGEAWSGFSWEAFHNLWSFVRLSLASAVMLCLEVWYLMA

VILFAGYLKNAEISVAALSICMNILGWTAMIAIGMNAAVSVRVSNELGAKHPRTAKFSLLVAVITSTVIGLAISIALLIFRDKYPSLFVGDEEVIIVVKD

LTPILAVSIVINNVQPVLSGVAVGAGWQAVVAYVNIVCYYVFGIPFGLLLGYKLNFGVMGIWCGMLTGTVVQTIVLTWMICRTNWDTEAAMAEGRIREWG

GEVSDQLLN

>AT1G15170.1

MGDAESTKDRLLLPVERVENVTWSDLRDGSFTVELKRLIFFAAPMAAVVIAQFMLQIVSMMMVGHLGNLSLASASLASSFCNVTGFSFI

IGLSCALDTLSGQAYGAKLYRKLGVQTYTAMFCLALVCLPLSLIWFNMEKLLLILGQDPSIAHEAGKYATWLIPGLFAYAVLQPLTRYFQNQSLITPLLI

TSYVVFCIHVPLCWFLVYNSGLGNLGGALAISLSNWLYAIFLGSFMYYSSACSETRAPLSMEIFDGIGEFFKYALPSAAMICLEWWSYELIILLSGLLPN

PQLETSVLSVCLQTISTMYSIPLAIAAAASTRISNELGAGNSRAAHIVVYAAMSLAVIDALIVSMSLLIGRNLFGHIFSSDKETIDYVAKMAPLVSISLM

LDALQGVLSGIARGCGWQHIGAYINLGAFYLWGIPIAASLAFWIHLKGVGLWIGIQAGAVLQTLLLALVTGCTNWESQADKARNRMALAYGT

>AT2G38510.1

MQVGEEMASLTKIACPIVMTSLLIFSRSIISMWFLSHLGKVELAGGALAMGFGNITGVSVLKGLSVGMDPICGQAFGAKRWTVLSHTFQ

KMFCLLIVVSVPIAVTWLNIEPIFLRLGQDPDITKVAKTYMLFFVPELLAQAMLHPLRTFLRTQGLTSPLTISAIVSILLHPLFNYVFVVRMRLGVKGVA

IAMAFNTMNIDVGLLVYTCFSDSLIKPWEGLALRSLFRGWWPLLSLAAPSAISVCLEYWWYEIMLFLCGLLGNPKASVAAMGILIQTTGILYVVPFAISS

AIATRVGHALGGGQPTRAQCTTVIGLILAVAYGLAAAVFVTALRSVWGKMFTDEPEILGLISAALPILGLCEIGNSPQTAACGVLTGTARPKDGARVNLC

AFYIVGLPVAVTTTFGFKVGFRGLWFGLLSAQMTCLVMMLYTLIRTDWSHQVKRAEELTSAAADKSHSEDETVHAEVQDDDDVSSNDLEIGLLQNTN

>AT4G39030.1

MLIKSQRLTLFSPLLSKTRRIPVNSHQTLVAESVITRRTLGAITATPSFHKNPVVIRRRIKLERVTRNCVRIDREIDEEEEEEEKERGD

LVKQSIWEQMKEIVKFTGPAMGMWICGPLMSLIDTVVIGQGSSIELAALGPGTVLCDHMSYVFMFLSVATSNMVATSLAKQDKKEAQHQISVLLFIGLVC

GLMMLLLTRLFGPWAVTAFTRGKNIEIVPAANKYIQIRGLAWPFILVGLVAQSASLGMKNSWGPLKALAAATIINGLGDTILCLFLGQGIAGAAWATTAS

QIVSAYMMMDSLNKEGYNAYSFAIPSPQELWKISALAAPVFISIFSKIAFYSFIIYCATSMGTHVLAAHQVMAQTYRMCNVWGEPLSQTAQSFMPEMLYG

ANRNLPKARTLLKSLMIIGATLGLVLGVIGTAVPGLFPGVYTHDKVIISEMHRLLIPFFMALSALPMTVSLEGTLLAGRDLKFVSSVMSSSFIIGCLTLM

FVTRSGYGLLGCWFVLVGFQWGRFGLYLRRLLSPGGILNSDGPSPYTVEKIKSI

>AT1G33080.1

MARREGEVTETLLKKSTENRGEDRDGLGMKEKVWRESKKLWVVAGPAIFTRFSTSGLSLISQAFIGHLGSTELAAYSITLTVLLRFSNG

ILLGMASALETLCGQAYGAKQYHMLGIYLQRSWIVLTGCTICLMPIYIFAGPILLALGQEERLVRVARIIALWVIGINISFVPSFTCQMFLQAQSKNKII

AYVAAVSLGVHVFLSWLLVVHFDFGIAGAMTSSLVAHWLPNIAQVLFVTCGGCTETWRGFSWLAFKDLWPVFKLSVSSGGMICLELWYNSILILLTGNLK

NAEVALNALAICININALEMMVAFGFMAAASVRVSNEIGSGNSNGAKFATMVVVSTSLSIGIIFFFIFLFLRERVSYIFTTSEAVATQVADLSPLLAFSI

LLNSIQPVLSGVAVGAGWQKYVTVVNLACYYLVGIPSGLFLGYVVGLQVKGVWLGMIFGIFVQTCVLTVMTMRTDWDQQVSSSLKRLNRWVEPESPSRNQ

TLQNE

>AT3G23560.1

MADPATSSPLLDDHVGGEDERGRRSRSSTLVQKVIDVEEAKAQMIYSLPMILTNVFYYCIPITSVMFASHLGQLELAGATLANSWATVS

GFAFMVGLSGSLETLCGQGFGAKRYRMLGVHLQSSCIVSLVFSILITIFWFFTESIFGLLRQDPSISKQAALYMKYQAPGLLAYGFLQNILRFCQTQSII

APLVIFSFVPLVINIATAYVLVYVAGLGFIGAPIATSISLWIAFLSLGTYVMCSEKFKETWTGFSLESFRYIVINLTLSLPSAAMVCLEYWAFEILVFLA

GVMPNPEINTSLVAICVNTEAISYMLTYGLSAAASTRVSNELGAGNVKGAKKATSVSVKLSLVLALGVVIVLLVGHDGWVGLFSDSYVIKEEFASLRFFL

AASITLDSIQGVLSGVARGCGWQRLVTVINLATFYLIGMPIAAFCGFKLKFYAKGLWIGLICGIFCQSSSLLLMTIFRKWTKLNVATV

>AT3G08040.1

MTETGDDLATVKKPIPFLVIFKDLRHVFSRDTTGREILGIAFPAALALAADPIASLIDTAFVGRLGAVQLAAVGVSIAIFNQASRITIF

PLVSLTTSFVAEEDTMEKMKEEANKANLVHAETILVQDSLEKGISSPTSNDTNQPQQPPAPDTKSNSGNKSNKKEKRTIRTASTAMILGLILGLVQAIFL

IFSSKLLLGVMGVKPNSPMLSPAHKYLSIRALGAPALLLSLAMQGIFRGFKDTKTPLFATVVADVINIVLDPIFIFVLRLGIIGAAIAHVISQYFMTLIL

FVFLAKKVNLIPPNFGDLQFGRFLKNGLLLLARTIAVTFCQTLAAAMAARLGTTPMAAFQICLQVWLTSSLLNDGLAVAGQAILACSFAEKDYNKVTAVA

SRVLQMGFVLGLGLSVFVGLGLYFGAGVFSKDPAVIHLMAIGIPFIAATQPINSLAFVLDGVNFGASDFAYTAYSMVGVAAISIAAVIYMAKTNGFIGIW

IALTIYMALRAITGIARMATGTGPWRFLRGRSSSSSS

>AT3G59030.1

MSSTETYEPLLTRLHSDSQITERSSPEIEEFLRRRGSTVTPRWWLKLAVWESKLLWTLSGASIVVSVLNYMLSFVTVMFTGHLGSLQLA

GASIATVGIQGLAYGIMLGMASAVQTVCGQAYGARQYSSMGIICQRAMVLHLAAAVFLTFLYWYSGPILKTMGQSVAIAHEGQIFARGMIPQIYAFALAC

PMQRFLQAQNIVNPLAYMSLGVFLLHTLLTWLVTNVLDFGLLGAALILSFSWWLLVAVNGMYILMSPNCKETWTGFSTRAFRGIWPYFKLTVASAVMLCL

EIWYNQGLVIISGLLSNPTISLDAISICMYYLNWDMQFMLGLSAAISVRVSNELGAGNPRVAMLSVVVVNITTVLISSVLCVIVLVFRVGLSKAFTSDAE

VIAAVSDLFPLLAVSIFLNGIQPILSGVAIGSGWQAVVAYVNLVTYYVIGLPIGCVLGFKTSLGVAGIWWGMIAGVILQTLTLIVLTLKTNWTSEVENAA

QRVKTSATENQEMANAGV

>AT1G33110.1

MAGGGGELTAALLKKTAENGGEEKDELGLKQKVWIESKKLWIVAAPAIFTRFSTFGVSIISQSFIGHLGPIELAAYSITFTVLLRFSNG

ILLGMASALETLCGQAYGAKQNHMLGIYLQRSWIVLTGCTICLTPVYIFSGPILLALGQEERIVRVARIIALWVIGINFSFVPSFTCQMFLQAQSKNKII

AYVAAVSLGVHVFLSWLLMVHFNFGITGAMTSTLVAFWLPNIAQLLFVTCGGCKDTWRGFSMMAFKDLWPVFKLSMSSGGMLCLELWYNSILVLLTGNLK

NAEVALDALAICLNINGLEMMIALGFLAAASVRVSNELGSGNPKGAKFATLTAVFTSLSLGIVLFFVFLFLRGRVSYIFTTSEAVAAEVADLSPLLAFSI

LMNSVQPVLSGVAVGAGWQGYVTYVNLACYYLVGIPIGIILGYVVGLQVKGVWIGMLFGIFVQTCVLTVMTLRTDWDQQVSTSLRRLNRWVVPESRDVNQ

VSSEE

>AT5G17700.1

MSGGGGEMEERLLNGSETEQRRESLYLRKKIWSEVRKMWRIALPSTLFRVMSFGCVVVAQAFIGHSSETGLAAYALLQSTFIRFIYGIM

AGMSSATETLCGQAYGAEQYHMMGIYLQRSWIVDTFIATLFVPFIVLAGPILRLLGQNVVISETVDEIYPWVIPYLYSIVFTMTMQMYLQAQMKNAIIGI

LSTLALVLDIAATWWCVSVMGMGIHGALLGLNISSWSVAIAEFVYVFGGWCPHTWTGFSTAAFLDLIPMLKLSISSGFMLCLEYWYMSIIVLMSGYAKDA

NIAISAFSICQYIYSWEMNICFGLMGAACVRVANELGKGDADAVRFSIKVVLVVSAVIGVICSALCLAFGGQISYLFSDSQAVSDAVADLSIVLSISILF

NIIQPILSGVAIGAGMQSMVALVNLASYYAIGVPLGVLLVYVFNFGIKGLWSGMLAGVGIQTLILCYVIYKTDWELEVKKTNERMKTWTLNLPAVQSTTI

STRDEERK

>AT1G73700.1

MEDGVTPPLLITEKDTTMIRVKEEVKKQLWLSAPLIGVSLLQYSLQVISVMFVGHLGSLPLSAASIATSFASVTGFTFLLGTASALETL

CGQAYGAKLYGKLGIQMQRAMFVLLILSVPLSIIWANTEQILVLVHQDKSIASVAGSYAKYMIPSLFAYGLLQCINRFLQAQNNVFPVFVCSGITTCLHL

LLCWLFVLKTGLGYRGAALAISVSYWFNVILLSCYVKFSPSCSHSWTGFSKEAFQELYDFSKIAFPSAVMVCLELWSFELLVLASGLLPNPVLETSVLSI

CLNTSLTIWQISVGLGGAASIRVSNELGAGNPQVAKLAVYVIVGIAVAEGIVVVTVLLSIRKILGHAFSSDPKIIAYAASMIPIVACGNFLDGLQCVLSG

VARGCGWQKIGACVNLGSYYLVGVPLGLLLGFHFHIGGRGLWLGIVTALSVQVLCLSLVTIFTNWDKEAKKATNRVGSSDDKDGDVQ

>AT4G25640.2

MDPTAPLLTHGGEVEEDYAPARSWTDVKRVLSTESAKLWMIAAPVGFNIICQYGVSSVTNIFVGHIGEVELSAVSISLSVIGTFSFGFL

LGMGSALETLCGQAYGAGQVNMLGVYMQRSWIILFVSCFFLLPIYIFATPVLRLLGQAEEIAVPAGQFTLLTIPQLFSLAFNFPTSKFLQAQSKVVAIAW

IGFVALSLHVIMLWLFIIEFGWGTNGAALAFNITNWGTAIAQIVYVIGWCNEGWTGLSWLAFKEIWAFVRLSIASAVMLCLEIWYMMSIIVLTGRLDNAV

IAVDSLSICMNINGLEAMLFIGINAAISVRVSNELGLGRPRAAKYSVYVTVFQSLLIGLVFMVAIIIARDHFAIIFTSSKVLQRAVSKLAYLLGITMVLN

SVQPVVSGVAVGGGWQGLVAYINLGCYYIFGLPFGYLLGYIANFGVMGLWSGMIAGTALQTLLLLIVLYKTNWNKEVEETMERMKKWGGSETTSKDILAS

GWPSGLRRQTQVLVFVRGRGFKPHF

>AT1G51340.2

MMSEDGYNTDFPRNPLYIFFSDFRSVLKFDELGLEIARIALPAALALTADPIASLVDTAFIGQIGPVELAAVGVSIALFNQVSRIAIFP

LVSITTSFVAEEDACSSQQDTVRDHKECIEIGINNPTEETIELIPEKHKDSLSDEFKTSSSIFSISKPPAKKRNIPSASSALIIGGVLGLFQAVFLISAA

KPLLSFMGVKHDSPMMRPSQRYLSLRSLGAPAVLLSLAAQGVFRGFKDTTTPLFATVIGDVTNIILDPIFIFVFRLGVTGAATAHVISQYLMCGILLWKL

MGQVDIFNMSTKHLQFCRFMKNGFLLLMRVIAVTFCVTLSASLAAREGSTSMAAFQVCLQVWLATSLLADGYAVAGQAILASAFAKKDYKRAAATASRVL

QLGLVLGFVLAVILGAGLHFGARVFTKDDKVLHLISIGLPFVAGTQPINALAFVFDGVNFGASDFGYAAASLVMVAIVSILCLLFLSSTHGFIGLWFGLT

IYMSLRAAVGFWRIGTGTGPWSFLRS

>AT4G00350.1

MEIPVREERRSSSSSAGPLQQTISLAADDAIDSGPSSPLVVKVSVFETEHETTKLIHAPSTLLGETTGDADFPPIQSFRDAKLVCVVET

SKLWEIAAPIAFNILCNYGVNSFTSIFVGHIGDLELSAVAIALSVVSNFSFGFLLGMASALETLCGQAFGAGQMDMLGVYMQRSWLILLGTSVCLLPLYI

YATPLLILLGQEPEIAEISGKFTTQIIPQMFALAINFPTQKFLQSQSKVGIMAWIGFFALTLHIFILYLFINVFKWGLNGAAAAFDVSAWGIAIAQVVYV

VGWCKDGWKGLSWLAFQDVWPFLKLSFASAVMLCLEIWYFMTIIVLTGHLEDPVIAVGSLSICMNINGWEGMLFIGINAAISVRVSNELGSGHPRAAKYS

VIVTVIESLVIGVVCAIVILITRDDFAVIFTESEEMRKAVADLAYLLGITMILNSLQPVISGVAVGGGWQAPVAYINLFCYYAFGLPLGFLLGYKTSLGV

QGIWIGMICGTSLQTLILLYMIYITNWNKEVEQASERMKQWGAGYEKLEKIAT

>AT4G21903.2

MNGSNETVERRIELRRPLVDTEKKLPLEVGLESVLTESSLPYRRRVYLGMCIELKLLLRLALPAILVYLINGGMGISARIFAGHLGSTQ

LAAASIGNSSFSLVYALMLGMGSAVETLCGQAYGAHRYEMLGIYLQRATIVLALVGFPMTILYTFSYPILLLLGEPKTVSYMGSLYIAGLIPQIFAYAVY

FTAQKFLQAQSVVAPSAYISAAALVLQISLTWITVYAMGQGLMGIAYVLTISWWFIVGAQTFYVITSVRFKDTWTGFSWKSLHGLWSFFKLSAGSAVMIC

LELWYTQILVLLAGLLKDPALSLDSLSICMSISALSFMVSVGFNAAVSVRTSNELGAGNPKSALFSTWTATFVSFVISVVEALVVIASRDNVSYIFTSDA

DVAKAVSDLCPFLAVTIILNGIQPVLSGVAVGCGWQTYVAYVNIGCYYIVGIPIGCILGFTFNFQAKGIWTGMIGGTLMQTLILLYVTYQADWDKEVMLH

EIKLKKRESDWICGTTKSLSRISNFVMK

>AT2G04040.1

MEEPFLLRDELLVPSQVTWHTNPLTVELKRVSRLAAPMATVTIAQYLLPVISVMVAGHNGELQLSGVALANSFTNVTGFSIMCGLVGAL

ETLCGQAYGAKQYEKIGTYAYSAIASNIPICFLISILWLYIEKILISLGQDPEISRIAGSYAFWLIPALFGQAIVIPLSRFLLTQGLVIPLLFTAVTTLL

FHVLVCWTLVFLFGLGCNGPAMATSVSFWFYAVILSCYVRFSSSCEKTRGFVSRDFVSSIKQFFQYGIPSAAMICLEWWLFEILILCSGLLPNPKLETSV

LSICLTIETLHYVISAGVAAAVSTRVSNNLGAGNPQVARVSVLAGLCLWIVESAFFSILLFTCRNIIGYAFSNSKEVLDYVADLTPLLCLSFILDGFTAV

LNGVARGSGWQHIGAWNNTVSYYLVGAPVGIYLAFSRELNGKGLWCGVVVGSTVQATILAIVTASINWKEQAEKARKRIVSTENRLA

>AT2G04050.1

MEEPFLLQDEHLVPCKDTWKSGQVTVELKKVSSLAAPMAAVTIAQYLLPVISVMVAGHNGELQLSGVALATSFTNVSGFSILFGLAGAL

ETLCGQAYGAKQYEKIGTYTYSATASNIPICVLISVLWIYIEKLLISLGQDPDISRVAGSYALWLIPALFAHAFFIPLTRFLLAQGLVLPLLYCTLTTLL

FHIPVCWAFVYAFGLGSNGAAMAISVSFWFYVVILSCYVRYSSSCDKTRVFVSSDFVSCIKQFFHFGVPSAAMVCLEWWLFELLILCSGLLPNPKLETSV

LSICLTTASLHYVIPGGVAAAVSTRVSNKLGAGIPQVARVSVLAGLCLWLVESAFFSTLLFTCRNIIGYAFSNSKEVVDYVANLTPLLCLSFILDGFTAV

LNGVARGSGWQHIGALNNVVAYYLVGAPVGVYLAFNRELNGKGLWCGVVVGSAVQAIILAFVTASINWKEQAEKARKRMVSSENRLA

>AT1G33100.1

MAGRGGELTEALVKKTGREEEDELGMKEKVWIESKKLWVVAAPAIFTRYSTFGVSMVTQAFIGHLGPTELAAYSITFTILLRFSNGILL

GMAGALGTLCGQAYGAKQYQMLGIYLQRSWIVLTGGTICLMPVFIFAGPILLALGQEERIVRVARVLALWVIGINFSFVPSFTCQMFLQAQSKNKIISYV

TAVSLGLHVFFSWLLVAHFNFGITGAMTSMLIAFWLPIIVQLLYVTCGGCKDTWRGFSMLAFKDLWPVLKLSLSSGGMLCLELWYNSVLVLLTGNLKNAE

VALDALAICISINALEMMIALGFLAAVSVRVSNELGSGNPKGAKFATLIAVFTSLSIGIVLFFVFLFLRGRISYIFTTSEAVAAEVADLSPLLAFSILLN

SVQPVLSGVAIGAGWQGYVAYVNLACYYLVGIPIGVILGYVVGLQVKGVWIGMLFGIFVQTCVLTVMTLRTDWDQQVSTSLRNINRWVVPESRDANQISS

EE

>AT1G66780.1

MENGFSLVPKEEEEEEDYSNEKSEDQTSYYLSTEMMKKVSFMAAPMVAVAASQYLLQVISIVMAGHLDELSLSAVAIATSLTNVTGFSL

IFGLAGALETLCGQAFGAGQFRNISAYTYGSMLCLLLVCFPISLLWVFMDKLLELFHQDPLISQLACRYSIWLIPALFGYSVLQSMTRFFQSQGLVLPLF

LSSLGALFFHVPFSWLLVYKLRFGIVGAALSIGFSYWLNVGLLWAFMRDSALYRKNWNLRAQEIFLSMKQFITLAIPTAMMTCLEWWSFELLILMSGLLP

NSKLETSVLSICLTMSSLHYVIVNAIGAAASTHVSNKLGAGNPKAARSAANSAIFLGMIDAAIVSISLYSYRRNWAYIFSNESEVADYVTQITPFLCLSI

GVDSFLAVLSGVARGTGWQHIGAYANIGSYYLVGIPVGSILCFVVKLRGKGLWIGILVGSTLQTIVLALVTFFTNWEQEVAKARDRVIEMIPQEII

>AT4G21910.4

MEVPSETTNLADLRRPLVVPVVSERKPPADVGLGLESVLTERSLPYRRRVYLGACIEMKLLFRLALPAILVYLVNSGMGISARIFAGHL

GKNELAAASIGNSCFSLVYGLMLGMGSAVETLCGQAYGAHRYEMLGIYLQRATIVLALVGLPMTLLYTFSYPILILLGEPKTVSYMGSKYIAGLIPQIFA

YAVNFTAQKFLQAQSVVAPSAFISAAALILQILLTWITVYVMDMGFMGIAYVLTISWWVIVGSQCFYIAVSPKFRHTWTGLSWRSLQGLWSFFKLSAGSA

VMICLEMWYSQILVLLAGLLENPARSLDSLSICMSISALSFMVSVGFNAAVSVRTSNELGAGNPKSAWFSTWTATFVSFVISVTEALAVIWFRDYVSYIF

TEDADVAKAVSDLCPFLAITIILNGIQPVLSGVAVGCGWQTYVAYVNVGCYYVVGIPVGCILGFTFDFQAKGIWTGMIGGTLMQTLILLYVTYRTDWDKE

VMLHEIKWKKRGNVWICGTTRRSLSKTSYNKFGGVIDNKEKEISVVCAGDFNVRSFSWLLCLFYGNRTIKIYFCLMDYSLFTKSCS

>AT4G22790.1

MSETSKSESLDPEVSEGLCSKTLMQSIVHELKLQMRIGLPLVVMNLLWFGKMTTTSVFLGRQGELNLAGGSLGFSFANVTGFSVLYGIS

AAMEPICGQAFGAKNFKLLHKTLFMAVLLLLLISVPISFLWLNVHKILTGFGQREDISFIAKKYLLYLLPELPILSFLCPLKAYLSSQGVTLPIMFTTAA

ATSLHIPINIVLSKARGIEGVAMAVWITDFIVVILLTGYVIVVERMKENKWKQGGWLNQSAQDWLTLIKLSGPCCLTVCLEWWCYEILVLLTGRLPNPVQ

AVSILIIVFNFDYLLYAVMLSLGTCVATRVSNELGANNPKGAYRAAYTTLIVGIISGCIGALVMIAFRGFWGSLYTHHDQLILNGVKKMMLIMAVIEVVN

FPLMVCGEIVRGTAKPSLGMYANLSGFYLLALPLGATLAFKAKQGLQGFLIGLFVGISLCLSILLIFIARIDWEKEAGKAQILTCNTEDEQTSQGSGQDS

HS

>AT5G10420.1

MDKKSGGTKAIEEATVPLLECHNAAEEGGGMKREIWIETKKIWYIVGPSIFTGLATYSILIITQAFAGHLGDLELAAISIINNFTLGFN

YGLLLGMASALETLCGQAFGAREYYMLGVYMQRYWIILFLCCILLLPMYLFATPILKFIGQSDDIAELTGTIALWVIPVHFAFAFFFPLNRFLQCQLKNK

VIAISAGVSLAVHILVCWFFVYGYKLGIIGTMASVNVPWWLNIFILFLYSTRGGCTLTWTGFSSEAFTGLLELTKLSASSGIMLCLENWYYKILMLMTGN

LVNAKIAVDSLSICMSVNGWEMMIPLAFFAGTGVRVANELGAGNGKGARFATIVSITLSLMIGLFFTVIIVIFHDQIGSIFSSSEAVLNAVDNLSVLLAF

TVLLNSVQPVLSGVAVGSGWQSYVAYINLGCYYLIGLPFGLTMGWIFKFGVKGIWAGMIFGGTAIQTLILIIITTRCDWDNEAHKSSVRIKKWLVSDAGN

>AT1G71140.1

MDSAEKGLLVVSDREEVNKKDGFLRETKKLSYIAGPMIAVNSSMYVLQVISIMMVGHLGELFLSSTAIAVSFCSVTGFSVVFGLASALE

TLCGQANGAKQYEKLGVHTYTGIVSLFLVCIPLSLLWTYIGDILSLIGQDAMVAQEAGKFATWLIPALFGYATLQPLVRFFQAQSLILPLVMSSVSSLCI

HIVLCWSLVFKFGLGSLGAAIAIGVSYWLNVTVLGLYMTFSSSCSKSRATISMSLFEGMGEFFRFGIPSASMICLEWWSFEFLVLLSGILPNPKLEASVL

SVCLSTQSSLYQIPESLGAAASTRVANELGAGNPKQARMAVYTAMVITGVESIMVGAIVFGARNVFGYLFSSETEVVDYVKSMAPLLSLSVIFDALHAAL

SGVARGSGRQDIGAYVNLAAYYLFGIPTAILLAFGFKMRGRGLWIGITVGSCVQAVLLGLIVILTNWKKQARKARERVMGDEYEEKESEEEHEYIS

>AT1G64820.1

METDFSLVRKEEEEEEDNRNGMSYLSMEMMKKVSSMAAPMVAVSVSQFLLQVISMVMAGHLDELSLSAVAIATSLTNVTGFSLIVGFAG

ALDTLCGQAFGAEQFGKIGAYTYSSMLCLLVFCFSISIVWFFMDKLLEIFHQDPLISQLACRYSIWLIPALFGFTLLQPMTRYFQSQGITLPLFVSSLGA

LCFHIPFCWLLVYKLKFGIVGAALSIGFSYWLNVFLLWIFMRYSALHREMKNLGLQELISSMKQFIALAIPSAMMICLEWWSFEILLLMSGLLPNSKLET

SVISICLTTSAVHFVLVNAIGASASTHVSNELGAGNHRAARAAVNSAIFLGGVGALITTITLYSYRKSWGYVFSNEREVVRYATQITPILCLSIFVNSFL

AVLSGVARGSGWQRIGGYASLGSYYLVGIPLGWFLCFVMKLRGKGLWIGILIASTIQLIVFALVTFFTNWEQEATKARDRVFEMTPQVKGNQKTQIIVEE

DTQVLLNHIAETV

>AT2G34360.1

MREEREDMLSWPLIGEKEKRSRFVKEEVEKQLLLSGPLIAVSLLQFCLQIISVMFVGHLGSLPLSAASIATSFASVTGFTFLMGTASAM

DTVCGQSYGAKMYGMLGIQMQRAMLVLTLLSVPLSIVWANTEHFLVFFGQDKSIAHLSGSYARFMIPSIFAYGLLQCLNRFLQAQNNVIPVVICSGVTTS

LHVIICWVLVLKSGLGFRGAAVANAISYWLNVILLSCYVKFSPSCSLTWTGFSKEARRDIIPFMKLVIPSAFMVCSLEMWSFELLVLSSGLLPNPVLETS

CPRTVWMIPFGLSGAASTRVSNELGSGNPKGAKLAVRVVLSFSIVESILVGTVLILIRKIWGFAYSSDPEVVSHVASMLPILALGHSLDSFQTVLSGVAR

GCGWQKIGAFVNLGSYYLVGVPFGLLLGFHFHVGGRGLWLGIICALIVQGVCLSLITFFTNWDEEVKKATSRAKSSSEVKEFAVDNGSILV

>AT4G38380.1

MESSRVVVGGGLPLANRRNSSFAKPKIQQGTFLPLSRINNVSAPQKCSLHTNPNPMFPFVTRRKSQTNPDCGVVKLGEEDDSCSSLDKL

PEVNGVHTGVARPVDIKRELVMLSLPAIAGQAIDPLTLLMETAYIGRLGSVELGSAGVSMAIFNTISKLFNIPLLSVATSFVAEDIAKIAAQDLASEDSQ

SDIPSQGLPERKQLSSVSTALVLAIGIGIFEALALSLASGPFLRLMGIQSMSEMFIPARQFLVLRALGAPAYVVSLALQGIFRGFKDTKTPVYCLGIGNF

LAVFLFPLFIYKFRMGVAGAAISSVISQYTVAILMLILLNKRVILLPPKIGSLKFGDYLKSGGFVLGRTLSVLVTMTVATSMAARQGVFAMAAHQICMQV

WLAVSLLTDALASSGQALIASSASKRDFEGVKEVTTFVLKIGVVTGIALAIVLGMSFSSIAGLFSKDPEVLRIVRKGVLFVAATQPITALAFIFDGLHYG

MSDFPYAACSMMVVGGISSAFMLYAPAGLGLSGVWVGLSMFMGLRMVAGFSRLMWRKGPWWFMHTSDKRLA

>AT2G04066.1

MEASVHNYSRSPGFYVEKILKVSIKRIEKKKENRYVTAFFSTLLFTCRNIIGYTFSNSKEVVDYVADISPLLCLSFILDGLTAVLNGVA

RGCGWQHIGALINVVAYYLVGAPVGVYLAFSREWNGKGLWCGVMVGSAVQATLLAIVTASMNWKEQAEKARKRIISTKNGLV

>AT5G44050.1

MGERDDEAEGILEKAKIPLLKDQNVAEEENGEIKKEIWLETKKLWRIVGPAIFTRVTTNLIFVITQAFAGHLGELELAAISIVNNVIIG

FNYSLFIGMATALETLCGQAFGAKKYDMFGVYLQRSWIVLFLFSILLLPMYIFATPILKFMGQPDDIAELSGIISVWAIPTHFSFAFFFPINRFLQCQLK

NSVIAISSGVSLVVHIFVCWLFVYVLELGVIGTIATANVSWWLNVFILFTYTTCGGCPLTWTGFSMESFTRLWEFTKLSASSGIMVCLENWYYRMLIVMT

GNLEDARIDVDSMSICMSINGLEMMVPLAFFAGTSVRVANELGAGNGKRARFAMIISVTQSLIIGIIISVLIYFLLDQIGWMFSSSETVLKAVNNLSILL

SFAILLNSVQPVLSGVAVGSGWQSLVAFINLGCYYFIGLPLGIVMGWMFKFGVKGIWAGMIFGGTMVQTLILIFITMRCDWEKEAQNAKVRVNKWSVSDA

RK

>AT1G15180.1

MGDAESTSKTSLLLPVERVENVTWRDLRDGLFTAELKRLICFAAPMAAVVIAQFMLQIISMVMVGHLGNLSLASASLASSFCNVTGFSF

IVGLSCALDTLSGQAYGAKLYRKVGVQTYTAMFCLALVCLPLTLIWLNMETLLVFLGQDPSIAHEAGRYAACLIPGLFAYAVLQPLTRYFQNQSMITPLL

ITSCFVFCLHVPLCWLLVYKSGLGNLGGALALSFSNCLYTIILGSLMCFSSACSETRAPLSMEIFDGIGEFFRYALPSAAMICLEWWSYELIILLSGLLP

NPQLETSVLSVCLQTTATVYSIHLAIAAAASTRISNELGAGNSRAANIVVYAAMSLAVVEILILSTSLLVGRNVFGHVFSSDKETIDYVAKMAPLVSISL

ILDGLQGVLSGIARGCGWQHIGAYINLGAFYLWGIPIAASLAFWIHLKGVGLWIGIQAGAVLQTLLLTLVTGCTNWESQADKARNRMALAYGT

>AT1G71870.1

MEDKIQSDDFTSHKNPTLPQVIEELKELWAMVLPITAMNCLVYVRAVVSVLFLGRLGSLELAGGALSIGFTNITGYSVMVGLASGLEPV

CSQAYGSKNWDLLTLSLHRMVVILLMASLPISLLWINLGPIMLFMGQNPEITATAAEYCLYALPDLLTNTLLQPLRVYLRSQRVTKPMMWCTLAAVAFHV

PLNYWLVMVKHWGVPGVAIASVVTNLIMVVLLVGYVWVSGMLQKRVSGDGDGGSTTMVAVVAQSSSVMELVGGLGPLMRVAVPSCLGICLEWWWYEIVIV

MGGYLENPKLAVAATGILIQTTSLMYTVPMALAGCVSARVGNELGAGRPYKARLAANVALACAFVVGALNVAWTVILKERWAGLFTGYEPLKVLVASVMP

IVGLCELGNCPQTTGCGILRGTGRPAVGAHVNLGSFYFVGTPVAVGLAFWLKIGFSGLWFGLLSAQAACVVSILYAVLARTDWEGEAVKAMRLTSLEMRK

VGQDEESSLLLLDDEKLGDVL

>AT1G12950.1

MEKDNDFKDPFLASTEEEELDPATQKALMEYLGVGSRASSLVSFSSTAVDIPPISGVGDFVREFRIESRKLWKLAGPAIFTTMSQYSLG

AVTQVFAGHISTLALAAVSIENSVIAGFSFGIMLGMGSALETLCGQAFGAGKVSMLGVYLQRSWVILSVTALFLSLIYIFAAPILTFIGQTAAISAMAGI

FSIYMIPQIFAYAINFPTAKFLQSQSKIMVMAGISGVVLVIHSFFTWLVMSRLHWGLPGLALVLNTSWWVIVVAQLVYIFNCTCGEAWSGFTWEAFHNLW

GFVKLSLASAAMLCLEIWYFMALVLFAGYLKNAEVSVAALSICMNILGWAAMVAFGTNAAVSVRVSNELGASHPRTAKFSLVVAVILSTAIGMFIAAGLL

FFRNEYPVLFVEDEEVRNVVRELTPMLAFCIVINNVQPVLSGVAVGAGWQAVVAYVNIACYYLFGVPFGLLLGFKLEYGVMGIWWGMVTGTFVQSIVLTW

MICKTNWEKEASMAEERIKEWGGVPAEKETLLN

>AT1G23300.1

METLNVDHEDTISSEQEHRAHTKSDTDMPPISGGRDFIRQFAAESKKLWWLAGPAIFTSFCQYSLGAVTQILAGHVNTLALAAVSIQNS

VISGFSVGIMLGMGSALATLCGQAYGAGQLEMMGIYLQRSWIILNSCALLLCLFYVFATPLLSLLGQSPEISKAAGKFSLWMIPQLFAYAVNFATAKFLQ

AQSKVIAMAVIAATVLLQHTLLSWLLMLKLRWGMAGGAVVLNMSWWLIDVTQIVYICGGSSGRAWSGLSWMAFKNLRGFARLSLASAVMVCLEVWYFMAL

ILFAGYLKNPQVSVAALSICMNILGWPIMVAFGFNAAVSVRESNELGAEHPRRAKFLLIVAMITSVSIGIVISVTLIVLRDKYPAMFSDDEEVRVLVKQL

TPLLALTIVINNIQPVLSGVAVGAGWQGIVAYVNIGCYYLCGIPIGLVLGYKMELGVKGIWTGMLTGTVVQTSVLLFIIYRTNWKKEASLAEARIKKWGD

QSNKREEIDLCEEDENNSNGENNHRK

>AT5G65380.1

MRGGDGEEGSESRVALLKSPHTAEEDGEGLKDRILVETKKLWQIVGPAIFSRVTTYSMLVITQAFAGHLGDLELAAISIVNNVTVGFNF

GLLLGMASALETLCGQAFGAKKYHMLGVYMQRSWIVLFFCCVLLLPTYIFTTPVLKFLGQPDDIAELSGVVAIWVIPLHFAFTLSFPLQRFLQCQLKNRV

TAYAAAVALVVHILVCWLFVDGLKLGVVGTVATISISWWVNVLILLVYSTCGGCPLTWTGLSSEALTGLWEFLKLSASSGVMLCLENWYYRILIIMTGNL

QNARIAVDSLSICMAINGWEMMIPLAFFAGTGVRVANELGAGNGKGARFATIVSVTQSLIIGLFFWVLIMLLHNQIAWIFSSSVAVLDAVNKLSLLLAFT

VLLNSVQPVLSGVAVGSGWQSYVAYINLGCYYCIGVPLGFLMGWGFKLGVMGIWGGMIFGGTAVQTMILSFITMRCDWEKEAQKASARINKWSNTIK

>AT2G04070.1

MEEPFLPQDEQIVPCKATWKSGQLNVELKKVSRLAVPMATVTIAQYLLPVISVMVAGHNGELQLSGVALATSFTNVSGFSIMFGLVGSL

ETLSGQAYGAKQYEKMGTYTYSAISSNIPICVLISILWIYMEKLLISLGQDPDISRVAGSYALRLIPTLFAHAIVLPLTRFLLAQGLVLPLLYFALTTLL

FHIAVCWTLVSALGLGSNGAALAISVSFWFFAMTLSCYVRFSSSCEKTRRFVSQDFLSSVKQFFRYGVPSAAMLCLEWWLFELLILCSGLLQNPKLETSV

LSICLTTATLHYVIPVGVAAAVSTRVSNKLGAGIPQVARVSVLAGLCLWLVESSFFSILLFAFRNIIGYAFSNSKEVVDYVADLSPLLCLSFVLDGFTAV

LNGVARGCGWQHIGALNNVVAYYLVGAPVGIYLAFSCELNGKGLWCGVVVGSAVQAIILAIVTASMNWKEQAKKARKRLISSENGLA

>AT2G04100.1

MEDPLLLGDNQIITGSLKPTPTWRMNFTAELKNLSRMALPMATVTVAQYLLPVISVMVAGHRSELQLSGVALATSFTNVSGFSVMFGLA

GALETLCGQAYGAKQYAKIGTYTFSAIVSNVPIVVLISILWFYMDKLFVSLGQDPDISKVAGSYAVCLIPALLAQAVQQPLTRFLQTQGLVLPLLYCAIT

TLLFHIPVCLILVYAFGLGSNGAALAIGLSYWFNVLILALYVRFSSSCEKTRGFVSDDFVLSVKQFFQYGIPSAAMTTIEWSLFEFLILSSGLLPNPKLE

TSVLSICLTTSSLHYVIPMGIGAAGSIRVSNELGAGNPEVARLAVFAGIFLWFLEATICSTLLFICRDIFGYAFSNSKEVVDYVTELSPLLCISFLVDGF

SAVLGGVARGSGWQHIGAWANVVAYYLLGAPVGLFLGFWCHMNGKGLWIGVVVGSTAQGIILAIVTACMSWNEQAAKARQRIVVRTSSFGNGLA

>AT2G38330.1

MAAVATSFCFSPHRSPSRFGNPNSSIRRTIVCKSSPRDESPAVSTSSQRPEKQQNPLTSQNKPDHDHKPDPGIGKIGMEIMSIALPAAL

ALAADPITSLVDTAFVGHIGSAELAAVGVSVSVFNLVSKLFNVPLLNVTTSFVAEEQAIAAKDDNDSIETSKKVLPSVSTSLVLAAGVGIAEAIALSLGS

DFLMDVMAIPFDSPMRIPAEQFLRLRAYGAPPIVVALAAQGAFRGFKDTTTPLYAVVAGNVLNAVLDPILIFVLGFGISGAAAATVISEYLIAFILLWKL

NENVVLLSPQIKVGRANQYLKSGGLLIGRTVALLVPFTLATSLAAQNGPTQMAGHQIVLEIWLAVSLLTDALAIAAQSLLATTYSQGEYKQAREVLFGVL

QVGLATGTGLAAVLFITFEPFSSLFTTDSEVLKIALSGTLFVAGSQPVNALAFVLDGLYYGVSDFGFAAYSMVIVGFISSLFMLVAAPTFGLAGIWTGLF

LFMALRLVAGAWRLGTRTGPWKMLWSAPEKPE

>AT5G52450.1

MRDDRERGEGDLSWPLIGEKSSVKEEVKKQLWLSGPLIAVSLLQFCLQVISVMFVGHLGSLPLSAASIATSFASVTGFSFLMGTASALD

TLCGQAYGAKKYGMLGIQMQRAMFVLTLASIPLSIIWANTEHLLVFFGQNKSIATLAGSYAKFMIPSIFAYGLLQCFNRFLQAQNNVFPVVFCSGVTTSL

HVLLCWVLVFKSGLGFQGAALANSISYWLNVVLLFCYVKFSPSCSLTWTGFSKEALRDILPFLRLAVPSALMVCLEMWSFELLVLLSGLLPNPVLETSVL

SICLNTSGTMWMIPFGLSGAASTRISNELGAGNPKVAKLAVRVVICIAVAESIVIGSVLILIRNIWGLAYSSELEVVSYVASMMPILALGNFLDSLQCVL

SGVARGCGWQKIGAIINLGSYYLVGVPSGLLLAFHFHVGGRGLWLGIICALVVQVFGLGLVTIFTNWDEEAKKATNRIESSSSVKDFAVDDRSVVVF

>AT1G47530.1

MGKDKTLPLLDPREPPELTGTKSASKVWAKEFGEESKRLWELAGPAIFTAISQYSLGALTQTFSGRLGELELAAVSVENSVISGLAFGV

MLGMGSALETLCGQAYGAGQIRMMGIYMQRSWVILFTTALFLLPVYIWAPPILSFFGEAPHISKAAGKFALWMIPQLFAYAANFPIQKFLQSQRKVLVMA

WISGVVLVIHAVFSWLFILYFKWGLVGAAITLNTSWWLIVIGQLLYILITKSDGAWTGFSMLAFRDLYGFVKLSLASALMLCLEFWYLMVLVVVTGLLPN

PLIPVDAISICMNIEGWTAMISIGFNAAISVRVSNELGAGNAALAKFSVIVVSITSTLIGIVCMIVVLATKDSFPYLFTSSEAVAAETTRIAVLLGFTVL

LNSLQPVLSGVAVGAGWQALVAYVNIACYYIIGLPAGLVLGFTLDLGVQGIWGGMVAGICLQTLILIGIIYFTNWNKEAEQAESRVQRWGGTAQE

>AT3G21690.1

MDSSPNDGVHQPLLHPQPSPSPPESTNGELETVLSDVETPLFLRLRKATIIESKLLFNLAAPAVIVYMINYLMSMSTQIFSGHLGNLEL

AAASLGNTGIQVFAYGLMLGMGSAVETLCGQAYGGRKYEMLGVYLQRSTVLLTLTGLLLTLIYVFSEPILLFLGESPAIASAASLFVYGLIPQIFAYAAN

FPIQKFLQSQSIVAPSAYISTATLFVHLLLSWLAVYKLGMGLLGASLVLSLSWWIIVVAQFVYIVTSERCRETWRGFSVQAFSGLWSFFKLSAASAVMLC

LETWYFQILVLLAGLLENPELALDSLSICMTISGWVFMISVGFNAAISVRVSNELGAGNPKSAAFSVIIVNIYSLITCVILAIVILACRDVLSYAFTEGK

EVSDAVSDLCPLLAVTLVLNGIQPVLSGVAVGCGWQTFVAKVNVGCYYIIGIPLGALFGFYFNFGAKGIWTGMIGGTVIQTFILAWVTFRTDWTKEVEEA

SKRLDKWSNKKQEVVPE

>AT3G23550.1

MADPTSKDDHDGEGGRDKSSTFVQKLIDVEEAKTQIIYSLPMIFTNLFYYCIPLTSVMFASQLGQLELAGATLANSWATVTGFAFMTGL

SGALETLCGQGFGAKSYRMLGIHLQSSCIVSLVFTILITILWFFTESVFLLLRQDPSISKQAALYMKYLAPGLLAYGFLQNILRFCQTQCIVTPLVLFSF

LPLVINIGTTYALVHLAGLGFIGAPIATSISLWIAFVSLGFYVICSDKFKETWTGFSMESFHHVVLNLTLSIPSAAMVCLEYWAFEILVFLAGLMRNPEI

TTSLVAICVNTESISYMLTCGLSAATSTRVSNELGAGNVKGAKKATSVSVKLSLVLALGVVIAILVGHDAWVGLFSNSHVIKEGFASLRFFLAASITLDS

IQGVLSGVARGCGWQRLATVINLGTFYLIGMPISVLCGFKLKLHAKGLWIGLICGMFCQSASLLLMTIFRKWTKLTAATV

>AT5G52050.1

MSQSNRVRDEVTLPLLQKTSHLKNHSSVLSVFLNEAISICKISYPLVLTGLFLYVRSFVSLSFLGGLGDATLAGGSLAAAFANITGYSL

FSGLTMGVESICSQAFGARRYNYVCASVKRGIILLLVTSLPVTLLWMNMEKILLILKQDKKLASEAHIFLLYSVPDLVAQSFLHPLRVYLRTQSKTLPLS

ICTVIASFLHLPITFFLVSYLGLGIKGIALSGVVSNFNLVAFLFLYICFFEDKLSVNEDEKITEETCEDSVREWKKLLCLAIPSCISVCLEWWCYEIMIL

LCGFLLDPKASVASMGILIQITSLVYIFPHSLSLGVSTRVGNELGSNQPKRARRAAIVGLGLSIALGFTAFAFTVSVRNTWAMFFTDDKEIMKLTAMALP

IVGLCELGNCPQTTGCGVLRGSARPKIGANINGVAFYAVGIPVGAVLAFWFGFGFKGLWLGMLAAQITCVIGMMAATCRTDWELEAERAKVLTTAVDCGS

SDDDAKEDMEAGMVDK

>AT1G11670.1

MGSEATTAVNNLQQPLLESTKSEADFRMESVLTDTHLSYFRRIYLASLIEMKYLFHLAAPAIFVYVINNGMSMLTRIFAGRLGSMQLAA

ASLGNSGFNMFTLGLMLGMGSAVETLCGQAHGAHRYDMLGVYLQRSTIVLVITGLPMTLLFIFSKPLLISLGEPADVASVASVFVYGMIPMIFAYAVNFP

IQKFLQSQSIVTPSAYISAATLVIHLILSWLSVFKFGWGLLGLSVVHSLSWWIIVLAQIIYIKISPRCRRTWDGFSWKAFDGLWDFFQLSAASAVMLCLE

SWYSQILVLLAGLLKDPELALDSLAICMSISAMSFMVSVGFNAAASVRVSNELGAGNPRSAAFSTAVTTGVSFLLSLFEAIVILSWRHVISYIFTDSPAV

AEAVAELSPFLAITIVLNGVQPVLSGVAVGCGWQAYVAYVNIGCYYIVGIPIGYVLGFTYDMGARGIWTGMIGGTLMQTIILVIVTFRTDWDKEVEKASR

RLDQWEDTSPLLKQ

>AT2G21340.1

MQIQCKTLTFTVSSIPCNPKLPFPSSLTLRSWNPSFPSFRSSAVSGPKSSLKLNRFLRNCASTNQELVVDGETGNGSISELQGDAANGS

ISPVEVEAEVEEVKVDDLATQSIWGQMKEIVMFTGPAAGLWLCGPLMSLIDTAVIGQGSSLELAALGPATVICDYLCYTFMFLSVATSNLVATSLARQDK

DEVQHQISILLFIGLACGVTMMVLTRLFGSWALTAFTGVKNADIVPAANKYVQIRGLAWPAVLIGWVAQSASLGMKDSWGPLKALAVASAINGVGDVVLC

TFLGYGIAGAAWATMVSQVVAAYMMMDALNKKGYSAFSFCVPSPSELLTIFGLAAPVFITMMSKVLFYTLLVYFATSMGTNIIAAHQVMLQIYTMSTVWG

EPLSQTAQSFMPELLFGINRNLPKARVLLKSLVIIGATLGIVVGTIGTAVPWLFPGIFTRDKVVTSEMHKVIIPYFLALSITPSTHSLEGTLLAGRDLRY

ISLSMTGCLAVAGLLLMLLSNGGFGLRGCWYALVGFQWARFSLSLFRLLSRDGVLYSEDTSRYAEKVKAA

>AT3G03620.1

MSTQEEMEERLLREGSDAEGQSNNRESIYLRTKVWSEVNKMWRIALPSSLFRMTSFGSIIVAQAFIGHSSELGLAAYALLQSTFIRFLY

GLMGGMSSATETLCGQAYGAEQYHTMGIYLQRSWIVDMAVTTLFLPFIVLAGPILRLLGQNVEITKTVDEIYPWMIPYVYSLIFTMTIQMYLQAQMRNAI

VGVLSTLSLALDLVVTWWCVSVMGMGIGGALLGLNVGSWAMVLAEFVYIFGGWCPFTWTGFSIAAFVDLIPMLKLSISSGFMICLEYWYMSILVLMAGYT

KDAKIAISAFSICQYIYTWELNICLGFLGAACVRVANELGKGDAHAVRFSIKVILTISTLMGVIFSALCLAFCGRISYLFSNSDEVSDAVNDLSVILAVS

ILLNSIQPILSGVAVGAGMQSIVAVVNLASYYAIGIPLGLILTYVFHLGVKGLWSGMLAGIAIQTIILCYIIYKTDWELEVKRTCERMKVWSLKPSNEES

NPIIREESRSK

>AT1G15150.1

MQDAERTTNDPVDRIEKVTWRDLQDGSFTAELKRLICFAAPMAAVVIIQFMIQIISMVMVGHLGRLSLASASFAVSFCNVTGFSFIIGL

SCALDTLSGQAYGAKLYRKLGVQAYTAMFCLTLVCLPLSLLWFNMGKLIVILGQDPAIAHEAGRYAAWLIPGLFAYAVLQPLIRYFKNQSLITPLLVTSS

VVFCIHVPLCWLLVYKSGLGHIGGALALSLSYWLYAIFLGSFMYYSSACSETRAPLTMEIFEGVREFIKYALPSAAMLCLEWWSYELIILLSGLLPNPQL

ETSVLSICFETLSITYSIPLAIAAAASTRISNELGAGNSRAAHIVVYAAMSLAVMDALMVSMSLLAGRHVFGHVFSSDKKTIEYVAKMAPLVSISIILDS

LQGVLSGVASGCGWQHIGAYINFGAFYLWGIPIAASLAFWVHLKGVGLWIGILAGAVLQTLLLALVTGCTNWKTQAREARERMAVAHESELTESELPI

>AT3G26590.1

MAKDKDITETLLTAAEERSDLPFLSVDDIPPITTVGGFVREFNVETKKLWYLAGPAIFTSVNQYSLGAITQVFAGHISTIALAAVSVEN

SVVAGFSFGIMLGMGSALETLCGQAFGAGKLSMLGVYLQRSWVILNVTALILSLLYIFAAPILASIGQTAAISSAAGIFSIYMIPQIFAYAINFPTAKFL

QSQSKIMVMAVISAVALVIHVPLTWFVIVKLQWGMPGLAVVLNASWCFIDMAQLVYIFSGTCGEAWSGFSWEAFHNLWSFVRLSLASAVMLCLEVWYFMA

IILFAGYLKNAEISVAALSICMNILGWTAMIAIGMNTAVSVRVSNELGANHPRTAKFSLLVAVITSTLIGFIVSMILLIFRDQYPSLFVKDEKVIILVKE

LTPILALSIVINNVQPVLSGVAVGAGWQAVVAYVNIACYYVFGIPFGLLLGYKLNYGVMGIWCGMLTGTVVQTIVLTWMICKTNWDTEASMAEDRIREWG

GEVSEIKQLIN

>AT2G04080.1

MEEPFLPRDEQLVSCKSTWQSGQVTVELKKVSRLAAPMATVTIAQYLLPVISVMVAGHIGELELAGVALATSFTNVSGFSIMFGLVGAL

ETLCGQAYGAEQYEKIGTYTYSAMASNIPICFIISILWIYIEKLLITLGQEPDISRVAGSYSLWLVPALFAHAIFLPLTRFLLAQGLVISLLYSAMTTLL

FHIAVCWTLVFALGLGSNGAAIAISLSFWFYAVILSCHVRFFSSCEKTRGFVSNDFMSSIKQYFQYGVPSAGLICLEWWLFELLILCSGLLPNPKLETSV

LSICLTIGTLHYVIPSGVAAAVSTRVSNKLGAGNPQVARVSVLAGLCLWLVESAFFSTLLFTCRNIIGYTFSNSKEVVDYVADISPLLCLSFILDGLTAV

LNGVARGCGWQHIGALINVVAYYLVGAPVGVYLAFSREWNGKGLWCGVMVGSAVQATLLAIVTASMNWKEQAEKARKRIISTENGLV

>AT5G19700.1

METPNIISHTNLLSKIDLEKQNPAPIFPTITELKSEARSLFSLAFPTILAALILYARSAISMLFLGHIGELELAGGSLAIAFANITGYS

VLAGLALGMDPLCSQAFGAGRPKLLSLTLQRTVLFLLTSSVVIVALWLNLGKIMIYLHQDPSISSLAQTYILCSIPDLLTNSFLHPLRIYLRAQGITSPL

TLATLAGTIFHIPMNFFLVSYLGWGFMGVSMAAAASNLLVVIFLVAHVWIAGLHQPTWTRPSSECFKDWGPVVTLAIPSCIGVCLEWWWYEIMTVLCGLL

IDPSTPVASMGILIQTTSLLYIFPSSLGLAVSTRVGNELGSNRPNKARLSAIVAVSFAGVMGLTASAFAWGVSDVWGWIFTNDVAIIKLTAAALPILGLC

ELGNCPQTVGCGVVRGTARPSMAANINLGAFYLVGTPVAVGLTFWAAYGFCGLWVGLLAAQICCAAMMLYVVATTDWEKEAIRARKLTCTEGVDVVITTT

QTNGDLSEPLIYVVTVATD

>AT1G58340.1

MCNSKPSSASSSLLSCKDKTHISKLETCDTDNPHYSEFRDTDSLDLKRWPSFLEGLEEVKAIGKISGPTAMTGLLMYSRAMISMLFLGY

LGELELAGGSLSIGFANITGYSVISGLSMGMEPICGQAYGAKQMKLLGLTLQRTVLLLLSCSVPISFSWLNMRRILLWCGQDEEISSVAQQFLLFAIPDL

FLLSLLHPLRIYLRTQNITLPVTYSTAVSVLLHVPLNYLLVVKLEMGVAGVAIAMVLTNLNLVVLLSSFVYFTSVHSDTWVPITIDSLKGWSALLSLAIP

TCVSVCLEWWWYEFMIILCGLLANPRATVASMGILIQTTALVYVFPSSLSLGVSTRISNELGAKRPAKARVSMIISLFCAIALGLMAMVFAVLVRHHWGR

LFTTDAEILQLTSIALPIVGLCELGNCPQTTGCGVLRGCARPTLGANINLGSFYFVGMPVAILFGFVFKQGFPGLWFGLLAAQATCASLMLCALLRTDWK

VQAERAEELTSQTPGKSPPLLPIASSKSRSTSGTEDMMRTMLV

>AT1G66760.2

MKKSIETPLLLNTKQSQDEDKEKIRWEKMKKVASMAAPMVAVNMSQYLLQATSTMIVGHRSELALAGIALGSSFANVTGFGVLFGLSGS

LETLCGQAYGAKQYHKLGSYTFTSIVFLLIISVPISILWMFMNQILLLLHQDPQIAELAGVYCLWLVPALFGYSVLESLVRYFQSQSLIYPMVLSSLAAL

SFHVPLCWLMVHKFDFGAKGAAASIGISYWLNAVFLWVYMKRSSRCVETRIYMSKDVFVHTNIFFQFAIPSAMMCCLEWLAFEVITLLSGLLPNSKLETS

VISICLTTSSLHYNLVNGIGDAASTNVANELGAGNPRGARDSAAAAIIIAAVESVIVSSSLFLSRSVWPYAYSNVEEVISYVTDITPILCISILMDSFLT

VLSGIVRGTGWQKIGAYVNITSYYVIGIPVGLLLCFHLHFNGKGLWAGLVTGSTLQTLILFLVIGFTNWSKEAIKARERIGDEKVWRHDSLLN

>AT1G33090.1

MAGEGGELTAALLKKTTENGGEENDELGLKEKVWIESKKLWVVAAPSIFTKFSTYGVSLVTQGFVGHIGPTELAAYSITFTVLLRFSNG

ILLGMASALGTLCGQAYGAKQYHMLGIHLQRSWIVLTGCTICIMPIFIFSGPILLALGQEDHIVRVARVIALWLIAINFTFVPAFTCQIFLQSQSKNKII

AYVSAVTLGLHVFFSWLLVVHFNFGITGAMTSTLVAFWMPNIVQLLYVTSGGCKDTWRGFTMLAFKDLWPVFKLSLSSGGMVCLELWYNSILVLLTGNLK

NAEVAIDALAICINVNALQMMIALGFLAAVSVRVSNELGRGNPEGAKFATIVAVFTSLSIGLVLFFVFLFLRGRISYIFTTSEAVAAEVADLSPLLAFSI

LLNSVQPVLSGVAVGAGWQGYVAYINLACYYLLGIPVGLVLGYVVGLQVKGVWIGMLFGIFVQTCVLTIMTLRTDWDQQVSTSLKNINRWVVPESRDANQ

ISSEE

>AT1G61890.1

MNSESLENLHRPLIESSKSFVDYRLETVLTDRELPYFRRIYLAMMIEMKFLFHLAAPAIFVYVINNGMSILTRIFAGHVGSFELAAASL

GNSGFNMFTYGLLLGMGSAVETLCGQAHGAHRYEMLGVYLQRSTVVLILTCLPMSFLFLFSNPILTALGEPEQVATLASVFVYGMIPVIFAYAVNFPIQK

FLQSQSIVTPSAYISAATLVIHLILSWIAVYRLGYGLLALSLIHSFSWWIIVVAQIVYIKMSPRCRRTWEGFSWKAFEGLWDFFRLSAASAVMLCLESWY

SQILVLLAGLLKNPELALDSLAICMSISAISFMVSVGFNAAASVRVSNELGAGNPRAAAFSTVVTTGVSFLLSVFEAIVVLSWRHVISYAFTDSPAVAEA

VADLSPFLAITIVLNGIQPVLSGVAVGCGWQAFVAYVNIGCYYVVGIPVGFVLGFTYDMGAKGIWTGMIGGTLMQTIILVIVTLRTDWDKEVEKASSRLD

QWEESREPLLKQ

>AT1G15160.1

MEDAESTTKDPVDRVEKVTWRDLQDGSFTAELKKLICFAAPMAAVVITQSMLQIITMVIVGHLGRLSLASASFAISFCNVTGFSFIMGL

SCALDTLSGQAYGAKLYRKLGVQAYTAMFCLTLVCLPLSLLWFNMGKLLVILGQDPSIAHEAGRFAAWLIPGLFAYAVLQPLTRYFKNQSLITPLLITSC

VVFCLHVPLCWLLVYKSGLDHIGGALALSLSYWLYAIFLGSFMYFSSACSETRAPLTMEIFEGVREFIKYALPSAAMLCLEWWSYELIILLSGLLPNPQL

ETSVLSVCLQTLSMTYSIPLAIAAAASTRISNELGAGNSRAAHIVVYAAMSLAVVDALMVGTSLLAGKNLLGQVFSSDKNTIDYVAKMAPLVSISLILDS

LQGVLSGVASGCGWQHIGAYINFGAFYLWGIPIAASLAFWVHLKGVGLWIGIIAGAVLQTLLLALVTGCINWENQAREARKRMAVAHESELTESELPF

**The protein sequences of 52 rice MATEs**

>Os01g31980

MGSSDSQAPLLLPRGSHRKEEEEEEYAAAGKVRGCCGGDGEGGWWRE

ATAEAGRLASLAAPMIAVALLQLMMQLISTVMVGHLGEVALAGAAIAN

SLTNVSGFSVLMGLACGLETICGQAYGAEQYHKLALYMYRSIIVLLVVS

VPIAIIWVFIPEVLPLIGQQPEIASEVGKYALWLIPGLFAFTVAQCLSKFLQ

TQSLIFPMVLSSSITLALFIPLCWFMVYKVGMGNAGAALSVSICDWVEVT

VLGLYIVLSPSCEKTRAPLTWEAFSGIGSFLRLAVPSALMICLEWWSYEL

LVLLSGILPNPALETSVLSICISTVVLVYNLPHGIGTAASVRVSNELGAGN

PEGALVVGVALSVILCSAVLVSVTLLALRHFIGIAFSNEEEVINYVTRMVP

VLSISVITDSLQGVLSGVSRGCGWQLGAYVNLGAFYLVGVPVALFFGFA

MHLGGMGFWMGMVAGGATQVTLLSIITAMTNWRKMAEKARDRVFEE

RIPTQSV

>Os01g49120

MAAAAREEQPLLLRREEGEEEGEEVGWRRRWGSEAGKLAYLALPMVA

VSLTNYAVQVFSNMMVGHLPGVLPLSSAAIATSLASVTGFSLLIGMASA

LETLCGQAYGAKQYHTLGVHTYRAILTLLVVCIPLSLLWVFMGKILVLIG

QDPLISHGAGRYIVWLIPGLFANALIQPITKFLQSQSLIMPMLVASVATLV

FHIPLCWLMVFKTGLGYTGAALSISISYWLNVAMLVAYILLSSSCKETRT

PPTIEAFKGLDGFLRLALPSALMICLEWWSFELLILMSGLLPNPELQTSVL

SICLTSITLLFTIPYGLGAGGSTRVANELGAGNPEGARSAVYVVLSVAVT

EALIVCGTLLASRRLLGRAYSSEEEVISFVAMMVPLVCITVVTDGLQGV

MSGIARGCGWQHLGAYVNLGSFYLLGIPMAILLGFVLHMGAKGLWMGI

VCGSISQITLLSAITFFTNWQKMAENARERVFSEKPTEPSRYHLVE

>Os01g56050

MAPPAGTEAAGAGHRKNWRGESGNLWRIAGPVILTEIFQFLIGFVTAAF

VGHIGKVELAAVSVVNGVVEGLAFGLLVKNHARTLGMGSALETLCGQA

VGAGQPRMLGVYLQRSWVICLATSLALLPLYLLASPALRLLRQSAAISSV

AGRYARWCAPQLFAYAVNFPMQKFYQAQSRVWAVTAISAAALAAHAL

LNWLVVARLGHGVVGAALVGDVSWWLLNAAQFAYLVGGSFPEAWSG

FSRKAFTSLGGFVKLSLSSAVMLCLEMWYYTAVLILVGCLKNPEIQVGAI

SICMNYQLWTLMVAVGFNAAVSVRVANELGANHPKAAKFSVIVAVVTS

AAVGLVFTLVALVARKQLPRLFTDDDVLVRETAKLGYLLAATIFLNSIQP

VLSGVAIGAGWQSSVAFVNIGCYYLVGLPIAAVFGFRLSLNATGIWVGM

LIGTILQTVILLVILYRTKWQKEAMLAEERIKVWGGGVELPTIQEAS

>Os01g69010

MARSSSAPESMEADHQLTATVTAAASGDMPVTEQQQQKQMVAVAAPP

ATENADAAGGGGGDNGDHLPPTATSLLGGARRTGLHLFVLNARSALRL

DELGAEVLRIAVPASLALTADPLASLIDTAFIGRIGSVEIAAVGVAIAVFN

QVMKVCIYPLVSVTTSFVAEEDAILSKGAAGADDDNDDGHDAKGHGAS

AAAVADPEKQQVVGVDSAETNGAEVSTAAVRTTDDKKAAAAGVGVG

KCRRRFVPSVTSALIVGAFLGLLQAVFLVAAGKPLLRIMGVKPGSPMMIP

ALRYLVVRSLGAPAVLLSLAMQGVFRGFKDTKTPLYATVTGDLANIAL

DPILIFTCRFGVVGAAIAHVISQYLITLIMLCKLVRKVDVIPSSLKSLKFRR

FLGCGFLLLARVVAVTFCVTLAASLAARHGATAMAAFQICAQVWLASS

LLADGLAVAGQALLASAFAKKDHYKVAVTTARVLQLAVVLGVGLTAF

LAAGMWFGAGVFTSDAAVISTIHRGVPVGVAAVTIPCLVLLSSHGGFVG

IWIALAIYMSVRAFASTWRMGAARGPWKFLRK

>Os02g02980

MSAHLRLLSAAPLPALLPTRRLPAVPTPALAARAARLVLSRPLTEPQPPR

LPRGAAARCRGVAVAGADGDEPAAAAAGTAGLWEQVRDIVVFAGPAL

GLWICGPLMSLIDTMVIGQTSSLQLAALGPGTVFCDYLCYIFMFLSIATSN

MVATSLAKKDEELAQHQVSMLLFVALTCGLGMFLFTKLFGTQVLTVFT

GSGNYDIISAANTYAQIRGFAWPAVLVGLVAQSASLGMKDSWGPLKAL

AAASVINGVGDLLLCSVCGYGIAGAAWATMVSQIVAAFMMMQNLNKR

GFRAFSFTIPSSSELLQIFEIAAPVFITMTSKVAFYALLTYSATSMGAITLA

AHQVMVNVLCMCTVWGEPLSQTAQSFMPELIYGAKCNLMKARMLLKS

LVMIGAITGTTVGAVGTLVPWLFPSLFTNDFMVVQQMHKVLIPYFCALL

VTPSVHSLEGTLLAGRDLRFLSQSMGACFGIGTFLLMIIRNKFGSLPGCW

WILVLFQWGRFGSALQRLLSPTGMLYNENFNNHHDEYVKVKAT

>Os02g45380

MSSPRRDGRGAVDDLTASLLHKGDGGEAVFVVVVVPPVAEEEEPPPVLT

CKPPGRFARAVKEAWSVPFPMMPSMSAGAAGAEARSILGLALPMILTGL

LLYLRSMISMLFLGRLGGLALAGGSLAIGFANITGYSVLSGLAMGMEPIC

GQAFGAGHYDLLGVTMQRTVLLLVAASVPIAGLWVHMRPLLLLCGQD

AAIAAVAETYILASLPDLLLQAFLHPVRIYLRTQSINLPLTVCAALAIALH

LPINYVAVSVLGLGIKGVALASVLANLNLVLFLFGYIWFKGVHKRTGGF

ALSADCLRGWGELVSLALPSCISVCLEWWWYEIMILLCGLLANPQATVA

SMGILIQTTSLIYIFPSSLGFGVSTRVSNELGANRPERACRAATVGLMLGF

AFGGVASAFACHVRGAWATMFTADPAIVALTASVLPILGACELGNCPQT

TGCGVLRGSARPKDAASINLRSFYLVGTPVALILAFWYHYDFRGLWLGL

LAAQATCVVRMLLVIGETDWTAEAKRAQQLTGAADIKDCGGKGDHVA

VIEQPDEQC

>Os02g57570

MAYTEPLLLSARHTTQTSPRHHLLRSRHAAAAAADGRMVVAVQDDET

GALVAAVGKGDEDDDDDDAVAGEEDEDEDDAPVVRTARGAWEVFAA

ESRRLWAIGAPIAFNVICLYGTNSTTQIFVGHIGNRELSAVAIGLSVVSNF

SFGFLLGMGSALETLCGQAFGAGQVAMLGIYMQRSWIILAASAALLSPL

YVFAGPILRLLGQEESIAAAAGEFTVRIIPQMFALAINFPTQKFLQAQSKV

TVLAWIGFAALLAHVGLLALFVSALGWGIAGAAAAYDVSSWLTALAQV

AYVVGWCRDGWTGLSRKAFNELWAFVKLSLASAVMLCLEIWYMMVL

VVLTGHLDDAEIAVDSISIWYSLLP

>Os03g08900

MGDGRGDEEECRVALLNGGGAAKEGWQVVSGGDGKLRRRVWEESRK

LWVIVAPAIFSRVVTYSMNVITQAFAGHLGDLELAAISIANTVVVGFNFG

LMLGMASALETLCGQAFGAKKYHMMGVYMQRSWIVLLACAVLLLPM

YIYAEDVLLLTGQPPELSAMAGRVSVWFIPLHLSFAFLFPLQRFLQCQMK

NFASAAASGVALCVHVAISWLLVSRFRFGLVGIALTLNFSWWATAAML

FAYVACGGCPETWNGLSLEAFAGCHAMLLTEFVCLICSLENWYYRILIL

LTGNLKNAAIAVDALSICMTINAWELMIPLAFFAGTGVRVANELGAGNG

KGARFATIVSSVTSLVIGLFFWVLIVGLHDKFALIFTSSDVVLDAVDNLSV

LLAFTILLNSIQPVLSGVAVGSGWQSMVAYVNIGTYYLIGIPMGILLGWL

FKLGVLGIWAGMIGGTAVQTLILAIITIRCDWDKEAMIASTRMDKWSQV

R

>Os03g11734

MAGLKKMEEVTAAAAAVAASSTAEKRAAAVVVPDAALTMNGAAGAE

EKTAAAAAAPEDLPAPAALSGWPRRVGLYLFVMNIRSVFKLDELGSEVL

RIAVPASLALAADPLASLVDTAFIGRLGSVEIAAVGVSIAIFNQVSKVCIY

PLVSVTTSFVAEEDAIISKCIEENSSQDLEKASPVDSETNNLPVSGPDKVE

CVNSCIPTECTNPSDQGCKRKYIPSVTSAVIVGSFLGLLQAVFLVFSAKFV

LNIMGVKNDSPMLRPAVRYLTIRSLGAPAVLLSLAMQGVFRGFKDTKTP

LYATVVGDAANIILDPILMFVCHMGVTGAAVAHVISQYLITMILLCRLIR

QVDVIPPSLKSLKFGRFLGCGFLLLARVVAVTFCVTLASSLAARHGPTIM

AAFQICCQLWLATSLLADGLAVAGQAVLASAFAKNDKGKVVVATSRVL

QLSIVLGMGLTVVLGVGMKFGAGIFTKDIDVIDVIHKGIPFVAGTQTINSL

AFVFDGINFGASDYTYSAYSMVGVAAISIPCLVYLSAHNGFIGIWIALTIY

MSLRTIASTWRMGAARGPWVFLRK

>Os03g12790

MCNSGTSSSPSAPAPPPPPLTSFKHSSHLLRLVDDDADDGHALLLSKVAG

EAQAIGRVSVPMAVTGLVMYSRALISMLFLGRLGELALAGGSLALGFAN

ITGYSVLSGLALGMEPICGQAFGARRGKLLALALHRTVLLLLAVALPISL

LWVTSTGYILKQLGQDEGVADAAQTFAAYASADLAVLAVLHPLRVYLR

SQNLTLPITACSLFSVLLHGPINYLLVVRLRMGVAGVALAVALTDLNLLL

ALLCFLAISGAHRDSWVGPTSDCLRGWPALLRLAVPTATAVCLEWWW

YELMIVLSGLLANPRATVASMGILIQATSLVYVFPSSLGQGASTRVSHQL

GAGRPAGARRAAGAALSIGLVVGAAAATFMVSVRSHWGRMFTSDGEIL

RLTAVALPIAGLCELGNCPQTAGCGVLRGSARPASGARINLASFYLVGM

PVGVALAFGARLGFAGLWLGLLAAQAACAVWMARAVAATDWDVEVA

RAKELTKASTTGSGTNHQHECNNSNTNTANAKANTKTTTSPAANNINA

GGGGSSDNRGYVPISESGHNDGSDDLEKLEEGLMVATSGGCCGCGDAL

GVDTKAGDKQQCSNGGAGTAEGNAGQRRGSASSERAPLISVGDDEEAG

EENDGDGGGGGHV

>Os03g37411

MAGGDQRGDSPSSHELSGRLEGILADGEAPWARRACKAAALEVRLLAPI

AAPAIVVYVPNNVLSISTQIFCGHLGNLELAASSLGNNGIQIFAYGLMLG

MGSAVETLCGQAYGVHKYDMLGVYMQRSTVLLMATGVPLAVIYAFSR

PILVLLGESPEIASAAAVFVYGLVPQIFAYAANFPIQKFLQAQSIVAPSAY

TSAATLVLHLVVGWLVVYQLGMGLLGASLVLSLSWWVIVAAQFVYIA

ASKRCRRTWTGFSWMAFSGLPEFLKLSTASAVMLCLETWYFQILILLAG

LLDDPQLALDSLTVCMTLAGWVMMISIGFNAAASVRVGNELGAGHPRA

AAFSVVVVTAVSFVITVVMAVVFLMFRDYISYIFTEGETVARAVSDLCPF

LAATLILNGIQPVLSGVAVGCGWQKIVAYINVGCYYFVGIPLGFLLGFKF

HLGAKGIWTGMLGGTCMQTLILFWITFRTDWNKEVEEAKKRLNQWED

KKQPLLAGTVDY

>Os03g37470

MATTAASPEIAGAARLYVVGLIPQIFAYAANFPIQKFLQAQSIVAPSAYIS

AATLAAHVALSWFAVYKLGLGLLGASLILSLSWWVIVLAQFAYIVVSDR

CRLTWAGFSSKAFSGLPEFLQLSAASAVMLCLETWYFQVTVLIAGLLKD

PEIALDSLAVCMSISGWVFMVSVGFNAAASVRVSNELGAGNPRAAAFSV

KVVTSLSLIVAAIIAAIVMCLREYLSYVFTQGEEVARAVSSMTPLLAVTIV

LNGIQPVLSGVAVGCGWQAFVAYVNIGCYYIIGVPFGCVLGFHFDLGAM

GIYGGMIVGLFVQTLILVYVTFRTDWNREVGEAKKRLNKWGDIAKPLL

ANED

>Os03g37490

MGSSVKDAGGGKEEQQQQLESPLLEAAVSSGGGDGGGGHGVSGELESI

LGDETVPWARRMWAATGVEMRLMLRLAAPAVLVYMINYLMSMSTQIF

SGHLGTLELAAASLGNTGIQVFAYGLMLGMGSAVETLCGQAYGAHKY

DMLGVYLQRSTVLLMATGVPLAVIYAFSRPILVLLGESPEIASAAAVFVY

GLVPQIFAYAANFPIQKFMQAQSIMAPSAYISAATLAFHLVLSYLVVYQF

GLGLLGASLMLSISWWVIVVAQFIYIVTSRRCRLTWTGFSMLAFSGLPDF

FKLSLASAVMLCLETWYFQILVLIAGLLKDPEMALASLSVCMTISGWVF

MISVGFNAAASVRVSNELGAGNPKSAAFSVVVVTVLSFFLSVVISLVILL

CRDYISYIFTDGEDVATAVSKLTPLLALTLILNGIQPVLSGVAVGCGWQA

FVAYVNVGCYYIVGIPLGCLLGFYFDLGAAGIWSGMIGGTLMQTLILMW

VTFRTNWNREVEEAMKRLNKWEDKTPLLSE

>Os03g37640

MAGGVSDGGAAHGASGRLESILSDSSMPLARRAWAATTIELGLLTRIAA

PAVVMYMINYLMSMSTQIFSGHLGNLELAAASLGNNGIQMFAYGLMLG

MGSAVETLCGQAFGAHKYDMLGVYLQRSAVLLTITGVPLAVIYGFSEPI

LVFMGQSPEIARAAAIFVYGLIPQIFAYAINFPIQKYMQAQSIVLPSAYISA

ATLALHVLLSWVVVYKVGLGLLGASLVLSISWWVIVAAQFAYIVTSPTC

RHTWTGFTWQAFAGLWDFLKLSAASAVMLCLESWYFQVLVLIAGLLPN

PELALDALSVCMTISGWVFMISVGFNAAASVRVSNELGAGNPKAAYFSV

WVVTISCAIISAILAVVILCLRNYISYLFTEGEVVSNAVADLCPLLAITLIL

NGIQPVLSGVAVGCGWQQFVAYVNIGCYYIVGVPLGVLLGFVFKLGVK

GIWGGMLGGTCMQTAILVWVTLRTDWNNEVEEAQKRLNKWEDKKKE

PLLTGIRDNN

>Os03g42830

MGKLVSKSWQESKLLWHIAFPAILTAVFQFSIGFVTVGFVGHIGQVELAA

VTVVENVIEGFAYGVLLGMGSALETLCGQAVGAGQVSMLGVYIQRSWII

CGATAVILTPTYVFTAGILIGLRQPTDIAAVAGTYTRWVIPQLFAYAANF

PLQKFFQSQSKVWAMTAISGIALALHVVLNYIFLTRLGHGLVAAALIGN

VTWWLIILAQFIYLVSGCFPEAWKGFSMLAFKNLAAFVKLSLASAIMLC

LELWYYTAVLILVGLLKDAKLQVDVMSVCINYQLWTLMVALGFNAAV

SVRVSNELGANRPKAAKFAVAMAVSTSAIVGAVFMAVFFIWRTQLPRFF

SDDADVVRESAKLGYLLAATIFLNSIQPVLSGVAIGAGWQSLVAFINIGC

YYLVGIPLGVLFGFKLKLDAMGIWVGMSLGTLLQTAILAFISFRTKWER

QAMMAEERIREWGGRNDDALPSTTTPTADDHNVDR

>Os03g62270

MAIPLQGKAQQQQGEGGKGDGAVDDDGDDQPSVASELRELWGMAAPI

TALNCVVYLRAMVSVLCLGRLGPLDLAGGALAIGLTNITGHSVLFGLAS

GLEPLCAQAFGSKNYDLLTLSLQRAVLLLTLAALPIALLWLHVGPILVAL

GQDPTISASAAAYAAYALPDLAASAVLQPLRVYLRSQGITKPMAACSAI

AVALHVPLNVLLVFGLGFGVRGVAAAQALTNTNMVLFLLAYIRWSRAC

DATWKGWARPAAVASGLAGLVRLAVPSCVGVCLEWWWYEVVTVLAG

YLPDPAAAVGAAGVLIQTTSLMYTVPMALAACVSTRVGNELGGGKPRR

ARMAAMVALGCAVVIGVVHVAWTAAFSREWVELFTREAAVVRLAAA

AMPILGLCELGNCPQTTGCGVLRGTARPAVGARINLLSFYLVGTPVAVT

LAFGARVGFGGLWYGLLSAQAACVALVLLAVVWRTDWHLEALRAKK

LTGLEMIAAAAEGDDDECKRLIAPLPPPDGHDVAVDVV

>Os03g64150

MTPPPPSPPHERKTWAESVASEFRAQRGIAFPLIAMNLTWFAKLAVTTAF

LGRLGDLQLAAGTLGFSFANVTGFAVLTGLCAAMDPICGQAHGASNGK

LLRKTLVMATILLLGASIPIAFLWLHVDAVLLRFGQQADMSSNARSYVV

CLLPDLAVTSFVNPLKSYLSAQGVTLPTLFASALALALHVPLTMWMART

RGIQGVATAVWVSDLAVAVMLAGYVLVSERRRKAGGGGGWVEQTRG

EWVRLLRLAVPSCLNTCLEWWCYEILVLLTGRLPDARRTVAVMAVTLN

FDYLLFAGMLSLSVSASVRVSNELGAGEAWAARRAGMVSIVGGAVGG

VGGGVAMVAARRAWGSIYSSDAGVREGVGRAMEVMAVLEVVNFPLN

VCGGIVRGTARPAVGMYAVVAGFYVLALPLGVALAFKARLGIQGLLLG

FLVGAAASLAVLLTFIARMDWPAEAQKARTRTTATVAQFHQHDEVVQP

>Os04g30490

MAAPPSMEEPLLGGGNGEEKKGGSSRLAVVAEVRKQLYLAGPLIAGWL

LQNVVQMISVMFVGHLGELELSSASIATSFAGVTGFSLLAGMASSLDTLC

GQAFGAKQHRLVGVYKQRAMVVLGLASVCVAAVWAYTGELLLLFGQ

DPEIAAAAGSYIRWMIPALLAYGPLQCHVRFLQTQNAVMPVMLSSGAA

AACHLPVCWLLVYGAGLGSKGAALANAVAYLANAAALAAYVRLSPAC

RSTWTGFSSEAFHDLVGFMRLAVPSALMVCLEWWSFELLVLLSGLLPNP

KLEASVLSICLNSGSLAFMIPFGLGSAISTRVSNELGAGRPEAARLASRVV

MALGLVVGVAIGLAMILVRHLWGYAYSNEEEVVQYVAKMMPILAVSF

LFDDLQCVLSGVARGCGWQKIGAIVNLGAYYLVGIPAALCFAFVYHLG

GMGLWLGIMCALIVQMLLLLAITVCTNWEKEALKAKERVFSSSLPADM

T

>Os04g48290

MTTCADDQTGCAFFAPLLSSKGAEVVILVAGDEAEEQQPAPVLTSKPPG

RLAKAVNEAWSVSLGVAFPVTPSMFTCSARGEARSILGLAFPMILTGLLL

YLRSMISMLFLGHLGGLALAGGSLAIGFANITGYSVLSGLAMGMEPICG

QAFGAGNYALLGVTMQRTVLLLIAAAIPIGGLWVQMRPLLLFCGQDAAI

AAVAETYIFASLPDLVLQAFLHPVRIYLRTQSINLPLTVCAGLAIAIHLPIN

YVLVVVLGLGVKAVALASVLANLNLVLFLLAYIFLKGVHKRTGGFLLS

AESFRGWGELISLALPSCVSVCLEWWWYEIMILLCGLLLNPQATVASMG

ILIQTTSLIYIFPSSLSFGVSTRVSNELGAGQPEEASRAATVGLVLGFGFGA

FASAFAFLVRNVWASMFTADPAIVALTASVLPILGLCELGNCPQTTGCG

VLRGSARPKDAASINLRSFYLVGTPVALVMAFWFHLDFRGLWFGLLAA

QATCTVRMLLVIGRTDWAAEAKRSKQLTGAGAANMESDDRVAADEKS

RLPVDTDVERSSDHTDRC

>Os05g48040

MEERIPLLSKRFPADGTAGVGGGREEEGGDRWWSGLAREAGKVGSMA

LPMAAMSVAQNAVQVASNMMVGHLPGVLPLSASAIATSLASVSGFSLL

VGMASGLETLCGQAYGAKQYDKLGVQTYRAIVTLTVVTIPISLLWVFIG

KLLTLIGQDPVISHEAGRYIVWLIPGLFAYAVCQPLTKFLQSQSLIFPMLW

SSIATLLLHIPLSWLLVFKTSMGFTGAALAISISYWLNTFMLAAYIRFSCS

CKVTRSPPTIEAFRGVGLFLRIALPSALMLCFEWWSFEILVLLSGLLPNPE

LESSVLSICLTTTSLMYTIPYGLGGAASTRVANELGAGNPEGARSAVHLV

MSIAGTEAVLVTGMLFAAQRILGYAYSSDEEVVTYFTSMVPFVCISVAA

DSLQGVLSGIARGCGWQHLGAYVNLGSFYLVGIPVALLLGFGFKMEGK

GLWLGIACGSVLQFLLLAVIAFFSNWQKMAEKARERIFGETPSEKQHLV

LDATNSV

>Os06g29844

MAAKGSPEEEALLAGVGGDHQLVESDELAPAAAVVREEVKKQLWLAV

PLVAGALLQNVIQMISVMFVGHLGELPLAGASMASSFASVTGLSLLLGM

ASALDTLCGQAFGSRQYHLLGVYKQRAMLLLTAVSVPLVVVWFYTGDI

LVAFGQDADIAAEAGAYARWMIPALFAYGPLQCHVRFLQTQNVVLPVM

ASAGAAALCHLVVCWALVYAAGMGSKGAALSNAVSYWINVAILAVYV

RVSSSCKKTWTGFSMEAFHDPLSFFRLAIPSALMVCLEMWSFELIVLLSG

ILPNPKLETSVLSISLNTAAFVWMIPFGLGSAISTRVSNELGAGRPRAARL

AVRVVVFMAVSEGLVIGLVLVGVRYIWGHAYSDEEEVVTYVAKMMLV

IAVSNFFDGIQCVLSGVARGCGWQKIGACVNLGAYYIVGIPSAYLIAFVL

HVGGMGLWLGIICGLLVQVLLLMAITLCTNWDKEAANAKDRVFSSSLPS

DLAT

>Os06g29950

MASALDTLCGQAFGAQQYHLLGIYKQRAMLLLTAVSVPLAVVWFYTG

DILRLFGQEADIAAEAGTYARWMIPALFAYGLLHCQIRFLQTQNVVLPV

MAAAGATALCHLLVCWVLVYAAGMGNRGAALSNAVSYWINVAILAV

YVRVSSSCKKTWTGFSTEAFRDALGFFRLAVPSALMVWSSSQPKAADV

RAINQPQHCFLGVDDPLWPRLCHKSKQEIERDGRWTPILRRPKGSGWIG

AAIAIITTGNHRLVTATISTTHAIFTIRGDENIGGFTWQLGVIFDKYGERFI

NRDNHHEVFILGVQTFEHVVGRLIEVSVELAITFNRRTRVSNELGAGRPH

AACLAVRVSVFMAISEGLVIGLVLISVRNIWGHAYSNEEEVVKYVGKVL

LVISVSNFFDGIQCVLSGVARGCGWQKIGACVNLGAYYIVGIPSAYLIAFI

LHLSGMGLWLGITCGILVQVVLLMAFTLCTNWDKEAANAKHRALNSSL

PSDTAT

>Os06g29994

MEKASCLEEALLLPESCKEEEITASDEVKRQLRLAGPLIAGSLLQNLIQMI

SVMFVGHLGELPLAGASMASSFAAVTGFSLLLGLASALDTLCGQAFGAR

QYHLLGVYKQRAMLLLTAVSVPLAVAWYYTGDILLLFGQDADIAAEAG

AYARWMIPALFAYGPLQCHVRFLQTQNMVVPVMAAAGAAALCHLGVC

WALVHAAGMGSRGAALGNAVSYWINVGVLAVYVRVSRSCKKTWTGF

SMEAFRDPLSFFRLAIPSALMVCLEWWSFELLVLLSGLLPNPKLETSVLSI

TLNTANCLFMIPFGLGAAISTRVSNELGAGRPRAARLAVRVVTLLATLEG

LGMALVLACVRYVWGHAYSNEEEVVAYVAKMMLVLAVSNFLDGIQC

VLSGVARGCGWQKIGACINLGAFYVVGVPAAYLAAFVLRAGGLGLWM

GIICGVAVQTLLFLAITSRTDWQKEAKMAKDRVFSSSLPTDLAT

>Os06g36330

MCTTTSAPSVPEVATPADGGGHHVYVSLPQCTDGGDVEGGHCRPVVHQ

VKCRGGDDDGGGGGRGGGVVMPAAGETVREAAALCRLACPIALTALM

LYSRSALSMLFLGSLGDLPLAAGSLAVAFANITGYSVLSGLSLGMDPLCS

QAFGARQPRLLGLTLYRSVLFLLCCSLPLSALWLNMAKILLFLGQDRDIT

AMAQDYLLFSLPDLFSFSLIHPLRVYLRSQGITQPLAVAAAAAVVFHVPA

NYVLVGRLRLGAPGVAAAASASNFVLLAVLLAYVARRDEALREAGGPT

AEWLAGWGPLARLAAPSCVSVCLEWWWYEVMILLCGLLPEPRPAVAS

MGVLMQTTALVYVFPSSLGFGVSTRVGNELGANRPGRARAAAHVAVA

GAAAMGLAAMAFATGMRHAWGRLFTADADILRLTAAALPVVGLCELG

NCPQTVGCGVLRGTARPARAAHVNLGAFYLVGMPVAVVLAFGLGVGF

VGLWVGLLAAQVCCAGLMLCVVGSTDWEAQARRAQALTSSAAVSGK

ADAAEGGGRWPEKGEHQEWEKRRHVALISSEEADPETAEVL

>Os06g49310

MSGGGGEVEAAAEAAPLLVPHDPQPAVGAEVRRQVGLAAPLVACSLLQ

YSLQVVSVMFAGHLGELSLSGASVASSFANVTGFSVLLGMGSALDTFCG

QSYGAKQYDMLGTHAQRAIFVLMLMGVPLAFVLAFAGQILIALGQNPEI

SSEAGLYAVWLIPGLFAYGLLQCLTKFLQTQNIVHPLVVCSGATLVIHIL

LCWVMVHCFDLGNRGAALSISLSYWFNVILLAIYVKVSEVGRRSWPGW

SREALKLKDVNMYLRLAIPSTFMTCLEYWAFEMVVLLAGFLPNPKLETS

ILSISLNTMWMVYTIPSGLSSAISIRVSNELGARNPQAARLSVFVSGIMCL

TEGILVAIITVLVRDIWGYLYSNEEEVVKYVAAMMPILALSDFMDGIQCT

LSGAARGCGWQKVCSVINLCAYYTIGIPSAVTFAFVLKIDGKGLWLGIIC

AMTVQILALVVMLLRTSWNEEAEKARARVQGSDGRITLA

>Os07g01750

MQDQRAGPAKSKEKKRVSRHKQAKLTTVIIKAVPLTTKELRQKKSELRV

ERRRVKMGGDERAVAAPLLQQQQDGGGGDGERRRRRWWWGWWDGE

EAAGQLAFAAPMVATSMAYYAIPLVSVMYAGRLGELELAGATLGNSW

GTVTGIALMPWLNIRGVLAAKGLQKDKLDTGLSGSLETLCGQGYGAKM

YHMMGVYLQASIITSAFFSVLVSLLWFYSEPVLIFLRQDPEVTRTATLFLR

YSIPAQFAYGFIQCTLRFLQTQSVVTPLVVFALLPLVLHFGITHAFVHYLG

FGYAGAGMSTSVSLWLSFLMLAAYVCLSERFKHTWEGFSTEAFRHVLP

GLKLAIPSAVMVCFEYWAFEVLVLVAGLMPNSHMSTSIIAMCENTEAIS

YMITYGFAAAISTRVSNELGAGNVAKAKKALAVTLVLSLLLGVAFLLLL

GLGHDLWAGLFSKSDAVISEFASMTPLLIGSVVLDSTQGVLSGVSRGCG

WQHLAAWTNLVAFYIVGLPLSILFGFKLGLQTKDQMGKIRADNERQRR

RFCLLRLERSELTSLAAAYASLVESNIEVKSFRA

>Os07g31884

MSHFRQVRGAHAASSLLPFSHRPPRATTTTTPPRPNRCRRLHIHSSASHH

HIHNQLRHRRASHHRRREIVVVVVRCSGRDDDGVMASTDPLLGGKEEE

EGGGEVRRARRWWVGRVVDTEEAWAQTRFAVPMVLTNMSYYAIPLVS

VMFSGHLGDVHLAGATLGNSWATVTGYAFVTGMSGALETLCGQAYGA

RMYRMLGLYLQSSLLMSAAVSVLVSALWCFTEPLLLLLRQDPAVSAAA

SAFVRAQVPGLFAFSFLQCLLRYLQTQSVVAPLVACSLAPFLLHVALAH

LLVNALGLGLAGAGAAVSITFWASCLMLLAYVLRSERFAETWNGFSAE

AFRFVVPTIKLATPSAVMVCLEYWAFELLVLIAGLLPNPTVSTSLIAMCSS

TEAIAYMITYGFSAAVSTRVSNEIGAGNVEGAKNAVAVTLKLSVFLAAA

FVLLLGFGHGLWAGLFSGSAIIAAEFAAVAPLMMASILLDSAQGVLSGV

ARGCGWQHLAAVTNLVAFYVIGMPLSIFFAFKLKWYTKGLWMGLICGL

TCQTCTLMVITARTKWSKIVDAMQEKKASYVA

>Os07g33310

MGHAVDGRLEALLSGGGGGGEAAAPWARRMAAAAALELRLLAPLAAP

AVVVYMLIIVMSSATQIFCGQLGNVQLAASSLGNNGIQVFAYGLMLGM

GSAVETLCGQAYGAGRHEMLGVYLQRSAVLLTAAGVPLAALYACSER

VLLLLGQSPEISRAAAGFARGLIPQIFAYAANFPIQKFLQAQSIVAPSAAV

LAASFALHLPLSWAAVRVLGLGLPGAALALSATWWVLVAGQFAYIVRS

PRCAATWTGFTWAAFHDLAAFARLSAASAVMLALEVWYFQVLILLAG

MLPDPQIALDALTVCTSIQSWVFMISVGFNAAASVRVGNELGAGNPRSA

AFSTWMVTALSAIIAAIAGVVVILLRDKLSYIFTQGEAVSRAVSDLCPLL

VGTIVLCGIQPVLSGVAVGCGWQALVAYINIGCYYLIGLPLGVLLGFKFD

YGIKGLWGGMIGGTLIQTLILIWITFRTDWNKEVEDARRRLDKWDDTKQ

PLLVNRQ

>Os08g37432

MEDEATSVAAPLLRPRGGVDAEAVKQQLWPAGARVAGEWWVESKKL

WRVVGPAIFQRIALYGINVVSQAFIGHMGDLELAAFSIASTVVAGFNFGF

LLGMASALETLCGQAFGAKKYHMLGVYLQRSWLVLLMFAVALTPTYV

LMEDLLLLIGQPADLASLAGKMSVWLLPQHFAMAMLLPLTRFLQSQLK

NWVTAVTAGVALALHLVITYLLVNTLHLGLLGAVAAANVAWWIVVLG

QLVYVVGGWCPLSWKGFSMEAFADFWEFIKLSSASGVMLCLENWYYR

VLVLLTGYLNNAEIAVDALSICLTINGWEMMIPFGFLAATGVRVANELG

AGSGKGARFAIVVSVTTSVAIGLVFWCLIIAYNDKIALLFSSSKVVLDAV

SDLSVLLAFTVLLNSVQPVLSGVAIGSGWQALVAYVNVGSYYLVGVPIG

AILGWPLHFGVGGIWSGLIGGTAVQTLILAYLTISCDWDEEAKKASTRM

EVWASSK

>Os08g43250

MCHCSSKVVAVAQCHQPLLPPPEACPALHDRPRSARGGGGAIAEVASIV

RLAMPMVGAGLLMYMRSLVSMLFLGRLGRLPLAGGSLALGFANITGYS

VLSGLAAGMDPVCGQAFGAGRTSVLAAALRRTVVLLLAASVPIAALWL

AMHRVLVAAGQDPDIAACAYEFILCSLPDLAVQSFLHPLRVYLRAQSITL

PLTYAAAAALALHVPVNVLLVHGLGLGIRGVALAAVWTNLNFLLFLVA

YAYFSGLIRGDDDDDGGNGKAGEEGATTTTTTMEWGWLVKLSVHSCM

SVCLEWWWYEIMVLLCGVLADPKAAVAAMGILIQTTSLLYIFPHSLSCA

VSTRVGHELGAGRPERARLAARVGLACGAALGVVACAFAASLRGVWA

RMFTADATILRLASSALPILGAAELGNCPQTVGCGVLRGSARPGRAARIN

VSAFYGVGMPAALALAFWPARLDFRGMWAGMLAAQLVCAALMLLAV

RRTDWDEQAARAREITGAVAGVVVGDGDVVVKGDHADAAKVKADSG

LLVVTVLS

>Os08g43654

MSSTSGSAWDHSNNGGGGSPELREALLLGDGGSSPESREIKGIAVKKQD

DLEEIRSVGELMRLAAEENRRLWYLAGPAIFTSLAQYSLGAVTQVFAGH

LTTLELDAVSTENMVIAGLAFGIMYGMGSALETLCGQAFGAKQHHMLG

IYLQRSWVILTAMSVILLPIYLFATPILRFFHQDDEIAVLASRFSLYMIPQL

FAYALNFPIQKFLQAQSKVMAMAAVSAAVLLFHVALTWLLLVPLRMGL

VGLAVALNVSWWLVVLGQLAYIVMGYCPGAWNGFDWLAFTDLLSFAR

LSLGSAIMICLEFWFYMFLIVIVGNLPNAQVAVAAVSICTNLFGWQIMVF

FGFNAAISVRVSNELGAGRPRAARLAIAVVLVSSVAIGVAFFAAVLLLRD

VYGAPFTGSPEVVRAVASLGVVFAFSLLLNSVQPVLSGVAVGAGWQWL

VAYINLGCYYCVGIPVGYAIAFPLRRGVQGMWGGMLTGVGLQTAILVAI

TARTNWNKEASEAHARIQHWGGTAKLAVDDPI

>Os08g44870

MGSVSPPAPEEDAAAVESAGAAARMFWHETKRLWAIGTPIAIGTITNYAI

SSVTTMFIGHLGNLPLAAASVGLSVFATFALGFLLGMGSALETLCGQAF

GAGQVSMLGVYLQRSWIILLGATVLMVPVYVLAEPLLLLVGQDPEVAR

AAGRFTLYILPGAFAFAVNFPSGKFLQAQSKVGVLAWIGVAGLAFHVGI

TYLAVSVLGWGLPGAAAAYDVSQWASSLAQVAYIMGWCREGWRGWS

MAAFHDLAAFLRLSIESAVMLCLEIWYLGLITVLTGDLDDAQMAVDSLG

ICMNINGYEGMIFIGLNAAISVRVSNELGSGRPRAAMHAVVVVVAESLLI

GLLCMALVLAFSDKLALVYTSDAHLLRAVSRIAGLLGVTMVLNSVQPV

LSGVAVGGGWQGLVAYINLACYYLFGLPVGYLLGYYFNLGVGGVWGG

MLCGVALQTLILLFVVWRTDWKAEAAQASARVHKWGGTDETKPLLQG

DHSDRD

>Os09g29284

MASVPLLAEWPAGKEKEEGRVRRRLPALAREAWEESKKLWEIVGPAVF

LRLVLYSFNIISQAFAGHIGDLELAAFSIANNVITGLNFGFLLGMASALET

LCGQAYGAKQCSMLGIYLQRSWIILFVFAVLLVPTYVFTAPLLEALGQPA

ALARKAGMVSVYMLPSHFQYAVLLPLNKFLQSQRKNWVTVVTAAAAF

PVHIAVSWLLVSRLRFGVLGAAMSLGVSGWLVTLLQLAYVVGGGCPVT

WSGFSPLAFVDLWGFVKLSVSSGVMVCLETWYYKILILLTGHLKNSELA

VNALSICMSFQSWEMMIPVGFLAGTGVRVANELGAGNGKGAKFATIVS

TTTSFLIGLFFSALALAFHDKIALVFSSSNAVIDAVDNISFLLAVTILLNGV

QPVLSGVAIGSGWQAAVAYVNIGCYYFIGVPIGVLLGWSFNLGVFGIWA

GMIAGTAIQTIILAHMTIQCDWNKEVLQASERVQRWGNPK

>Os09g35600

MASRHSDEATQCHQQLLVMPAATASYPKLHDRPRLAGAAAGVLGEVA

SILCLAGPMVGAGILLYLRSLVSMVFLGRLGQLPLAGGSLALGFANITGY

SVLSGLAGGMDPVCGQAFGAGRTDLLRAALRRTVVLLLAASVPISALW

VAMHRVLVATGQDPDIAATAYAYILCSLPDLAVQCFLHPIRIYLRAQSVT

LPLTYAAAAALLLHVPINVVLVDRLGLGIRGVALGAVCTNLNCLLFLAA

YVCLSGMYGGRAKACASAAAPAAGEEDDDGGVREWWSLVRLSVHSC

MSVCLEWWWYEIMVLLCGVLADPKAAVAAMGVLIQTTSLIYIFPHSLG

CAVSTRVGHELGAGRPERARLVARVGVGLGAALGIVAFGFAVSVRAAW

ARMFTAEDAILRLAAAALPLLGAAELGNCPQTAGCGVLRGSARPERAA

RINVAAFYGVGMPVALALAFWPAGLDFRGMWGGMLAAQLVCAWLML

RAVLGTDWAEQAERARELTGGGDGYAAVAAVIVDDDKAKQHAEMDK

PQQVDNTLLMAIDCV

>Os09g37610

MELAGGGTGVVRQRAEPLGAGLLLRGGGRSVGGGGIGCARRATLRGLA

LSPLARRAVSAAGGHFLPRRAVRAAAAAGDGGFYGEEDAASDQPFPAR

ASPSDDANDSTAVRSLGGDHPGEIKKELLNLALPAIVGQAIDPVAQLLET

AYIGRLGPVELASAAVGVSVFNIISKLFNIPLLSITTSFVAEDVARHDSDQF

TSEGNMSSESGGRKRLPSISSAILLAAAIGVIEASALILGSEILLSIMGVSHA

STMHSPAKLFLSLRALGAPAVVVSLAIQGIFRGLKDTKTPLLYSGLGNIS

AVLLLPFLVYSLNLGLNGAALATIASQYLGMFLLLWSLSKRAVLLPPKIE

DLDFVGYIKSGGMLLGRTLSVLITMTLGTAMAARQGTIAMAAHQICLQV

WLAVSLLSDALAVSAQALIASSFAKLDYEKVKEVTYYVLKIGLLVGAAL

ALLLFASFGRIAELFSKDPMVLQIVGSGVLFVSASQPINALAFIFDGLHFG

VSDFSYSASSMITVGAISSLFLLYAPKVFGLPGVWAGLALFMGLRMTAG

FLRLGSRAGPWWFLHQKEPTYKLHSSTC

>Os10g11354

MKAAAMEEPLIAGSSGGAAEKNGGEEEGLVVTEIKKQLYLAGPLVVGM

LLQNVVQMISVMFVGHLGELALSSASMATSFAGVTGFSLLAGMASSLDT

LCGQAFGAKQHHMLGVYKQRAMLVLALVSVPIAAVWAFTGEILLVVG

QDPEIAAGAGSYIRWMIPTLFVYGPLQCHVRFLQTQSAVVPVMLSAGAT

AANHVLVCWLLVHRLGLGAKGAALANAVSFLTNLSVLAIYVRLSPACR

RTWTGFSGEAFRDVLGFLRLAVPSALMVCMEWWSFELLVLLSGLLANP

KLETAVLSICLNTNSFAFMVPLGLGAAISTRVSNELGAGRPQAARLATRV

VMLLAFLVGTSEGLVMVIVRNLWGYAYSNEEEVADYIAKMMPILAVSIL

FDAIQCVLSGVVRGCGRQQIGAFINLGAYYLAGIPVAFFFAFVCHLGGM

GLWFGILCGLVVQMLLLLTITLCTNWDKEALKAKDRVCSSSLPKDLAT

>Os10g11860

MEGQGLVSRSWQESKLLWRVAFPAVLVELLQFSIGFVTASFVGHLGVVE

LAAVTAVESILEGFAYGVLFGMGCALDTLCGQAVGAGRLDVLGLYVQQ

SWIVCGATAVALTPTYAFAEPILRSLLRQPADVAAVAGPYALWSLPRLF

AHAANFPLQKFFQTQSRVWALAAISAAVLAVHAALTYAAVVRLRYGLR

GAAVAGNLSYWLIDAAQFVYLVSGRFPDAWKGFTMTAFSNLAAFVKLS

LVSAIMICLEFWYYAALLILVGLLKNARLQLDIMSICINYQFWTMMVAM

GFSEAISVRVSNELGARRPKEAKFSVAVASLTSAMIGAIFMSIFFIWRTSL

PSLFSDDKEVVDGAARLGYLLAVTVFFGNIGPVLSGVAVGAGWQILVAF

VNIGCYYLVGIPFGVLFGFKLKLGALGIWMGMLTGTLLQMAILFFIIMRT

KWEAQAILAEKRISELGETTAND

>Os10g13940

MNGESLLDRSSSADAAMNDAVPGHRHHHPLSVFLRDARLAFRWDELGR

EIMGIAVPGALALMADPVASLVDTAFIGHIGPVELAAVGVSIAVFNQVSR

IAIFPLVSVTTSFVAEEDATSSDREKYEINGENEFNVSDSEMEELVSHEEA

SAAPSKSSFETDSSDVKIEHKRKNIPSVSTALLLGGVLGLLQALLLVICAK

PLLGYMGVKQGSAMLMPALKYLVVRSLGAPAVLLSLAMQGVFRGLKD

TKTPLYATVAGDATNIVLDPIFMFVFQYGVSGAAIAHVISQYFIASILLWR

LRLHVDLLPPSFKHMQFSRFLKNGFLLLARVIAATCCVTLSASMAARLG

SVPMAAFQICLQIWLASSLLADGLAFAGQAILASAFARQDHSKAAATAS

RILQLGLVLGLLLSIFLGIGLRLGSRLFTDDQDVLHHIYLGIPFVSLTQPIN

ALAFVFDGINYGASDFGYAAYSMILVAIVSIIFIVTLASYNGFVGIWIALT

VYMSLRMLAGFLRIGTARGPWTFYAAQRMHSHEVVGLC

>Os10g20350

MSSALDTLCGQAYGAGQHRLLGVYAQRAMLVLAAAAVPIALVWASAG

EILLLFGQDPAIAAEAGAYARWMIPSLAAYVPLACALRFLQAQGIVVPV

MASSGVAAVGHVAVCWALVHKAGMGSKGAALSGAVTYWTNLAVLAL

YVRLSGACETTWTGFSIDAFRELRRFTELAVPSAMMVCLEWWSFEILVL

LSGILPNPQLETSVLSICLSTSSLLFMVPRGIGSSLSTRVSNELGGGHPRAA

RMAARVAIAMTVLVCLVLVIAMIFLRNVWGNAYSSEEEVVAYIASMLP

VLAVSFFIDGINGALSGVLTGCGKQNIGAHVNLAAFYLVGIPTAVLLAFV

LHLNGEGLWLGLVCGSISKVGMLLFITLRTDWGKEAIMAKERVFSSSLP

TR

>Os10g20390

MASTAAEVKRLLRLAGPLMAGFVLRNSVQMVSVMFVGHLGELQLAGS

SLAASLASVTGFSLLSGMSSALDTLCGQAYGAGQHRLLGVYAQRAMLV

LAAAAVPIALVWASAGEILLLFGQDPAIAAEAGAYARWMIPSLAAYVPL

ACALRFLQAQGIVVPVMASSGVAAVAHVAVCWALVHKAGMGSKGAA

LSGAVTYWTNFAVLAFYARLSGACKTTWTGFSMNAFRELRRFTELAVP

SAMMVCLEWSSFEILVLLSGILPNPQLETAVLSISLSTASLLIMVPRGIGSS

LRVRTPHMTPYIISDVTHQYFISPGSKGLSQCST

>Os10g20450

MEKPAASVEEPLLVGAGEKKGESAAAAELKRLLRLAGPLVASGVLRNV

VQMVSVMFVGHLGELPLAGASLATSLANVTGFSLLFGMASALDTLCGQ

AYGARQHHLLGVYKQRAMLVLAVAAVPIALVWASAGEILLLFGQDPAI

AAEAGAYARWLIPSLVPFVPLVCHIRFLQAQSAVLPVMASCGVTAASHV

AVCWALVRKAGMGSRGAALANAVSYGVNLTIMSLYVRLSRSCEKTWT

GFSMEAFRELRQYAELAIPAAMMVCLEWWSFEFLVMLSGLLPNPKLETS

VLSICLNTGALLVMVPIGLSTAISTRVWNELGAGNPQAAKLATRVVICM

AMTEGSVVAFTMILLRNSWGHMYSDEAEVVTYIARMIPVLAISFFIDGM

HSALSGVLTGCGKQKIGARVNLGAFYLAGIPMAVFLAFVLHLNGMGLW

LGIVCGSLSKLILLFWITMSINWEKESTRAKELVFSSSLPVA

>Os10g20470

MAAAAVHEPLLAAAPPTPGKAADGDGPEEGRRLASAEAKRLLRLAGPI

VASCILQCVVNMVSVMFVGHLGELPLAGASLATSLANVTGYSLLTGMA

TAMDTLCGQAYGARQYHLLGVYKQRAMVVLAAACVPIALVWASAGRI

LLLLGQDAGIAAEAGAYARWMLPSLAAYVPLQCHIRFLQTQTVVLPVT

ASSAATALLHPLVCWLLVFRAGMGSKGAALANAISYAVNLAILAVYVR

ASNTCKGRWSGFSGEAFKELRQFAALAMPSAMMICLEWWSFEILVLLSG

LLPNPQLETSVLSICLNTGALLYMVPLGLCSSISTRVSNEIGAGQPQAAKR

ATRVVMYMALSEGLVISFTMFLLRNVWGYMYSNEQEVVTYIARMLPIL

DISFFIDGLHSSLSGVLTGCGKQKIGAAVNLGAFYLVGIPVAVLLAFYLH

LNGMGLWLGIVCGSIIKLLVLIIVSCCIDWEKEAILAKDRVFSSSLPVA

>Os10g37920

MCEALVDRQLLPPCGCNGGGDVVVVVVPKTSAAPVLEDRPKTSAAAVS

KGGEAASILRLSLPMIMTGLILYIRPMISMLFLGRLGELALAGGSLAIGFA

NITGYSVLSGLAMGMEPVCGQAVGAGNLPLVGATMQRMVLLLLAVSV

PVAFLWAWMEPLLLLCGQDAAIAAAAQRYILFCLPDLLFLSLLHPLRIYL

RVQSINLPLTACAALAVAAHLPINHLLVSVLGLGIEGVALASAWANLNL

VIFLLAFVYVSGVHRDTGGFSLPRKMFKDVDGWVRLVRLAAESCASVC

LEWWWYEIMILLCGLLANPRATVASMGILIQTTSLLYIFPSSLSFGVSTRV

SNELGANRPSAARAAARAGLALSAVQGLASLAFAVSVRGAWARMFTPD

ADILALTASVLPILGLCELGNCPQTTGCGVLRGSARPRDGAHINLGAFYG

VGTPVAVGLAFWAGMDFRGLWLGLLAAQAACVAVMLVVIQRTDWDV

QAKLAQVLAGAAASGGDHGVNEAGGNDAVAHVKVAAPHGDEDSSLLI

TVST

>Os11g03240

MERTTEDDERPTVPLLEPKPASNEEEEEVGSVRRRVVEENKKLWVVAGP

SICARFSSFGVTVISQAFIGHIGATELAAYALVSTVLMRFSNGILLGMASA

LETLCGQSYGAKQYHMLGVYLQRSWLVLFCCAVILLPVYIFTTPLLIALG

QDPEISAVAGTISLWYIPVMFSYIWAFMLQMYLQAQSKNMIVTYLAFLN

LGIHLFLSWLLTVKFQLGLAGVMGSMVISFWIPVFGQLAFVFFGGCPLT

WTGFSSSAFTDLGAIMKLSLSSGVMLCLELWYNTILVLLTGYMKNAEVA

LDALSICLNINGWEMMISIGFLSAIGVRVANELGAGSARRAKFAIFNVVT

TSFLIGFVLFVLFLFFRGSLAYIFTESKAVADEVADLAPLLAFSILLNSVQP

VLSGVAIGSGWQSVVAYVNVTSYYLIGIPLGAILGYVLGFQAKGIWIGM

LLGTLVQTLVLLFITLRTNWKKQVEITRERLNRWYMDENGRSQNSIGNA

>Os11g03484

MEKPGDDEKLTVPLLEPKPATYKHQEDDDAEEDEVGSVRRRVVEENKK

LWVVAGPSICARFSSFGVTVISQAFIGHIGATELAAYALVSTVLMRFSNGI

LIGMASALETLCGQSYGAKQYHMLGIYLQRSWIVLFCCAVILLPIYLFTT

PLLIALGQDPDISVVAGTISLWYIPIMFSYVWGLTIQMYLQSQSKNMIVT

YLSLLNFGLNLFLSWLMVVKFHLGLAGVMGSMVIACWIPIFGQLAYVFF

GGCPQTWTGFSSSAFTDLGAIIKLSISSGVMLCVELWYNTILVLLTGYMK

NAEVALDALSICLNINGWEMMIAIGFLAATG

>Os11g03500

MERPGDEHDDCRTVPLLEPKHAHGEGSNNKQEEDEEEVGSLGRRVLVE

SKKLWVVAGPSICARFSTFGVTVISQAFIGHVGATELAGYALVSTVLMRF

SGGILLGMASALETLCGQSYGAKQYHMLGIYLQRSWIVLLCCAVLLLPI

YLFTTPLLIFLGQDPKIAAMAGTISLWYIPVMISNVGNFTLQMYLQAQSK

NMIVTYLAMLNLGLHLFLSWLLTVQFYLGLAGVMGSMLAFVFFGGCPL

TWTGFSFAAFTELGAIVKLSLSSGVMLCVELWYNTILVLLTGYMKNAEI

ALDALSICLNINGWEMMISIGFLSAKGVRVANELGAGSARRAKFAIFNV

VTTSFSIGFMLFVLFLIFRGRLVYIFTESTVVADAVAELSPLLAFSNLLNSI

QPVLSGVAVGSGWQSVVAYVNVTSYYLFGIPIGVILGYVLGFQVKGIWI

GMLLGTLVQTIVLLFITLRTDWEKQVEIARQRLNRWSMDENGRQQNPG

ENP

>Os12g01580

MTATSPPMRSVAAAALVLTPTPTLNRLSFPFAHRHCPSTAAPRWRPARC

RGKPAVEDVVHDDEEETWRREANPERKDGGEEMLGRGWFMVDEIGME

ILTIALPAVLALAADPITALIDTAFVGHVGSTELAAVGVSISIFNLVSKLLN

VPLLNVTTSFVAEQQAVDADYNSSVENSHIGEEISISQEKAGEQRKFLPA

VSTSLALAAGIGLMETVALILGSGTLLDIVGVPVDSPMRIPAEQFLTLRAY

GAPPVIVALAAQGAFRGFMDTKTPLFAVVAGNLVNALLDAIFIFPLGLG

VSGAALATVTSEYLTAFILLWKLNSKIVLFSWNIVSGDIIRYLKSGALLIA

RTIAVVLTFTVSTSLAAREGSVPMAGYEICLQVWLTISLLNDALALAGQA

LLASEYAKGNYKKARIVLYRVLQIGGVTGAALSTTLLLGFGYLSMLFTD

DAAVLDVAQTGVWFVTVSQPINAVAFVMDGLYYGVSDFAFVAYSTLFA

GAISSAVLLVAAPKFGLGGVWAGLTLFMSLRAIAGFWRLGSKGGPWKII

WSETE

>Os12g03200

MERTTEDDERLTDPLLEPKPAINGGGGGSNEEEEEVGSLGRRLVEENKK

LWVVAGPSICARATSFGATVVSQAFIGHIGATELAAYALVSTVLMRLSV

GILIGMASALETLCGQSYGAKQYHMLGIYLQRSWIVLFCCAVILLPIYLFT

TPLLIALGQDPDISVVAGTISLWYIPIMFSYVWGLTIQMYLQSQSKNMIVT

YLSLLNFGLHLFLSWLMVVKFHLGLAGVMGSTVIACWIPIFGQLAYVFF

GGCPQTWTGFSSSAFTDLGAIIKLSISSGVMLCVELWYNTILVLLTGYMK

NAEVALDALSIW

>Os12g03230

MGSMVISFWIPVFGQLAFVFFGGCPLTWTGFSSSAFTDLGAIMKLSLSSG

VMLCLELWYNTILVLLTSYMKNAEVALDALSICLNINGWEMMISIGFLS

AIGVRVANELGAGSARRAKFAIFNVVTTSFLIGFVLFVLFLFFRGSLAYIF

TESKAVADEVADLAPLLAFSILLNSVQPVLSGVAIGSGWQSIVAYVNVTS

YYLIGIPLGAILGYVLGFQVKGIWIGMLLGTLVQTLVLLFITLRTDWKKQ

VEITRERLNRWYMDENGRSQNSIGNA

>Os12g03260

MERPGDEHDDCRTAPLLEPKHAHGEGSNNDKQEEDEEEVGSLGPRVLV

ESKKLWVVAGPSICARFSTFGVTVISQAFIGHIGATELAGYALVSTVLMR

FSGGILLGMASALETLCGQSYGAKQYHMLGIYLQRSWIVLLCCAVLLLPI

YLFTTPLLIFLGQDPKIAAMAGTISLWYIPVMISNVGNFTLQMYLQAQSK

NMIVTYLAMLNLGLHLFLSWLLTVQFHLGLAGVMGSMVIAYWIPVFGQ

LAFVFFGGCPLTWTGFSSAAFTELGAIVKLSLSSGVMLCVELWYNTILVL

LTGYMKNAEIALDALSICLNINGWEMMISIGFLSATGVRVANELGAGSA

RRAKFAIFNVVTTSFSIGFMLFVLFLIFRGRLAYIFTESKVVADAVAELSP

LLAFSILLNSIQPVLSGVAVGSGWQSVVAYVNVTSYYLFGIPIGVILGYVL

GFQVKGIWIGMLLGTLVQTIVLLFITLRTDWEKQVEIARQRLNRWSMDE

NGRQQNPGENP

>Os12g36660

MEELKLMRRLCLPISALNLLHYVKSMVTVLCLGRLGRAELAGGALAVG

LTNVTGYSVLSGLALGLEPLAGQAFGSGTGRTRSRPRRALRRAVLLLLA

ASFPVAALWACAGPAARAARQDAAVARAAGSYCRYAIPDLAAASVLLP

ARVYLRSKGETRRLASCAALAVALVHAPATAYLGARLRVPGVAMAAC

MTSFATLAFLWISLTWAPAQNEPDEPADLEEWAGVGQWAEWADLLRLS

LPSCLSVCLEWWWYELMTIAAGYLRDPHTALATAAIVIQTTSLLYTIPVT

LSSAVSTRVANELGAGRPRSAQVSFVVAMGIAMMGSCVGLTWTTFGRG

LWVQVFTTDPTIQSLTTSVLPVIGLCELANCPQTTGCGVLRGSARPAVGA

AINLYSFYLVGAPVALVLAFGLDMGFLGLCLGLLSAQVKIVNL

>Os12g42130

MAETSSARSPLLDVDESSGASEELLRREPVPRSVLSRLAAWEAGNLWRIS

WASILITLLSFTLSLVTQMFVGHLGELELAGASITNIGIQGLAYGIMIGMA

SAVQTVCGQAYGARKFRAMGIVCQRALVLQFATAIVIAFLYWYAGPFL

RLIGQAADVAAAGQLYARGLVPQLLAFALFCPMQRFLQAQNIVNPVAYI

TMAVLIFHILISWLTVFVLGFGLLGAALTLSFSWWVLVALTWGLMVWT

PACKETWTGLSVLAFRGLWGYAKLAFASAVMLALEIWYVQGFVLLTGF

LPDPEIALDSLSICINYWNWDFQIMLGLSYAASIRVGNELGAGHPNVARF

SVFVVITASVAFSILATILVLVLRYPLSTLYTSSTTVIEAVIKLTPLLSISIFL

NGIQPILSGVAVGSGWQVVVAYVNVGAYYLIGLPIGCVLGYKTSLGAAG

IWWGLIIGVSVQTVALIIITARTNWDNEVMKAIQRLRQTAVDDGTVPIVD

DIE

**The protein sequences of 38 well-known plant MATE transporters**

>AtTT12

MSSTETYEPLLTRLHSDSQITERSSPEIEEFLRRRGSTVTPRWWLKLAVWE

SKLLWTLSGASIVVSVLNYMLSFVTVMFTGHLGSLQLAGASIATVGIQGLA

YGIMLGMASAVQTVCGQAYGARQYSSMGIICQRAMVLHLAAAVFLTFLYWY

SGPILKTMGQSVAIAHEGQIFARGMIPQIYAFALACPMQRFLQAQNIVNPL

AYMSLGVFLLHTLLTWLVTNVLDFGLLGAALILSFSWWLLVAVNGMYILMS

PNCKETWTGFSTRAFRGIWPYFKLTVASAVMLCLEIWYNQGLVIISGLLSN

PTISLDAISICMYYLNWDMQFMLGLSAAISVRVSNELGAGNPRVAMLSVVV

VNITTVLISSVLCVIVLVFRVGLSKAFTSDAEVIAAVSDLFPLLAVSIFLN

GIQPILSGVAIGSGWQAVVAYVNLVTYYVIGLPIGCVLGFKTSLGVAGIWW

GMIAGVILQTLTLIVLTLKTNWTSEVENAAQRVKTSATENQEMANAGV

>AtFFT

MDPTAPLLTHGGEVEEDYAPARSWTDVKRVLSTESAKLWMIAAPVGFNII

CQYGVSSVTNIFVGHIGEVELSAVSISLSVIGTFSFGFLLGMGSALETLC

GQAYGAGQVNMLGVYMQRSWIILFVSCFFLLPIYIFATPVLRLLGQAEEI

AVPAGQFTLLTIPQLFSLAFNFPTSKFLQAQSKVVAIAWIGFVALSLHVI

MLWLFIIEFGWGTNGAALAFNITNWGTAIAQIVYVIGWCNEGWTGLSWLA

FKEIWAFVRLSIASAVMLCLEIWYMMSIIVLTGRLDNAVIAVDSLSICMN

INGLEAMLFIGINAAISVRVSNELGLGRPRAAKYSVYVTVFQSLLIGLVF

MVAIIIARDHFAIIFTSSKVLQRAVSKLAYLLGITMVLNSVQPVVSGVAV

GGGWQGLVAYINLGCYYIFGLPFGYLLGYIANFGVMGLWSGMIAGTALQT

LLLLIVLYKTNWNKEVEETMERMKKWGGSETTSKDILASGWPSGLRRQTQ

VLVFVRGRGFKPHF

>MtMATE2

MDSHTPLLNTTAATSSSSELLELDGGDYLEVKGFKQARKVFAIETLRIWK

IALPIVFNILCQYGVNSITNIFVGHLGDIQLSAISLINSVIGTFAFGFML

GMGSATETLCGQAFGAGQVHMLGVYMQRSWIILFVTSIILLPIYIFAAPI

LKLLGQQEDMADLAGSFALLVIPQFLSLSFNFPTQKFLQSQSKVNVIAWI

GLVALIVHIGLLWLLIYVLDLGLTGAAIAFDVTSWGITLAQLVYVVIWCK

DCWNGLSWLALKDIWAFVRLSVASAVMLCLEIWYMMSLIVLAGHLDNAVI

AVDSISICMNFNGWEGMIFIGVNAAISVRVSNELGLRHPRAAKYSVYVTV

FQSLFMGIFFMGVILVTKDYFAIVFTNSKTLQVAVADLGNLLAVTMVLNS

VQPVISGVAVGGGWQALVAYINVGCYYLFGLPLGYILGNVAELGVKGLWG

GMICGILLQTLLLSGILYKTNWNKEVDNTSARVQQWGGQTVEVDSNGVDK

P

>RcMATE1

MAILICSRNKHPTTPKIANYKENHPLKAEESNSSSSATSPSKETESNSTA

TLIDGVIRSDTLPPDRISIIETTDLHPAPSTLLSNDGGDYPPIQSFEDAK

YICTLESLKLWAIAAPIAFNILCNYGVNSFTNIFVGHIGDIELSAVAISL

SVVANFSFGFLLGMGSALETLCGQAFGAGQIDLLGVYMQRSWIILFVTCC

FLLPLYVYATPILKLLGQEADIAELAGNFTIQVIPQMFSLAVNFPTQKFL

QAQSKVGVLAWIGFVALIAHVGVLYLFVSVFKWGLAGAAAAYDVSAWGIA

LAQVVYIVGWCKDGWTGLSWLAFKDIWGFAKLSIASAVMICLEIWYFMTI

IVLTGHLEDPIIAVGSLSICMNINGWEGMLFIGINAAISVRVSNELGSQH

PRAAKYSVIITCMESLLIGVTCACIVMLTKDEFSFIFTDSLEMRKAVANL

AYLLGLTMILNSVQPVISGVAVGGGWQALVAYINLFCYYVIGLPLGFLLG

YKTSLHVQGIWMGMIFGTFLQTLILIYIIYTTNWNKEVEEASERMRKWGA

H

>PtMATE

KAEESSSSSSAGSPPSEKLRSVSASPFIDGVSRSDSFVRDNHHFLETADL

HPAPSTLITNDQGGDYPPAIGFGDAKYICLLESSKLWAIAGPIAFNILCN

YGVNSFTNIFVGHIGDIELSAVAISLSVIANFSFGFLLGMGSALETLCGQ

AFGAGQVNLLGVYMQRSWIILFVACLFLLPLYVFATPVLKLLGQRKDIAE

LAGKFTIQVIPQMFSLAINFPTQKFLQAQSKVGVLAWIGLAALIIHIGVL

YLFINVFKWGLAGAAIAYDISSWGIALAQLAYVVGWCKDGWKGLSWLAFK

DIWAFVRLSIASAVMLCLEIWYFMTIIVLTGHLEDPIIAVGSLSICMNIN

GWEGMLFIGINAAISVRVSNELGSGHPRAAKYAVIVTCIESLLVGILCAV

IILATRNHFAIIFTASEEMRKAVANLAYLLGITMILNSIQPVISGVAVGG

GWQALVAYINLFCYYVVGLPLGFLLGYKTKLHVKGIWIGMIIGTCLQTLI

LVFIVYKTNWNKEVEQASERMRKWG

>BrTT12

MSSTETYEPLLRRLHSDSQITERSSPEIEEFLGRGRSTVTPRWWLRLFVW

ESKLLWKLSGASIVVSVLNYMLSFVTVMFTGHLGSLELAGASIATVGIQG

LAYGIMLGMASAVQTVCGQAYGARQYSSMGIICQRAMVLHLAAAVLLTFL

YWYSGPILKAMGQTVAIAHEGQVFARGMIPQIYAFALACPMQRFLQAQNI

VNPLAYMSLGVFLLHTLLTWLVTNVLDFGLLGAALILSFSWWLLAAVNGL

YIVMSPNCRETWTGFSARALTGIWPYLKLTVASAVMLCLEIWYNQGLVII

SGLLTNPTISLDAISICMYYLNWDMQFMLGLSAAISVRVSNELGAGNPRV

AKLSVVVVNITTVLISLLLCIVVLVFRVGLSKAFTSDKEVIVAVSDLFPL

LAVSIFLNGIQPILSGVAIGSGWQAVVAYVNLVTYYVIGLPIGCVLGFKT

SLGVAGIWWGMIAGVILQTITLIVLTLRTNWTSEVENAAHRLKASANESQ

EMATEGV

>MtAC122162

MDDNDISNNAVKNKWTMPLSVFFKDASLVFKMDSLAKEILGIAFPSALAV

AADPIASLIDTAFIGHLGPVELAAAGVSIAVFNQASRITIFPLVSITTSF

VAEEDTMDRINTKAAEKQFNESGKAKSNEVMPDDHLLQDIEAGATKQDST

LKNGDDANSNISKSSIVTNSSNKSESKPIRKKRHIASASTALLFGTVLGL

IQAATLIFAAKPLLGAMGLKYDSPMLVPAVKYLRLRALGAPAVLLSLAMQ

GIFRGFKDTTTPLYVIVSGYALNVAMDPLLIFYFKLGIRGAAISHVLSQY

IMATLLLFILMKKVDLLPPSMKDLQIFRFLKNGGLLLARVIAVTFCVTLS

ASLAARLGPIPMAAFQTCLQVWMTSSLLADGLAVAIQAILACSFAEKDYN

KVTTAATRTLQMSFVLGVGLSLVVGGGLYFGAGVFSKNVAVIHLIRLGLP

FVAATQPINSLAFVFDGVNYGASDFAYSAYSLVMVSIASVTSLFFLYKSK

GFIGIWIALTIYMSLRMFAGVWRMGTGTGPWRFLRGQSLS

>Sb-MATE1

MEEHRSPAHAKPEAEQPPQQQVPAAMAVAVAVDVAAPAALQNSTAAPAEN

GDVAAAGAAENGTAASAANGDGGGSELLGGPRWTGLHLFVMNIRSVFKLD

ELGAEVLGIAVPASLALTADPLASLIDTAFIGRLGSVEIAAVGVAIAVFN

QVMKVCIYPLVSVTTSFVAEEDAVLSKGGAKVIDNGEEEEELEAGQVGPE

KHTAAAGADPEKQQQPADEEAAKNGGEGCAPAVVAGRSSGKKSGNRRFVP

SVTSALIVGALLGLFQTVFLVAAGKPLLRLMGVKPGSPMVMPALRYLTLR

ALGAPAVLLSLAMQGVFRGFKDAKTPLYAIVAGDAANIVLDPILIFGCRL

GVIGAAIAHVLSQYLITLIMLSKLVRKVDVVPPSLKCLKFRRFLGCGFLL

LARVVAVTFCVTLAASLAARHGPTAMAAFQICTQVWLATSLLADGLAVAG

QAMIASAFAKEDRYKVAATAARVLQLGVVLGAALTALLGLGLQFGAGVFT

SDAAVIKTIRKGVPFVAGTQTLNTLAFVFDGINFGASDYAFSAYSMIGVA

AVSIPSLIFLSSHGGFVGIWVALTIYMGVRALASTWRMAAAQGPWKFLRQ

>Nt-MATE1

MGKSMKSEVEQPLLIAAHGGSSELEEVLSDTQLPYFRRLRYASWIEFQLL

YRLAAPSVAVYMINNAMSMSTRIFSGQLGNLQLAAASLGNQGIQLFAYGL

MLGMGSAVETLCGQAYGAHRYEMLGVYLQRATVVLSVTGIPLTVVYLFSK

NILLALGESKLVASAAAVFVYGLIPQIFAYAVNFPIQKFLQAQSIVAPSA

FISLGTLFVHILLSWVVVYKIGLGLLGASLVLSFSWWIIVVAQFIYIIKS

ERCKATWAGFRWEAFSGLCQFVKLSAGSAVMLCLETWYMQILVLLSGLLK

NPEIALASISVCLAVNGLMFMVAVGFNAAASVRVSNELGAAHSKSAAFSV

FMVTFISFLIAVVEAIIVLSLRNVISYAFTEGEIVAKEVSELCPFLAVTL

ILNGIQPVLSGVAVGCGWQAFVAYVNVGCYYGVGIPLGCLLGFKFDLGAK

GIWTGMIGGTVMQTVILLWVTFRTDWNKKVECAKKRLDKWENLKGPLNKE

>Nt-MATE2

MGKSMKSEVEQPLLAAAHGGSSELEEVLSDSQLPYFRRLRYASWIEFQLL

YRLAAPSVAVYMINNAMSMSTRIFSGQLGNLQLAAASLGNQGIQLFAYGL

MLGMGSAVETLCGQAYGAHRYEMLGVYLQRATVVLSLTGIPLAVVYLFSK

NILLALGESKLVASAAAVFVYGLIPQIFAYAVNFPIQKFLQSQSIVAPSA

FISLGTLFVHILLSWVVVYKIGLGLLGASLVLSFSWWIIVVAQFIYILKS

ERCKATWAGFRWEAFSGLWQFVKLSAGSAVMLCLETWYFQILVLLSGLLK

NPEIALASISVCLAVNGLMFMVAVGFNAAASVRVSNELGAAHPKSAAFSV

FMVTFISFLIAVVEAIIVLSLRNVISYAFTEGEVVAKEVSSLCPYLAVTL

ILNGIQPVLSGVAVGCGWQAFVAYVNVGCYYGVGIPLGCLLGFKFDFGAK

GIWTGMIGGTVMQTIILLWVTFSTDWNKEVESARKRLDKWENLKGPLNKE

>AtEDS5

MLIKSQRLTLFSPLLSKTRRIPVNSHQTLVAESVITRRTLGAITATPSFH

KNPVVIRRRIKLERVTRNCVRIDREIDEEEEEEEKERGDLVKQSIWEQMK

EIVKFTGPAMGMWICGPLMSLIDTVVIGQGSSIELAALGPGTVLCDHMSY

VFMFLSVATSNMVATSLAKQDKKEAQHQISVLLFIGLVCGLMMLLLTRLF

GPWAVTAFTRGKNIEIVPAANKYIQIRGLAWPFILVGLVAQSASLGMKNS

WGPLKALAAATIINGLGDTILCLFLGQGIAGAAWATTASQIVSAYMMMDS

LNKEGYNAYSFAIPSPQELWKISALAAPVFISIFSKIAFYSFIIYCATSM

GTHVLAAHQVMAQTYRMCNVWGEPLSQTAQSFMPEMLYGANRNLPKARTL

LKSLMIIGATLGLVLGVIGTAVPGLFPGVYTHDKVIISEMHRLLIPFFMA

LSALPMTVSLEGTLLAGRDLKFVSSVMSSSFIIGCLTLMFVTRSGYGLLG

CWFVLVGFQWGRFGLYLRRLLSPGGILNSDGPSPYTVEKIKSI

>VvAM1

METPLLKSGAERGYGGEGGDYPPLTTWREVRSMLWRETVKVWRVAGPLA

FQILCQFGTNLVTTVFVGHIGNLELSAVSISVSVIGTFSFGFMLGMGSA

LETLCGQAYGAGQVQLLGVYLQRSWIILLVSCIILLPIYIFATPILKAL

GQEDEIADLAGQFTLETIPQLFSLAIIFPTQKFLQAQSKVNVQATICFV

ALILDIGMLAVFIFVFGWGTTGAAIAYDISSWVTAVAQVVYAISWCKEG

WTGLTWSAFREIWAFVRLSLASAVMLCLEIWYFMIIILLTGHLQNAVIA

VGSLSICMTFGGLEVVMFMGMNAAVSIRVSNELGYGHPRAAKYSVFVAV

SQSLLIGIFCMVVVLLARDYIAIIFTTNKEMQEAVSHLAYLLGVTMLLN

SLQPVFSGVAVGGGWQAMVAYINLGCYYIIGIPLGYLLGYKAKLGVQGL

WGGMICGTALQTLVLLFIVYRTNWNREVEQTTERMQKWGGQRIEADDV

>VvAM3

METPLLNSGAEEGYSGPDGDYQPLRSWREVRSMVWKETVKLWRVAGPLA

FQILCQFGTNSMTSVFVGHIGNLQLSAVSISLSVIGTFSFGFMLGMGSA

LETLCGQAYGAGQVHLLGVYLQRSWIILLVTCVILSPVYVFATPILKVL

GQEDAIADLAGQFTIETIPQLFSLAIIFPTQKFLQAQSKVNVQATIAFV

ALILHIGMLSVFIFVFGWGTTGAAIAYDISNWVIAVSQVVYAIGWCKEG

WTGLTWSAFREIWAFVRLSIASAVMLCLEIWYFTIIVLLTGHLQNAVIA

VGSLSICMTFNGWEGMLFIGMNAALSIRVSNELGYGHPRAAKFSVYVAV

SQSLLIGILCMVVVLLARDYIAFIFTSNKEMQEAVSNLAYLLGATMLLN

SMQPVLSGVAVGSGWQALVAYINLGCYYIIGVPLGCLLGYLAKFGVKGL

WGGMICGTALQTLILLFIVYRTNWNKEVEQTTERMQKWGGVQIETKKTS

DDV

>MtMATE1

MENQPFLVGLDSHSHTHIADLSSDAIEEFLEHRPIGLRWWLKLVAWESR

LLWILSGASIVVYLCNFMLSFVTMMFCGHLGSLELAGASIASVGIQGLA

YGIMLGMASAVQTVCGQAYGAKKHAAMCITLQRAIILHFGAAVILTFLY

WFSGDFLKVIGQTESIAVQGQVFARGLIPQLYAFAFSCPMQRFLQAQNI

VNPLAYMAVGVLLLHALLSWLVVVVLGYGLLGAALTLSFSWWILVFLNA

LYIIFSPKCKETWTGFTMKAFIGIWPYFKLTVASAVMLCLEIWYNQGLV

LISGLLSNPTVALDSISICMNYLNWDMQVMLGLGAAASVRISNELGAAH

PRVAKFAIFVVNGNSILISVVLSAIILIFRDGLRNLFTSDSEVIEAVSD

LTPLLAISVLLNGIQPILSGVAIGSGWQALVAYVNLACYYVIGLTVGCV

LGFKTSLGVAGIWWGMILGVFIQTVTLIILTARTNWGVEVEKAIVRVKR

SAEDDTLDRLVADV

>RcEEF49069

MEAPLLTPSLSAGDDEDYTPVKSFKDIKSVFWTETVKIWKIATPIVFNI

MCQYGINSVTNIFVGHIGDFELSAVAISLSVIGTFSFGFMLGMGSALET

LCGQAFGAGQVHMLGIYMQRSWIILWITCIFLLPIYVFATPILKLLGQE

DSVADLAGQFTILIIPQLFSLAVNFPTQKFLQAQSKVRVLAWIGFVAFI

LHIPLLWLLIYVFGWGTSGAAIAYDITNWGMSIAQVVYVIGWCKEGWTG

LSSSAFKEIWAFVRLSLASAVMLCLEIWYMMSIIVLTGHLDNAVIAVGS

LSICMNFNGWEAMLFIGVNAAISVRVSNELGSGHPRAAKYSVYVTIFQS

FLIGLLSMVIILITKDHFAIIFTNSKAMQVAVSKLAFLLGITMVLNSIQ

PVIGGVAIGSGWQALVAYINIGCYYIFGLPLGFFLGYKTKLGVAGLWGG

MIAGTALQTLLLLIVLYRTNWNKEVEQTSERVRKWGGQENTEKTADGV

>AtALF5

MADPATSSPLLDDHVGGEDERGRRSRSSTLVQKVIDVEEAKAQMIYSLP

MILTNVFYYCIPITSVMFASHLGQLELAGATLANSWATVSGFAFMVGLS

GSLETLCGQGFGAKRYRMLGVHLQSSCIVSLVFSILITIFWFFTESIFG

LLRQDPSISKQAALYMKYQAPGLLAYGFLQNILRFCQTQSIIAPLVIFS

FVPLVINIATAYVLVYVAGLGFIGAPIATSISLWIAFLSLGTYVMCSEK

FKETWTGFSLESFRYIVINLTLSLPSAAMVCLEYWAFEILVFLAGVMPN

PEINTSLVAICVNTEAISYMLTYGLSAAASTRVSNELGAGNVKGAKKAT

SVSVKLSLVLALGVVIVLLVGHDGWVGLFSDSYVIKEEFASLRFFLAAS

ITLDSIQGVLSGVARGCGWQRLVTVINLATFYLIGMPIAAFCGFKLKFY

AKGLWIGLICGIFCQSSSLLLMTIFRKWTKLNVATV

>AtDTX1

MEEPFLPQDEQIVPCKATWKSGQLNVELKKVSRLAVPMATVTIAQYLLPVISVMVAGHNGELQLSGVALATSFTNVSGFSIMFGLVGSL

ETLSGQAYGAKQYEKMGTYTYSAISSNIPICVLISILWIYMEKLLISLGQDPDISRVAGSYALRLIPTLFAHAIVLPLTRFLLAQGLVLPLLYFALTTLL

FHIAVCWTLVSALGLGSNGAALAISVSFWFFAMTLSCYVRFSSSCEKTRRFVSQDFLSSVKQFFRYGVPSAAMLCLEWWLFELLILCSGLLQNPKLETSV

LSICLTTATLHYVIPVGVAAAVSTRVSNKLGAGIPQVARVSVLAGLCLWLVESSFFSILLFAFRNIIGYAFSNSKEVVDYVADLSPLLCLSFVLDGFTAV

LNGVARGCGWQHIGALNNVVAYYLVGAPVGIYLAFSCELNGKGLWCGVVVGSAVQAIILAIVTASMNWKEQAKKARKRLISSENGLA

>AtADS1

MCNPSTTTTTTGSENQESRTGLFLDLFSINSFEPTKRNLRHCENRGSPLMAEAVTEAKSLFTLAFPIAVTALVLYLRSAVSMFFLGQLG

DLELAAGSLAIAFANITGYSVLSGLALGMEPLCSQAFGAHRFKLLSLTLHRTVVFLLVCCVPISVLWFNVGKISVYLHQDPDIAKLAQTYLIFSLPDLLT

NTLLHPIRIYLRAQGIIHPVTLASLSGAVFHLPANLFLVSYLRLGLTGVAVASSITNIFVVAFLVCYVWASGLHAPTWTDPTRDCFRGWAPLLRLAGPSC

VSVCLEWWWYEIMIVLCGLLVNPRSTVAAMGVLIQTTSFLYVFPSSLSFAVSTRVGNELGANRPKTAKLTATVAIVFAAVTGIIAAAFAYSVRNAWGRIF

TGDKEILQLTAAALPILGLCEIGNCPQTVGCGVVRGTARPSTAANVNLGAFYLVGMPVAVGLGFWAGIGFNGLWVGLLAAQISCAGLMMYVVGTTDWESE

AKKAQTLTCAETVENDIIKAVVASTIDGECDEAEPLIRITVLY

>AtZF14

MCNSKPSSASSSLLSCKDKTHISKLETCDTDNPHYSEFRDTDSLDLKRWPSFLEGLEEVKAIGKISGPTAMTGLLMYSRAMISMLFLGY

LGELELAGGSLSIGFANITGYSVISGLSMGMEPICGQAYGAKQMKLLGLTLQRTVLLLLSCSVPISFSWLNMRRILLWCGQDEEISSVAQQFLLFAIPDL

FLLSLLHPLRIYLRTQNITLPVTYSTAVSVLLHVPLNYLLVVKLEMGVAGVAIAMVLTNLNLVVLLSSFVYFTSVHSDTWVPITIDSLKGWSALLSLAIP

TCVSVCLEWWWYEFMIILCGLLANPRATVASMGILIQTTALVYVFPSSLSLGVSTRISNELGAKRPAKARVSMIISLFCAIALGLMAMVFAVLVRHHWGR

LFTTDAEILQLTSIALPIVGLCELGNCPQTTGCGVLRGCARPTLGANINLGSFYFVGMPVAILFGFVFKQGFPGLWFGLLAAQATCASLMLCALLRTDWK

VQAERAEELTSQTPGKSPPLLPIASSKSRSTSGTEDMMRTMLV

>AtFRD3

MTETGDDLATVKKPIPFLVIFKDLRHVFSRDTTGREILGIAFPAALALAADPIASLIDTAFVGRLGAVQLAAVGVSIAIFNQASRITIF

PLVSLTTSFVAEEDTMEKMKEEANKANLVHAETILVQDSLEKGISSPTSNDTNQPQQPPAPDTKSNSGNKSNKKEKRTIRTASTAMILGLILGLVQAIFL

IFSSKLLLGVMGVKPNSPMLSPAHKYLSIRALGAPALLLSLAMQGIFRGFKDTKTPLFATVVADVINIVLDPIFIFVLRLGIIGAAIAHVISQYFMTLIL

FVFLAKKVNLIPPNFGDLQFGRFLKNGLLLLARTIAVTFCQTLAAAMAARLGTTPMAAFQICLQVWLTSSLLNDGLAVAGQAILACSFAEKDYNKVTAVA

SRVLQMGFVLGLGLSVFVGLGLYFGAGVFSKDPAVIHLMAIGIPFIAATQPINSLAFVLDGVNFGASDFAYTAYSMVGVAAISIAAVIYMAKTNGFIGIW

IALTIYMALRAITGIARMATGTGPWRFLRGRSSSSSS

>AtMATE

MMSEDGYNTDFPRNPLYIFFSDFRSVLKFDELGLEIARIALPAALALTADPIASLVDTAFIGQIGPVELAAVGVSIALFNQVSRIAIFP

LVSITTSFVAEEDACSSQQDTVRDHKECIEIGINNPTEETIELIPEKHKDSLSDEFKTSSSIFSISKPPAKKRNIPSASSALIIGGVLGLFQAVFLISAA

KPLLSFMGVKHDSPMMRPSQRYLSLRSLGAPAVLLSLAAQGVFRGFKDTTTPLFATVIGDVTNIILDPIFIFVFRLGVTGAATAHVISQYLMCGILLWKL

MGQVDIFNMSTKHLQFCRFMKNGFLLLMRVIAVTFCVTLSASLAAREGSTSMAAFQVCLQVWLATSLLADGYAVAGQAILASAFAKKDYKRAAATASRVL

QLGLVLGFVLAVILGAGLHFGARVFTKDDKVLHLISIGLPFVAGTQPINALAFVFDGVNFGASDFGYAAASLVMVAIVSILCLLFLSSTHGFIGLWFGLT

IYMSLRAAVGFWRIGTGTGPWSFLRS

>OsFRDL1

MAGLKKMEEVTAAAAAVAASSTAEKRAAAVVVPDAALTMNGAAGAEEKTAAAAAAPEDLPAPAALSGWPR

RVGLYLFVMNIRSVFKLDELGSEVLRIAVPASLALAADPLASLVDTAFIGRLGSVEIAAVGVSIAIFNQV

SKVCIYPLVSVTTSFVAEEDAIISKCIEENSSQDLEKASPVDSETNNLPVSGPDKVECVNSCIPTECTNP

SDQGCKRKYIPSVTSAVIVGSFLGLLQAVFLVFSAKFVLNIMGVKNDSPMLRPAVRYLTIRSLGAPAVLL

SLAMQGVFRGFKDTKTPLYATVVGDAANIILDPILMFVCHMGVTGAAVAHVISQYLITMILLCRLIRQVD

VIPPSLKSLKFGRFLGCGFLLLARVVAVTFCVTLASSLAARHGPTIMAAFQICCQLWLATSLLADGLAVA

GQAVLASAFAKNDKGKVVVATSRVLQLSIVLGMGLTVVLGVGMKFGAGIFTKDIDVIDVIHKGIPFVAGT

QTINSLAFVFDGINFGASDYTYSAYSMVGVAAISIPCLVYLSAHNGFIGIWIALTIYMSLRTIASTWRMG

AARGPWVFLRK

>OsFRDL4

MQIDELSNTCMARSSSAPESMEADHQLTATVTAAASGDMPVTEQQQQKQMVAVAAPPATENADAAGGGGG

DNGDHLPPTATSLLGGARRTGLHLFVLNARSALRLDELGAEVLRIAVPASLALTADPLASLIDTAFIGRI

GSVEIAAVGVAIAVFNQVMKVCIYPLVSVTTSFVAEEDAILSKGAAGADDDNDDGHDAKGHGASAAAVAD

PEKQQVVGVDSAETNGAEVSTAAVRTTDDKKAAAAGVGVGKCRRRFVPSVTSALIVGAFLGLLQAVFLVA

AGKPLLRIMGVKPGSPMMIPALRYLVVRSLGAPAVLLSLAMQGVFRGFKDTKTPLYATVTGDLANIALDP

ILIFTCRFGVVGAAIAHVISQYLITLIMLCKLVRKVDVIPSSLKSLKFRRFLGCGFLLLARVVAVTFCVT

LAASLAARHGATAMAAFQICAQVWLASSLLADGLAVAGQALLASAFAKKDHYKVAVTTARVLQLAVVLGV

GLTAFLAAGMWFGAGVFTSDAAVISTIHRGVPFVAGTQTINTLAFVFDGVNFGASDYAFAAYSMVGVAAV

TIPCLVLLSSHGGFVGIWIALAIYMSVRAFASTWRMGAARGPWKFLRK

>HvAACT1

MEEGAAASMMTGDKKWVAVVDVPADADAATAANGHGPEEKAAEDLPAALSGCPRTTGLYLFVMNIRSVFK

LDELGSEVLRIAVPASLALAADPLASLVDTAFIGRLGSVEIAAVGVSIAIFNQVSKVCIYPLVSVTTSFV

AEEDAIISKYLEENSSQDLEKASHVHSDACNVPASGPDTPVCANSCIPTECTDLSNQGCKKRYIPSVTSA

LIVGSFLGLVQAVFLIFSAKFVLGIMGVKHDSPMLEPAVRYLTIRSLGAPAVLLSLAMQGVFRGFKDTKT

PLYATVVGDATNIILDPILMFVCHMGVTGAAVAHVISQYLITMILICRLVQQVDVIPPSLKSLKFGRFLG

CGFLLLARVVAVTFCVTLASSLAARDGPTIMAAFQICCQLWLATSLLADGLAVAGQAVLASAFAKNDHKK

VIAATSRVLQLSIVLGMGLTVVLGLFMKFGAGVFTRDADVINVIHKGIPFVAGTQTINALAFVFDGINFG

AQDYTYSAYSMVGVASISIPCLVYLSAHKGFIGIWVALTIYMSLRTVASTWRMGAARGPWVFLRK

>Nt-JAT1

MVEELPQSLKEKKWQINWDAVSQELKKTSRFMAPMVAVTVFQYLLQVVSVMMVGHLGELALSSVAIATSL

TNVTGFSLLTGLVGGMETLCGQAYGAQQYHKLSTYTYTAIISLFLVCIPICVLWCFMDKLLILTGQDHSI

SVEARKYSLWVIPAIFGGAISKPLSRYSQAQSLILPMLLSSFAVLCFHLPISWALIFKLELGNIGAAIAF

SISSWLYVLFLASYVKLSSSCEKTRAPFSMEAFLCIRQFFRLAVPSAVMVCLKWWSFEVLALVSGLLPNP

KLETSVMSICITISQLHFSIPYGFGAAASTRVSNELGAGNPQKARMAVQVVMFLTVVETLVFNTSLFGSR

HVLGKAFSNEKQVVDYIAAMTPFLCLSIVTDSLQIVITGIARGSGWQHIGAYINLVVFYVIAIPLAVVLG

FVLHLKAKGLWIGIVVGCAIQSIVLSIVTGFTDWEKQAKKARERVHEGRS

>BoMATE

MMSEDGYTKETPCDFPRNPLCIFLSDFRSVFKFDELGLEIARIALPAALALTADPIASLVDTAFIGQIGP

VELAAVGVSIALFNQVSRIAIFPLVSITTSFVAEEDACSSEENTNQDYKECIETGINNTKEETQELIPGN

ISTPDESKNSCSIFSVSDSPVKKRNIPSASSALIIGAILGLLQAVFLISSAKPLLSFMGVKHDSPMLGPA

QRYLSLRSLGAPAVLLSLATQGVFRGFKDTTTPLYATVIGDVTNIILDPIFIFVFRLGVTGAATAHVISQ

YLMCGILLWKLMGQVDIFKMSTKHLQFCRFMKNGFLLLMRVVAVTFCVTLSASLAAREGPTSMAAFQVCL

QVWLATSLLADGFAVAGQAILASAFAKKDYKRAAATASRVLQLGLVLGFLLAVILGTGLHFGARLFTKDD

KVLHLISIGLPFVAGTQPINALAFVFDGVNFGASDFGYAAASLVMVAIVSVLCLVLLSATHGFIGLWFGL

TIYMSLRAAVGFWRIGTATGPWSFLRR

>EcMATE1

MAEDSDVRVTDSVIEESDLNTPEAMPEGPDQHDPVVVIEENDQHPLRSKWNIPISVLFRDARHVFKKDEL

GREIWGIAFPAALALAADPIASLIDTAFIGRLGPVEIAAVGVSIAIFNQASKVTIFPLVSITTSFVAEEE

TIGKTCASLEEDENPKKCSPKNIEMKELMPDDEMLEKLERGSTNNREVTDLVPTEDFSATTCKSTPIFSS

KPKKAKLSKERRHIPSASTALVLGGILGLLQTLLLIFGAKPLLSLMGIKSGSPMMTPARKYLTLRALGAP

AVLLSLAMQGVFRGFKDTKTPLYATIAGDLTNIVLDPILIFVCGLGVSGAAIAHVLSQYLISLILLLRLM

KQVNLLPPSCKDLQFRRFLKNGILLLARVIAATICVTLAASTAARLGSIPMAAFQVCLQVWMTSSLLADG

LAVAGQAILASAFAEKDYDRAIAAGVRVLQMGFVLGMGLAVLVGVGLRFGSGVFSKDINVQHLIFVGIPF

IAATQPINCLAFVLDGVNFGASDFAYSAYSMVTVSLISIASLFLLSKSTGYVGIWVALTIYMVLRALVGL

GRMGTGSGPWRFIREGLLPQRL

>GmFRD3b

MDENGSSSNAPKNKRNLPVSVFFKDARHVFKMDSIAKEILGIAFPSALAVAADPIASLIDTAFIGHLGPV

ELAAAGVSIALFNQASRITIFPLVSITTSFVAEENTIEKINTEKKLSDKAKSKEQVMLDDHSLQDIEKVA

SKENNETENVEMNDCNTSICKSTSDTSSSSSNKSVPKDGRKKRHVASASTALLFGTILGLLQATTLIFAA

KPLLAAMGLKPDSPMLNPAIKYLRLRSLGAPAVLLSLAMQGIFRGFKDTTTPLYVILSGYALNVILDPVL

IFYCKLGIKGAAISHVLSQYLMALALMVILTRKVDLVPPSIKDLQIFRFLKNGGLLLARVIAVTFCQTLA

ASLAARFGPIPMAAFQTCLQVWLTSSLLADGLAVAVQAILACSFAEKDYEKVLVAATRTLQMSFVLGVGL

SFAVGFGLYFGAGIFSKSVLVVHLIRIGLPFVAATQPINSLAFVFDGVNYGASDFAYSAYSLVTVSLASV

ASLFLLSKSKGFVGIWIALTIYMSLRMFAGVWRMGTGTGPWRFLRGRSMS

>ScFRDL1

MEEGAAASMTVREKRVAVGVPADAATAAANGHGPEEKAAEELPAPSALSGWPRTTGMYLFVMNIRSVFKL

DELGSEVLRIAVPASLALAADPLASLVDTAFIGRLGSVEIAAVGVSIAIFNQVSKVCIYPLVSVTTSFVA

EEDAIISKYLEENNSKDLEKAAHVHSDACNVPASGGDTPVCANSCIPTECADPSNQGCKRRYIPSVSSAL

IVGSFLGLVQAVFLIFSAKVVLGIMGVKRDSPMLEPAVRYLTIRSLGAPAVLLSLAMQGVFRGFKDTKTP

LYATVVGDATNIILDPILMFVCHMGVTGAAVAHVISQYLITMILICRLVQQVDVIPPSLKSLKFGRFLGC

GFLLLARVVAVTFCVTLASSLAARDGPTIMAAFQICCQLWLATSLLADGLAVAGQAVLASAFAKNDTKKV

IAATSRVLQLSIVLGMGLTVVLGLFMKFGAGVFTKDAAVIDVIHRGIPFVAGTQTINALAFVFDGINFGA

QDYTYSAYSMVGVASISIPCLVYLSAHKGFIGIWVALTIYMSLRTIASTWRMGAARGPWAFLRK

>ScFRDL2

MHLLGVFFHGATLTFERDDLGREIMGIAVPGALALMADPLASLVDTAFIGHIGPVEIAAVGVSIVVFNQV

TRIAVFPLVSVTTSFVAEEDATSSDRNKVEISGDNEHNVSEMDELITHEENNATSGKSSFETDSSEINTE

HRRKKIPSVSTALLLGGVLGLVETLLLVFCAKPILDFMGVKADTGMLKPALQYLVLRSLGAPAVLLSLAM

QGVFRGLKDTRTPLYATVAGDAINIVLDPIFMFVFQYGVSGAAVAHVISQYFIAAILLCRLSLQVELLPP

NLKHLPIGRFLKNGSLLLARVIAATCCVTLSASMAARLGSTQMAAFQICLQIWLASSLLADGLAFAGQAI

LASAFARKDHSKAKATASRVLQLGLILGLLLGLLLGVGLHTGSRLFTEDQGVLHHIYVATPFVALTQPIN

ALAFVFDGVNYGASDFAYAAYSLILVAIVSIACIVTLANYCGFIGIWIALSIYMSLRMFAGLWRIGTARG

PWAFLRS

>TaMATE1B

MEEGAAASMTVGEKRVAVDVPAGAAAAANGHGPEEKAAEDVPAPSALSGWPRTTGMYLFVMNIRSVFKLD

ELGSEVLRIAVPASLALAADPLASLVDTAFIGRLGSVEIAAVGVSIAIFNQVSKVCIYPLVSVTTSFVAE

EDAIISKYIEENNSKDLEKAAHVHSDACNVPASGGDTPVCANSCIPTECADPSNQGCKRRYIPSVTSALI

VGSFLGLVQAVFLIFSAKVVLGIMGVKHDSPMLEPAVRYLTIRSLGAPAVLLSLAMQGVFRGFKDTKTPL

YATVVGDATNIILDPILMFVCHMGVTGAAVAHVISQYLITMILICRLVQQVDVIPPSLKSLKFGRFLGCG

FLLLARVVAVTSCVTLASSLAARDGPTIMAAFQICCQLWLATSLLADGLAVAGQAVLASAFAKNDHKKVI

AATSRVLQLSIVLGMGLTVVLGLFMKFGAGVFTKDATVIDVIHKGIPFVAGTQTINALAFVFDGINFGAQ

DYTYSAYSMVGVASISIPCLVYLSAHKGFIGIWVALTIYMSLRTVASTWRMGAARGPWAFLRK

>ZmMATE1

MHPPTHMRARAPIGNSGPDSLSYFIPLHPLLGVWTMHGGRERERESLLFLPDPSAGAMEGGGEHHHPLSV

FLRDARLAFRWDELGQEIMRIAVPGALALMADPVASLVDTAFIGHIGPVELGAVGVSIAVFNQVSRIAVF

PLVSVTTSFVAEEDAMSNGRDNDKIHQQNERNVSVSEMDELIPPEGASASTSISSFETDSCEVSVEQKRK

NIPSVSTALLLGGVLGLLETLLLVLSAKPILGYMGVKPDSAMMKPALQYLVLRSLGAPAVLLSLAIQGVF

RGFKDTKTPLYATVAGDAINIVLDPIFMFVFQYGVSGAAIAHVISQYFIASILLWRLRLHVDLLPPSFKH

LQFGRFLKNGFLLLARVIAATCCVTLSASMAARLGSTPMAAFQICLQTWLACSLLADGLAFAGQAILASA

FARKDYPKATATASRILQLALVLGLLLSILLGVGLRIGSRLFTSDQGVLHHIYIGIPFVCLTQPINALAF

VFDGINYGASDFGYAAYSMVLVAVVSIICILTLESYGGFIGIWIALVIYMSLRMFAGFWRIGTAQGPWAY

LRG

>VvMATE2

MGSEEYQPLLLGLNSHARIPDLSSFAVEEFLAHKPVAVRWWPRLFGWESRLLWLLSGSSIVASIFNYMLS

FVTLMFTGHLGALELAGASIASVGIQGLAYGIMLGMASAVQTVCGQAYGAKKYKAMGIICQRAIILHLGA

AVLLTFLYWFSGPFLRAIGQSDSISAQGQIFARGLILQLYAFAISCPMQRFLQAQNIVNPLAYMAVGVFF

LHVLLTWLVVYVLDYGLLGAALTLSFSWWILVVVIALYILLSPSCKETWTGFSSKAFKGMWPYFKLTVAS

AVMLCLEIWYNQGLVLISGLLSNPTISLDSISICMNYLNWDMQFMLGLSAATSVRVSNELGASHPKVAKL

SVLVVNTNSIIISIFFSAIILIFKVGLSKLFTNDAEVIEAVSNLTPLLAISVFLNGIQPILSGVAIGSGW

QAIVAYVNLATYYLIGLPIGCVLGFKTSLGVAGIWWGMIIGVLLQTVTLIILTARTDWNAEVSKAAERLR

NSANVENLNLLEDV

>SbMATE

MEEHRSPAHAKPEAEQPPQQQVPAAMAVAVAVDVAAPAALQNSTAAPAENGDVAAAGAAENGTAASAANG

DGGGSELLGGPRWTGLHLFVMNIRSVFKLDELGAEVLGIAVPASLALTADPLASLIDTAFIGRLGSVEIA

AVGVAIAVFNQVMKVCIYPLVSVTTSFVAEEDAVLSKGGAKVIDNGEEEEELEAGQVGPEKHTAAAGADP

EKQQQPADEEAAKNGGEGCAPAVVAGRSSGKKSGNRRFVPSVTSALIVGALLGLFQTVFLVAAGKPLLRL

MGVKPGSPMVMPALRYLTLRALGAPAVLLSLAMQGVFRGFKDAKTPLYAIVAGDAANIVLDPILIFGCRL

GVIGAAIAHVLSQYLITLIMLSKLVRKVDVVPPSLKCLKFRRFLGCGFLLLARVVAVTFCVTLAASLAAR

HGPTAMAAFQICTQVWLATSLLADGLAVAGQAMIASAFAKEDRYKVAATAARVLQLGVVLGAALTALLGL

GLQFGAGVFTSDAAVIKTIRKGVPFVAGTQTLNTLAFVFDGINFGASDYAFSAYSMIGVAAVSIPSLIFL

SSHGGFVGIWVALTIYMGVRALASTWRMAAAQGPWKFLRQ

>GmMATE75

MDENRSSNEPNKWKMPLFVFFKDARNVFKLDALSREILGIALPSALAVSADPIASLIDTAFIGRLGPVEL

AAAGVSISLLNQASRITIFPLVNITTSFVAEEDTIQKLNTKAAENGNSKAKFGETIVPEDHMLQDMEKGT

PKVMNTDAPTEFREDKDESQEYNATGNNDTNIGDGANTICKFSSVTSSKKSKDKVGKKKRLIASASTALL

FGTILGLIQAAVLIFATKPLLGVMGVKRDSPMLKPAESYLRLRSFGAPAVLLSLAMQGIFRGFKDTTTPL

YVIVSGYALNVILDPIFIFTLKLGIKGAAIAHVLSQYMMAFTLLLILMKKVHLLPPRIKDLQIFRFLKNG

GLLMLKVIAVTFCVTLATSLAARLGSIPMAAFQTCLQVWMTSSLLADGLAVAVQAILACSFTEKDYKKQQ

QQQQGHCK

>ELS1

METPNIISHTNLLSKIDLEKQNPAPIFPTITELKSEARSLFSLAFPTILAALILYARSAISMLFLGHIGE

LELAGGSLAIAFANITGYSVLAGLALGMDPLCSQAFGAGRPKLLSLTLQRTVLFLLTSSVVIVALWLNLG

KIMIYLHQDPSISSLAQTYILCSIPDLLTNSFLHPLRIYLRAQGITSPLTLATLAGTIFHIPMNFFLVSY

LGWGFMGVSMAAAASNLLVVIFLVAHVWIAGLHQPTWTRPSSECFKDWGPVVTLAIPSCIGVCLEWWWYE

IMTVLCGLLIDPSTPVASMGILIQTTSLLYIFPSSLGLAVSTRVGNELGSNRPNKARLSAIVAVSFAGVM

GLTASAFAWGVSDVWGWIFTNDVAIIKLTAAALPILGLCELGNCPQTVGCGVVRGTARPSMAANINLGAF

YLVGTPVAVGLTFWAAYGFCGLWVGLLAAQICCAAMMLYVVATTDWEKEAIRARKLTCTEGVDVVITTTQ

TNGDLSEPLIYVVTVATD

>OsFRDL2

MNGESLLDRSSSADAAMNDAVPGHRHHHPLSVFLRDARLAFRWDELGREIMGIAVPGALALMADPVASLV

DTAFIGHIGPVELAAVGVSIAVFNQVSRIAIFPLVSVTTSFVAEEDATSSDREKYEINGENEFNVSDSEM

EELVSHEEASAAPSKSSFETDSSDVKIEHKRKNIPSVSTALLLGGVLGLLQALLLVICAKPLLGYMGVKQ

GSAMLMPALKYLVVRSLGAPAVLLSLAMQGVFRGLKDTKTPLYATVAGDATNIVLDPIFMFVFQYGVSGA

AIAHVISQYFIASILLWRLRLHVDLLPPSFKHMQFSRFLKNGFLLLARVIAATCCVTLSASMAARLGSVP

MAAFQICLQIWLASSLLADGLAFAGQAILASAFARQDHSKAAATASRILQLGLVLGLLLSIFLGIGLRLG

SRLFTDDQDVLHHIYLGIPFVSLTQPINALAFVFDGINYGASDFGYAAYSMILVAIVSIIFIVTLASYNG

FVGIWIALTVYMSLRMLAGFLRIGTARGPWTFYAAQRMHSHEVVGLC

>TaMATE1

MEEGAAASMTVGEKRVAVDVPAGAAAAANGHGPEEKAAEDVPAPSALSGWPRTTGMYLFVMNIRSVFKLD

ELGSEVLRIAVPASLALAADPLASLVDTAFIGRLGSVEIAAVGVSIAIFNQVSKVCIYPLVSVTTSFVAE

EDAIISKYIEENNSKDLEKAAHVHSDACNVPASGGDTPVCANSCIPTECADPSNQGCKRRYIPSVTSALI

VGSFLGLVQAVFLIFSAKVVLGIMGVKHDSPMLEPAVRYLTIRSLGAPAVLLSLAMQGVFRGFKDTKTPL

YATVVGDATNIILDPILMFVCHMGVTGAAVAHVISQYLITMILICRLVQQVDVIPPSLKSLKFGRFLGCG

FLLLARVVAVTFCVTLASSLAARDGPTIMAAFQICCQLWLATSLLADGLAVAGQAVLASAFAKNDTKKVI

AATSRVLRLSIVLGMGLTVVLGLFMKFGAGVFTKDAAVIDVIHKGIPFVAGTQTINALAFVFDGINFGAQ

DYTYSAYSMVGVASISIPCLVYLSAHRGFIGIWVALTIYMSLRTVASTWRMGAARGPWAFLRK
